# Process-Ready Nickel-Catalyzed Suzuki–Miyaura Coupling Enabled by *tri*-ProPhos

**DOI:** 10.1021/acscatal.5c07157

**Published:** 2025-11-06

**Authors:** Jin Yang, Hengyuan Zhao, Johnathan E. Schultz, Steven R. Wisniewski, Eric M. Simmons, Tianning Diao

**Affiliations:** † Department of Chemistry, 5894 New York University, 100 Washington Square East, New York, New York 10003, United States; ‡ Chemical Process Development, Bristol Myers Squibb Company, New Brunswick, New Jersey 08903, United States

**Keywords:** Suzuki–Miyaura, nickel, ProPhos, green-solvent, heterocycles, pharmaceutical process synthesis

## Abstract

The synthesis of active pharmaceutical ingredients (APIs) containing heteroaromatic motifs often relies on palladium-catalyzed Suzuki–Miyaura coupling (Pd-SMC), a transformation that can account for a significant portion of the production costs for small-molecule drugs. Nickel-catalyzed SMC offers a more compelling alternative; however, its large-scale implementation has been hindered by high catalyst loadings and a limited scope of heterocyclic coupling partners. Another unmet need in process synthesis is the adoption of polar solvents, such as alcohols and water, to reduce waste generation, improve safety, and improve compatibility with hydrophilic molecules. Here, we introduce a (*tri*-ProPhos)Ni catalyst that enables efficient and robust Ni-SMC of heterocycles in *i*-PrOH and water. The *tri*-ProPhos ligand features a phosphine moiety tethered to three hydroxyl groups, which can substitute the halide in the oxidative addition intermediate to form a nickel-alkoxy species. This pathway not only facilitates transmetalation but also enhances catalyst stability. Moreover, the hydrophilic nature of the ligand allows Ni-SMC to be performed in pure water. The (*tri*-ProPhos)Ni catalyst accommodates a wide range of heteroaromatic core structures, including those present in APIs, with catalyst loadings as low as 0.03–0.1 mol %. The method has been validated on decagram scale and represents a versatile platform with significant potential for adoption in commercial process synthesis.

## Graphical abstract

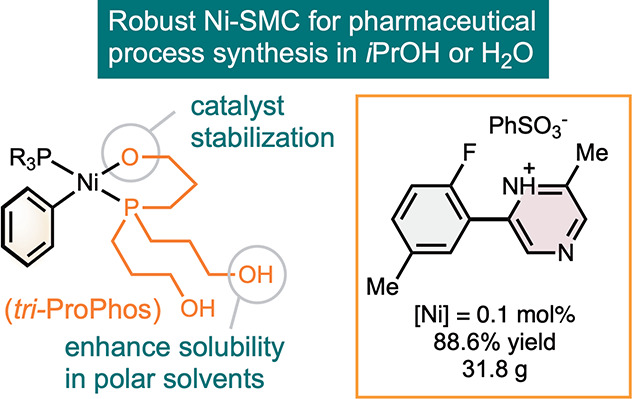

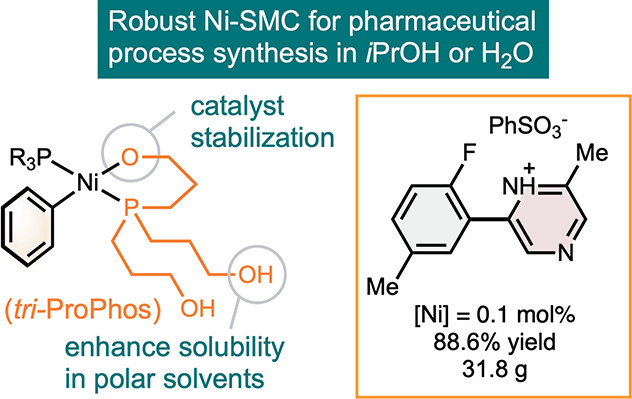

## Introduction

Heterocycles are prevalent in commercial small-molecule drugs, with around 82% of the new compounds approved by the FDA between 2013 and 2023 containing at least one heterocyclic ring.^1^ Their widespread use is attributed to the versatility of heterocycles to engage in noncovalent interactions with biological targets and impart conformations of substituents, thereby enhancing target affinity and specificity. In modern pharmaceutical process synthesis, heteroaromatic frameworks are commonly constructed using palladium-catalyzed Suzuki–Miyaura coupling (Pd-SMC), which couples electrophilic (hetero)­aryl, (pseudo)­halides with (hetero)­aryl boron nucleophiles (Scheme 1A).^2,3^ Between 2017 and 2021, palladium catalysis accounted for over 80% of all catalytic reactions performed in process synthesis, with 35% dedicated to Pd-SMC for biaryl and (hetero)­biaryl bond formation.^4,5^ However, the commodity cost of palladium catalysts has fluctuated significantly in recent years, typically averaging around 3,600 USD/mol, which, coupled to the stringent pharmaceutical standards for removing residual heavy metals from active pharmaceutical ingredients (APIs),^6^ can contribute substantially to the overall expense of drug production.

**Scheme 1:**
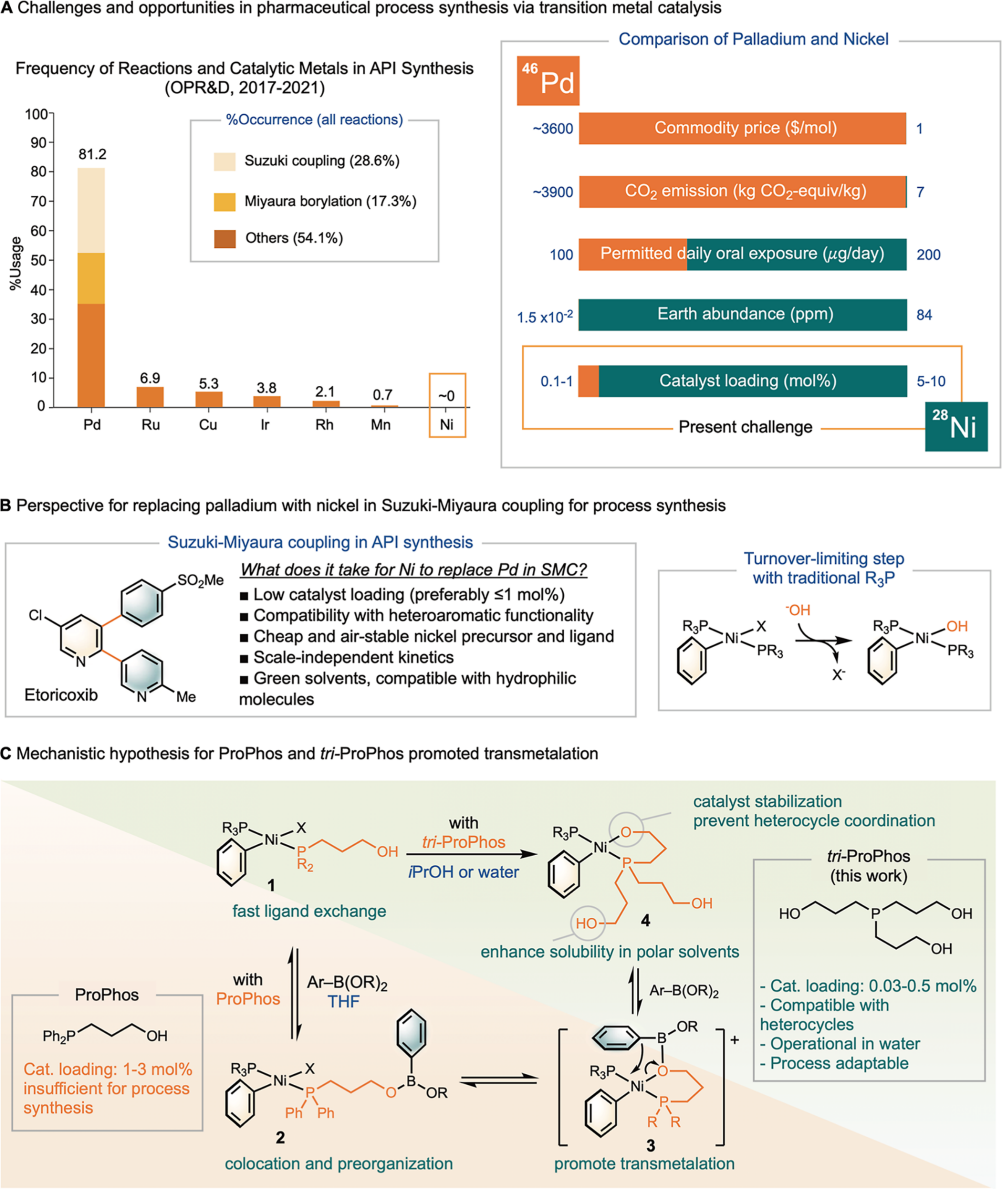
Overview of Challenges and Opportunities in Ni-SMC for Process Synthesis and Design Principle of ProPhos and *tri*-ProPhos

Compared to Pd-SMC, nickel catalysts offer a more compelling alternative due to their lower cost, higher tolerance for residual metal levels,^7^ greater earth abundance, and lower environmental impact (Scheme 1A).^8–10^ However, to date, only one commercial process utilizing Ni-SMC has been reported, in the synthesis of pictilisib.^11^ While this example highlights the potential of Ni-SMC in process synthesis, significant challenges continue to limit its broader application.^12,13^ Key limitations include the frequent need for high catalyst loadings (5–10 mol %),^14^ slow reaction rates,^15^ reliance on air-sensitive and expensive precatalysts and ligands,^16^ limited heteroarene scope, and poor catalyst stability.^17^ Despite extensive research efforts, including high-throughput screening both in academia and industry,^18^ no catalyst has yet been identified that effectively addresses all of these challenges.

For Ni-SMC to be adoptable in process synthesis, an ideal nickel catalyst must incorporate an air-stable, cost-effective precatalyst and ligand, operate with low catalyst loadings (preferably ≤1 mol %), and accommodate a broad range of heteroaryl functionality in both the nucleophile and electrophile coupling partners (Scheme 1B).^14^ State-of-the-art Ni-SMC systems typically utilize either monodentate or bidentate phosphine ligands. Monodentate ligands often provide high reactivity but suffer from low functional group tolerance and poor catalyst stability,^19–24^ whereas bidentate ligands improve catalyst stability but frequently compromise reactivity.^25–27^ Although higher temperatures can accelerate reaction rates, they also increase the risk of protodeboronation.^28,29^ Our previous study on (Ph_2_MeP)­Ni-catalyzed SMC^22^ revealed that the turnover-limiting step involves substitution of the halide with hydroxide on the nickel-aryl-halide intermediate, forming a “nickel-oxo” species from which transmetalation proceeds rapidly (Scheme 1B).^30^ However, attempts to promote this ligand substitution by applying strong bases could lead to catalyst deactivation by forming unreactive [Ni­(OR)_2_]_*n*_ oligomers.^31^

Another unmet need in process synthesis is the adoption of green solvents. Solvents typically account for 80–90% of the total mass used in pharmaceutical batch chemical operations and contribute substantially to waste generation.^32^ The use of nonflammable and noncombustible solvents is also preferred for safety.^12^ Among all solvents, water is ranked as the most ideal in terms of cost, safety, and sustainability.^33^ However, most transition metal catalysts, particularly those based on earth-abundant base metals, are poorly soluble and often incompatible with polar solvents like water. Beyond its sustainability benefits, water can also effectively solubilize hydrophilic molecules, which are often difficult to handle in petroleum-based nonpolar solvents.

Recently, our lab developed a scaffolding ligand, ProPhos (diphenylphosphino)­propanol, which features a hydroxyl group tethered to the phosphine moiety.^30^ This hydroxyl group coordinates to the boronic acid or ester (**1**→**2**) prior to transmetalation (**2**→**3**), promoting colocation of the catalyst and the boronate substrates (Scheme 1C, orange pathway). While ProPhos significantly improved the reactivity of Ni-SMC with heterocyclic substrates, it still required catalyst loadings of 1–3 mol % in THF/water, which remains higher than those typically achieved with palladium catalysts and is still suboptimal for industrial adoption.^30^

To address the need for more robust nickel catalysts suitable for large-scale applications in green solvents, we developed a new ligand, *tri*-ProPhos, by incorporating three tethered propyl alcohol groups on to the phosphine. We hypothesized that the increased number of hydroxyl groups would improve catalyst stability by enhancing the probability of chelation. While one hydroxyl group can coordinate to nickel, the remaining two serve as hydrophilic arms to enhance solubility in polar green solvents, such as alcohols and water. Furthermore, the presence of three tethered hydroxypropyl groups may provide additional stabilization through a statistically a higher chance of chelation to the nickel center.

Herein, we report that *tri*-ProPhos enables Ni-SMC at catalyst loadings as low as 0.03 mol % in green solvents, including *i*-PrOH and water. We propose that in polar media, (*tri*-ProPhos)­Ni-SMC proceeds through an alternative pathway in which one hydroxyl group of *tri*-ProPhos displaces the halide of intermediate **1** (**1**→**4**, Scheme 1C, green pathway). This intramolecular ligand substitution is expected to accelerate transmetalation due to a lower activation entropy (Δ*S* ^‡^) and a more favorable entropic driving force (Δ*S*). The formation of intermediate **4**, in which all three hydroxyl groups available for chelation, further stabilize the catalyst and helps prevent deactivation by heterocyclic substrates. These combined effects result in a highly efficient and robust catalytic system with strong potential for adoption in industrial-scale, sustainable process synthesis.

## Methods

The SMC of electron-rich 3-pyridinyl boronic acid **6** is notoriously challenging due to its strong tendency to coordinate to the metal center, leading to catalyst poisoning.^22^ The few reported examples of Ni-SMC of **6** require catalyst loadings of up to 10 mol % and still result in unsatisfactory yields.^34,35^ To benchmark performance, we selected the coupling of **6** with aryl bromide **5** as a model reaction. We compared ProPhos and *tri*-ProPhos with commonly used bidentate (saffron bars) and monodentate (canary bars) phosphine ligands, including PPh_2_Me^22^ and dppf (dppf = 1,1′-bis­(diphenylphosphino)­ferrocene),^16,36^ both previously identified as effective ligands for SMC. We evaluated ligand performances using 1 mol % NiCl_2_·6H_2_O ($21/mol) as the precatalyst and 2.5 equiv of K_3_PO_4_ as the base (Scheme 2A).

**Scheme 2:**
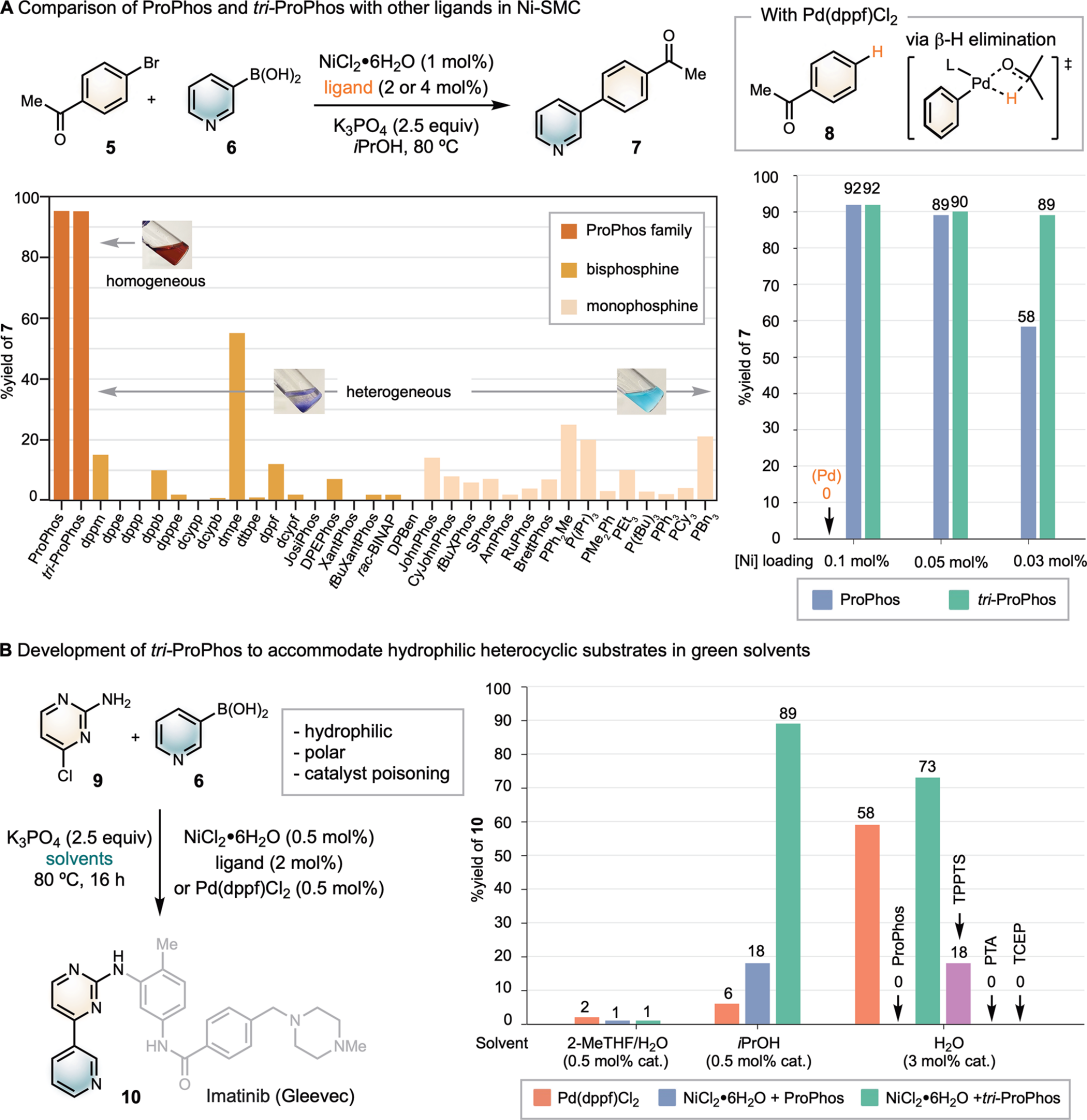
Comparison of *tri*-ProPhos with Previous Ligands/Catalysts

In *i*-PrOH, both ProPhos and *tri*-ProPhos fully solubilized NiCl_2_·6H_2_O (Scheme 2A, orange bars) and their use in Ni-SMC yielded product **7** in ≥95% yield after 6 h. In contrast, most common monodentate and bidentate phosphine ligands resulted in heterogeneous catalyst mixtures (saffron and canary bars, cf. Table S2), leaving NiCl_2_·6H_2_O largely undissolved. These reactions gave only low to modest yields of **7**, highlighting the superior reactivity of ProPhos and *tri*-ProPhos under these conditions. We next evaluated the performance of ProPhos and *tri*-Prophos at lower catalyst loadings over 16 h. At 0.1 and 0.05 mol %, both ligands maintained high efficiency, affording **7** in ≥90% yields. In contrast, Pd­(dppf)­Cl_2_ gave no detectable product and instead led to protodehalogenation of **5**, forming acetophenone **8**, likely through β-H elimination from *i*-PrOH followed by reductive elimination. This contrast highlights the superior selectivity of nickel in alcohol solvents, which may be attributed to its inherently slower β-H elimination rate.^37^ At 0.03 mol % catalyst loading, the yield with ProPhos decreased significantly, while *tri*-ProPhos maintained a high yield of 89%. Comparison of the timecourse revealed that although ProPhos gave a faster initial rate, catalyst deactivation led to a plateau at 50% conversion (Figure S10). In contrast, the reaction with *tri*-ProPhos proceeded to full conversion without noticeable catalyst deactivation, even at this low loading.

We next expanded our investigation to the SMC of 3-pyridinyl boronic acid **6** and 2-amino-4-chloropyrimidine **9**, affording (hetero)­biaryl **10**, a synthetic intermediate to the leukemia drug imatinib (Gleevec). This SMC is particularly challenging: in addition to the potential catalyst poisoning by **6**, electrophile **9** is highly polar and poorly soluble in most organic solvents. Moreover, the chloride atom of **9** is attached to the same carbon as one of the nitrogen atoms of the heterocycle, a motif prone to protodehalogenation and catalyst deactivation via κ-2 coordination from the 2-aminopyrimidine moiety.^38^ As a result, conducting the SMC in the standard 2-MeTHF/H_2_O solvent mixture gave only minimal product formation (Scheme 2B). Switching to *i*-PrOH as the solvent, both (ProPhos)­NiCl_2_ and Pd­(dppf)­Cl_2_ gave low yields of **10**. In contrast, using *tri*-ProPhos with NiCl_2_·6H_2_O achieved an 89% yield with just 0.5 mol % nickel loading. Notably, the reaction was also successful in pure water, where *tri*-ProPhos again outperformed Pd­(dppf)­Cl_2_ at the same catalyst loading, while ProPhos was not effective. In aqueous media, the reaction with *tri*-ProPhos formed a homogeneous biphasic mixture: the catalyst, base, and hydrophilic substrates were completely dissolved in the aqueous phase, while the hydrophobic substrates formed a thin layer of organic phase. This characteristic is important for scalability and process synthesis, where solid–liquid biphasic and solid–liquid–liquid triphasic mixtures pose a number of challenges for scale up.^39^ Applying ProPhos and other common water-soluble phosphine ligands in Ni-SMC, such as triphenylphosphine-3,3′,3″trisulfonic acid trisodium salt (TPPTS), 1,3,5-triaza-7-phosphaadamantane (PTA), and tris­(2-carboxyethyl)­phosphine hydrochloride (TCEP), resulted in no to minimal yield of **10** (cf. Scheme S3).

We next explored the application of (*tri*-ProPhos)­Ni in promoting Ni-SMC of hydrophilic heterocyclic coupling partners at low catalyst loadings in *i*-PrOH and water (Scheme 3). The system proved compatible with a variety of electrophiles, including (hetero)­aryl bromides and chlorides, and nucleophiles such as (hetero)­aryl boronic acids, esters, and trifluoroborates. Notably, aryl chlorides (**11**–**13**), which are typically more cost-effective and widely available than aryl bromides, were successfully employed.^40,41^ The coupling with potassium trifluoroborate nucleophiles (**13**), highly relevant in medicinal chemistry, highlights the capability of (*tri*-ProPhos)Ni compared to ProPhos.^30^ This system also enabled efficient coupling of *ortho*-substituted, unprotected aniline (**14**), a known challenging substrate. Interestingly, the synthesis of **14** in water outperformed that in *i*-PrOH, possibly due to improved solubility of polar substrates and hydrogen-bonding interactions with aniline, which may mitigate catalyst poisoning. The broad functional group tolerance was demonstrated by successful couplings involving 2-, 3- and 4-functionalized pyridines (**7**, **11**, **12**, **17**, **20**, and **25**), pyrimidines (**12** and **19**), pyrazoles (**15**), indoles (**16**), sulfones (**17**), carboxylic acids (**19**), esters (**20**, **23** and **24**), and amides (**21**, **23** and **24**). In most cases, high yields were achieved with catalyst loadings as low as 0.05–0.1 mol % in *i*-PrOH. Reactions in water generally required higher catalyst loadings, particularly when using electron-rich nucleophiles susceptible to protodeboronation.

**Scheme 3:**
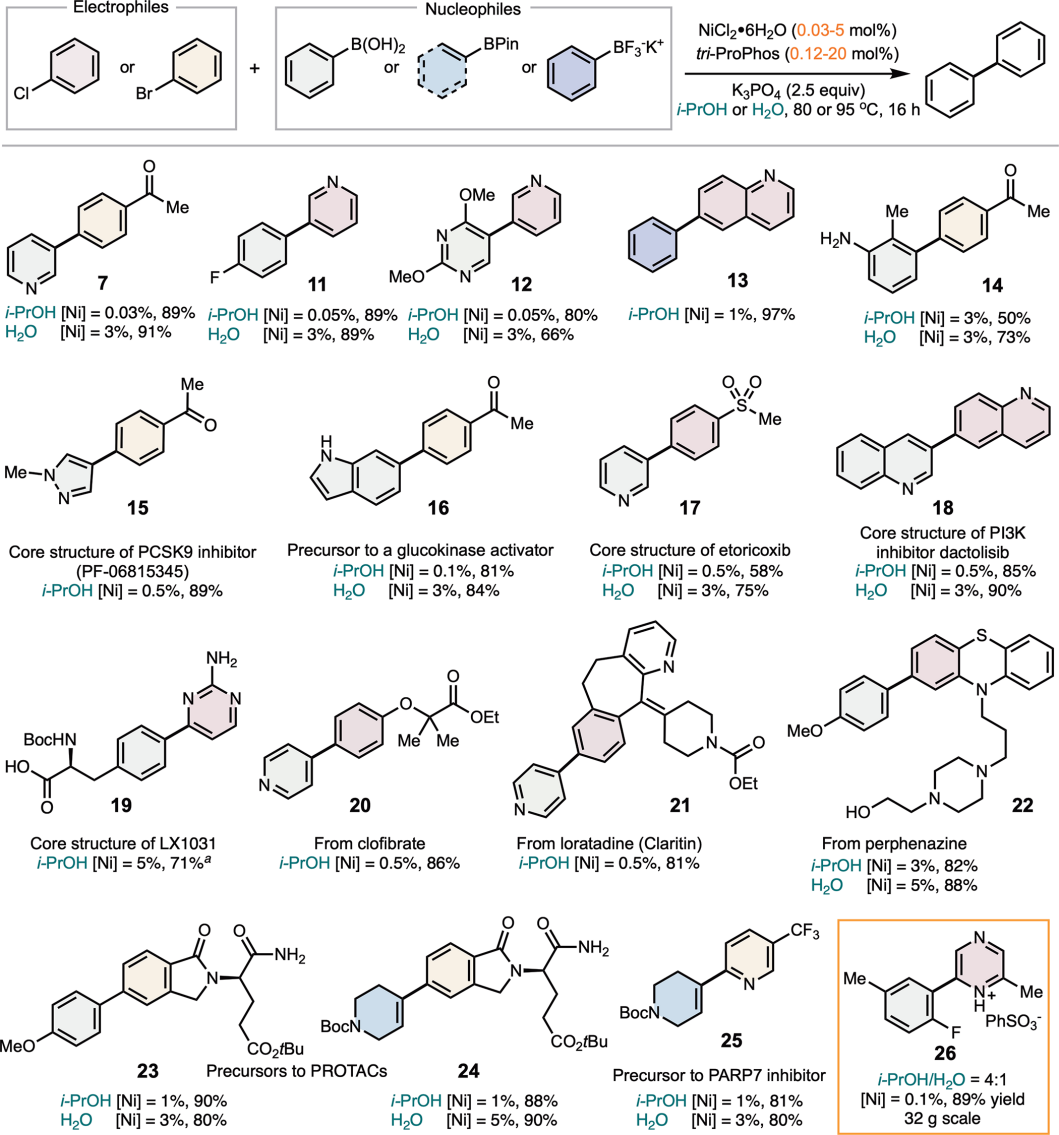
Scope of Ni-SMC Facilitated by *tri*-ProPhos^a^

a General conditions: electrophile (0.1–0.2 mol, 1 equiv), nucleophile (1.5 equiv). Isolated yields unless otherwise noted. ^*a*^Product **19** is highly polar. The yield was determined by NMR using 1,3,5-trimethoxybenzene as the internal standard and product standard obtained from preparative HPLC.

Many of the heteroaromatic Ni-SMC products shown in Scheme 3 represent core structures of APIs or synthetic intermediates or derivatives related to commercial drugs (**15**–**25**). These include scaffolds found in the PCSK9 inhibitor PF-06815345 (**15**),^42^ a glucokinase activator (**16**),^43^ etoricoxib (**17**),^44^ the PI3K inhibitor dactotisib (**18**),^45^ and the tryptophan hydroxylase inhibitor (LX1031) (**19**).^46^ Several examples highlight the robustness of the method: the synthesis of **15** employs a five-membered Lewis-basic arylboronic acid, typically challenging in Ni-SMC;^22^ **17** contains sulfone, a polar and oxidizing functionality previously unexplored in Ni-SMC; and **19** includes both an amino acid and a 2-aminopyrimidine moiety, both of which can strongly coordinate to and deactivate catalyst.

We also demonstrated the utility of (*tri*-ProPhos)­Ni-SMC in the late-stage functionalization of chloride-containing drug molecules, including clofibrate (**20**), loratadine (**21**), and perphenazine (**22**), which gave the corresponding products in 81–88% yields. Notably, while 4-pyridinylboronic acid remains a particularly challenging nucleophile,^22^ even for Pd-SMC, it was readily engaged in (*tri*-ProPhos)­Ni-SMC to deliver **21** in 81% yield with only 0.5 mol % nickel. In contrast, a recent report using a Pd-Negishi reaction achieved a 58% yield for **21** with 0.5 mol % Pd catalyst.^47^ Additionally, 4-arylpiperidine derivatives are widely used as drug precursors and are typically synthesized via Pd-SMC.^48^ We successfully prepared **24**–**25** using (*tri*-ProPhos)­Ni-SMC. Notably, prior syntheses of **24** ^49^ and **25** ^50^ required palladium loadings as high as 10 mol %.

Finally, we validated the scalability of the (*tri*-ProPhos)­Ni-SMC system by synthesizing pyrazine derivative **26** on a 30 g scale. Pyrazine is a privileged scaffold in pharmaceutical compounds due to its unique electronic properties and hydrogen-bonding capabilities.^51^ The nucleophile bears an *ortho*-fluoro substituent, a class of substrates susceptible to rapid protodeboronation.^52^ For this scale-up, we learned that a mixed solvent system of *i*-PrOH and water (4:1) was required to enable a robust scale-up, which accommodated a low catalyst loading of 0.1 mol %, improved the stability of the boronic acid, and enabled full solubilization of the base, affording a homogeneous biphasic reaction mixture upon heating. The reaction proceeded to full conversion, and compound **26** was isolated in 89% yield on a 32 g scale through a direct-drop crystallization as the benzenesulfonate salt.

We conducted mechanistic studies to elucidate the origins of the exceptional performance of *tri*-ProPhos in Ni-SMC of heterocycles in polar solvents, with respect to both reactivity and compatibility. In a mixed solvent of *i*-PrOH and water, the reaction mixture forms a homogeneous biphasic solution. By monitoring the reaction using GC, we profiled the reaction kinetics for a series of reactions with varying concentrations of substrates, catalysts, and bases (Scheme 4A). The reaction shows an initial induction period, which we attribute to catalyst activation of the complex initially formed from *tri*-ProPhos and NiCl_2_·6H_2_O as well as the solubilization of K_3_PO_4_. We then used the Variable Time Normalization Analysis (VTNA) to derive the rate law (Figures S6–S9).^53^ The reaction exhibits a first-order dependence on [catalyst] and [base], but no dependence on the concentration of either substrate. When replacing *i*-PrOH with the perdeuterated *i*-PrOH-*d*_8_, the reaction rate was significantly slower compared to that in *i*-PrOH, resulting in a primary kinetic isotope effect (KIE) of 2.9 (Figure S11), as determined by independent rate measurements of parallel reactions.

**Scheme 4:**
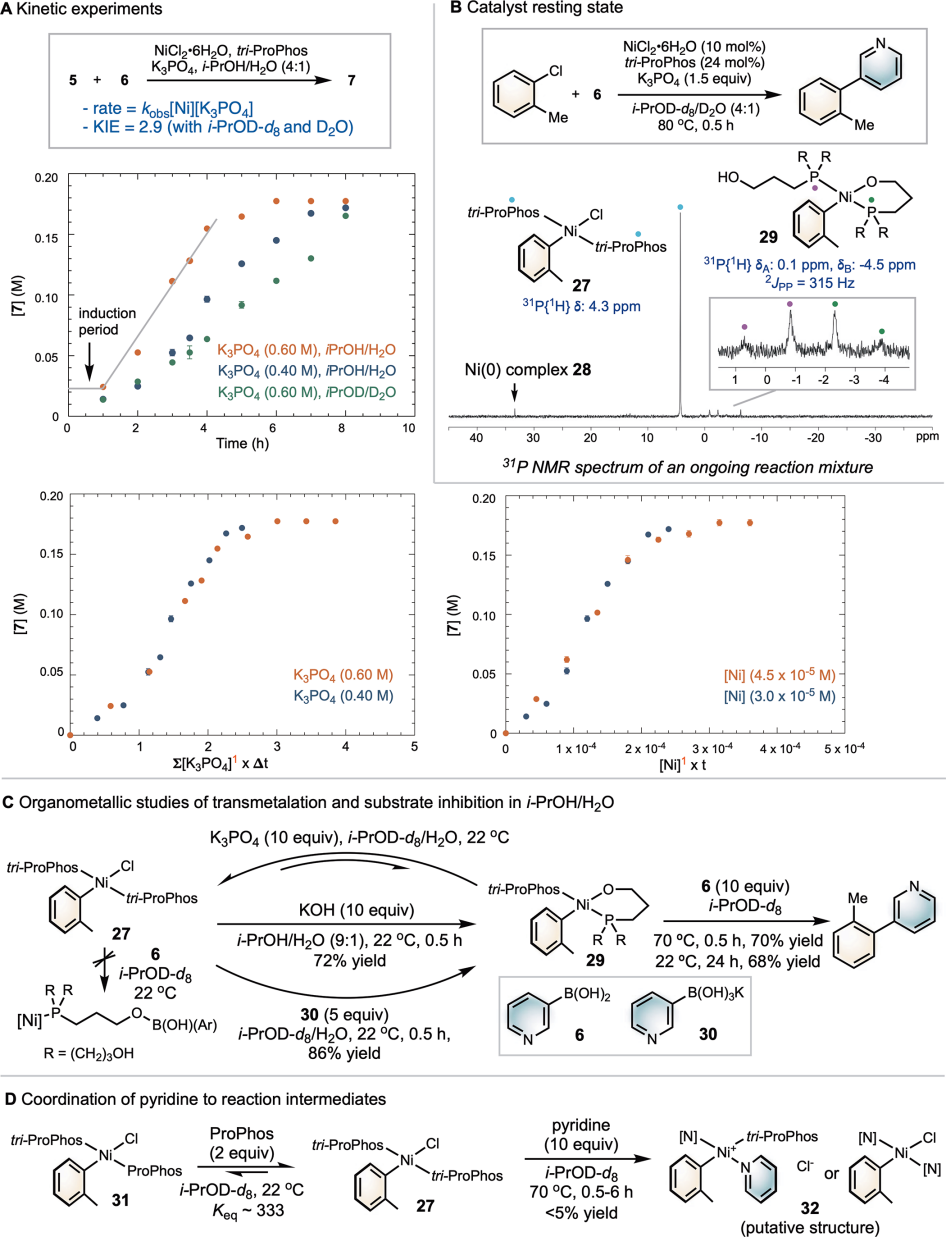
Mechanistic Studies of (*tri*-ProPhos)­Ni-SMC of Heterocycles in *i*-PrOH

Monitoring the catalytic reaction of *ortho*-tolyl chloride with boronic acid **6** in 4:1 *i*-PrOH/water by ^31^P­{H} NMR spectroscopy revealed a major singlet at 4.3 ppm, consistent with the independently synthesized (*tri*-ProPhos)_2_Ni­(*o*-Tol)Cl **27** (Scheme 4B). These data suggest that (*tri*-ProPhos)_2_Ni­(*o*-Tol)Cl **27**, formed via oxidative addition, is likely the catalyst resting state. In addition to the major species, minor resonances were observed in solution, including a singlet at 33 ppm and an AB quartet at 0.1 and −4.5 ppm with a coupling constant of 315 Hz. These species are assigned to (*tri*-ProPhos)_*n*_Ni­(0) **28** and (*tri*-ProPhos)­(κ^2^-*tri*-ProPhos)­Ni­(*o*-Tol) **29**, respectively, based on independent synthesized complexes (cf. Figures S1–S5).

The addition of boronic acid **6** to a solution of (*tri*-ProPhos)_2_Ni­(*o*-Tol)Cl **27** in *i*-PrOH-*d*_8_ resulted in no evidence of coordination between **6** and the hydroxyl group of *tri*-ProPhos (Scheme 4C, cf. Figure S15). This contrasts with our previous observations with ProPhos in THF/C_6_D_6_,^30^ suggesting a different mechanism is operative with *tri*-ProPhos in *i*-PrOH. When **27** was mixed with K_3_PO_4_ in *i*-PrOH-*d*_8_/H_2_O at 22 °C, a small portion of the complex was converted into (*tri*-ProPhos)­(κ^2^-*tri*-ProPhos)­Ni­(*o*-Tol) **29**, resulting in broad ^31^P NMR resonances (Scheme 4C, cf. Figure S19). In dry *i*-PrOH-*d*_8_, where KCl is insoluble, the ^31^P signals appeared sharp, showing two distinct signals corresponding to **27** and **29**, respectively (Figure S20). This result indicates rapid interconversion between **27** and **29** in the *i*-PrOH-*d*_8_/H_2_O solution. Upon treatment with the stronger base KOH, **27** was cleanly converted to **29** in high yield (72%). Notably, the addition of 10 equiv of boronic acid **6** to isolated **29** led to rapid formation of biaryl **10** in 70% yield within 30 min at 70 °C. To determine whether transmetalation could occur directly between **27** and a boronate species, we treated **27** with independently synthesized boronate **30** in *i*-PrOH/H_2_O (Figure S16). This resulted only in the formation of **29** in approximately 86% yield, with no evidence of transmetalation. These data rule out the boronate pathway and suggest that the formation of **29** is a required intermediate for productive cross-coupling.

We next investigated the coordination affinity of *tri*-ProPhos (Scheme 4D). Addition of 2 equiv of ProPhos to **27** resulted in minor ligand exchange, yielding a small amount of the mixed-ligand complex **31** (cf. Figure S25). The equilibrium constant suggests that *tri*-ProPhos binds more strongly to nickel than ProPhos. When 10 equiv of pyridine were added to **27**, only minor formation of the pyridine-bound complex **32** was observed, with the majority of **27** remaining intact. The same experiment with **29** showed no evidence of pyridine coordination (Figure S22). These results indicate that *tri*-ProPhos binds tightly to nickel and can effectively outcompete pyridine for coordination. This strong ligand affinity likely underlies the system’s broad functional group tolerance and resistance to catalyst deactivation by Lewis basic heterocycles.

Collectively, the mechanistic data align with the catalytic cycle depicted in Scheme 5. The fast oxidative addition of aryl halides to **28** forms intermediate **27**, which is identified as the catalyst resting state. In the presence of base, one of the tethered hydroxyl groups of *tri*-ProPhos displaces the halide with concomitant deprotonation, forming the chelating intermediate **29**. This intermediate is analogous to the ″nickel-oxo” ^54,55^ and “palladium-oxo” species^56–58^ proposed and observed in previous studies. The formation of **29** is turnover-limiting and is promoted by the base, consistent with the first-order dependence on [catalyst] and [base], as well as the primary KIE of the *i*-PrOH solvent. The subsequent transmetalation (**29**→**34**) is rapid, which may be accompanied by the dissociation of one molecule of *tri*-ProPhos (**29**→**33**). The following steps—reductive elimination (**34**→**35**), hydrolysis of the boronic acid on *tri*-ProPhos, and the coordination of a second *tri*-ProPhos ligand to nickel—are all fast and their sequence may vary.

**Scheme 5:**
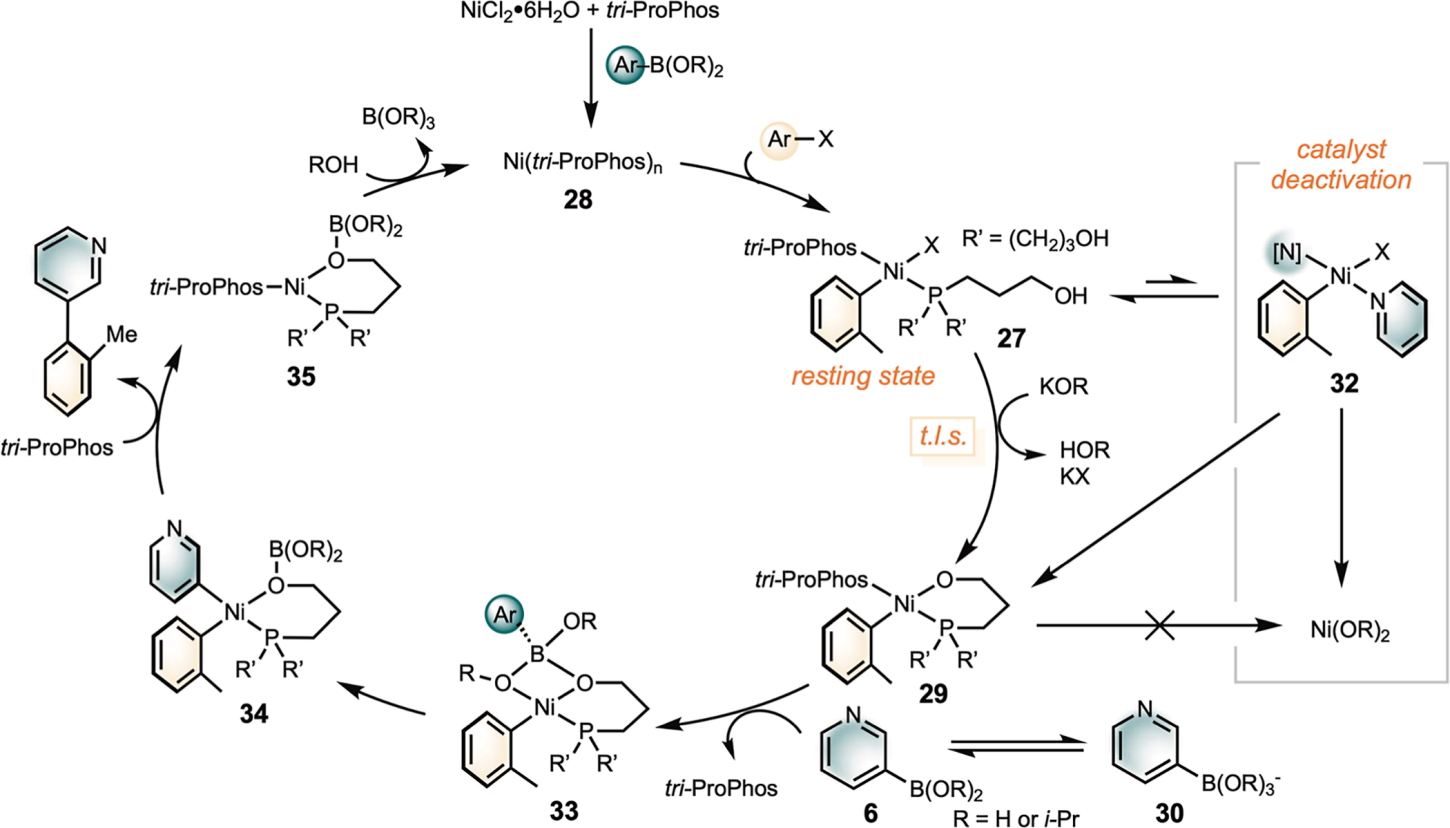
Proposed Mechanism for (*tri*-ProPhos)­Ni-SMC of Heterocycles in *i*-PrOH/H_2_O

The superior performance of *tri*-ProPhos in green polar solvents, compared to traditional monodentate and bidentate phosphine ligands, can be attributed to several factors. Unlike bidentate phosphines, the hydroxyl arm in *tri*-ProPhos is more labile, allowing for greater flexibility and facilitating ligand dissociation and oxidative addition. Compared to monodentate ligands, *tri*-ProPhos offers greater catalyst stability: the hydroxyl group prevent undesired coordination or ligand displacement by heterocyclic substrates, which could otherwise lead to the formation of off-cycle L_2_Ni­(Ar)­X species like **32** ^22^ or inactive [Ni­(OR)_2_]_*n*_ oligomers.^31^

The presence of three pendant hydroxyl groups renders *tri*-ProPhos significantly more effective than ProPhos, particularly at very low catalyst loadings. These hydroxyl groups enhance competitive binding over heterocyclic substrates, thereby ensuring broad substrate compatibility. Moreover, they provide multiple hydrogen-bonding sites that promote catalyst solubility in polar solvents such as *i*-PrOH and water. In these solvents, *tri*-ProPhos promotes the formation of the key “nickel-oxo” intermediate **29** through intramolecular ligand substitution (**27**→**29**). This pathway benefits from both an entropic driving force upon halide dissociation and a kinetic advantage conferred by the proximity of the pendant hydroxyl group to the nickel center. In this context, *i*-PrOH or water serves as a buffering solvent, modulating basicity to enable efficient deprotonation of ProPhos while preventing [Ni­(OR)_2_]_*n*_ aggregation and minimizing boronic acid decomposition that can occur in the presence of strong bases. This mechanism contrasts with our previous observations using ProPhos in THF.^30^ The combination of rapid transmetalation, enhanced catalyst solubility, and resistance to deactivation accounts for the high reactivity of (*tri*-ProPhos)­Ni.

## Conclusion

In summary, the development of *tri*-ProPhos has enabled highly efficient Ni-SMC of heteroaromatic substrates in green polar solvents, *i*-PrOH and water. This catalyst system employs the cost-effective and air-stable Ni­(II) precatalyst NiCl_2_·6H_2_O and facilitates robust synthesis of the core structures and synthetic intermediates of APIs at catalyst loadings ranging from 0.03 to 1 mol %. Considering the complexity of potential off-cycle pathways and the susceptibility to catalyst deactivation, the 100-fold reduction in catalyst loading with *tri*-ProPhos compared to the original ProPhos conditions highlights the remarkable efficiency and stability of these catalysts under optimized conditions. The high catalyst efficiency has been validated on decagram scale. Mechanistic studies reveal that in polar solvents, the hydroxyl group on *tri*-ProPhos ligands promotes intramolecular ligand substitution, leading to the formation of a chelating “nickel-oxo” intermediate that enables rapid transmetalation. Additionally, *tri*-ProPhos enhances precatalyst solubility and prevents deactivation caused by heterocycle coordination, while remaining hemilabile to facilitate substrate activation. This advancement addresses challenges associated with high catalyst loadings and limited functional group compatibility in base-metal catalysis, providing a more cost-effective and environmentally friendly alternative to current API syntheses. We anticipate that the *tri*-ProPhos will enable the routine implementation of base metal catalysis in large scale production and modernize pharmaceutical process synthesis.

## Supplementary Material



## Notes

All relevant data supporting the findings of this study are available in the main text and the Supporting Information. Detailed experimental procedures and characterization data for all newly synthesized compounds are provided in the Supporting Information (PDF)
