# Supplementary material for: Process-Ready Nickel-Catalyzed Suzuki–Miyaura Coupling Enabled by tri-ProPhos
Source: ACS Catal. 2025 Nov 6;15(22):19302–11. doi: 10.1021/acscatal.5c07157 (PMC12645473; doi:10.1021/acscatal.5c07157)
Supplement: Supplementary file 1 [file cs5c07157_si_001.pdf]

Supplementary Information

Process-Ready Nickel-Catalyzed Suzuki-Miyaura Coupling Enabled by *tri*-ProPhos

Authors: Jin Yang,[1] Hengyuan Zhao,[1] Johnathan E. Schultz,[2] Steven R. Wisniewski,[2] Eric M. Simmons[2] and Tianning Diao[1]*

Affiliations:

[1]Department of Chemistry, New York University, 100 Washington Square East, New York, NY 10003

[2]Chemical Process Development, Bristol Myers Squibb Company, New Brunswick, NJ 08903

*Correspondence to: diao@nyu.edu

Table of Contents

Table of Contents 2
1. Materials and Methods 3
2. Synthesis and Characterization of Phosphine Ligands and Organometallic Compounds 4
3. Screenings of Phosphine Ligands and Catalysts in Ni-SMC 14
4. Substrate Scope using (*tri*-ProPhos)Ni Catalysts 23
5. Kinetic Studies of Ni-SMC 42
6. Organometallic Studies 49
7. NMR Spectra 68
8. References 131

1. Materials and Methods

All air- and moisture-sensitive reactions and manipulations were performed under nitrogen in a glovebox or using conventional Schlenk techniques. Common solvents (e.g., THF) were dried and deoxygenated by passing through alumina in a solvent purification system. Deuterated solvents were purchased from commercial sources. Deuterated solvents used in the glovebox (e.g., *i*PrOD-d_8) were degassed using three freeze-pump-thaw cycles and stored over activated molecular sieve for 3 days before use. Unless otherwise specified, reagents and phosphine ligands were purchased from commercial sources (*vide infra*). The nickel precatalysts ($NiCl_2$•$6H_2O$ and $Ni(NO_3)_2$•$6H_2O$) and Pd(dppf)Cl_2 were purchased from commercial sources. Complex Ni(TMEDA)Cl(*o*-Tol) was prepared based on the literature.[1] Phosphine ligands and other organometallic compounds were obtained from commercial suppliers or prepared according to the methods described in Sections 2.

NMR spectra were recorded on a Bruker Avance 400 spectrometer (400.30 MHz for ^{1}H, 376.46 MHz for ^{19}F, 162.04 MHz for ^{31}P, 128.38 MHz for ^{11}B and 100.67 MHz for ^{13}C), and a Bruker Avance 500 spectrometer (500.20 MHz for ^{1}H, 470.61 MHz for ^{19}F, 202.49 MHz for ^{31}P, and 125.79 MHz for ^{13}C). Unless otherwise noted, chemical shifts were reported in ppm at room temperature. ^{1}H chemical shifts were referenced to residual proteo-solvent peak at 7.26 ppm ($CHCl_3$), 4.79 ppm (DHO), 3.31 ppm (CD_2HOD), 2.50 ppm ((CD_2H)S(O)(CD_3)) and 1.00 ppm (C_3D_7HOD); ^{13}C chemical shifts were referenced to 77.1 ppm ($CDCl_3$), 62.9 ppm (C_3D_7OD), 49.0 ppm (CD_3OD) and 29.8 ppm ((CD_3)$_2$SO). ^{1}H and ^{13}C chemical shifts were reported relative to tetramethylsilane (TMS), and ^{31}P chemical shifts were reported relative to 85% H_3PO_4 (aq.).

High resolution mass spectra (HRMS) were recorded on an Agilent 6224 TOF LC/MS (APCI source). GCMS data were obtained using a Shimadzu GC-2010 with a Shimadzu SH-Rxi-5Sil MS column. GC data for kinetic experiments and %yield determination were obtained using a Shimadzu GC-2010 Plus with a Restek Rxi-5MS column (L 15 m, ID 0.25, DF 0.25). UltiMate 3000 UHPLC system equipped with Luna 10u C18(2) 100A, AXIA column (250 x 21.2 mm) was used for some sample purifications.

2. Synthesis and Characterization of Phosphine Ligands and Organometallic Compounds

2.1 Modified synthesis of $PPh_2CH_2CH_2CH_2OH$ (ProPhos)

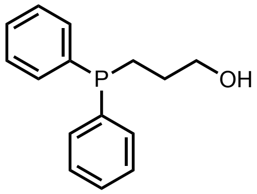

3-(Diphenylphosphino)-1-propanol (CAS: 2360-09-0). To a solution of diphenylphosphine (2.80 mL, 16.1 mmol, 1.0 equiv) and 3-chloropropanol (1.34 mL, 16.1 mmol, 1.0 equiv) in THF (20 mL), *n*-butyl lithium (14.2 mL, 2.50 M in hexanes, 35.5 mmol, 2.2 equiv) was added dropwise at 0 °C. The mixture was stirred for 1 h at room temperature. Afterward, deoxygenated NH_4Cl (aq., 40 mL) was added, and the mixture was evaporated. The residue was then extracted with ethyl acetate (EtOAc, 3 x 10 mL). The resulting suspension was filtered, and the solution was evaporated. The remaining residue was purified further by column chromatography using a solvent system of hexane/EtOAc (10:1) initially, followed by hexane/EtOAc (4:1), yielding the product (3.51 g, 14.4 mmol, 89%) as a white solid. The NMR data matched our previous reports.[2]

^{1}H NMR (500.20 MHz, $CDCl_3$) δ: 7.43 (td, *J* = 7.4, 2.2 Hz, 4H, H_o), 7.33 (tdd, *J* = 6.0, 4.4, 2.3 Hz, 6H, H_m &H_p), 3.71 (t, *J* = 6.4 Hz, 2H, OCH_2), 2.16 – 2.08 (m, 2H, PCH_2), 1.76 – 1.65 (m, 2H, CH_2), 1.50 (s, 1H, OH).

^{31}P{^{1}H} NMR (202.47 MHz, $CDCl_3$) δ: -16.2 (s).

^{13}C{^{1}H} NMR (125.79 MHz, $CDCl_3$) δ: 138.6 (d, *J* = 12 Hz, C_{ipso}), 132.8 (d, *J* = 18 Hz, C_o), 128.8 (s, C_p), 128.6 (d, *J* = 7 Hz, C_m), 63.7 (d, *J* = 14 Hz, CH_2O), 29.2 (d, *J* = 15 Hz, PCH_2), 24.4 (d, *J* = 12 Hz, CH_2).

LCMS (ESI-TOF, CH_3CN) m/z: $[M + H]^+$ calculated for $C_{15}H_{18}OP$ 245.11, found 245.11.

2.2 NMR characterization of $P(CH_2CH_2CH_2OH)_3$ (*tri*-ProPhos)

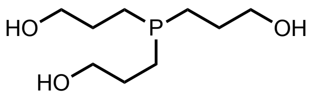

Tris(3-hydroxypropyl)phosphine (CAS: 4706-17-6). The commercial source indicated that the purity of this phosphine ligand exceeds 84%. To verify the actual purity and ensure the correct stoichiometry for catalytic reactions, NMR characterization was performed. The purity of this batch of compound was estimated to be 90% based on the $^{31}P\{^1H\}$ NMR analysis.

^1H NMR (500.20 MHz, CD_3OD) δ: 3.59 (t, *J* = 6.5 Hz, 6H, OCH_2), 1.71 – 1.60 (m, 6H, PCH_2), 1.51 – 1.41 (m, 6H, CH_2), OH signal not observed.

$^{31}P\{^1H\}$ NMR (202.47 MHz, CD_3OD) δ: -30.7 (s).

$^{13}C\{^1H\}$ NMR (100.67 MHz, CD_3OD) δ: 63.9 (d, *J* = 12 Hz, OCH_2), 30.0 (d, *J* = 13 Hz, PCH_2), 24.0 (d, *J* = 11 Hz, CH_2).

2.3 NMR characterization of $Ni(ProPhos)_2Cl(o\text{-}Tol)$ (S1) in *i*PrOH

Complex $Ni(ProPhos)_2Cl(o\text{-}Tol)$ was synthesized by using previously reported procedures.[2] To obtain a manageable solid product, we recommend performing multiple small-scale syntheses, as the high viscosity of free TMEDA can make larger-scale handling difficult.

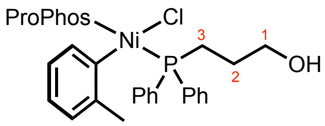

S1

^1H NMR (500.20 MHz, *i*PrOD-d_8) δ: 7.62 – 7.55 (m, 4H, H_o at Ph), 7.28 – 7.09 (m, 16H, H_o, H_m, H_p at Ph), 6.47 (d, *J* = 7.5 Hz, 1H, *o*-Tol), 6.37 (t, *J* = 7.2 Hz, 1H, *o*-Tol), 6.30 (t, *J* = 7.3 Hz, 1H, *o*-Tol), 6.17 (d, *J* = 7.3 Hz, 1H, *o*-Tol), 3.19 – 3.07 (m, 4H, H1), 2.43 (s, 3H, CH_3 at *o*-Tol), 1.78 – 1.62 (m, 4H, H3), 1.38 – 1.15 (m, 4H, H2, overlapped with some residual pentane). The OH signal is not observed in *i*PrOD-d_8, presumably due to the fast H/D exchange.

$^{31}P\{^1H\}$ NMR (202.47 MHz, *i*PrOD-d_8) δ: 14.5 (s).

$^{13}C\{^1H\}$ NMR (125.79 MHz, *i*PrOD-*d$_8$*) δ: 148.5 (s, *o*-Tol), 143.6 (s, *o*-Tol), 136.8 (s, *o*-Tol), 134.7 (t, *J* = 6 Hz, C_o at Ph), 133.4 (t, *J* = 5 Hz, C_o at Ph), 132.2 (t, *J* = 20 Hz, C_{ipso} at Ph), 131.7 (t, *J* = 20 Hz, C_{ipso} at Ph), 130.2 (s, C_p at Ph), 129.6 (C_p at Ph), 128.4 (t, *J* = 5 Hz, C_m at Ph), 128.2 (t, *J* = 4 Hz, C_m at Ph), 124.0 (s, *o*-Tol), 122.9 (s, *o*-Tol), 62.4 (t, *J* = 8 Hz, C1), 27.9 (s, C3), 26.3 (s, CH_3 at *o*-Tol), 23.0 (dt, *J* = 15.0 Hz, C2).

2.4 Synthesis of Ni(*tri*-ProPhos)$_2$Cl(*o*-Tol) (27)

A vial was charged with Ni(TMEDA)Cl(*o*-Tol) (50.0 mg, 0.166 mmol, 1.0 equiv), *tri*-ProPhos (82.9 mg, 0.398 mmol, 2.4 equiv) and THF (4 mL). The mixture was stirred at room temperature overnight, and the solvent was subsequently removed under vacuum to yield an orange oil. THF (2 mL) was added to the oil, and the suspension was filtered through Celite. The Celite was washed with THF (3 x 2 mL). The resulting orange filtrate was concentrated under vacuum, layered with pentane (5 mL), and stored at -35 °C overnight, yielding an orange crystal. The solid was further dried under vacuum for 5 h to afford a pale-yellow solid (56.1 mg, 0.0932 mmol, 56% yield).

The complex dissolves readily in alcohols like MeOH and *i*PrOH. It is also moderately soluble in neat water, though it gradually decomposes over time (*vide infra*). To obtain a manageable solid product, multiple small-scale syntheses were conducted, as the high viscosity of free TMEDA can make larger-scale handling difficult.

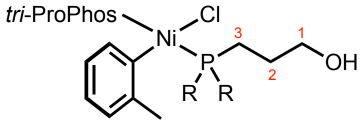

(R = $CH_2CH_2CH_2OH$)

27

^1H NMR (500.20 MHz, *i*PrOD-*d$_8$*) δ: 7.17 (d, *J* = 7.4 Hz, 1H, *o*-Tol), 6.62 (t, *J* = 5.5 Hz, 2H, *o*-Tol), 6.53 (t, *J* = 7.3 Hz, 1H, *o*-Tol), 3.40 (t, *J* = 6.3 Hz, 12H, H1), 2.77 (s, 3H, CH_3 at *o*-Tol), 1.72 – 1.58 (m, 12H, H3), 1.33 (dp, *J* = 11.1, 3.6 Hz, 12H, H2).

$^{31}P\{^1H\}$ NMR (202.47 MHz, *i*PrOD-*d$_8$*) δ: 4.3 (s).

$^{13}C\{^1H\}$ NMR (125.79 MHz, *i*PrOD-*d*$_8$) δ: 151.4 (s, *o*-Tol), 142.4 (s, *o*-Tol), 136.6 (s, *o*-Tol), 127.7 (s, *o*-Tol), 124.4 (s, *o*-Tol), 122.5 (s, *o*-Tol), 62.8 (t, *J* = 6 Hz, C1), 27.7 (s, C3), 27.3 (s, CH_3 at *o*-Tol), 19.2 (t, *J* = 12 Hz, C2).

HRMS (ESI-TOF, CH_3OH) m/z: $[M - Cl]^+$ calculated for $C_{25}H_{49}NiO_6P_2$ 565.2352, found 565.2370.

2.5 Synthesis of Ni(κ^2-PR$_2$CH$_2$CH$_2$CH$_2$O)(*tri*-ProPhos)(*o*-Tol) (29)

A vial was charged with Ni(*tri*-ProPhos)$_2$Cl(*o*-Tol) **27** (60.2 mg, 0.100 mmol, 1.0 equiv), KOH (56.1 mg, 1.00 mmol, 10 equiv), *i*PrOH (5 mL), and deoxygenated H_2O (0.5 mL). The mixture was stirred at room temperature for 30 min, and the solvent was subsequently removed under vacuum to yield a yellow oil containing a white solid. The mixture was extracted with *i*PrOH (3 × 3 mL) and filtered through Celite. The solvent of resulting yellow solution was removed under vacuum to give a yellow oily paste (40.5 mg, 0.0719 mmol, 72% yield).

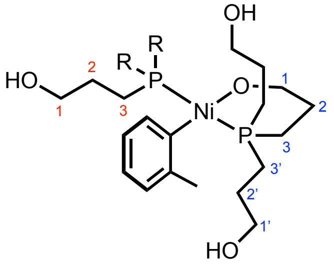

(R = $CH_2CH_2CH_2OH$)

29

^1H NMR (400.30 MHz, *i*PrOD-*d*$_8$) δ 7.14 (d, *J* = 7.4 Hz, 1H, *o*-Tol), 6.63 – 6.40 (m, 3H, *o*-Tol), 3.62 – 3.54 (m, 4H, H1’), 3.52 – 3.43 (m, 6H, H1), 3.24 – 3.05 (m, 2H, H1), 2.84 (s, 3H, CH_3 at *o*-Tol), 1.78 – 1.56 (m, *J* = 9.0 Hz, 14H, H3, H3 &H3’), 1.47 – 1.38 (m, 1H, H2), 1.36 – 1.16 (m, 8H, H2 & H2’), 1.11 – 1.05 (m, 1H, H2).

$^{31}P\{^1H\}$ NMR (162.04 MHz, *i*PrOD-*d*$_8$) δ: 0.1 (d, *J* = 315 Hz), -4.5 (d, *J* = 315 Hz).

$^{13}C\{^1H\}$ NMR (100.67 MHz, *i*PrOD-*d*$_8$) δ: 151.8 (dd, *J* = 39, 33 Hz, Ni-C_{ipso}), 142.6 (s, *o*-Tol), 136.9 (s, *o*-Tol), 126.6 (s, *o*-Tol), 123.5 (s, *o*-Tol), 121.5 (s, *o*-Tol), 62.3 (d, *J* = 11 Hz, C1), 62.0 (d, *J* = 15 Hz, C1), 29.8 (s, CH_2), 28.9 (d, *J* = 12 Hz, CH_2), 27.2 (s, CH_2), 25.8 (s, CH_2), 23.1 (d, *J* = 11 Hz, CH_2), 21.3 (d, *J* = 17 Hz, CH_2), 19.7 (d, *J* = 17 Hz, CH_2), 19.4 (d, *J* = 16 Hz, CH_2), 18.1 (d, *J* = 21 Hz, CH_2). One signal due to C1 was overlapped with solvent signals.

HRMS (ESI-TOF, CH_3CN) m/z: $[M + H]^+$ calculated for $C_{25}H_{49}NiO_6P_2$ 565.2352, found 565.2347.

*Characterization of Ni-metallacycle **29***

The most distinguishing feature between the isolated Ni-metallacycle complex, Ni(κ^2-$PR_2CH_2CH_2CH_2O$)(*tri*-ProPhos)(*o*-Tol) **29** and its parent complex Ni(*tri*-ProPhos)$_2$Cl(*o*-Tol) **27** lies in their $^{31}P\{^1H\}$ NMR spectra (Figure S1). Upon dehydrohalogenation, two distinct phosphine ligands in a Ni phosphine complex were expected to be observed two doublets. The notable large $^2J_{PP}$ coupling constant suggests that the two distinct phosphine ligands are positioned *trans* to each other (see examples of analogues[3, 4] and a recent example showing a similar $^2J_{PP}$ coupling constant[5]).

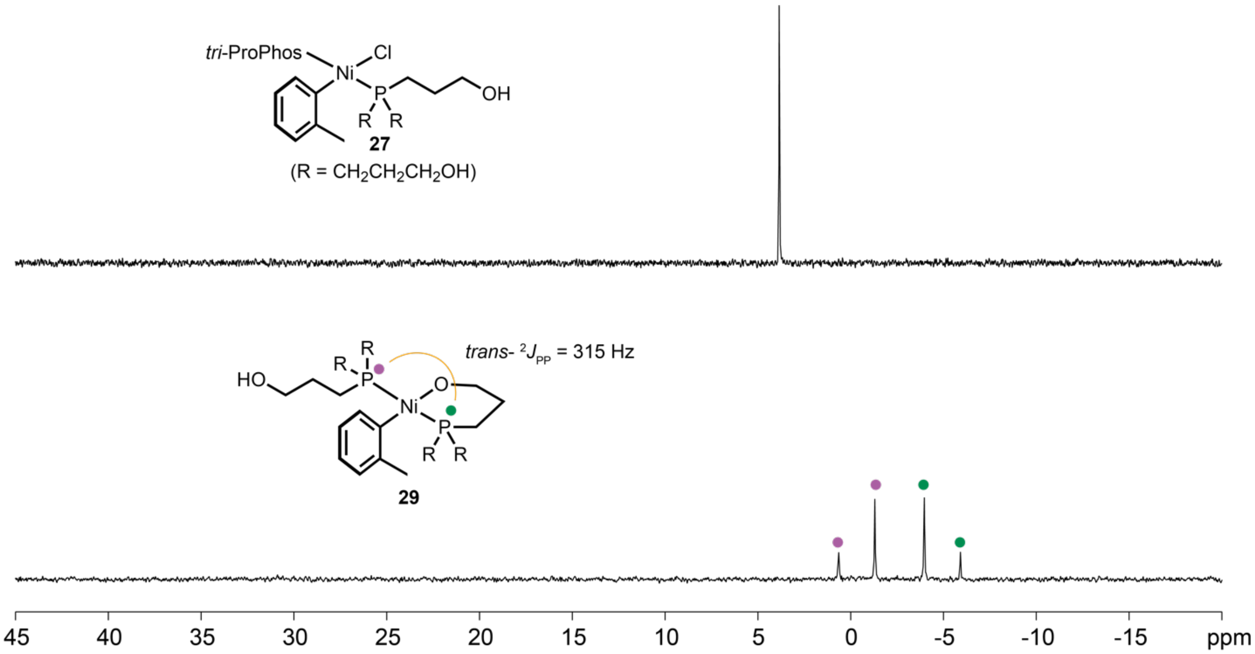

Figure S1. $^{31}P\{^1H\}$ NMR (162.04 MHz, *i*PrOD-*d$_8$*) spectra of complexes Ni(*tri*-ProPhos)$_2$Cl(*o*-Tol) **27** (top) and Ni(κ^2-$PR_2CH_2CH_2CH_2O$)(*tri*-ProPhos)(*o*-Tol) **29** (bottom). R = $CH_2CH_2CH_2OH$.

Further evidence for the formation of the Ni(*tri*-ProPhos) metallacycle comes from the ^1H NMR spectrum (Figure S2). After dehydrohalogenation, three distinct proton environments (labeled H^1, H$^{1'}$ and H^1) are expected near the oxygen atom. The ^1H NMR spectrum clearly shows these three types of protons between These appear in the range of 3.62 and 3.05 ppm with an integration ratio of 2:3:1, consistent with the proposed structure. Correlations observed in the $^1H/^1H$-COSY spectrum between H^1 and other two signals (1.47 – 1.38 ppm and 1.11 – 1.05 ppm) support the assignment of these lower-field resonances to H^2 on the metallacycle (Figure S3).

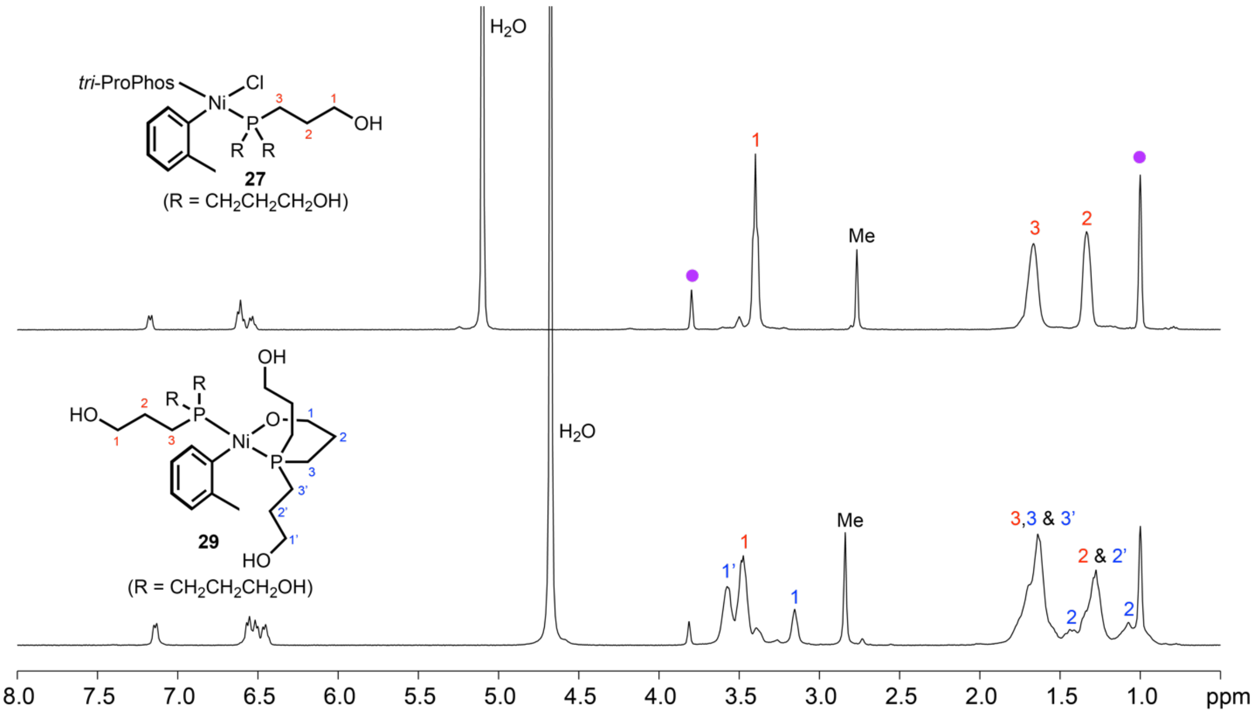

Figure S2. ^{1}H NMR (400.30 MHz, *i*PrOD-d_8) spectra of complexes Ni(*tri*-ProPhos)$_2$Cl(*o*-Tol) **27** (top) and Ni(κ^2-P̲R$_2$CH$_2$CH$_2$CH$_2$O̲)(*tri*-ProPhos)(*o*-Tol) **29** (bottom). Residual proteo-solvent (●). R = CH$_2$CH$_2$CH$_2$OH.

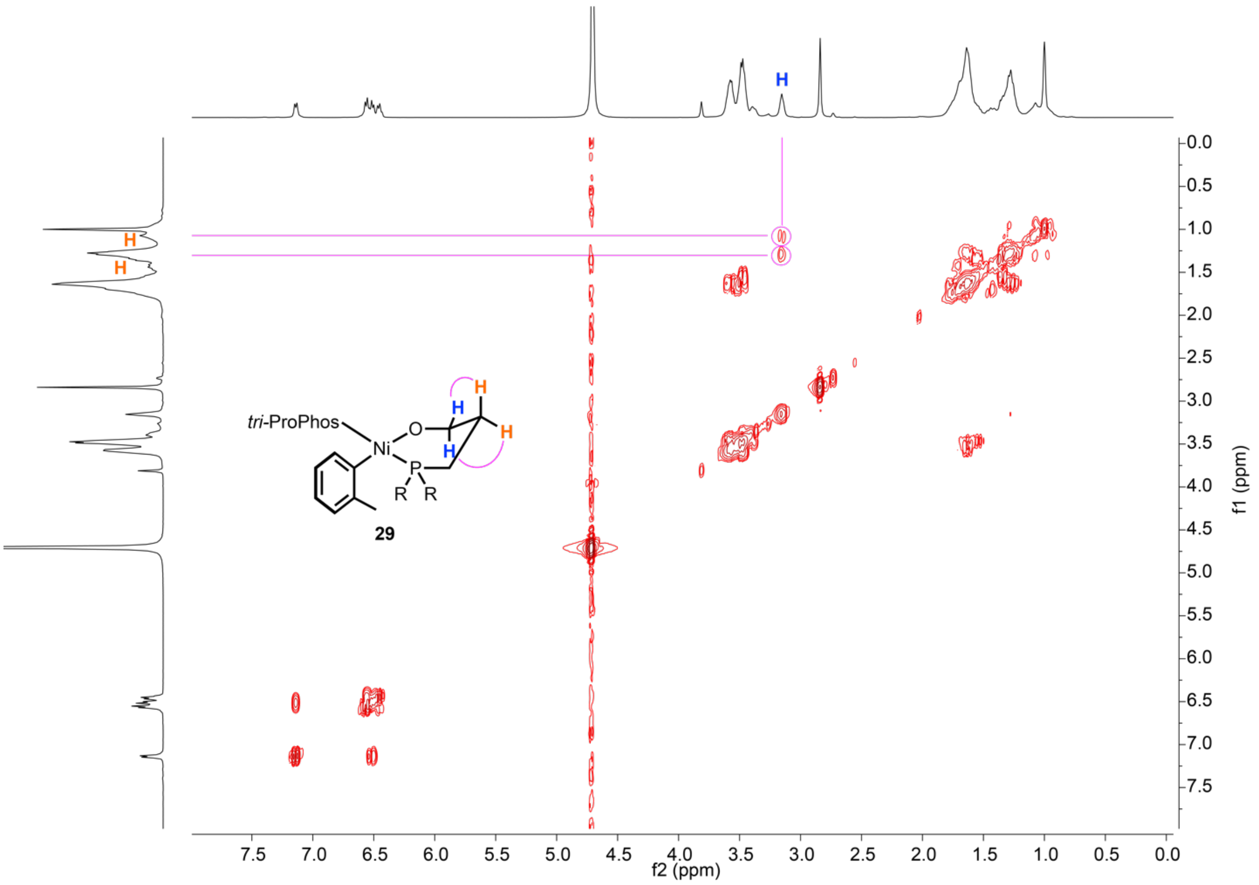

Figure S3. ^{1}H/^{1}H-COSY NMR (400.30 MHz, *i*PrOD-d_8) spectrum of Ni(κ^2-<u>P</u>$R_2CH_2CH_2CH_2$<u>O</u>)(*tri*-ProPhos)(*o*-Tol) **29**, showing the correlation between the metallacycle protons. R = $CH_2CH_2CH_2OH$.

2.6 Attempted synthesis of (*tri*-ProPhos)Ni(0) complexes

Attempted synthesis of Ni(tri-ProPhos)$_2$(cod). A vial was charged with Ni(cod)$_2$ (40.0 mg, 0.145 mmol, 1.0 equiv), *tri*-ProPhos (72.7 mg, 0.349 mmol, 2.4 equiv) and THF (5 mL). The mixture was stirred at room temperature overnight. Afterward, the solvent was removed under vacuum to afford a red paste. *i*PrOH (2 mL) was added, and the resulting suspension was filtered through a pad of Celite. The Celite was then rinsed with additional *i*PrOH (3 × 2 mL). The combined orange filtrates were concentrated under reduced pressure, layered with cyclohexane (5 mL), and stored at –35 °C overnight, which still gave a red paste. Attempts to solidify the material by triturating with pentane were unsuccessful.

The ^{31}P{^{1}H} NMR analysis of this crude showed a major singlet signal at 12.4 ppm, which was tentatively assigned to the target compound Ni(*tri*-ProPhos)$_2$(cod) (Figure S4). However, the oily nature and the presence of various byproducts pose the challenges to further purify this compound.

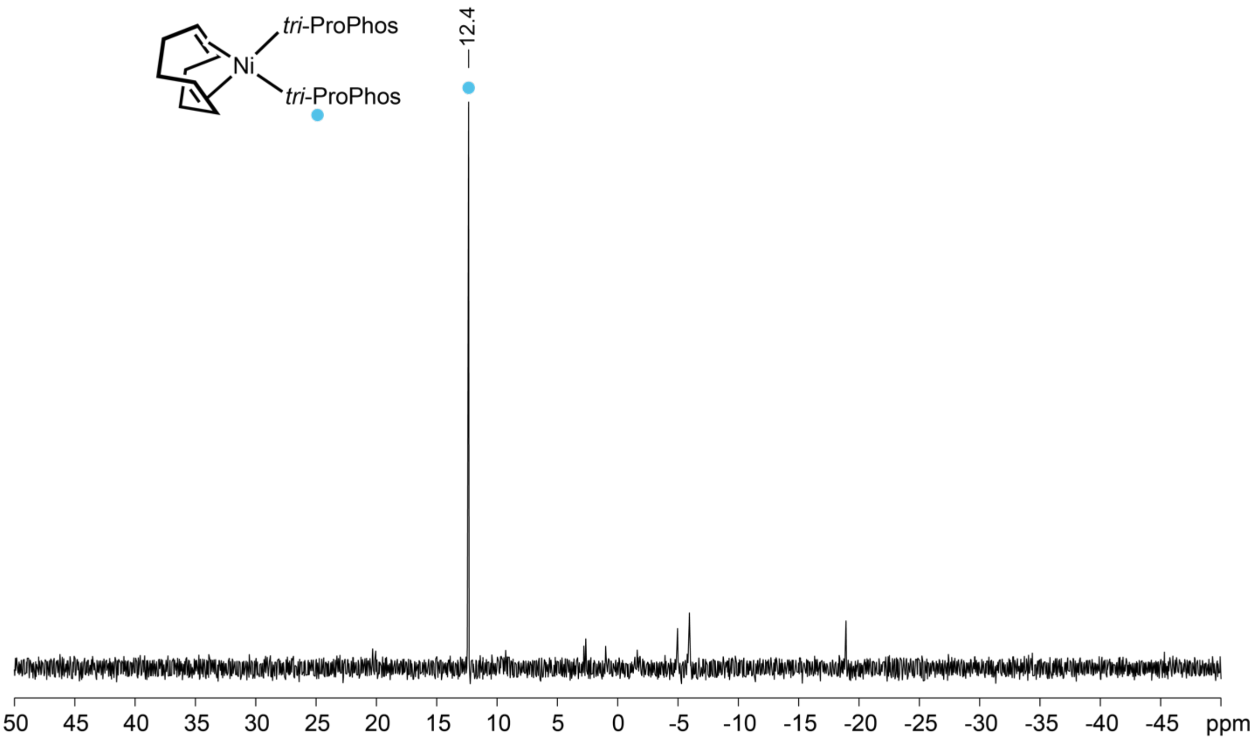

Figure S4. $^{31}P\{^{1}H\}$ NMR (202.49 MHz, *i*PrOD-d_8) spectra of the crude containing Ni(*tri*-ProPhos)$_2$(cod) and other unidentified species.

Attempted synthesis of Ni(tri-ProPhos)$_4$ ***28****.* A vial was charged with Ni(cod)$_2$ (60.0 mg, 0.218 mmol, 1.0 equiv), *tri*-ProPhos (227 mg, 1.09 mmol, 5.0 equiv) and THF (5 mL). The mixture was stirred at room temperature overnight. The solvent was removed under vacuum, yielding a red paste. *i*PrOH (2 mL) was added, and the mixture was filtered through a Celite pad, which was subsequently washed with additional *i*PrOH (3 × 2 mL). The combined orange filtrates were concentrated, layered with cyclohexane (5 mL), and stored at –35 °C overnight, but still resulted in a red paste. Similar to the attempted synthesis of Ni(*tri*-ProPhos)$_4$, further attempts to induce solidification by trituration were unsuccessful.

The $^{31}P\{^{1}H\}$ NMR spectrum of the crude mixture revealed a complex array of signals (Figure S5). The dominant singlet at 32.8 ppm was tentatively attributed to the desired complex, Ni(*tri*-ProPhos)$_4$ **28**. A minor set of peaks at 35.9 and 30.3 ppm, integrating in a 1:1 ratio, was assigned to a solvent-bound Ni(0) complex, Ni(*tri*-ProPhos)$_4$(L). Another minor pair of doublets at 10.0 and 4.6 ppm likely corresponds to Ni(*tri*-ProPhos)$_2$(L)$_3$. Similar solvent-coordinated Ni(0) phosphine species have been observed previously in (PPh$_2$Me)Ni(0) and (ProPhos)Ni(0) complexes.[2] Additionally, we cannot rule out the possibility of the pendant –OH group chelating to the Ni(0) center, which could account for the presence of additional unidentified species in the mixture. As

with earlier observations, the oily consistency and the formation of multiple byproducts continue to hinder the purification of this compound.

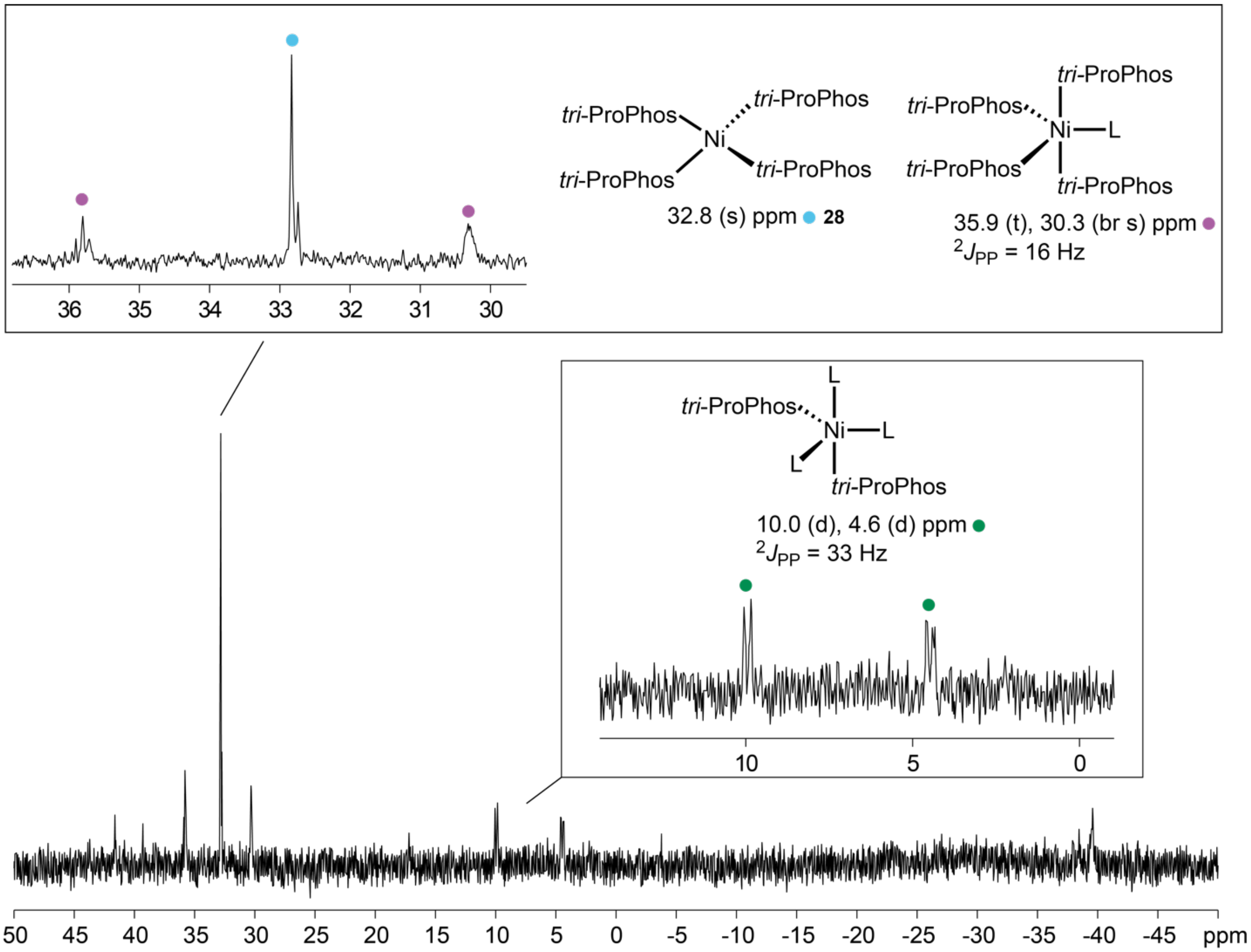

Figure S5. $^{31}P\{^1H\}$ NMR (162.04 MHz, *i*PrOD-*d*$_8$) spectrum of the crude containing Ni(*tri*-ProPhos)$_4$ **28** and other unidentified and tentatively assigned species. L = *i*PrOH or *i*PrOD or the pendant –OH group at *tri*-ProPhos.

2.7 Synthesis of K[B(3-Py)(OH)$_3$] (30)

The synthesis is slightly modified from the literature procedure.[6] To a 20 mL vial, B(3-Py)(OH)$_2$ (826 mg, 6.72 mmol, 1 equiv), KOH (377 mg, 6.72 mmol, 1 equiv), and THF (10 mL) were sequentially added. The mixture was sonicated at room temperature for 30 min. After that, the solvent volume was concentrated to ~2 mL under vacuum. The suspension was filtered by a frit, and the precipitate was washed with hexane (5 x 5 mL). The precipitate was dried under vacuum to yield the product as a white solid (986 mg, 5.51 mmol, 82%).

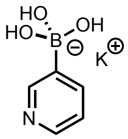

30

^{1}H NMR (400.30 MHz, D_2O) δ: 8.62 – 8.52 (m, 1H), 8.32 (dd, *J* = 5.1, 1.8 Hz, 1H), 8.03 (dt, *J* = 7.6, 1.9 Hz, 1H), 7.38 (ddd, *J* = 7.6, 5.1, 1.0 Hz, 1H).

^{1}H NMR (500.20 MHz, *i*PrOD-d_8) δ: 8.62 – 8.50 (m, 1H), 8.10 – 8.05 (m, 1H), 7.84 (d, *J* = 7.4 Hz, 1H), 7.04 – 6.94 (m, 1H).

^{11}B NMR (128.43 MHz, D_2O) δ: 2.7 (br s).

^{11}B NMR (160.48 MHz, *i*PrOD-d_8) δ: 5.1 (br s).

^{13}C{^{1}H} NMR (100.67 MHz, D_2O) δ: 149.9 (s), 144.5 (s), 141.7 (s), 123.7 (s). One quaternary ^{13}C signal at 3-Py group is not observed.

^{13}C{^{1}H} NMR (125.79 MHz, *i*PrOD-d_8) δ: 153.8 (s), 145.0 (s), 142.2 (s), 122.8 (s). One quaternary ^{13}C signal at 3-Py group is not observed.

HRMS (ESI-TOF, H_2O) m/z: $[M – H_2O – K]^–$ calculated for $C_5H_5B_1N_1O_2$ 122.0420, found 122.0425.

3. Screenings of Phosphine Ligands and Catalysts in Ni-SMC

3.1 General procedure

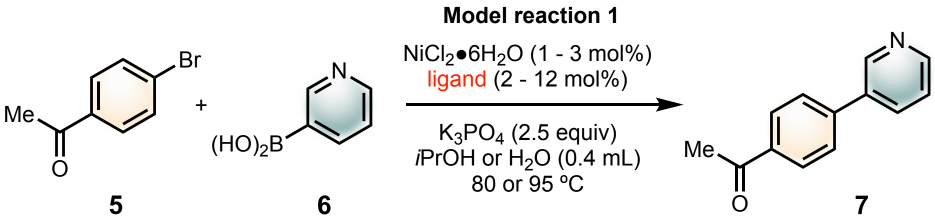

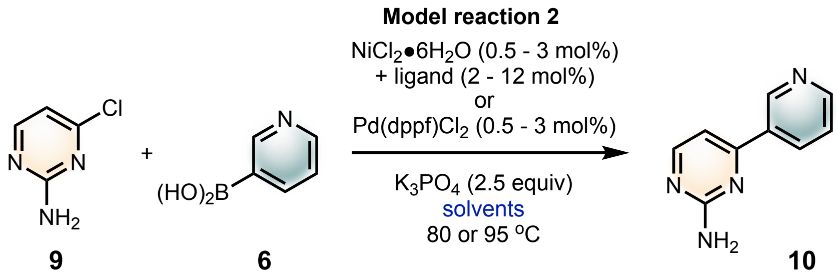

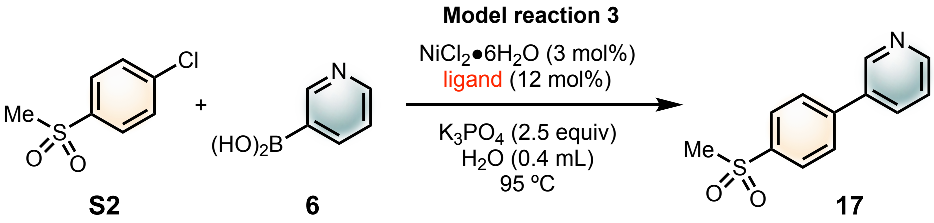

Model reaction(s) in iPrOH and 2-MeTHF.

In a nitrogen-filled glove box, $NiCl_2•6H_2O$ (7.5 µmol, 1.7 mg) combined with *either* monodentate/hemi-labile phosphines (30 µmol, 4.0 equiv with respect to Ni) *or* bidentate phosphines (15 µmol, 2.0 equiv with respect to Ni) or $Pd(dppf)Cl_2$ (7.5 µmol, 5.5 mg) was in a 2 mL glass vial containing isopropanol (*i*PrOH, 1.5 mL) or 2-methyltetrahydrofuran (2-MeTHF, 1.5 mL). The vial was then removed from the glove box, placed in a shaker, and heated to 70 °C with agitation at 800 rpm for 30 min. The resulting stock solution or suspension was brought back into the glove box for further use. Separately, an aryl halide (0.20 mmol, 1.0 equiv) and pyridin-3-ylboronic acid **6** (0.30 mmol, 1.5 equiv) were weighed into a 2 mL glass vial, along with solid K_3PO_4 (0.50 mmol, 2.5 equiv). The precatalyst solution/suspension (0.4 mL) was added to the vial. For suspensions, an aliquot was taken while vigorously shaking the vial to ensure even distribution of the catalyst. The vial was then sealed, removed from the glove box, and placed in a shaker, heated to 80 °C with agitation at 800 rpm for 6 or 16 h. If applicable, cosolvent water was added before the heating. After cooling to room temperature, *n*-decane was added as an internal standard. The samples were quenched with water and extracted with ethyl acetate. The

organic layer was combined, and an aliquot was analyzed by GC-FID. Calibration curves for three isolated biaryl products **7**, **10** and **17** (see Section 4) were created to determine their R_f values for quantitative analysis.

Model reaction(s) in H_2O.

Followed the procedure in *i*PrOH and 2-MeTHF. The catalyst preparation was conducted outside the glovebox. Molecular water was used. The resulting stock solution or suspension was purged with nitrogen gas before adding to the substrates under a nitrogen atmosphere. For catalytic reactions using >1 mol% catalyst loading, the catalyst and/or ligand was directly weighed/measured without preparing a stock solution. The catalyst reactions were performed at 95 °C.

Choice of ligands for screenings

Prior mechanistic studies suggest that Ni-catalyzed Suzuki–Miyaura couplings of aryl substrates typically proceed through a closed-shell Ni(0)/Ni(II) cycle. This is consistent with high-throughput screening results,[7, 8] which highlight phosphine ligands (good σ-donors) as privileged ligands for the cross-coupling of heteroaromatic substrates. Interestingly, some NHC ligands[9], which are also good σ-donors, or even "naked nickel" systems[10] have enabled these couplings. A selection of the most relevant and high-performing ligands for this transformation is shown in Scheme S1. Since many of the top ligands reported are phosphine-based, we chose to compare ProPhos and *tri*-ProPhos against a set of widely used, commercially available P-ligands, including monophosphines, Buchwald-type ligands, and bisphosphines, in model reaction 1 (Scheme S2). Note: To rule out the possibility that impurities in commercially available *tri*-ProPhos is responsible for the observed catalytic activity, the isolated complex Ni(*tri*-ProPhos)$_2$Cl(*o*-Tol) **27** was used as a precatalyst in the model reaction 1 (Table S4). This reaction proceeded with high efficiency, delivering the desired product **7** in approximately 90% yield even under air (*vide infra*). Furthermore, *tri*-ProPhos ligands obtained from various suppliers (e.g., Sigma, Strem Chemicals) were also tested in the same model reaction, all affording product **7** in yields exceeding 90%. These results strongly suggest that these impurities present in commercial *tri*-ProPhos have a negligible impact on the catalytic performance.

Scheme S1. Selected Ni Catalysts/Precursors and Ligands Used in Ni-SMC of Heteroaromatics.[a]

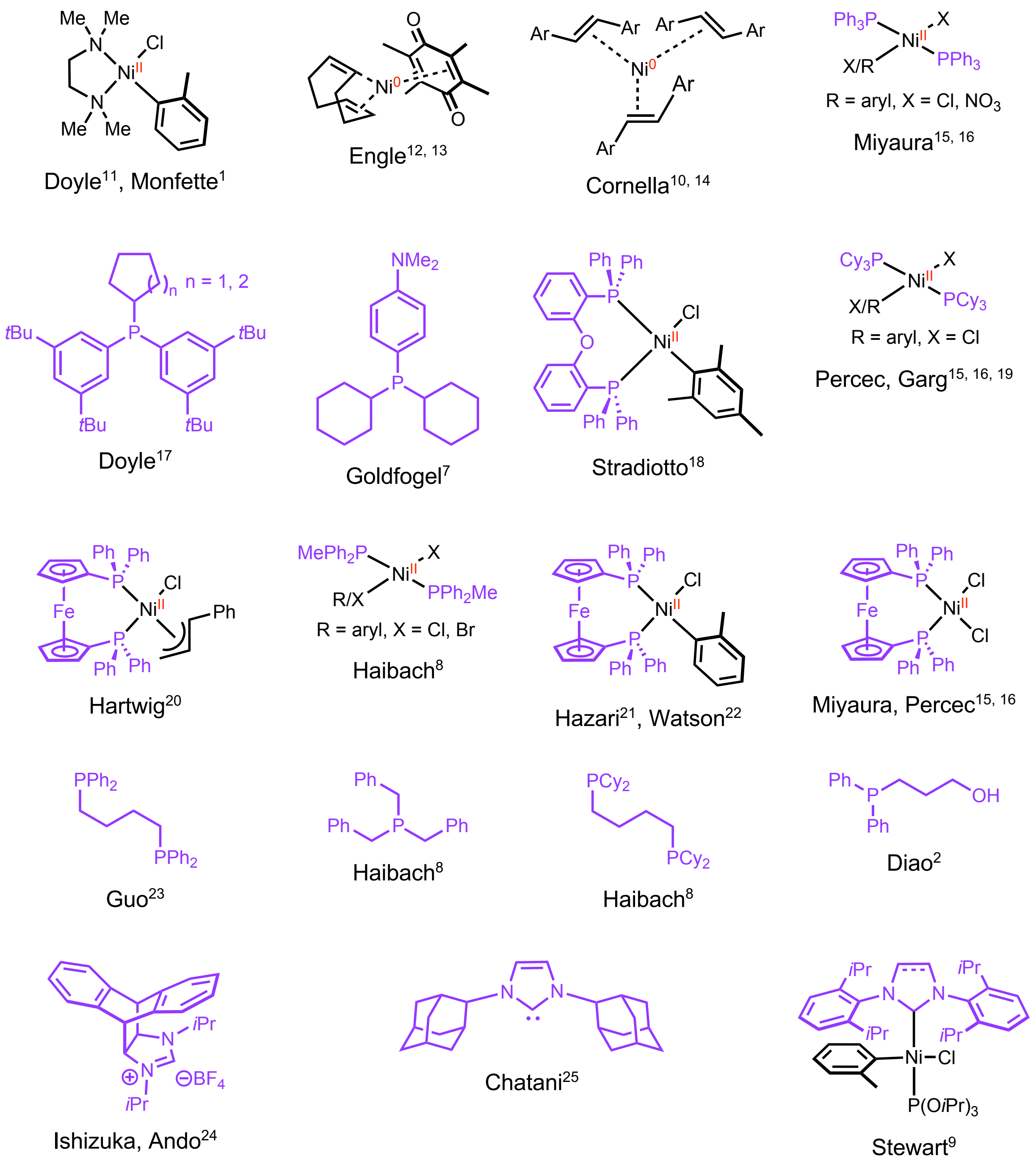

[a]Ligands highlighted in purple.

Scheme S2. Structures of Phosphine Ligands in the Screening Experiments.

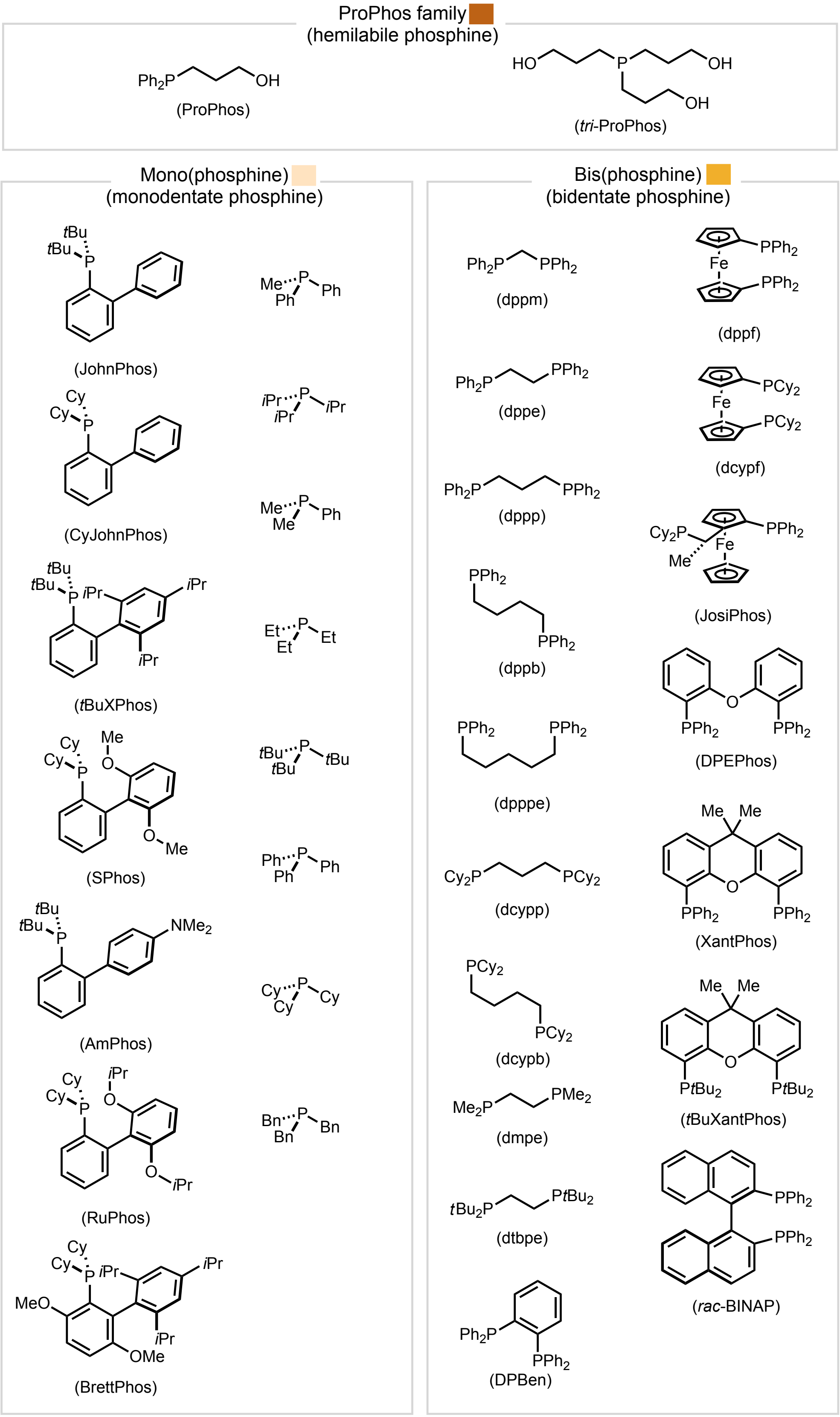

Table S1. Evaluation of Phosphine Ligands in Model Reaction 1.

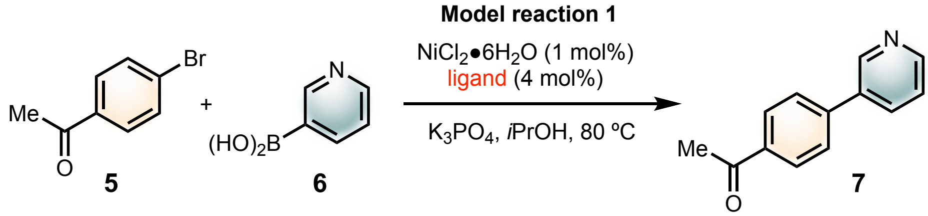

Entry	Ligands	Type	%Yield of 7	CAS	Source
1	*tri*-ProPhos	ProPhos family	95	4706-17-6	Strem Chemicals
2	ProPhos	ProPhos family	95	2360-09-0	Synthesized [a]
3	JohnPhos	monophosphine	14	224311-51-7	Strem Chemicals
4	CyJohnPhos	monophosphine	8	247940-06-3	Strem Chemicals
5	*t*BuXPhos	monophosphine	6	564483-19-8	Sigma- Aldrich
6	SPhos	monophosphine	7	657408-07-6	Ambeed
7	AmPhos	monophosphine	2	932710-63-9	Ambeed
8	RuPhos	monophosphine	4	787618-22-8	Ambeed
9	BrettPhos	monophosphine	7	1070663-78-3	Ambeed
10	PPh_2Me	monophosphine	25	1486-28-8	Ambeed
11	$P\textit{i}Pr_3$	monophosphine	20	6476-36-4	Thermo Fisher
12	$PPhMe_2$	monophosphine	3	672-66-2	Sigma Aldrich
13	PEt_3	monophosphine	10	554-70-1	Sigma Aldrich
14	$P\textit{t}Bu_3$	monophosphine	3	13716-12-6	Sigma Aldrich
15	PPh_3	monophosphine	2	603-35-0	Sigma Aldrich
16	PCy_3	monophosphine	4	2622-14-2	Sigma Aldrich
17	PBn_3	monophosphine	22	7650-89-7	Alfa
18	Dppm	bisphosphine	15	2071-20-7	Sigma Aldrich
19	Dppe	bisphosphine	0	1663-45-2	Sigma Aldrich
20	Dppp	bisphosphine	0	6737-42-4	Sigma Aldrich
21	Dppb	bisphosphine	10	7688-25-7	Sigma Aldrich
22	Dpppe	bisphosphine	2	27721-02-4	Sigma Aldrich

23	Dcypp	bisphosphine	0	103099-52-1	Ambeed
24	Dcypb	bisphosphine	2	65038-36-0	Ambeed
25	Dmpe	bisphosphine	55	23936-60-9	Strem Chemicals
26	Dtbpe	bisphosphine	1	107783-62-0	Ambeed
27	Dppf	bisphosphine	12	12150-46-8	Sigma Aldrich
28	Dcypf	bisphosphine	2	146960-90-9	Ambeed
29	JosiPhos	bisphosphine	0	155806-35-2	Sigma Aldrich
30	DPEPhos	bisphosphine	7	166330-10-5	Sigma Aldrich
31	XantPhos	bisphosphine	0	161265-03-8	Sigma Aldrich
32	*t*BuXantPhos	bisphosphine	2	856405-77-1	Strem Chemicals
33	*rac*-BINAP	bisphosphine	2	98327-87-8	Sigma Aldrich
34	DPBen	bisphosphine	0	13991-08-7	Sigma Aldrich
35	N/A	control	0	-	-

[a]Also commercially available.

Table S2. Solubility and Appearance of $NiCl_2 \cdot 6H_2O$ and Ligands in *i*PrOH.

Entry	Ligands	Appearance	Entry	Ligands	Appearance
1	*tri*-ProPhos	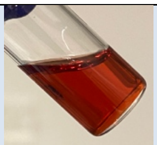 homogeneous	18	Dppm	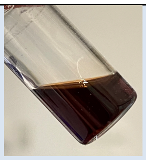 homogeneous
2	ProPhos	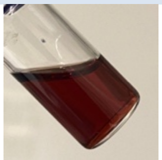 homogeneous	19	Dppe	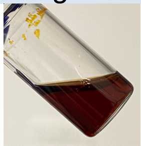 homogeneous
3	JohnPhos	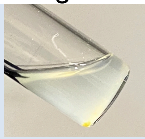 heterogeneous	20	Dppp	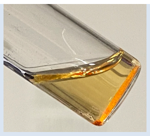 heterogeneous
4	CyJohnPhos	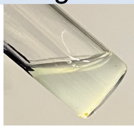 heterogeneous	21	Dppb	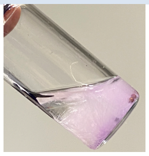 heterogeneous
5	*t*BuXPhos	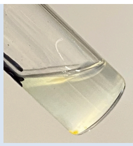 heterogeneous	22	Dpppe	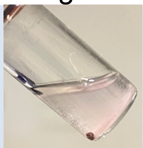 heterogeneous
6	SPhos	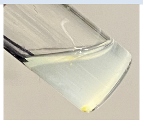 heterogeneous	23	Dcypp	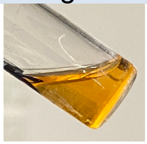 homogeneous
7	AmPhos /APhos	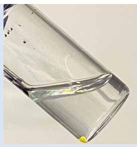 heterogeneous	24	Dcypb	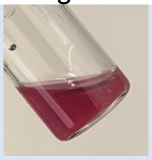 homogeneous*
8	RuPhos	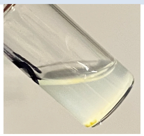 heterogeneous	25	Dppm	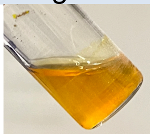 homogeneous*
9	BrettPhos	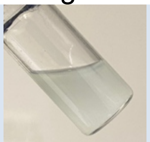 heterogeneous	26	Dtbpe	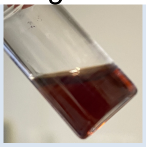 Homogeneous*

10	PPh_2Me	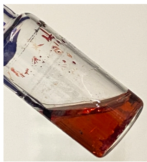 homogeneous*	27	Dppf	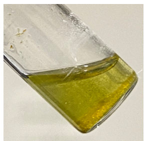 heterogeneous
11	P*i*Pr$_3$	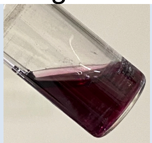 homogeneous	28	Dcypf	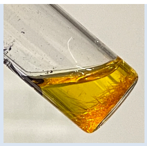 heterogeneous
12	$PPhMe_2$	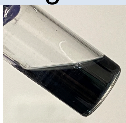 homogeneous	29	JosiPhos	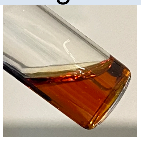 homogeneous
13	PEt_3	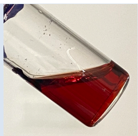 homogeneous	30	DPEPhos	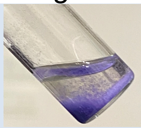 heterogeneous
14	P*t*Bu$_3$	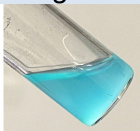 homogeneous	31	XantPhos	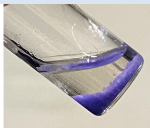 heterogeneous
15	PPh_3	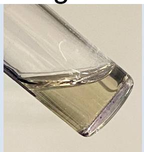 heterogeneous	32	*t*BuXantPhos	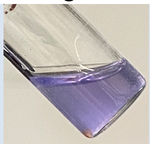 heterogeneous
16	PCy_3	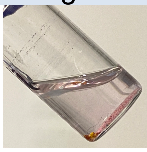 heterogeneous	33	*rac*-BINAP	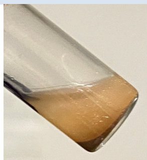 heterogeneous
17	PBn_3	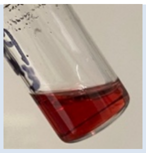 homogeneous	34	DPBen	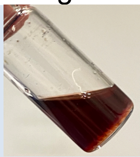 heterogeneous

* homogeneous at 70 ^{o}C.

3.2 Screening results under water condition

For the ligand screening in water, we selected only three commercially available ligands known to be water-soluble. Most other ligands shown in Scheme S2 were excluded because they were not soluble in water when we attempted to prepare the precatalyst solutions. This may explain why there are only few reports using water as the sole solvent for Ni-SMC.[26-29] Among them, only one example demonstrates successful coupling of heteroaryl substrates using Ni nanoparticles rather than molecular Ni complexes.[27]

Scheme S3. Screening Experiments of Water-Soluble Phosphine Ligands.

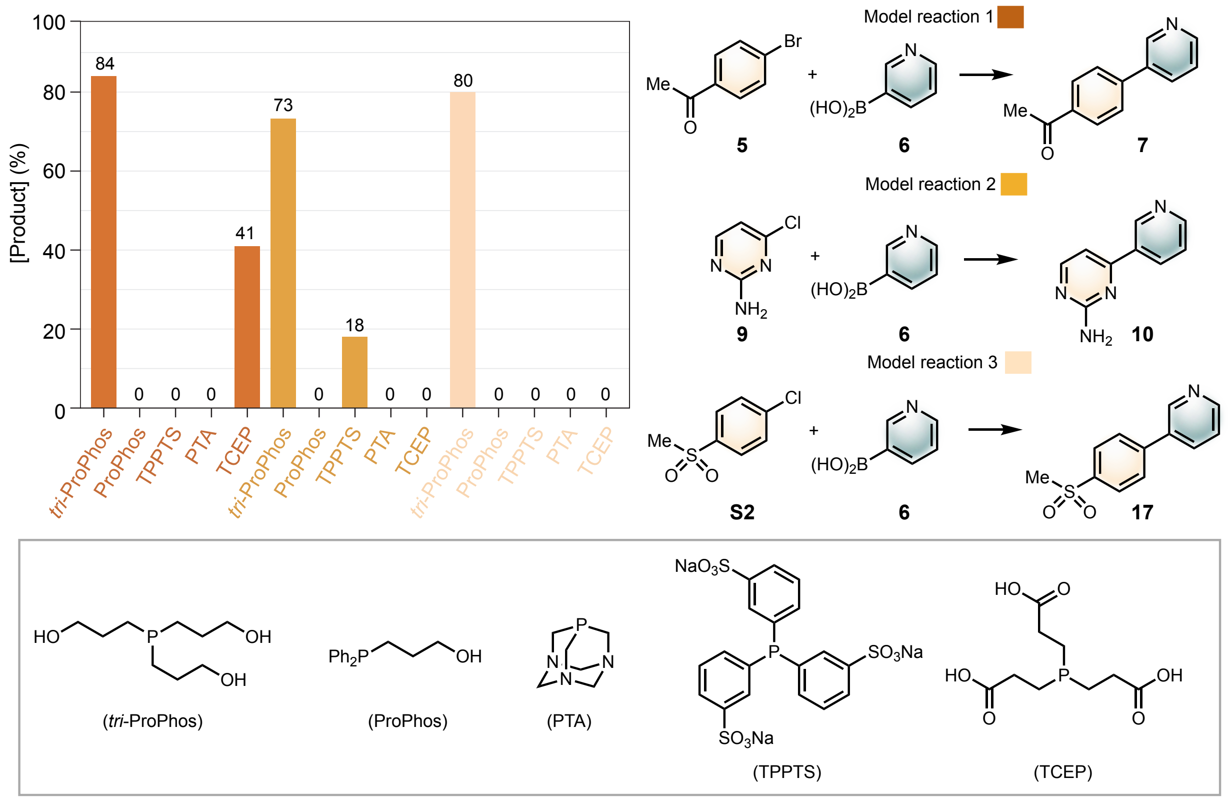

Condition: $NiCl_2{\bullet}6H_2O$ (3 mol%), ligand (12 mol%), K_3PO_4, H_2O, 95 °C, 16 h.

4. Substrate Scope using (*tri*-ProPhos)Ni Catalysts

4.1 General procedure for Ni-SMC

Method A – $NiCl_2$•$6H_2O$ in iPrOH

In most case, a vial was charged with $NiCl_2$•$6H_2O$ (1.4 mg, 6.0 μmol), *i*PrOH (2 mL) and *tri*-ProPhos (24 μmol). The vial was sealed, removed from the glove box, and placed in a shaker. It was then heated to 70 °C with agitation at 800 rpm for 30 min, resulting in a red stock solution. This solution was returned to the glove box for subsequent use. Appearance of the Ni precatalyst solution is shown in Table S2.

A separate vial was prepared with the aryl boronic acid or ester or boronate (0.30 mmol, 1.5 equiv), aryl halide (0.20 mmol if solid, 1.0 equiv), and K_3PO_4 (0.50 mmol, 2.5 equiv). If the aryl halide was liquid, it was added afterward (0.20 mmol, 1.0 equiv). The Ni phosphine stock solution and deoxygenated *i*PrOH (0.3 – 0.4 mL) were then added. The vial was sealed and removed from the glove box, then placed in a shaker. The reaction mixture was heated to 80 °C with agitation at 800 rpm for 20 h and then cooled to room temperature. The mixture was diluted with 10 mL of ethyl acetate (EtOAc) and washed with water or NH_4Cl solution (2 x 5 mL). The combined aqueous layers were further extracted with 5 mL of EtOAc. An aliquot of the combined organic layers was analyzed by gas chromatography (GC) or ultra-performance liquid chromatography-mass spectrometry (UPLC-MS) to monitor the reaction progress. The combined organic layers were then concentrated in vacuo, and the crude product was purified by flash chromatography. Multiple Ni catalyst loadings (0.03 – 5 mol%) with *tri*-ProPhos were used for synthesizing these compounds.

Method B – $NiCl_2$•$6H_2O$ in H_2O

In most case, a vial was charged with $NiCl_2$•$6H_2O$ (1.4 – 2.3 mg, 6.0 – 10 μmol, 3 – 5 mol%) and *tri*-ProPhos (4 equiv with respect to Ni). Molecular biology grade H_2O (0.3 – 0.4 mL) was added to prepare the precatalyst solution. The vial was sealed, purged by nitrogen gas for 5 min, and placed in a shaker. It was then shaken with agitation at room temperature at 800 rpm for 10 min, resulting in a dark orange solution. Substrates were prepared in a glovebox and followed by a similar procedure to Method A, and neopentyl glycol (NPG, 1.5 equiv) was added (if applicable). After the vial was sealed and removed from the glove box, the precatalyst solution was inject into the vial, then placed in a shaker. The reaction mixture was heated to 95 °C with agitation at 800 rpm for 20 h and then cooled to room temperature. Work-up steps were largely consistent with those described in Method A. 1, 3, 5-Tri-methoxylbenzene was used as an internal standard to determine the product yield by quantitative ^1H NMR (also for some examples using Method A).

4.2 Synthesis and characterization of Ni-SMC products

1-[4-(3-Pyridinyl)phenyl]ethenone **(7)**

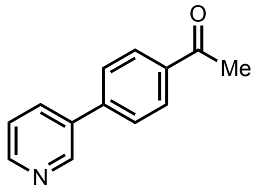

7

Method A: With 0.03 mol% Ni loading, 4-bromophenylethanone (39.8 mg, 0.200 mmol, 1.0 equiv) and pyridin-3-ylboronic acid (36.9 mg, 0.300 mmol, 1.5 equiv) were used, purified by flash chromatography (EtOAc:hexane), to give a white solid (35.1 mg, 0.178 mmol, 89%).

Method B: With 3 mol% Ni loading, 4-bromophenylethanone (39.8 mg, 0.200 mmol, 1.0 equiv) and 3-pyridineboronic acid pinacol ester (61.5 mg, 0.300 mmol, 1.5 equiv) were used, the isolated yield was determined to be 91% (36.0 mg, 0.183 mmol).

The NMR data of the isolated compound are consistent with the literature reports.[2, 30] A larger-scale synthesis (1.00 mmol) was performed using 0.1 mol% Ni loading, which gave a comparable yield. The isolated compound was used to generate a calibration curve for quantitative GC-FID analysis (for Section 3).

^{1}H NMR (400.30 MHz, $CDCl_3$) δ: 8.89 (s, 1H), 8.65 (d, *J* = 4.8 Hz, 1H), 8.07 (dd, *J* = 8.3, 1.7 Hz, 2H), 7.91 (dt, *J* = 8.0, 2.0 Hz, 1H), 7.68 (dd, *J* = 8.4, 1.8 Hz, 2H), 7.40 (dd, *J* = 8.1, 4.9 Hz, 1H), 2.65 (s, 3H, Me).

^{13}C{^{1}H} NMR (100.67 MHz, $CDCl_3$) δ: 197.7 (s, C=O), 149.5 (s), 148.5 (s), 142.5 (s), 136.7 (s), 135.6 (s), 134.6 (s), 129.3 (s), 127.4 (s), 123.8 (s), 26.8 (s, Me).

LCMS (ESI-TOF, CH_3OH) m/z: $[M + H]^+$ calculated for $C_{13}H_{12}NO$ 198.09, found 198.09.

4-(3-Pyridyl)-2-pyrimidinamine **10**

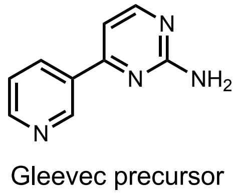

Gleevec precursor

10

Method A: With 0.5 mol% Ni loading, 4-chloropyrimidin-2-amine (25.9 mg, 0.200 mmol, 1.0 equiv) and pyridin-3-ylboronic acid (36.9 mg, 0.300 mmol, 1.5 equiv) were used, purified by flash chromatography (MeOH:DCM), to give a white solid (27.3 mg, 0.159 mmol, 79%). A gram-scale synthesis employing ProPhos was carried out, affording this compound in a comparable yield. The product was subsequently used to generate a calibration curve for quantitative GC-FID analysis (Section 3).
The NMR data of the isolated compound are consistent with the literature report.[31]

^{1}H NMR (400.30 MHz, DMSO-*d*$_6$) δ: 9.23 (dd, *J* = 2.3, 0.9 Hz, 1H), 8.68 (dd, *J* = 4.8, 1.7 Hz, 1H), 8.39 (ddd, *J* = 8.0, 2.3, 1.7 Hz, 1H), 8.36 (s, 1H), 7.53 (ddd, *J* = 8.0, 4.8, 0.9 Hz, 1H), 7.20 (d, *J* = 5.1 Hz, 1H), 6.77 (s, 2H).

^{13}C{^{1}H} NMR (100.67 MHz, DMSO-*d*$_6$) δ: 163.8 (s), 161.6 (s), 159.4 (s), 151.2 (s), 148.0 (s), 134.2 (s), 132.5 (s), 123.8 (s), 106.0 (s).

HRMS (ESI-TOF, CH_3OH) m/z: $[M + H]^+$ calculated for $C_9H_9N_4$ 173.0822, found 173.0827.

3-(4-Fluorophenyl)pyridine ***(11)***

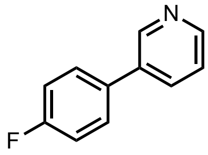

11

Method A: With 0.05 mol% Ni loading, 3-chloropyridine (19.0 µL, 0.200 mmol, 1.0 equiv) and (4-fluorophenyl)boronic acid (42.0 mg, 0.300 mmol, 1.5 equiv) were used, purified by flash chromatography (EtOAc:hexane), to give a brown liquid (30.9 mg, 0.178 mmol, 89%).
Method B: With 3 mol% Ni loading, 3-chloropyridine (19.0 µL, 0.200 mmol, 1.0 equiv) and (4-fluorophenyl)boronic acid (42.0 mg, 0.300 mmol, 1.5 equiv) were used, the isolated yield was determined to be 89% (30.8 mg, 0.178 mmol).

The NMR data of the isolated compound are consistent with the literature report.[32]

^{1}H NMR (400.30 MHz, $CDCl_3$) δ: 8.80 (d, *J* = 2.4 Hz, 1H), 8.58 (dd, *J* = 4.9, 1.6 Hz, 1H), 7.81 (ddt, *J* = 8.0, 2.4, 1.2 Hz, 1H), 7.58 – 7.49 (m, 2H), 7.34 (ddd, *J* = 7.9, 4.8, 1.0 Hz, 1H), 7.20 – 7.11 (m, 2H).

$^{19}F\{^1H\}$ NMR (470.61 MHz, $CDCl_3$) δ: -114.18 (s).

$^{13}C\{^1H\}$ NMR (100.67 MHz, $CDCl_3$) δ 163.0 (d, *J* = 248 Hz), 148.6 (s), 148.3 (s), 135.8 (s), 134.3 (s), 134.1 (d, *J* = 3 Hz), 128.9 (d, *J* = 8 Hz), 123.7, 116.2 (d, *J* = 22 Hz).

LCMS (ESI-TOF, CH_3CN) m/z: $[M + H]^+$ calculated $C_{11}H_9FN$ 174.07, found 174.07.

2,4-Dimethoxy-5-(pyridin-2-yl)pyrimidine ***(12)***

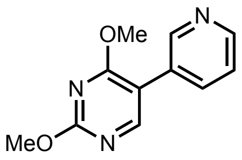

12

Method A: With 0.05 mol% Ni loading, 3-chloropyridine (19.0 µL, 0.200 mmol, 1.0 equiv) and (2,4-dimethoxypyrimidin-5-yl)boronic acid (55.2 mg, 0.300 mmol, 1.5 equiv) were used, purified by flash chromatography (EtOAc:hexane), to give a white solid (34.7 mg, 0.160 mmol, 80%).
Method B: With 3 mol% Ni loading, 3-chloropyridine (19.0 µL, 0.200 mmol, 1.0 equiv) and (2,4-dimethoxypyrimidin-5-yl)boronic acid (55.2 mg, 0.300 mmol, 1.5 equiv) were used, the isolated yield was determined to be 66% (28.8 mg, 0.133 mmol).
The lower yield observed under aqueous conditions is likely due to rapid protodeboronation of the boronic acid, as evidenced by the substantial formation of the corresponding protodeboronation product detected by GC-MS.
^1H NMR (400.30 MHz, $CDCl_3$) δ: 8.74 (d, *J* = 2.2 Hz, 1H), 8.59 (dd, *J* = 4.9, 1.6 Hz, 1H), 8.29 (s, 1H), 7.87 – 7.79 (m, 1H), 7.36 (ddd, *J* = 7.9, 4.9, 0.9 Hz, 1H), 4.05 (s, 3H, OMe), 4.04 (s, 3H, OMe).

$^{13}C\{^1H\}$ NMR (100.67 MHz, $CDCl_3$) δ: 168.4 (s), 165.3 (s), 157.8 (s), 149.6 (s), 148.9 (s), 136.3 (s), 129.5 (s), 123.4 (s), 113.1 (s), 55.2 (s, OMe), 54.4 (s, OMe).

HRMS (ESI-TOF, CH_3CN) m/z: $[M + Na]^+$ calculated for $C_{11}H_{11}N_3O_2Na$ 240.0743, found 240.0745.

6-Phenylquinoline ***(13)***

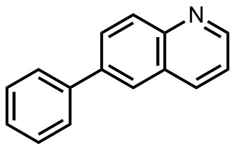

13

Method A: With 1 mol% Ni loading, 6-chloroisoquinoline (32.7 mg, 0.200 mmol, 1.0 equiv) and potassium phenyltrifluoroborate (55.2 mg, 0.300 mmol, 1.5 equiv) were used, purified by flash chromatography (EtOAc:hexane), to give a white solid (39.8 mg, 0.194 mmol, 97%). When ProPhos was used instead of *tri*-ProPhos, only a trace amount of product was determined by GCMS and TLC.

Method B: With 3 mol% Ni loading, 6-chloroisoquinoline (32.7 mg, 0.200 mmol, 1.0 equiv) and potassium phenyltrifluoroborate (55.2 mg, 0.300 mmol, 1.5 equiv) were used, but the reaction gave a trace amount of product as determined by GCMS and TLC. One possible explanation is that the boronate underwent rapid protodeboronation in water.

The NMR data of the isolated product are consistent with the literature report.[2]

^{1}H NMR (400.30 MHz, $CDCl_3$) δ: 8.91 (dd, *J* = 4.3, 1.7 Hz, 1H), 8.22 – 8.15 (m, 2H), 8.00 – 7.96 (m, 2H), 7.73 – 7.68 (m, 2H), 7.49 (dd, *J* = 8.4, 6.8 Hz, 2H), 7.43 – 7.37 (m, 2H).

^{13}C{^{1}H} NMR (100.67 MHz, $CDCl_3$) δ: 150.4 (s), 147.7 (s), 140.4 (s), 139.4 (s), 136.3 (s), 130.0 (s), 129.3 (s), 129.1 (s), 128.5 (s), 127.8 (s), 127.5 (s), 125.6 (s), 121.5 (s).

3'-Amino-2'-methyl-[1,1'-biphenyl]-4-yl)ethenone ***(14)***

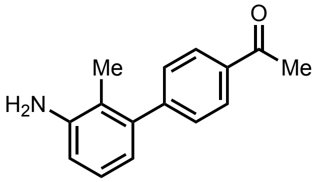

14

Method A: With 3 mol% Ni loading, 4-bromophenylethanone (39.8 mg, 0.200 mmol, 1.0 equiv) and (3-amino-2-methylphenyl)boronic acid HCl salt (40.5 mg, 0.300 mmol, 1.5 equiv) and K_3PO_4 (149 mg, 0.700 mmol, 3.5 equiv) were used, the isolated yield was determined to be 50% (22.7 mg, 0.101 mmol)

Method B: With 3 mol% Ni loading, 4-bromophenylethanone (39.8 mg, 0.200 mmol, 1.0 equiv) and (3-amino-2-methylphenyl)boronic acid HCl salt (40.5 mg, 0.300 mmol, 1.5 equiv), neopentyl

glycol (NPG, 31.2 mg, 0.300 mmol, 1.5 equiv) and K_3PO_4 (149 mg, 0.700 mmol, 3.5 equiv) were used, the NMR yield was determined to be 79%. The crude was purified by flash chromatography (EtOAc:hexane), to give a white solid (32.8 mg, 0.146 mmol, 73%).

^{1}H NMR (400.30 MHz, $CDCl_3$) δ: 8.00 (d, *J* = 8.0 Hz, 2H), 7.41 (d, *J* = 8.0 Hz, 2H), 7.09 (t, *J* = 7.8 Hz, 1H), 6.74 (d, *J* = 7.9 Hz, 1H), 6.69 (d, *J* = 7.5 Hz, 1H), 3.76 (br s, 2H, NH_2), 2.65 (s, 3H, Me), 2.06 (s, 3H, Me).

^{13}C{^{1}H} NMR (100.67 MHz, $CDCl_3$) δ: 198.0 (s, C=O), 147.6 (s), 145.2 (s), 142.0 (s), 135.6 (s), 129.8 (s), 128.2 (s), 126.5 (s), 120.3 (s), 119.7 (s), 114.8 (s), 26.8 (s, Me), 14.5 (s, Me).

HRMS (ESI-TOF, CH_3OH) m/z: $[M + H]^+$ calculated for $C_{15}H_{16}NO$ 226.1226, found 226.1229.

1-(4-(1-Methyl-1H-pyrazol-4-yl)phenyl)ethan-1-one ***(15)***

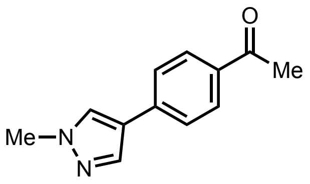

15

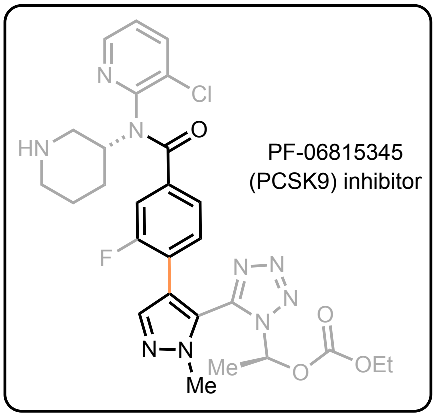

Method A: With 0.5 mol% Ni loading, 4-bromophenylethanone (39.8 mg, 0.200 mmol, 1.0 equiv) and (1-methyl-1*H*-pyrazol-4-yl)boronic acid (37.8 mg, 0.300 mmol, 1.5 equiv) were used, purified by flash chromatography (EtOAc:hexane), to give a white solid (35.5 mg, 0.177 mmol, 89%).
Method B: With 3 mol% Ni loading, 4-bromophenylethanone (39.8 mg, 0.200 mmol, 1.0 equiv) and (1-methyl-1*H*-pyrazol-4-yl)boronic acid (37.8 mg, 0.300 mmol, 1.5 equiv) were used, but the reaction gave a trace amount of product as determined by GCMS and TLC. One possible explanation is that the boronic acid underwent rapid protodeboronation in water, as a significant amount of the corresponding protodeboronation product was observed by GC-MS.
The NMR data of the isolated product are consistent with the literature report.[33]

^{1}H NMR (400.30 MHz, CDCl$_3$) δ: 7.95 (d, *J* = 8.5 Hz, 2H), 7.83 (s, 1H), 7.70 (s, 1H), 7.54 (d, *J* = 8.5 Hz, 2H), 3.96 (s, 3H, Me), 2.60 (s, 3H, Me).

^{13}C{^{1}H} NMR (100.67 MHz, CDCl$_3$) δ: 197.6 (s), 137.6 (s), 137.3 (s), 135.1 (s), 129.3 (s), 127.9 (s), 125.3 (s), 122.4 (s), 39.4 (s, Me), 26.7 (s, Me).

LCMS (ESI-TOF, CH$_3$CN) m/z: $[M + NH_4]^+$ calculated $C_{12}H_{16}N_3O$ 218.13, found 218.13.

1-[4-(1H-Indol-6-yl)phenyl]ethenone ***(16)***

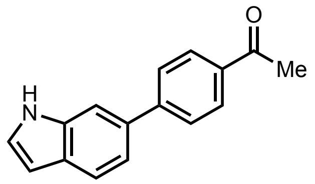

16

Method A: With 0.1 mol% Ni loading, 4-bromophenylethanone (39.8 mg, 0.200 mmol, 1.0 equiv) and (1*H*-indol-6-yl)boronic acid (48.3 mg, 0.300 mmol, 1.5 equiv) were used, purified by flash chromatography (EtOAc:hexane), to give a brown solid (38.1 mg, 0.162 mmol, 81%).

Method B: With 3 mol% Ni loading, 4-bromophenylethanone (39.8 mg, 0.200 mmol, 1.0 equiv) and (1*H*-indol-6-yl)boronic acid (48.3 mg, 0.300 mmol, 1.5 equiv) were used, purified by flash chromatography (EtOAc:hexane), the isolated yield was determined to be 84% (39.5 mg, 0.168 mmol).

Although this compound was synthesized using Pd-SMC in the patent,[34] the detailed data was not found.

^{1}H NMR (400.30 MHz, CDCl$_3$) δ: 8.29 (s, 1H), 8.07 – 8.02 (m, 2H), 7.78 – 7.66 (m, 4H), 7.43 (dd, *J* = 8.3, 1.6 Hz, 1H), 7.29 (dd, *J* = 3.2, 2.4 Hz, 1H), 6.60 (ddd, *J* = 3.1, 2.0, 1.0 Hz, 1H), 2.65 (s, 3H, Me).

^{13}C{^{1}H} NMR (100.67 MHz, CDCl$_3$) δ: 198.0 (s, C=O), 147.1 (s), 136.5 (s), 135.4 (s), 134.2 (s), 129.1 (s), 128.2 (s), 127.4 (s), 125.6 (s), 121.3 (s), 119.8 (s), 110.0 (s), 102.9 (s), 26.8 (s, Me).

HRMS (ESI-TOF, CH$_3$CN) m/z: $[M + K]^+$ calculated for $C_{16}H_{13}NOK$ 274.0629, found 274.0617.

3-(4-(Methylsulfonyl)phenyl)pyridine (**17**)

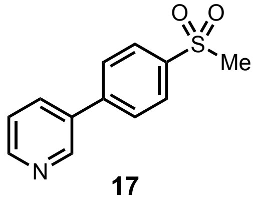

17

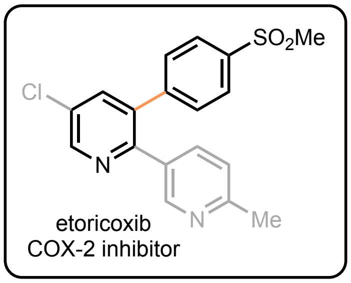

Method A: With 0.5 mol% Ni loading, 1-chloro-4-(methylsulfonyl)benzene (38.1 mg, 0.200 mmol, 1.0 equiv) and pyridin-3-ylboronic acid (36.9 mg, 0.300 mmol, 1.5 equiv) were used, purified by flash chromatography (EtOAc:hexane), to give a white solid (27.2 mg, 0.117 mmol, 58%).

Method B: With 3 mol% Ni loading, 1-chloro-4-(methylsulfonyl)benzene (38.1 mg, 0.200 mmol, 1.0 equiv) and pyridin-3-ylboronic acid (36.9 mg, 0.300 mmol, 1.5 equiv) were used, the NMR yield was determined to be 81%. The crude was purified by flash chromatography (EtOAc:hexane), to give a white solid (35.1 mg, 0.150 mmol, 75%).

The NMR data of the isolated compound are consistent with the literature reports.[35] The isolated compound was used to generate a calibration curve for quantitative GC-FID analysis (for Section 3). We attribute the modest yield observed in our Ni-catalyzed SMC reaction to the limited compatibility of the methylsulfonyl functional group under these conditions, which is consistent with the literature report for Ni(dppf)Cl(*o*-Tol).[36]

^{1}H NMR (400.30 MHz, $CDCl_3$) δ: 8.88 (s, 1H), 8.69 (s, 1H), 8.08 – 8.03 (m, 2H), 7.91 (ddd, *J* = 7.9, 2.4, 1.6 Hz, 1H), 7.78 (dq, *J* = 8.7, 2.2 Hz, 2H), 7.43 (dd, *J* = 7.9, 4.8 Hz, 1H), 3.10 (s, 3H, Me).

^{13}C{^{1}H} NMR (100.67 MHz, $CDCl_3$) δ: 149.9 (s), 148.4 (s), 143.5 (s), 140.2 (s), 135.0 (s), 134.8 (s), 128.4 (s), 128.2 (s), 124.0 (s), 44.7 (s, Me).

LCMS (ESI-TOF, CH_3CN) m/z: $[M + H]^+$ calculated $C_{12}H_{12}NO_2S$ 234.06, found 234.05.

3,6'-Biquinoline (***18***)

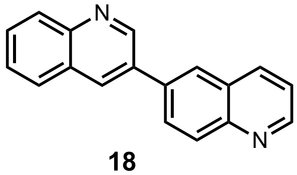

18

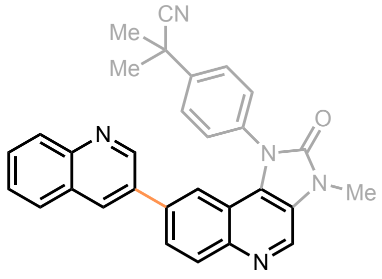

dactolisib
PI3K inhibitor – cancer treatment

Method A: With 0.5 mol% Ni loading, 6-chloroisoquinoline (32.7 mg, 0.200 mmol, 1.0 equiv) and quinolin-3-ylboronic acid (51.9 mg, 0.300 mmol, 1.5 equiv) were used, purified by flash chromatography (EtOAc:hexane), to give a white solid (43.6 mg, 0.170 mmol, 85%).

Method B: With 3 mol% Ni loading, 6-chloroisoquinoline (32.7 mg, 0.200 mmol, 1.0 equiv) and quinolin-3-ylboronic acid (51.9 mg, 0.300 mmol, 1.5 equiv) were used, the isolated yield was determined to be 90% (46.1 mg, 0.180 mmol).

The NMR data of the isolated product are consistent with the literature report.[37]

^{1}H NMR (400.30 MHz, CDCl$_3$) δ: 9.31 (d, *J* = 2.3 Hz, 1H), 8.97 (dd, *J* = 4.3, 1.7 Hz, 1H), 8.43 (d, *J* = 2.3 Hz, 1H), 8.27 (dd, *J* = 8.1, 1.6 Hz, 2H), 8.20 – 8.12 (m, 2H), 8.08 (dd, *J* = 8.7, 2.1 Hz, 1H), 7.92 (dd, *J* = 8.3, 1.5 Hz, 1H), 7.76 (ddd, *J* = 8.4, 6.9, 1.5 Hz, 1H), 7.66 – 7.55 (m, 1H), 7.47 (dd, *J* = 8.3, 4.2 Hz, 1H).

^{13}C{^{1}H} NMR (100.67 MHz, CDCl$_3$) δ: 151.0 (s), 145.0 (s), 148.0 (s), 147.7 (s), 136.5 (s), 136.1 (s), 133.9 (s), 133.1 (s), 130.7 (s), 129.9 (s), 129.5 (s), 129.1 (s), 128.7 (s), 128.2 (s), 128.1 (s), 127.4 (s), 126.3 (s), 122.0 (s).

HRMS (ESI-TOF, CH$_3$CN) m/z: $[M + H]^+$ calculated $C_{18}H_{13}N_2$ 258.1105, found 258.1117.

*(S)-2-((tert-Butoxycarbonyl)amino)-3-(4-(2-imino-2,3-dihydropyrimidin-4-yl)phenyl) propanoic acid (**19**)*

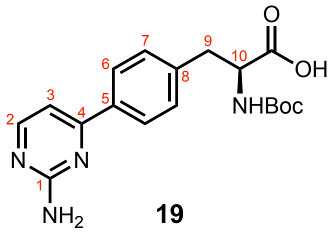

19

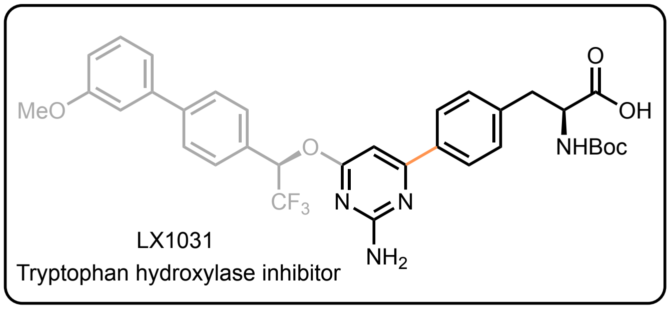

Method A: With 5 mol% Ni loading, 4-chloropyrimidin-2-amine (25.9 mg, 0.200 mmol, 1.0 equiv) and (*S*)-3-(4-boronophenyl)-2-((*tert*-butoxycarbonyl)amino)propanoic acid (92.7 mg, 0.300 mmol, 1.5 equiv) were used, the NMR yield was determined to be 71%. Purification of the product is difficulty. Initial purification by silica column is not effective. Crystallization of the crude in *i*PrOH/heptane give a sufficiently pure product as off-white solid (26 mg, purity > 77% by ^{1}H NMR). The mixture was further purified by HPLC for obtaining characterization data. NMR assignments are based on 2D NMR experiments.

Method B: With 5 mol% Ni loading, 4-chloropyrimidin-2-amine (25.9 mg, 0.200 mmol, 1.0 equiv) and (*S*)-3-(4-boronophenyl)-2-((*tert*-butoxycarbonyl)amino)propanoic acid (92.7 mg, 0.300 mmol, 1.5 equiv) were used, the NMR yield was determined to be 31%.

^{1}H NMR (500.20 MHz, DMSO-d_6) δ 8.26 (d, *J* = 5.2 Hz, 1H, H2), 7.93 (d, *J* = 7.9 Hz, 2H, H6), 7.27 (d, *J* = 8.0 Hz, 2H, H7), 7.08 (d, *J* = 5.2 Hz, 1H, H3), 6.59 (s, 2H, NH_2), 6.28 (s, 1H, NHBoc), 3.93 – 3.87 (m, 1H, H10), 3.10 (dd, *J* = 13.4, 5.0 Hz, 2H, H9), 2.93 (dd, *J* = 13.4, 7.2 Hz, 1H, H9), 1.33 (s, 9H, *t*-Bu). ^{1}H signal due to COOH was not observed.

^{13}C{^{1}H} NMR (100.67 MHz, DMSO-d_6) δ 164.9 (s, C1), 163.8 (s, C4), 158.5 (s, C2), 141.7 (s, C8), 134.1 (s, C5), 129.7 (s, C7), 126.2 (s, C6), 105.6 (s, C3), 77.5 (s, $C(CH_3)_4$), 55.7 (s, C10), 37.0 (s, C9), 28.2 (s, CH_3). ^{13}C signals due to C=O no were not observed.

HRMS (ESI-TOF, CH_3OH) m/z: $[M + H]^+$ calculated for $C_{18}H_{23}N_4O_4$ 359.1714, found 359.1703.

*Ethyl 2-methyl-2-(4-(pyridin-4-yl)phenoxy)propanoate (**20**)*

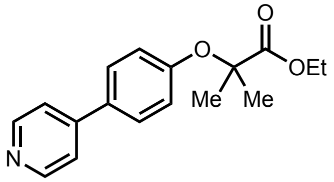

20

Method A: With 0.5 mol% Ni loading, clofibrate (48.5 mg, 0.200 mmol, 1.0 equiv) and pyridin-4-ylboronic acid (36.9 mg, 0.300 mmol, 1.5 equiv) were used, purified by flash chromatography (EtOAc:hexane), to give a colourless oil (49.3 mg, 0.173 mmol, 86%).

Method B: With 3 mol% Ni loading, clofibrate (48.5 mg, 0.200 mmol, 1.0 equiv) and pyridin-4-ylboronic acid (36.9 mg, 0.300 mmol, 1.5 equiv) were used, but the reaction gave a trace amount of product as determined by LCMS and TLC. One possible explanation is that the boronic species underwent rapid protodeboronation in water.

^{1}H NMR (400.30 MHz, $CDCl_3$) δ: 8.70 – 8.54 (m, 2H), 7.60 – 7.52 (m, 2H), 7.50 – 7.43 (m, 2H), 7.01 – 6.86 (m, 2H), 4.25 (q, J = 7.1 Hz, 2H, CH_2 at Et), 1.64 (s, 6H, Me), 1.26 (t, J = 7.2 Hz, 3H, Me at Et).

^{13}C{^{1}H} NMR (100.67 MHz, $CDCl_3$) δ: 174.2 (s, C=O), 156.7 (s), 150.3 (s), 147.9 (s), 131.6 (s), 128.0 (s), 121.3 (s), 119.2 (s), 79.4 (s), 61.7 (s), 25.5 (s), 14.2 (s).

HRMS (ESI-TOF, CH_3CN) m/z: $[M + H]^+$ calculated $C_{17}H_{20}NO_3$ 286.1438, found 286.1453.

*Ethyl 4-(8-(pyridin-4-yl)-5,6-dihydro-11H-benzo[5,6]cyclohepta[1,2-b]pyridin-11-ylidene)piperidine-1-carboxylate (**21**)*

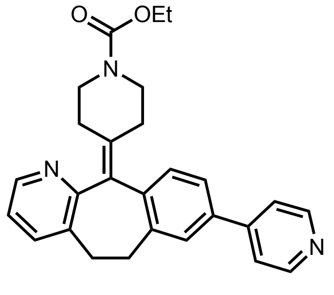

21

Method A: With 0.5 mol% Ni loading, loratadine (76.6 mg, 0.200 mmol, 1.0 equiv) and pyridin-4-ylboronic acid (36.9 mg, 0.300 mmol, 1.5 equiv) were used, purified by flash chromatography (DCM:MeOH), to give a white solid (68.8 mg, 0.162 mmol, 81%).
Method B: With 3 mol% Ni loading, loratadine (76.6 mg, 0.200 mmol, 1.0 equiv) and pyridin-4-ylboronic acid (36.9 mg, 0.300 mmol, 1.5 equiv) were used, but the reaction gave a trace amount of product as determined by LCMS and TLC. One possible explanation is that the boronic species underwent rapid protodeboronation in water.
The NMR data of the isolated product are consistent with the literature report.[38]

^{1}H NMR (400.30 MHz, $CDCl_3$) δ: 8.62 (d, J = 5.1 Hz, 2H), 8.40 (dd, J = 4.7, 1.7 Hz, 1H), 7.45 (ddd, J = 8.9, 4.5, 2.1 Hz, 5H), 7.35 – 7.29 (m, 1H), 7.13 – 7.05 (m, 1H), 4.12 (q, J = 7.7 Hz, 2H), 3.83 (br, 2H), 3.58 – 3.32 (m, 2H), 3.22 – 3.07 (m, 2H), 2.90 (tdd, J = 15.7, 8.4, 4.6 Hz, 2H), 2.59 – 2.22 (m, 4H), 1.24 (t, J = 7.1 Hz, 3H, Me).

$^{13}C\{^1H\}$ NMR (100.67 MHz, $CDCl_3$) δ: 157.3 (s), 155.6 (s), 150.3 (s), 148.0 (s), 146.8 (s), 140.2 (s), 138.7 (s), 137.6 (s), 137.6 (s), 137.2 (s), 134.7 (s), 133.6 (s), 130.2 (s), 127.8 (s), 124.8 (s), 122.4 (s), 121.5 (s), 61.4 (s), 45.0 (s), 44.9 (s), 32.1 (s), 31.7 (s), 30.9 (s), 30.7 (s), 14.8 (s).

HRMS (ESI-TOF, CH_3CN) m/z: $[M + H]^+$ calculated $C_{27}H_{28}N_3O_2$ 426.2176, found 426.2168.

2-(4-(3-(2-(4-Methoxyphenyl)-10H-phenothiazin-10-yl)propyl)piperazin-1-yl)ethan-1-ol (**22**)

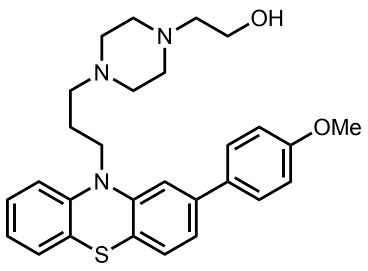

22

Method A: With 3 mol% Ni loading, perphenazine (40.4 mg, 0.100 mmol, 1.0 equiv) and (4-methoxyphenyl)boronic acid (22.8 mg, 0.300 mmol, 1.5 equiv) were used, purified by flash chromatography (DCM:MeOH), to give a white solid (38.8 mg, 0.816 mmol, 82%). For this large electrophile, more Ni loading seems to be needed even using *i*PrOH condition.
Method B: With 5 mol% Ni loading, perphenazine (40.4 mg, 0.100 mmol, 1.0 equiv), (4-methoxyphenyl)boronic acid (22.8 mg, 0.300 mmol, 1.5 equiv) and neopentyl glycol (18.7 mg,

0.180 mmol, 1.8 equiv) were used, the NMR yield was determined to be 94%. The crude was purified by flash chromatography (MeOH:DCM), to give a white solid (41.8 mg, 0.0879 mmol, 88%).

The NMR data of the isolated compound are consistent with the literature report.[39]

^{1}H NMR (400.30 MHz, CDCl$_3$) δ: 7.50 – 7.43 (m, 2H), 7.19 – 7.12 (m, 3H), 7.08 (dd, *J* = 7.9, 1.7 Hz, 1H), 7.02 (d, *J* = 1.7 Hz, 1H), 6.99 – 6.94 (m, 2H), 6.94 – 6.88 (m, 2H), 4.00 (t, *J* = 6.7 Hz, 2H), 3.85 (s, 3H, OMe), 3.60 (t, *J* = 5.4 Hz, 2H), 2.65 – 2.33 (m, 12H), 2.00 (p, *J* = 7.0 Hz, 2H).
^{13}C{^{1}H} NMR (100.67 MHz, CDCl$_3$) δ: δ 159.4 (s), 145.7 (s), 145.3 (s), 140.5 (s), 133.6 (s), 128.1 (s), 127.7 (s), 127.6 (s), 127.4 (s), 125.4 (s), 123.8 (s), 122.6 (s), 121.2 (s), 115.8 (s), 114.3 (s), 114.3 (s), 59.4 (s), 57.7 (s), 55.7 (s), 55.5 (s), 53.1 (s), 52.9 (s), 45.4 (s), 24.5 (s).

HRMS (ESI-TOF, CH$_3$CN) m/z: [M + H]$^+$ calculated for $C_{28}H_{34}N_3O_2S$ 476.2366, found 476.2370.

tert-Butyl (S)-5-amino-4-(5-(4-methoxyphenyl)-1-oxoisoindolin-2-yl)-5-oxopentanoate ***(23)***

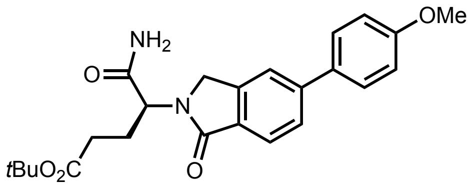

23

Method A: With 1 mol% Ni loading, *tert*-butyl (*S*)-5-amino-4-(5-bromo-1-oxoisoindolin-2-yl)-5-oxopentanoate (79.5 mg, 0.200 mmol, 1.0 equiv) and (4-methoxyphenyl)boronic acid (45.6 mg, 0.300 mmol, 1.5 equiv), purified by flash chromatography (EtOAc:hexane), to give a white solid (76.3 mg, 0.180 mmol, 90%).

Method B: With 3 mol% Ni loading, *tert*-butyl (*S*)-5-amino-4-(5-bromo-1-oxoisoindolin-2-yl)-5-oxopentanoate (79.5 mg, 0.200 mmol, 1.0 equiv) and (4-methoxyphenyl)boronic acid (45.6 mg, 0.300 mmol, 1.5 equiv), the isolated yield was determined to be 80% (67.5 mg, 0.159 mmol).

The NMR assignments of the PROTAC fragment are illustrated in detail in the example provided in **24**.

^{1}H NMR (400.30 MHz, CDCl$_3$) δ: 7.83 (dd, *J* = 7.9, 2.1 Hz, 1H, Ar), 7.65 – 7.59 (m, 2H, Ar), 7.55 – 7.51 (m, 2H, Ar), 7.02 – 6.97 (m, 2H, Ar), 6.49 – 6.38 (m, 1H, NH$_2$), 5.53 – 5.41 (m, 1H, NH$_2$), 4.93 (dd, *J* = 8.6, 6.0 Hz, 1H, NCH), 4.61 – 4.44 (m, 2H, NCH$_2$), 3.86 (s, 3H, OMe), 2.45 – 2.06 (m, 4H, CH$_2$), 1.41 (s, 9H, *t*-Bu).

$^{13}C\{^1H\}$ NMR (100.67 MHz, $CDCl_3$) δ: 171.9 (s, C=O), 171.9 (s, C=O), 169.4 (s, C=O), 160.0 (s), 145.1 (s), 142.5 (s), 132.8 (s), 130.2 (s), 128.7 (s), 127.1 (s), 124.2 (s), 121.2 (s), 114.6 (s), 81.0 (s), 55.5 (s), 54.2 (s), 47.4 (s), 32.1 (s), 28.2 (s), 24.3 (s).

HRMS (ESI-TOF, CH_3CN) m/z: $[M + H]^+$ calculated for $C_{24}H_{29}N_2O_5$ 425.2071, found 425.2056.

*tert-Butyl (S)-4-(2-(1-amino-5-(tert-butoxy)-1,5-dioxopentan-2-yl)-1-oxoisoindolin-5-yl)-3,6-dihydropyridine-1(2H)-carboxylate (**24**)*

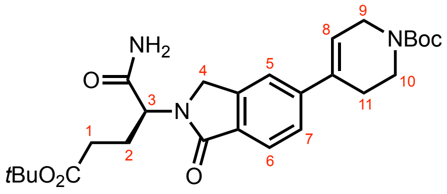

24

Method A: With 1 mol% Ni loading, *tert*-butyl (*S*)-5-amino-4-(5-bromo-1-oxoisoindolin-2-yl)-5-oxopentanoate (79.5 mg, 0.200 mmol, 1.0 equiv) and *tert*-butyl 4-(4,4,5,5-tetramethyl-1,3,2-dioxaborolan-2-yl)-3,6-dihydropyridine-1(2H)-carboxylate (92.8 mg, 0.300 mmol, 1.5 equiv) were used, purified by flash chromatography (EtOAc:hexane), to give a white solid (88.1 mg, 0.176 mmol, 88%).

Method B: With 5 mol% Ni loading, *tert*-butyl (*S*)-5-amino-4-(5-bromo-1-oxoisoindolin-2-yl)-5-oxopentanoate (79.5 mg, 0.200 mmol, 1.0 equiv) and *tert*-butyl 4-(4,4,5,5-tetramethyl-1,3,2-dioxaborolan-2-yl)-3,6-dihydropyridine-1(2H)-carboxylate (92.8 mg, 0.300 mmol, 1.5 equiv) were used, the isolated yield was determined to be 90% (90.0 mg, 0.180 mmol).

A related Pd-catalyzed SMC method for synthesizing the analogue of this compound with Cbz as the protecting group (84% yield) has been reported, utilizing 10 mol% $Pd(dppf)Cl_2$.[40]

^1H NMR (400.30 MHz, $CDCl_3$) δ: 7.77 (dd, J = 8.0, 1.6 Hz, 1H, H6), 7.47 (dd, J = 8.0, 1.5 Hz, 1H, Ar, H7), 7.43 (s, 1H, Ar, H5), 6.44 (s, 1H, NH_2), 6.12 (s, 1H, H8), 5.50 (s, 1H, NH_2), 4.90 (dd, J = 8.7, 6.2 Hz, 1H, H3), 4.53 (d, J = 17.0 Hz, 1H, H4), 4.43 (d, J = 17.1 Hz, 1H, H4), 4.10 (q, J = 3.0 Hz, 2H, H9), 3.65 (t, J = 5.6 Hz, 2H, H10), 2.55 (br s, 2H, H11), 2.43 – 2.09 (m, 4H, H1 & H2), 1.49 (s, 9H, *t*Bu), 1.40 (s, 9H, *t*Bu).

$^{13}C\{^1H\}$ NMR (100.67 MHz, $CDCl_3$) δ: 171.9 (s, C=O), 171.9 (s, C=O), 169.2 (s, C=O), 154.9 (s), 145.0 (s), 142.2 (s), 135.2 (s), 130.7 (s), 125.4 (s), 123.9 (s), 119.6 (s), 81.0 (s), 80.0 (s), 77.4 (s),

54.09 (s), 47.4 (s), 32.0 (s), 29.8 (s), 28.6 (s), 28.2 (s), 27.8 (s), 24.3 (s). The C=O signal at Boc was not detected probably due to the overlapping with other C=O signals.

HRMS (ESI-TOF, CH_3CN) m/z: $[M + H]^+$ calculated for $C_{27}H_{38}N_3O_6$ 500.2755, found 500.2747.

*tert-Butyl 5-(trifluoromethyl)-3',6'-dihydro-[2,4'-bipyridine]-1'(2'H)-carboxylate (**25**)*

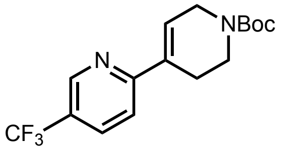

25

Method A: With 1 mol% Ni loading, 2-bromo-5-(trifluoromethyl)pyridine (45.2 mg, 0.200 mmol, 1.0 equiv) and *tert*-butyl 4-(4,4,5,5-tetramethyl-1,3,2-dioxaborolan-2-yl)-3,6-dihydropyridine-1(2H)-carboxylate (92.8 mg, 0.300 mmol, 1.5 equiv) were used, purified by flash chromatography (EtOAc:hexane), to give a white solid (53.0 mg, 0.161 mmol, 81%).

Method B: With 3 mol% Ni loading, 2-bromo-5-(trifluoromethyl)pyridine (45.2 mg, 0.200 mmol, 1.0 equiv) and *tert*-butyl 4-(4,4,5,5-tetramethyl-1,3,2-dioxaborolan-2-yl)-3,6-dihydropyridine-1(2H)-carboxylate (92.8 mg, 0.300 mmol, 1.5 equiv) were used, the isolated yield was determined to be 80% (52.5 mg, 0.160 mmol).

A related Pd-catalyzed SMC method for synthesizing this compound (91% yield) has been reported, utilizing 10 mol% Pd(dppf)Cl_2.[41]

^{1}H NMR (400.30 MHz, $CDCl_3$) δ: 8.81 (dt, J = 2.6, 0.9 Hz, 1H), 7.92 – 7.83 (m, 1H), 7.48 (d, J = 8.4 Hz, 1H), 6.75 (tt, J = 3.4, 1.6 Hz, 1H, =CH), 4.17 (q, J = 3.1 Hz, 2H, CH_2), 3.66 (t, J = 5.7 Hz, 2H, CH_2), 2.65 (dpd, J = 5.8, 2.9, 2.4, 1.4 Hz, 2H, CH_2), 1.49 (s, 9H, *t*Bu).

^{19}F{^{1}H} NMR (376.46 MHz, $CDCl_3$) δ: -62.26 (s).

^{13}C{^{1}H} NMR (100.67 MHz, $CDCl_3$) δ: 160.4 (s, C=O), 154.9 (s), 146.1 (q, J = 4 Hz), 134.8 (s), 133.7 (d, J = 4 Hz), 124.7 (q, J = 33 Hz), 123.9 (q, J = 272 Hz), 118.6 (s, =CH), 80.0 (s, *t*Bu), 44.0 (br s, CH_2), 39.9 (br s, CH_2), 28.6 (s, CH_3 at *t*Bu), 25.9 (s, CH_2).

HRMS (ESI-TOF, CH_3CN) m/z: $[M + Na]^+$ calculated for $C_{16}H_{19}F_3N_2O_2Na$ 351.1291, found 351.1276.

2-(2-Fluoro-5-methylphenyl)-6-methylpyrazine ***(26)***

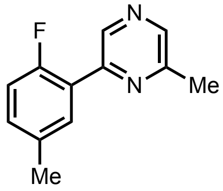

26

Method A: With 0.1 mol% Ni loading, 2-chloro-6-methylpyrazine (25.7 mg, 0.200 mmol, 1 equiv) and (2-fluoro-5-methylphenyl)boronic acid (46.2 mg, 0.300 mmol, 1.5 equiv) were used, purified by flash chromatography (EtOAc:hexane), to give a white solid (35.5 mg, 0.176 mmol, 88%).
Method B: With 3 mol% Ni loading, 2-chloro-6-methylpyrazine (25.7 mg, 0.200 mmol, 1 equiv) and (2-fluoro-5-methylphenyl)boronic acid (46.2 mg, 0.300 mmol, 1.5 equiv) were used, the NMR yield was determined to be 90%.

^{1}H NMR (500.20 MHz, CDCl$_3$) δ: 8.86 (s, 1H), 8.40 (s, 1H), 7.76 (dd, *J* = 7.4, 2.3 Hz, 1H), 7.23 – 7.17 (m, 1H), 7.07 (dd, *J* = 10.9, 8.4 Hz, 1H), 2.64 (s, 3H, Me), 2.40 (s, 3H, Me).

^{19}F{^{1}H} NMR (470.61 MHz, CDCl$_3$) δ: -121.10 (s).

^{13}C{^{1}H} NMR (100.67 MHz, CDCl$_3$) δ: 158.8 (d, *J* = 248 Hz), 153.6 (s), 148.5 (d, *J* = 3 Hz), 142.8 (s), 142.5 (d, *J* = 12 Hz), 134.4 (d, *J* = 4 Hz), 131.9 (d, *J* = 8 Hz), 131.2 (d, *J* = 3 Hz), 124.2 (d, *J* = 13 Hz), 116.2 (d, *J* = 23 Hz), 21.9 (s, Me), 20.8 (s, Me).

HRMS (ESI-TOF, CH$_3$CN) m/z: [M + H]$^+$ calculated for $C_{12}H_{12}FN_2$ 203.0979, found 203.0983.

Scale-up method

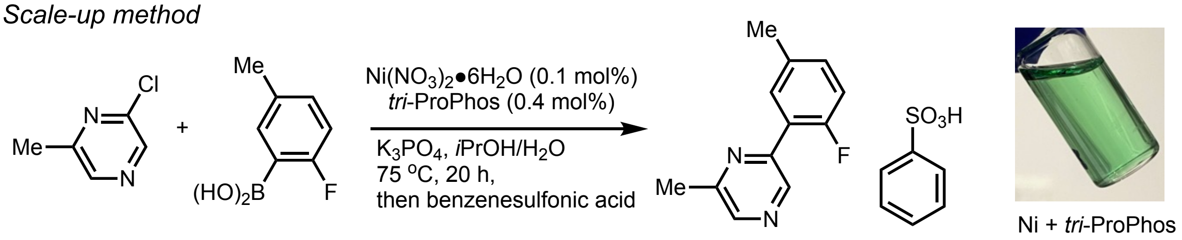

The reaction was performed in a 250 mL ChemGlass reactor in a glovebox under nitrogen flow. 2-Chloro-6-methylpyrazine (12.84 g, 100 mmol, 1.00 equiv) was dissolved in *i*PrOH (150 mL) and was added to the reactor by addition to a disposable fritted filter with drainage into the reactor. 2-Fluoro-5-methylphenylboronic acid (20.04 g, 130 mmol, 1.30 equiv) was added along with an

additional *i*PrOH (50 mL). Solid potassium phosphate trihydrate (66.59 g, 250 mmol, 2.50 equiv) was added. Water (50 mL) was then added, and it was observed the water layer was a saturated solution. The reactor was degassed by stirring the reactor while a nitrogen stream was bubbled through the contents. The jacket of the reactor was allowed to begin warming to 75 °C (jacket temperature). An additional degassed *i*PrOH (~30 mL) was added to the reactor, and ~20 mL of *i*PrOH was set aside for catalyst preparation.

The catalyst was prepared using $Ni(NO_3)_2•6H_2O$ (28.9 mg, 0.10 mmol, 0.1 mol%) and adding <10 mL of *i*PrOH for dissolution. The nickel solution was added to a vial of *tri*-ProPhos (**L3**, 104.9 mg, 0.40 mmol, 0.4 mol%) to form a green solution after shaking the vial. The catalyst solution was added to the reactor, and the remainder of the *i*PrOH was added for washing the catalyst vial into the reactor. Upon the reactor reaching 75 °C, the aqueous phase became more homogeneous, with better solubility of the base. The reaction was allowed to stir overnight, and the following morning, stirring was ceased, and the organic and aqueous layers were allowed to split. The lower aqueous layer was collected as a homogeneous solution that was observed to form precipitates upon cooling. *Note: a rag layer was observed at the split, and some materials stuck to the sides of the reactor. A portion of the rag was drained into the aqueous layer.* The organic extracts were collected, and the sides of the reactor were washed twice with small amounts of *i*PrOH with a total volume of ~10 mL. The saved organic extracts were transferred to a 1L reactor via addition to a disposable fritted funnel for polish filtration. The contents were diluted to 300 mL (KF = 8.20 wt% water) and were distilled to ~100 mL at ~34 °C distillation temperature at 100 mbar. 200 mL of *i*PrOH was added to the reactor to dilute to 300 mL (KF = 1.39 wt% water). Distillation to 100 mL was performed as previously described (KF = 0.721 wt% water). The jacket was set to temperature of 20 °C. A small scale test was performed by adding benzenesulfonic acid (0.194g) in *i*PrOH (1.0 mL) to ~1.0 mL of solution. 2.0 mL of heptane was added, and a slurry was formed. Benzene sulfonic acid was added to a round bottom flask and was dissolved in *i*PrOH (100 mL) and was added to the reactor via addition funnel. A slurry was observed to form. The material from the test precipitation was added to the reactor, and previous samplings, along with 100 mL heptane were added to the addition funnel. The material was added to the reactor in a dropwise manner over ~25-30 min. An additional 100 mL of heptane was added dropwise over ~15 min. The mixture was aged for the evening, and the following morning, the material was filtered in a disposable filter funnel with vacuum via a side arm flask. The reactor was washed with 200 mL of heptane, and upon largely deliquoring the initial solid cake, the heptane was used for solid wash. After largely deliquoring the solid, the material was worked with a spatual to aid in the removal of

the final solvent. The material was dried by vacuum via a side-arm flask for several hours with a final mass of the desired product **26** •$C_6H_5SO_3H$ (31.88 g, 88.51 mmol, 89%).

^{1}H NMR (400.30 MHz, DMSO-*d*$_6$) δ: 9.78 (s, 1H, SO_3H), 8.80 (d, *J* = 2.7 Hz, 1H), 8.54 (s, 1H), 7.72 (dd, *J* = 7.5, 2.3 Hz, 1H), 7.65 – 7.58 (m, 2H), 7.35 – 7.30 (m, 4H), 7.25 (dd, *J* = 11.1, 8.4 Hz, 1H), 2.58 (s, 3H, Me), 2.36 (s, 3H, Me).

^{19}F{^{1}H} NMR (376.46 MHz, DMSO-*d*$_6$) δ: -121.35 (s).

^{13}C{^{1}H} NMR (100.67 MHz, DMSO-*d*$_6$) δ: 158.1 (d, *J* = 246 Hz), 153.6 (s), 148.1 (s), 147.4 (d, *J* = 3 Hz), 143.1 (s), 141.6 (d, *J* = 11 Hz), 134.2 (d, *J* = 4 Hz), 132.1 (d, *J* = 8 Hz), 130.9 (d, *J* = 3 Hz), 128.5 (s), 127.7 (s), 125.5 (s), 123.6 (d, *J* = 13 Hz), 116.1 (d, *J* = 23 Hz), 21.3 (s, Me), 20.2 (s, Me).

3-(2-Methylphenyl)pyridine ***(S3)***

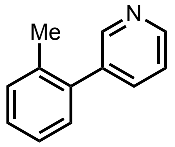

S3

Method A: With 3 mol% Ni loading, 2-bromotoluene (24.1 µL, 0.200 mmol, 1.0 equiv) and 3-pyridinylboronic acid (36.9 mg, 0.300 mmol, 1.5 equiv) were used, purified by flash chromatography (EtOAc:hexane), to give a colourless oil (29.2 mg, 0.73 mmol, 86%).

Method B: With 3 mol% Ni loading, 3-chloropyridine (19.0 µL, 0.200 mmol, 1.0 equiv) and 4,4,5,5-tetramethyl-2-(o-tolyl)-1,3,2-dioxaborolane (65.4 mg, 0.300 mmol, 1.5 equiv) were used, the NMR yield was determined to be 91%. The crude was purified by flash chromatography (EtOAc:hexane), to give a colourless oil (28.5 mg, 0.168 mmol, 84%).

The NMR data of the isolated compound are consistent with the literature report.[8] The isolated product was used to generate a calibration curve for quantitative GC-FID analysis (for Section 6).

^{1}H NMR (400.30 MHz, $CDCl_3$) δ: 8.59 (d, *J* = 5.5 Hz, 2H), 7.64 (dq, *J* = 7.9, 1.6, 1.1 Hz, 1H), 7.37 – 7.19 (m, 5H), 2.27 (s, 3H, Me).

^{13}C{^{1}H} NMR (100.67 MHz, $CDCl_3$) δ: 150.1 (s), 148.2 (s), 138.2 (s), 137.6 (s), 136.6 (s), 135.7 (s), 130.7 (s), 130.0 (s), 128.2 (s), 126.2 (s), 123.1 (s), 20.5 (s, Me).

LCMS (ESI-TOF, CH_3CN) m/z: $[M + H]^+$ calculated $C_{12}H_{12}N$ 171.10, found 171.10.

5. Kinetic Studies of Ni-SMC

5.1 General procedure of kinetic experiments

Followed by general procedure described in Section 3.1, $NiCl_2•6H_2O$ (1.0 equiv) and a phosphine (4.0 equiv, *tri*-ProPhos in most cases) were used for preparing stock solutions and the model reaction 1 was chosen for kinetic studies. In most cases, 4-bromoacetophenone **5** and pyridin-3-ylboronic acid **6** were weighed into a 2 mL crimp-top vial. Solid K_3PO_4 was added to the vial. Subsequently, appropriate volumes of the Ni phosphine stock solution and deoxygenated *i*PrOH (or *i*PrOD-d_8) were added to reach a total volume of 0.4 mL (or 2 mL for the reactions run in 4 mL vial). The vial was sealed and removed from the glove box. 0.1 mL of H_2O (or D_2O) was added to the vial (if applicable) and the vial was placed in a shaker. The reaction mixture was heated to 80 °C with agitation at 800 rpm. Note: the reaction mixture turned to be homogeneous within 0.5 hour after heating. Reaction progress was monitored by preparing a series of identical samples. At specific time points, a sample was removed from the shaker. Each sample was extracted with ethyl acetate, and the resulting organic layer was collected and washed with water. An aliquot of the organic layer was analyzed by gas chromatography (GC-FID) (Calibration curve for the isolated product **7** was created to determine their R_f values for quantitative analysis). Reported yield error bars reflect the standard deviation from duplicate experiments.

Note: The unique kinetic reaction profiles observed in these experiments do not follow a typical first-order or zero-order behavior, underscoring the complexity of Ni-catalyzed Suzuki–Miyaura cross-coupling (SMC) involving heterocyclic substrates. The S-shaped or linear trends in the reaction curves are consistent with prior studies using analogous model systems. [8] The initially slow reaction rate (i.e., induction period usually first 2 hours) is likely due to sluggish catalyst activation at the low catalyst loading used as well as the solubilization of K_3PO_4. As the system reaches equilibrium among base, boronic acid/ester, and their corresponding boronate species, the reaction profile transitions to a more linear phase. Toward the end of the reaction, a decline in [base] and/or catalyst deactivation may contribute to the observed plateauing of the rate (i.e., reduced catalytic activity).

Table S3. Reaction Variables of Kinetic Experiments Using Model Reaction 1.

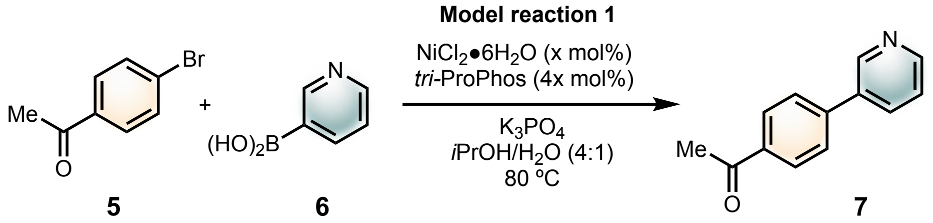

Entry	[Ni] (M)	[5] (M)	[6] (M)	[K_3PO_4] (M)
**1**	3.0×10^{-5}	0.20	0.26	0.40
**2**	4.5×10^{-5}	0.20	0.26	0.40
**3**	3.0×10^{-5}	0.40	0.26	0.40
**4**	3.0×10^{-5}	0.20	0.40	0.40
**5**	3.0×10^{-5}	0.20	0.26	0.60
**6**[a]	3.0×10^{-5}	0.20	0.26	0.60

[a]Kinetic isotope effect experiment run in *i*PrOD-d_8/D_2O.

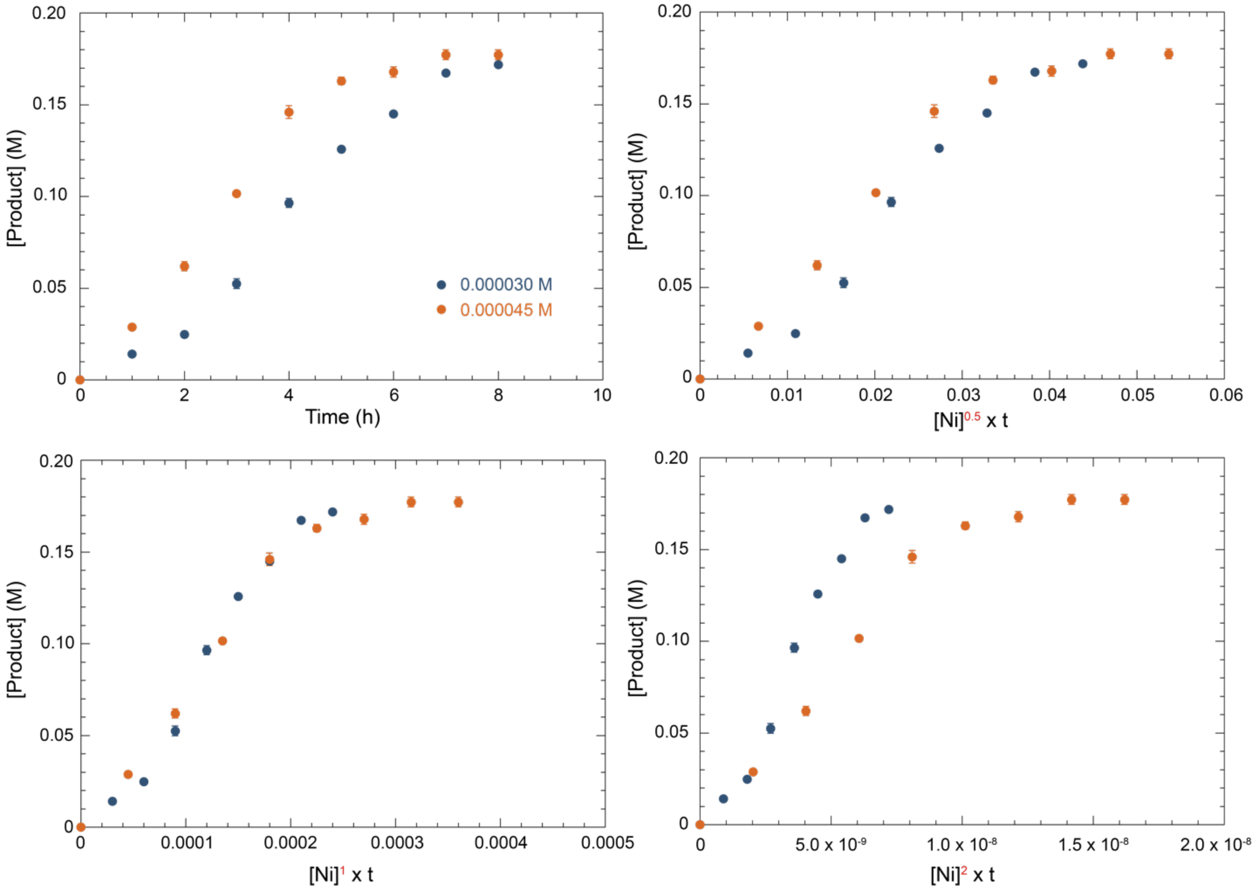

Figure S6. Determination of the order of the Ni catalyst, showing the first-order dependence is the best fit.

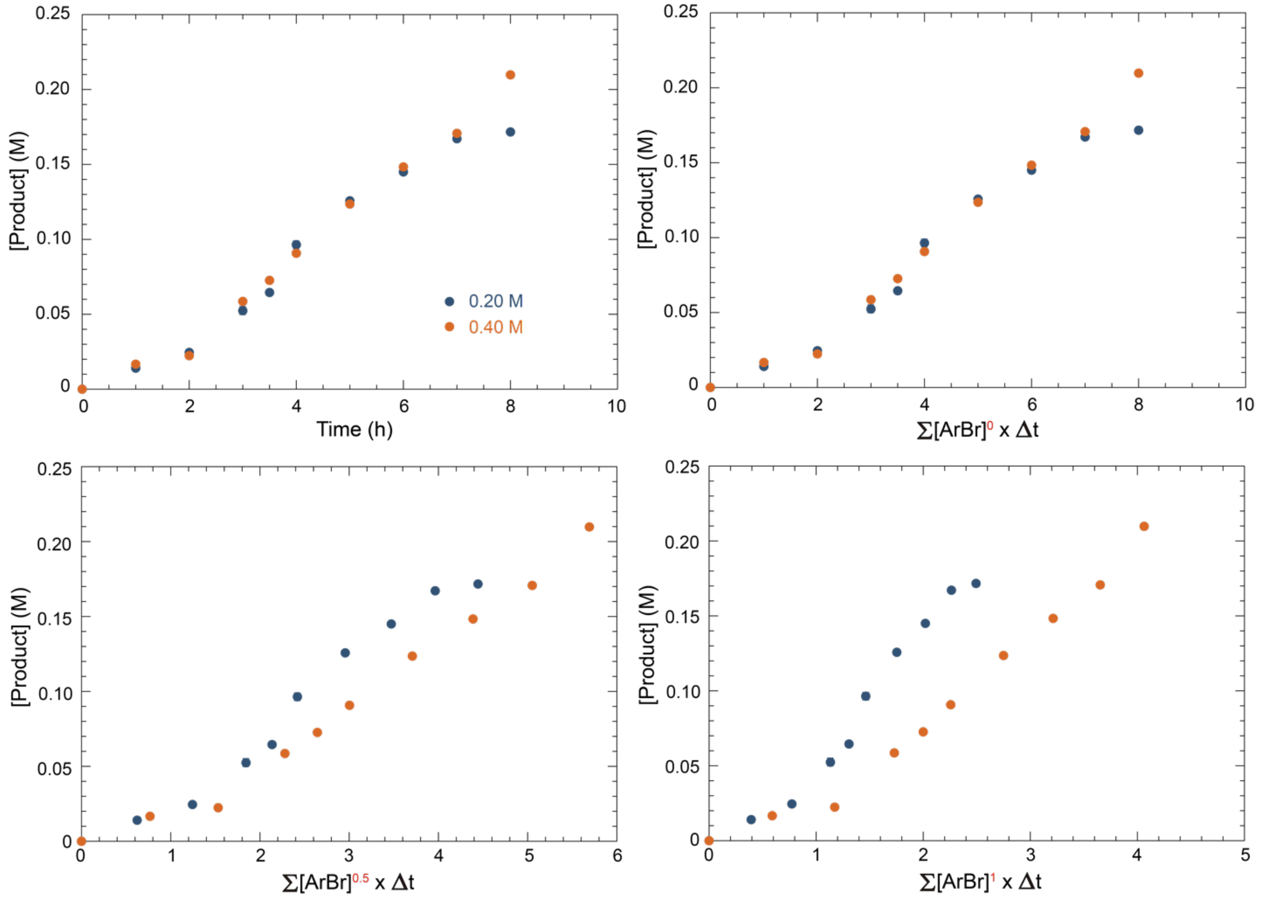

Figure S7. Determination of the order of ArBr **5**, showing the zero-order dependence is the best fit.

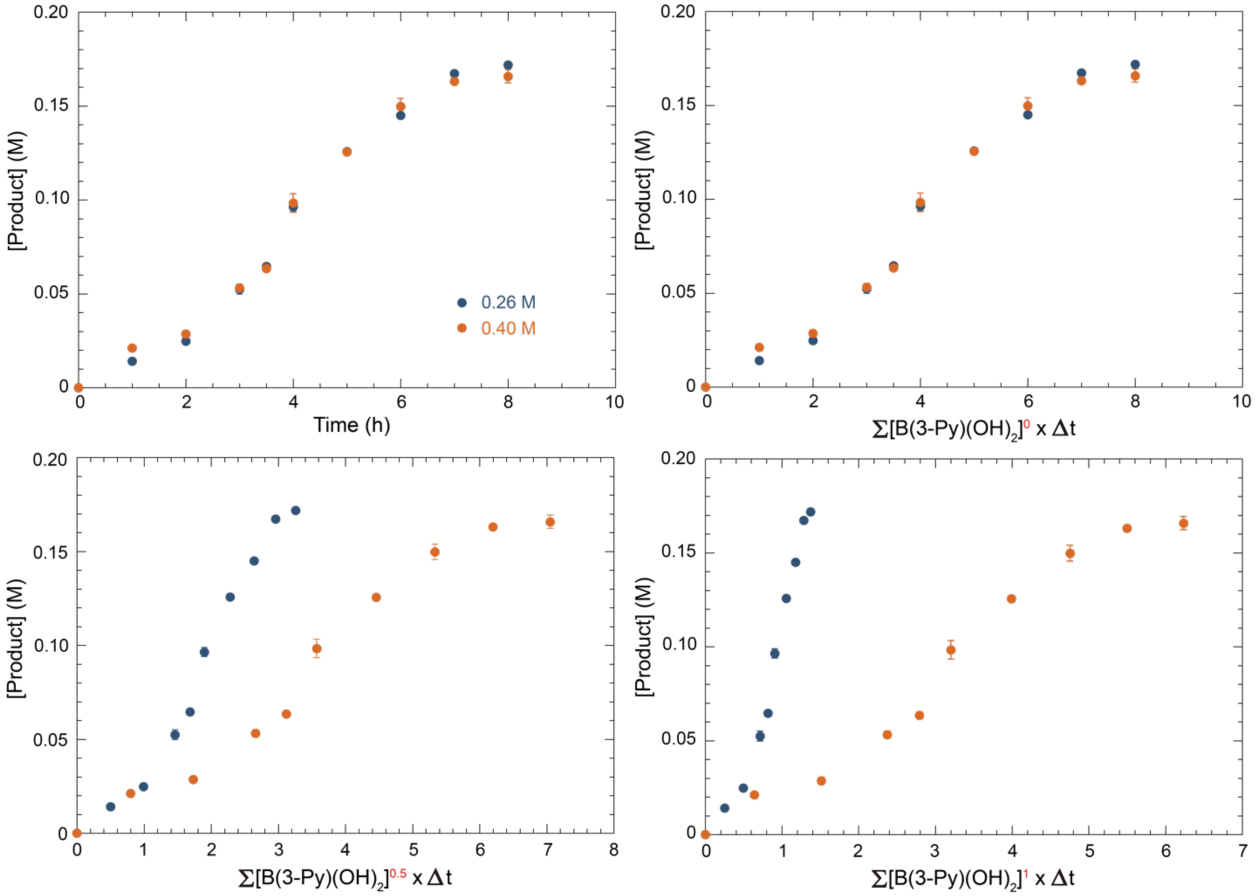

Figure S8. Determination of the order of $B(3\text{-}Py)(OH)_2$ **6**, showing the zero-order dependence is the best fit.

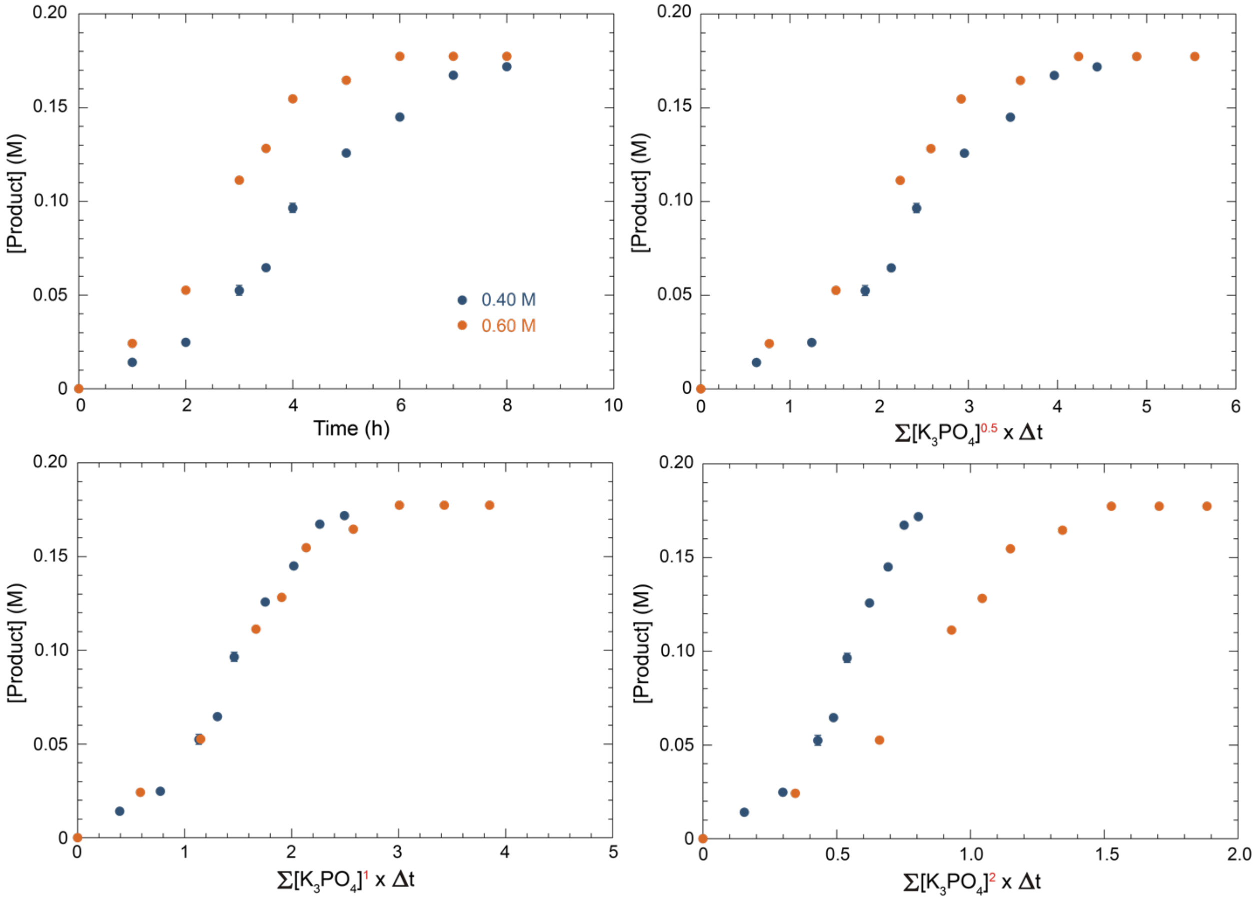

Figure S9. Determination of the order of [K_3PO_4], showing the first-order dependence is the best fit.

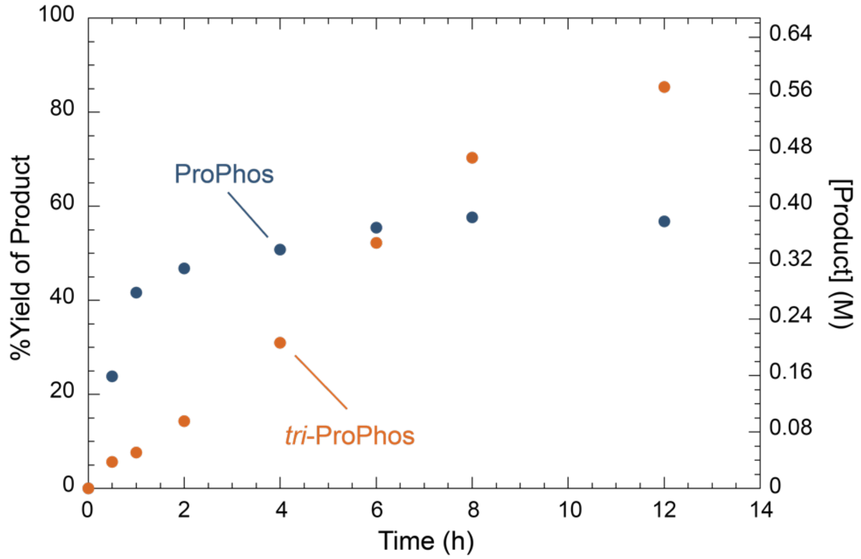

Figure S10. Cross-coupling model reaction 1 of 4-bromoacetophenone **5** and B(3-Py)(OH)$_2$ **6** using 0.03 mol% $NiCl_2•6H_2O$ and 0.12 mol% ProPhos. Condition: [**5**] = 0.667 M; [**6**] = 1.00 M; [Ni] = 2 x 10^{-4} M; [*tri*-ProPhos] = 8 x 10^{-4} M; K_3PO_4 = 106 mg; Time points = 0.5 h, 1 h, 2 h, 4 h, 6 h, 8 h and 12 h.

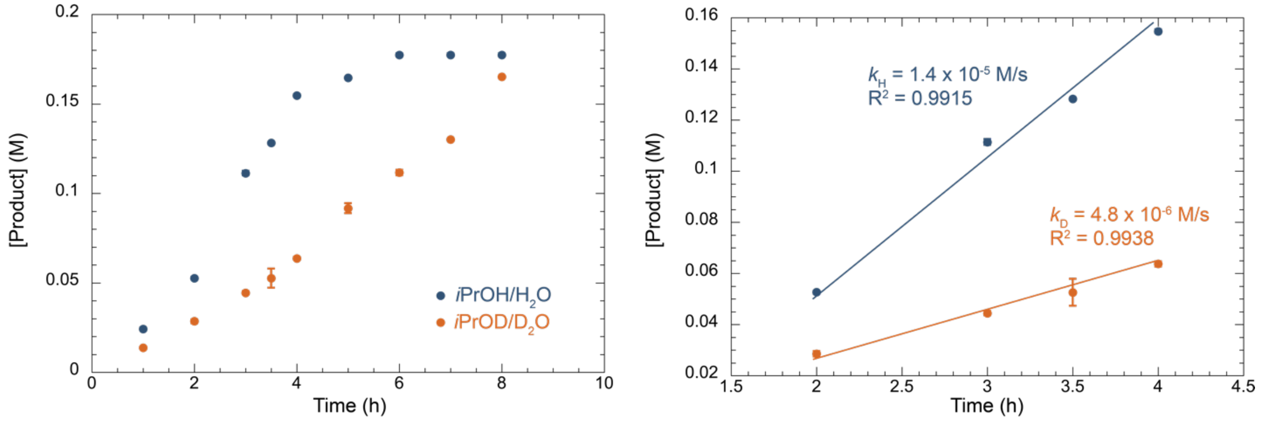

Figure S11. Determination of kinetic isotope effect (KIE) using the model reaction 1 of 4-bromoacetophenone **5** and B(3-Py)(OH)$_2$ **6**.

6. Organometallic Studies

6.1 Resting state analysis of the catalytic system using ProPhos

General procedure for detecting the catalyst resting state

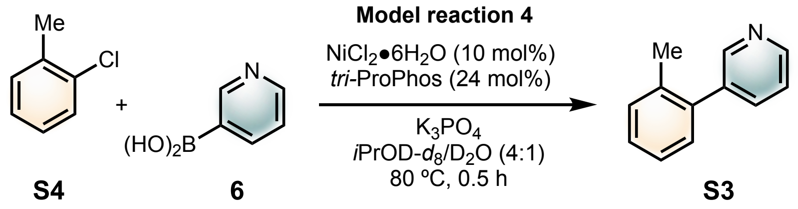

In a nitrogen-filled glove box, $NiCl_2$•$6H_2O$ (2.4 mg, 0.010 mmol, 10 mol%) and *tri*-ProPhos (6.0 mg, 0.024 mmol, 24 mol%) were combined in a 2 mL glass vial containing 0.4 mL of *i*PrOD-d_8. The vial was sealed, removed from the glove box, and placed on a shaker. The mixture was then heated to 70 °C while agitating at 800 rpm for 30 min, resulting in a red stock solution, which was subsequently brought back into the glove box for further use.

In a 2 mL crimp-top vial, B(3-Py)$(OH)_2$ **6** (15 mg, 0.12 mmol, 1.2 equiv) and solid K_3PO_4 (32 mg, 0.15 mmol, 1.5 equiv) were weighed, followed by the addition of 2-chlorotoluene **S4** (12 µL, 0.10 mmol, 1.0 equiv) via syringe transfer. The Ni phosphine stock solution was then completely added to the crimp-top vial, which was subsequently sealed and removed from the glove box. Then, 0.1 mL of deoxygenated D_2O was added to the vial before being placed on a shaker. The reaction mixture was heated to 80°C with agitation at 800 rpm. After 30 min, the sample was withdrawn from the shaker, cooled to room temperature. The homogenous mixture was transferred to an NMR tube for $^{31}P\{^1H\}$ NMR analysis to investigate the Ni speciation.

Parallel experiments under identical conditions were conducted to verify catalytic conversion. The sample were withdrawn at specific time points, cooled to room temperature, and extracted with ethyl acetate. The organic layer was collected, washed with water, and an aliquot was subjected to GC-MS analysis.

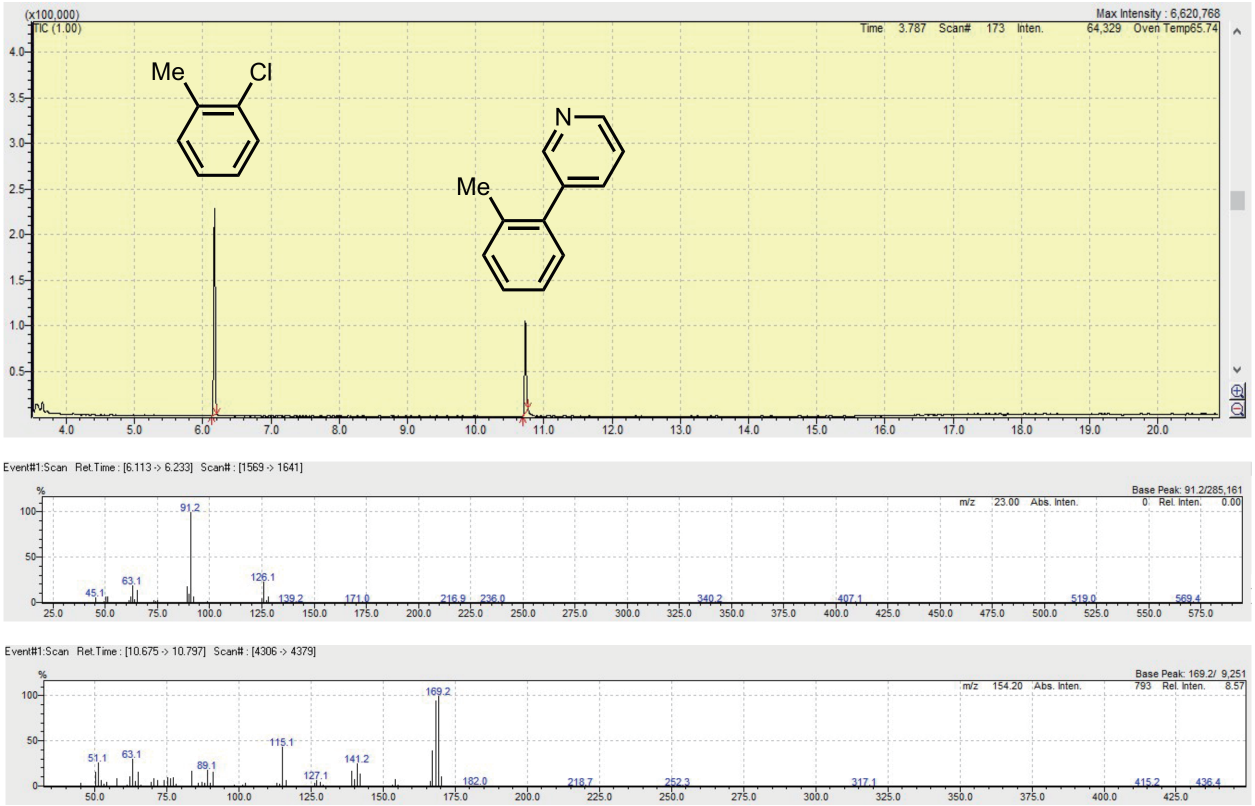

Figure S12. Gas chromatography and mass spectrometry (GC-MS) analysis of Ni-SMC of 2-chlorotoluene **S4** and B(3-Py)$(OH)_2$ **6** using 10 mol% $NiCl_2$•$6H_2O$ and 40 mol% *tri*-ProPhos at 80 ºC for 30 min. The mass spectrum (top) corresponding to the peak at 6.113 min matches unreacted 2-chlorotoluene **S4**, while the mass spectrum (bottom) corresponding to the peak at 10.675 min is consistent with the SMC product **S3**.

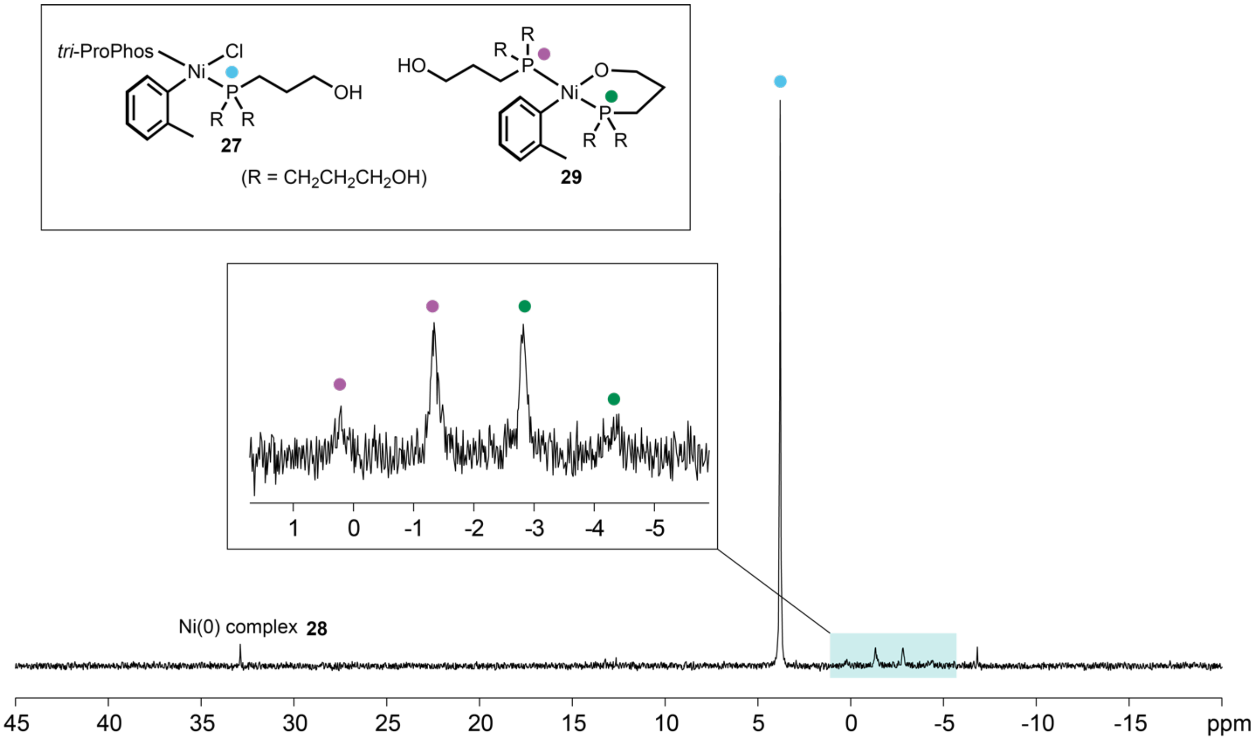

Figure S13. $^{31}P\{^1H\}$ NMR (202.47 MHz, *i*PrOD-d_8/H_2O = 4:1) spectrum of Ni-SMC of 2-chlorotoluene **S3** and B(3-Py)$(OH)_2$ **6** using 10 mol% $NiCl_2{\bullet}6H_2O$ and 40 mol% ProPhos at 80 °C for 30 min, showing the major Ni complex is Ni(*tri*-ProPhos)$_2$Cl(*o*-Tol) **27** and minor species including a Ni metalacyclic complex **29**, a tentatively assigned Ni(0) complex and unidentified species around -7 ppm.

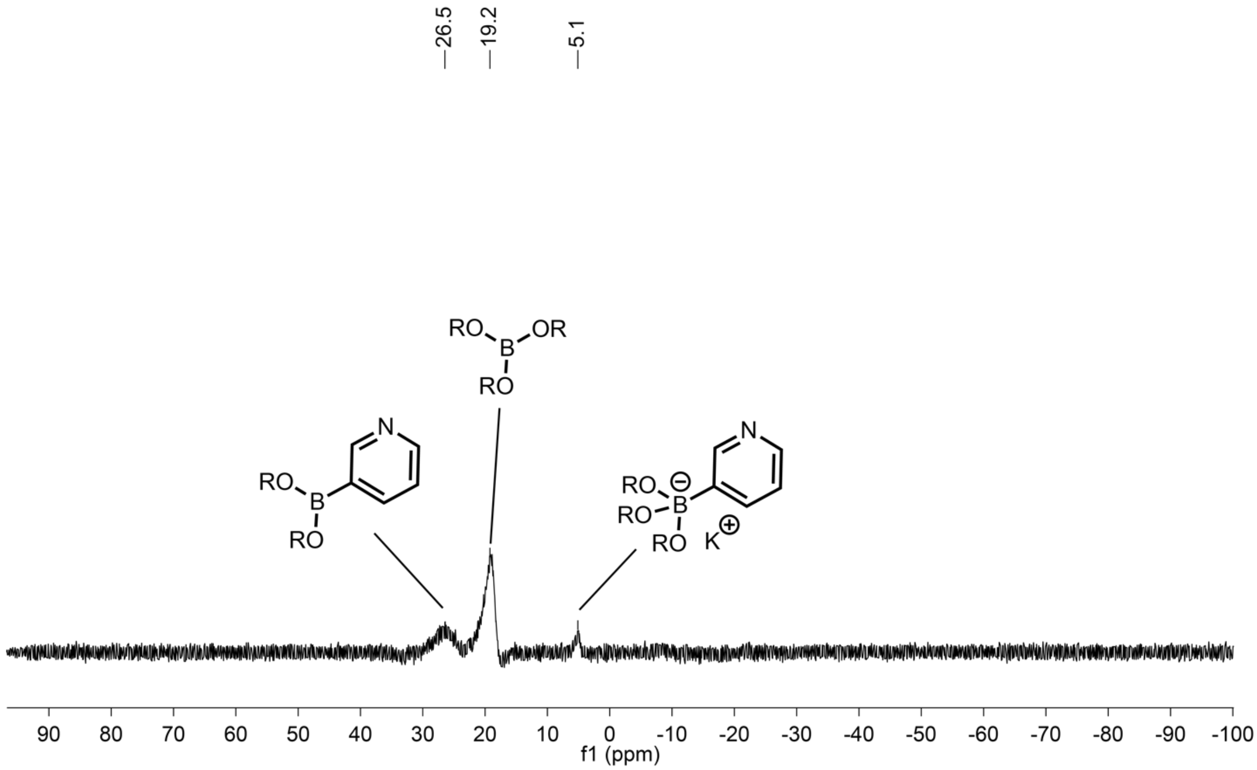

Figure S14. ^{11}B NMR (128.38 MHz, *i*PrOD-d_8/H_2O = 4:1) spectrum of Ni-SMC of 2-chlorotoluene and B(3-Py)$(OH)_2$ **6** using 10 mol% $NiCl_2$•$6H_2O$ and 40 mol% ProPhos at 80 °C for 30 min, showing the boronic species. R = H or *i*Pr. NMR shifts for these tentatively assigned boronic species based on the typical ^{11}B NMR shifts for analogues.[42] The mixture clearly indicates that an equilibrium may be present between the three-coordinate boronic acid/ester (e.g., **6**) and the four-coordinate boronate species (e.g., **30**). $B(OR)_3$ is the byproduct from the SMC.

6.2 Transmetalation of (*tri*-ProPhos)Ni complexes in isopropanol

General experimental procedure

Ni(*tri*-ProPhos)$_2$Cl(*o*-Tol) **27** (7.0 – 8.2 µmol, 1.0 equiv) was weighed into a 4 mL vial containing a stir bar. B(3-Py)(OH)$_2$ **6** (5.0 equiv) or K[B(3-Py)(OH)$_3$] **30** (5.0 equiv) or K_3PO_4 (10 equiv) was then added to the vial, followed by the addition of dry K_3PO_4 (10 equiv), if applicable. The appropriate amounts of *i*PrOD-*d*$_8$ and H_2O (if applicable) were added to the reaction mixture, which was then transferred into a J. Young NMR tube. *Note: a small amount of water was added to improve the solubility of salts such as K[B(3-Py)(OH)$_3$]* ***30*** *and K_3PO_4.* The tube's headspace was evacuated and backfilled with nitrogen, sealed, and placed in an oil bath at 70 °C when required. Reaction progress was monitored via $^{31}P\{^1H\}$ NMR spectroscopy using PPh_3 in a sealed capillary as the internal standard (in most cases). For GC analysis, the reaction mixture was diluted with ethyl acetate, washed with water, and the organic layer was collected and analyzed by GC-FID using *n*-decane as the internal standard.

*<u>Reaction of Ni(tri-ProPhos)₂Cl(o-Tol) **27** and B(3-Py)(OH)₂ **6**</u>*

Under *i*PrOH conditions, no direct coordination was observed between the *tri*-ProPhos ligand and boronic acid **6** (Figure S15). This lack of interaction is presumably due to competition between the solvent (*i*PrOH) and the -OH groups of *tri*-ProPhos for coordination with boronic acid **6** (Scheme S4) Therefore, pre-coordination of *tri*-ProPhos with **6** prior to transmetalation appears unlikely.

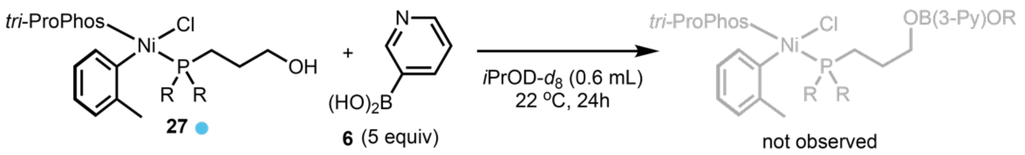

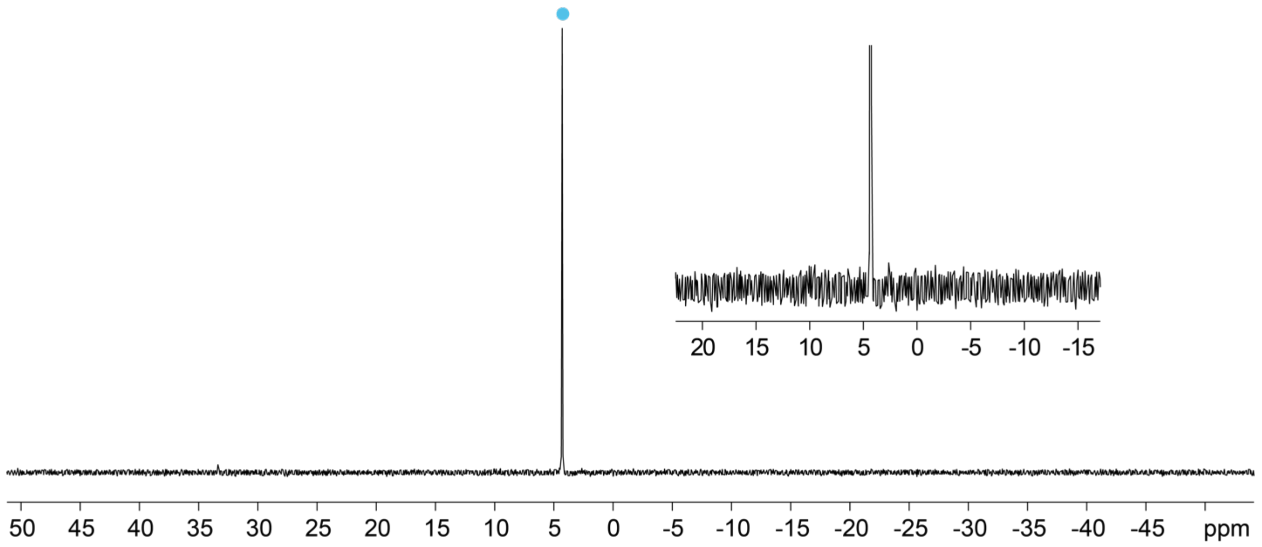

Figure S15. $^{31}P\{^1H\}$ NMR (202.47 MHz, *i*PrOD-d_8) spectrum of the reaction of Ni(*tri*-ProPhos)$_2$Cl(*o*-Tol) **27** and B(3-Py)(OH)$_2$ **6**. The inset shows no observable coordination between the (*tri*-ProPhos)Ni complex and the boronic acid in this polar solvent, suggesting a mechanistic pathway different from that observed with the (ProPhos)Ni catalyst in the less polar THF system.[2]

Scheme S4. Proposed Competitive Coordination of Boronic Acid **6** Between the Resting State Complex and Solvent. R = $(CH_2)_3OH$, R' = H or *i*Pr.

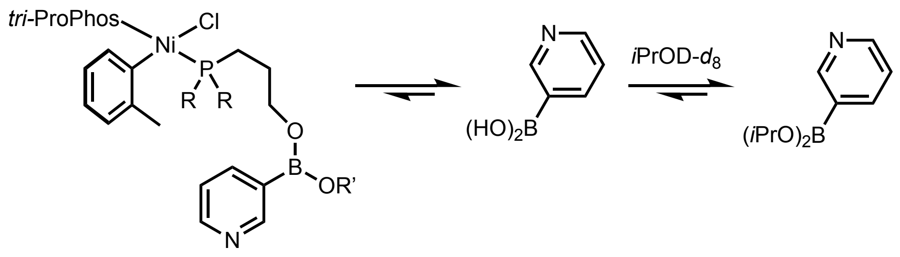

Reaction of Ni(tri-ProPhos)$_2$Cl(o-Tol) ***27*** *and K[B(3-Py)(OH)$_3$]* ***30***

Given our observation of a potential equilibrium between the three-coordinate boronic acid/ester and the four-coordinate boronate species (Figure S14), we considered if the resting-state complex Ni(*tri*-ProPhos)$_2$Cl(*o*-Tol) **27** might directly engage in transmetalation with the boronate species, such as K[BAr(OR)$_3$] (R = *i*Pr or H)—a pathway commonly proposed in boronate-mediated transmetalation.[43] To evaluate this possibility, we treated the Ni complex with isolated K[B(3-Py)(OH)$_3$] **30** in *i*PrOD-*d*$_8$. Notably, we observed the immediate formation of a Ni-metallacyclic complex **29** (Figure 16), accompanied by only trace amounts of the diaryl product, as detected by GC. These findings suggest that: 1) the direct boronate transmetalation pathway is unlikely to operate in this system; 2) although the boronate species is generated during catalysis, it likely acts as a base to dehydrohalogenate Ni(*tri*-ProPhos)$_2$ArX, leading to the formation of a Ni metallacycle prior to transmetalation.

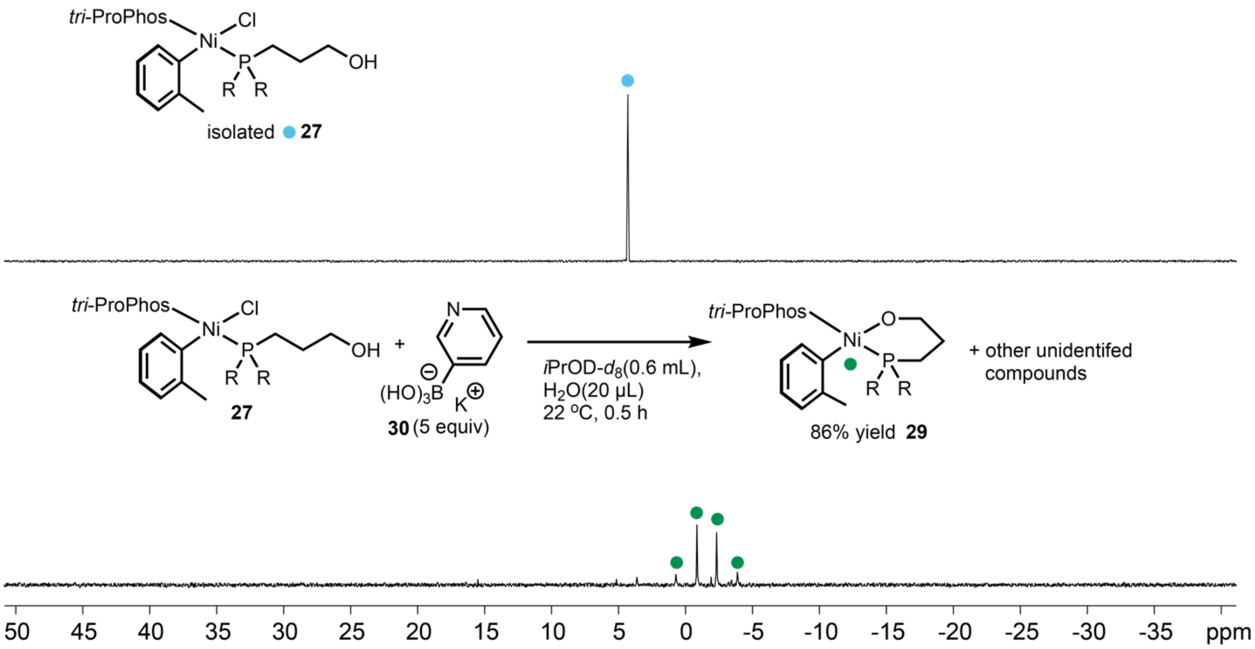

Figure S16. $^{31}P\{^1H\}$ NMR (202.47 MHz, *i*PrOD-*d*$_8$) spectrum of the reaction of Ni(*tri*-ProPhos)$_2$Cl(*o*-Tol) **27** and K[B(3-Py)(OH)$_3$] **30**. R = $(CH_2)_3OH$. The yield of the resulting metallacycle **29** was estimated by comparing its integral to those of all phosphorus-containing species in the mixture.

*Reaction of Ni(tri-ProPhos) metallacycle **29** and B(3-Py)(OH)$_2$ **6***

To test if a Ni metalacyclic complex is eligible for direct transmetalation of boronic acids, we treated the isolated complex **29** with B(3-Py)(OH)$_2$ **6** at 70 °C (Figure S17). The transmetalation occurred rapidly, giving the 70% biaryl product **S3** within 0.5 h. The identical transmetalation also occurred slowly at room temperature, which gave a comparable yield of 68% after 24 h (Figure S18).

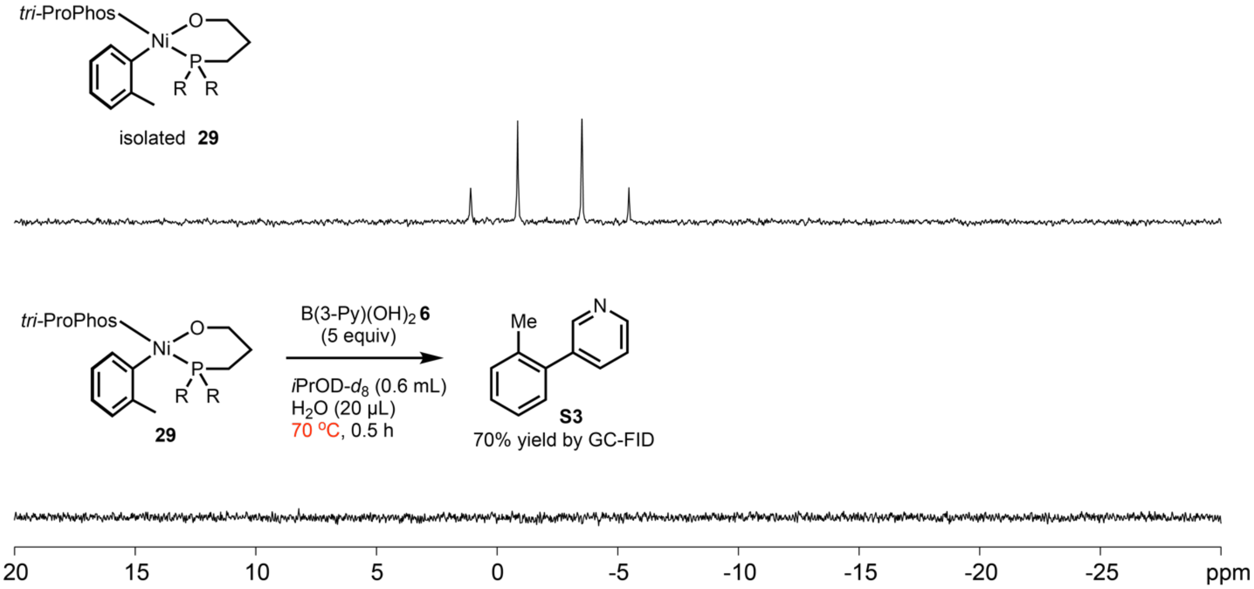

Figure S17. $^{31}P\{^1H\}$ NMR (162.04 MHz, *i*PrOD-*d*$_8$) spectrum of the reaction of Ni metallacycle **29** and B(3-Py)(OH)$_2$ **6** at 70 °C. R = $(CH_2)_3OH$.

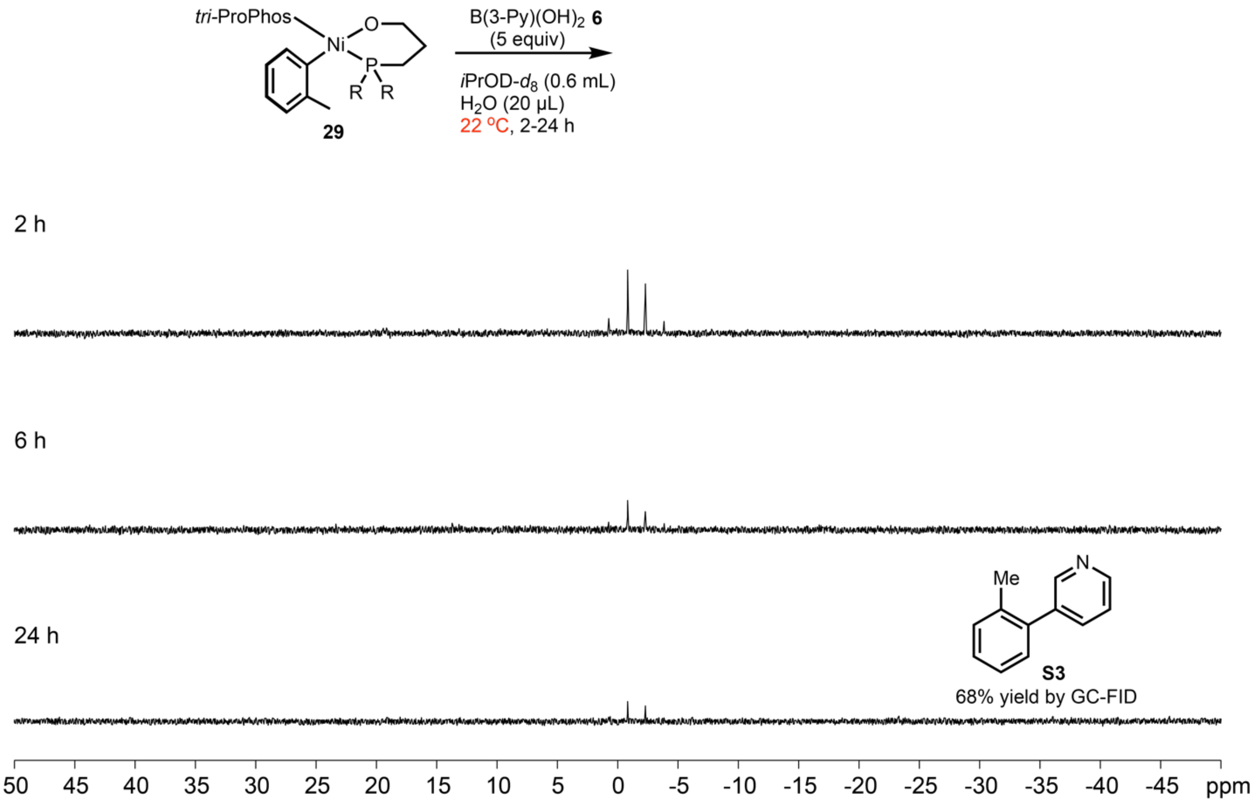

Figure S18. $^{31}P\{^{1}H\}$ NMR (162.04 MHz, *i*PrOD-d_8) spectrum of the reaction of Ni metallacycle **29** and B(3-Py)$(OH)_2$ **6** at room temperature. R = $(CH_2)_3OH$.

*Reaction of Ni(tri-ProPhos)$_2$Cl(o-Tol) **27** and K_3PO_4*

Considering the feasibility of direct transmetalation between the Ni metallacycle **29** and the boronic acid **6**, along with the observed zero-order dependence on [**6**], these findings suggest that transmetalation proceeds rapidly under catalytic conditions. Moreover, the rate law shows a first-order dependence on [K_3PO_4], leading us to propose that the turnover-limiting step is the dehydrohalogenation of the resting-state complex Ni(*tri*-ProPhos)$_2$ArX, which generates a Ni metallacyclic intermediate that is highly reactive toward transmetalation. Thus, we want to investigate if this dehydrohalogenation is slow when K_3PO_4 is used.

Treatment of isolated Ni(*tri*-ProPhos)$_2$Cl(*o*-Tol) **27** (1 equiv) with K_3PO_4 (10 equiv) resulted in a broad $^{31}P\{^{1}H\}$ NMR signal centered around 3 ppm (Figure S19 top), suggesting a dynamic equilibrium occurring in solution, with exchange processes fast on the NMR timescale. Based on our ability to isolate the corresponding Ni metallacycle under more strongly basic conditions, we propose that this equilibrium likely exists between Ni(*tri*-ProPhos)$_2$Cl(*o*-Tol) **27** and the metallacyclic complex **29**, with the equilibrium favoring the starting Ni complex **27**. Heating the mixture to 70 °C for 30 min led to significant decomposition, likely caused by protonolysis of the

Ni–aryl bond by *i*PrOD and/or residual water, as evidenced by the detection of deuterated toluene in GC-MS analysis.

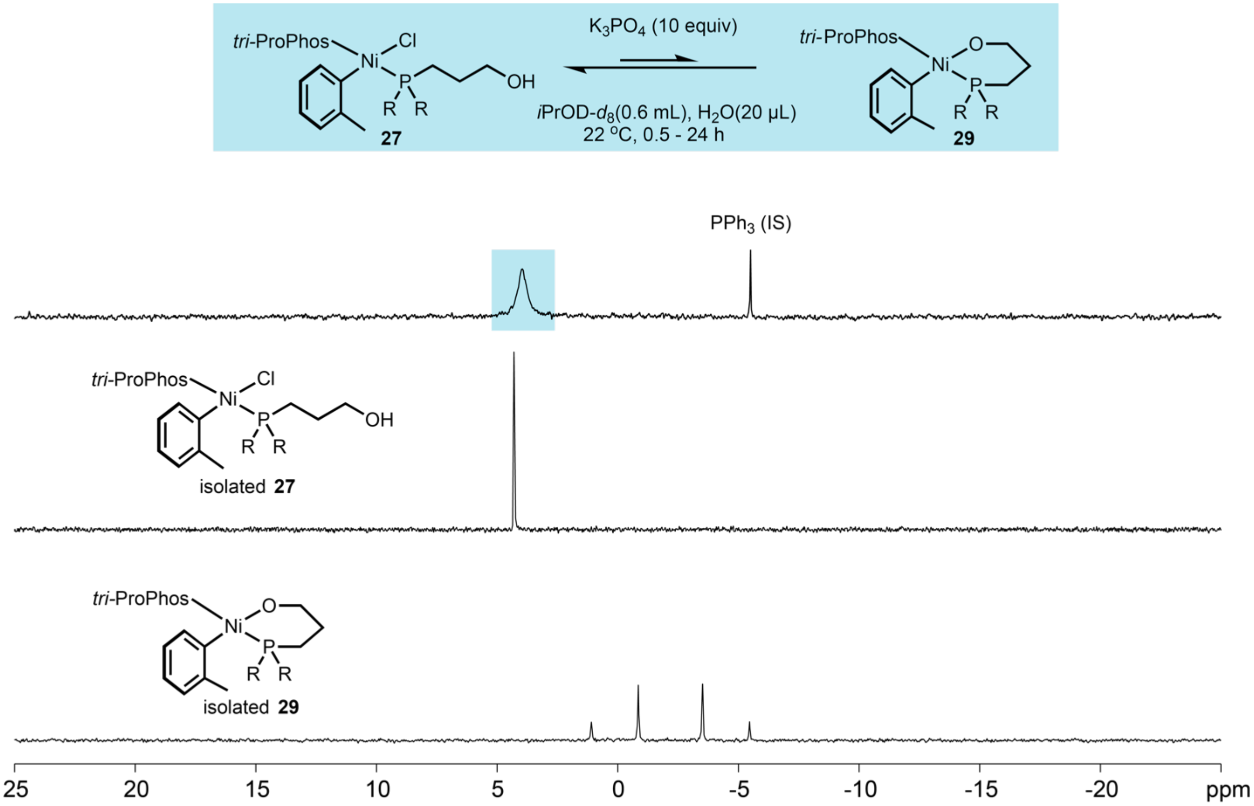

Figure S19. $^{31}P\{^1H\}$ NMR (162.04 MHz, *i*PrOD-d_8) spectra of: the reaction of Ni(*tri*-ProPhos)$_2$Cl(*o*-Tol) **27** and K_3PO_4 at room temperature (top); isolated Ni(*tri*-ProPhos)$_2$Cl(*o*-Tol) **27** (middle) and Ni metallacycle **29** (bottom). R = $(CH_2)_3OH$.

To support the existence of this equilibrium in the reaction mixture, we repeated the reaction in pre-dried *i*PrOD-d_8. Our rationale was that the reverse reaction likely requires both KCl and a proton source. Although *i*PrOD-d_8.can still serve as a weak proton source, the very low solubility of KCl in this solvent could significantly slow (or even suppress) the reverse process, allowing us to observe the formation of the Ni metallacycle intermediate more clearly by NMR. Indeed, under these pre-dried conditions, we detected slower formation of the Ni metallacycle (Figure S20, compared to that using KOH, *vide supra*), providing evidence that this equilibrium process does take place. However, because the reaction still occurred in a protic environment, we also observed decomposition of the Ni species over time, which we attribute to protonolysis of the Ni–aryl bond.

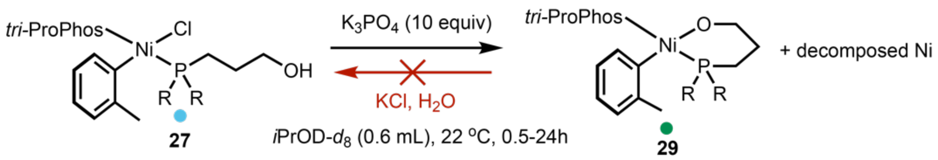

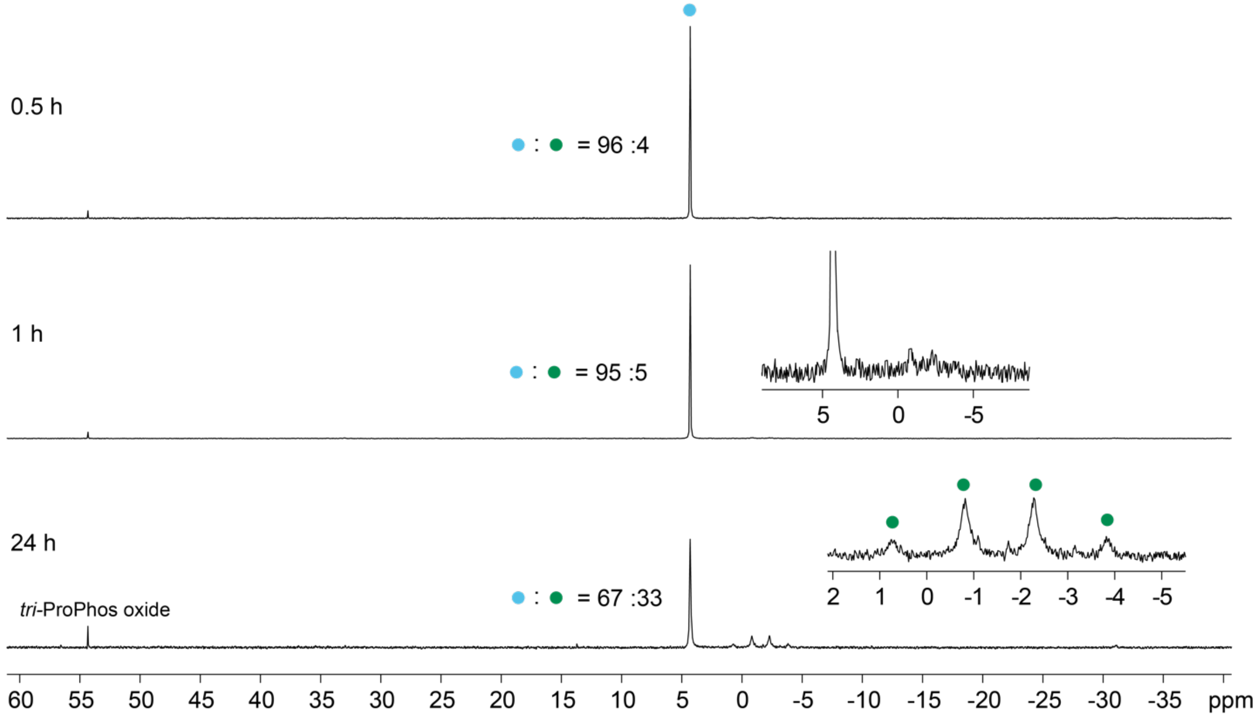

Figure S20. $^{31}P\{^1H\}$ NMR (202.47 MHz, *i*PrOD-*d*$_8$) spectra of the reaction of Ni(*tri*-ProPhos)$_2$Cl(*o*-Tol) **27** and K_3PO_4 at room temperature over time. The ratios of Ni(*tri*-ProPhos)$_2$Cl(*o*-Tol) **27** (•)to the Ni metallacycle **29** (•) are determined by their relative integrations. R = $(CH_2)_3OH$.

6.3 Substitutional chemistry of (*tri*-ProPhos)Ni complexes

NMR sample preparation followed by general procedure described in Section 6.2. The catalytic reactions for robustness test were followed by general procedure described in Section 3.

Coordination of pyridine to Ni(tri-ProPhos) complexes.

To explore the influence of heterocycle coordination on the Ni(*tri*-ProPhos) catalyst, the resting-state complex **27** was reacted with an excess of pyridine (Figure S21). Upon adding pyridine to the solution of the Ni complex in *i*PrOD-d_8 at 70 °C, we observed more complex decomposition (also seen in Section 6.2) instead of the pyridine coordination. Based on the internal standard, approximately 32% of of Ni(*tri*-ProPhos)$_2$Cl(*o*-Tol) **27** remained in solution after heating at 70 °C over 6 h (upon decomposition). The $^{31}P\{^{1}H\}$ signals observed at 13.7 and 13.1 ppm are tentatively assigned to the dimeric Ni metallacycle based on its analogue to our isolated (ProPhos)Ni metalacyclic dimer (that will be reported in due course). A minor signal at 33.4 ppm, which was also detected during the resting-state analysis, is attributed to a Ni(0) species. Additionally, a small signal at 24.5 ppm is tentatively assigned to the pyridine-ligated complex [Ni(*tri*-ProPhos)(py)$_2$(*o*-Tol)]Cl **32**, as its chemical shift closely matches that of the analogous complex [Ni(PPh_3)(py)$_2$(*o*-Tol)]PF_6 reported in the literature (25.4 ppm).[44] We proposed some potential decomposition pathways (Scheme S5). Therefore, to minimize degradation, most subsequent organometallic experiments involving Ni–aryl species in *i*PrOD were conducted at room temperature.

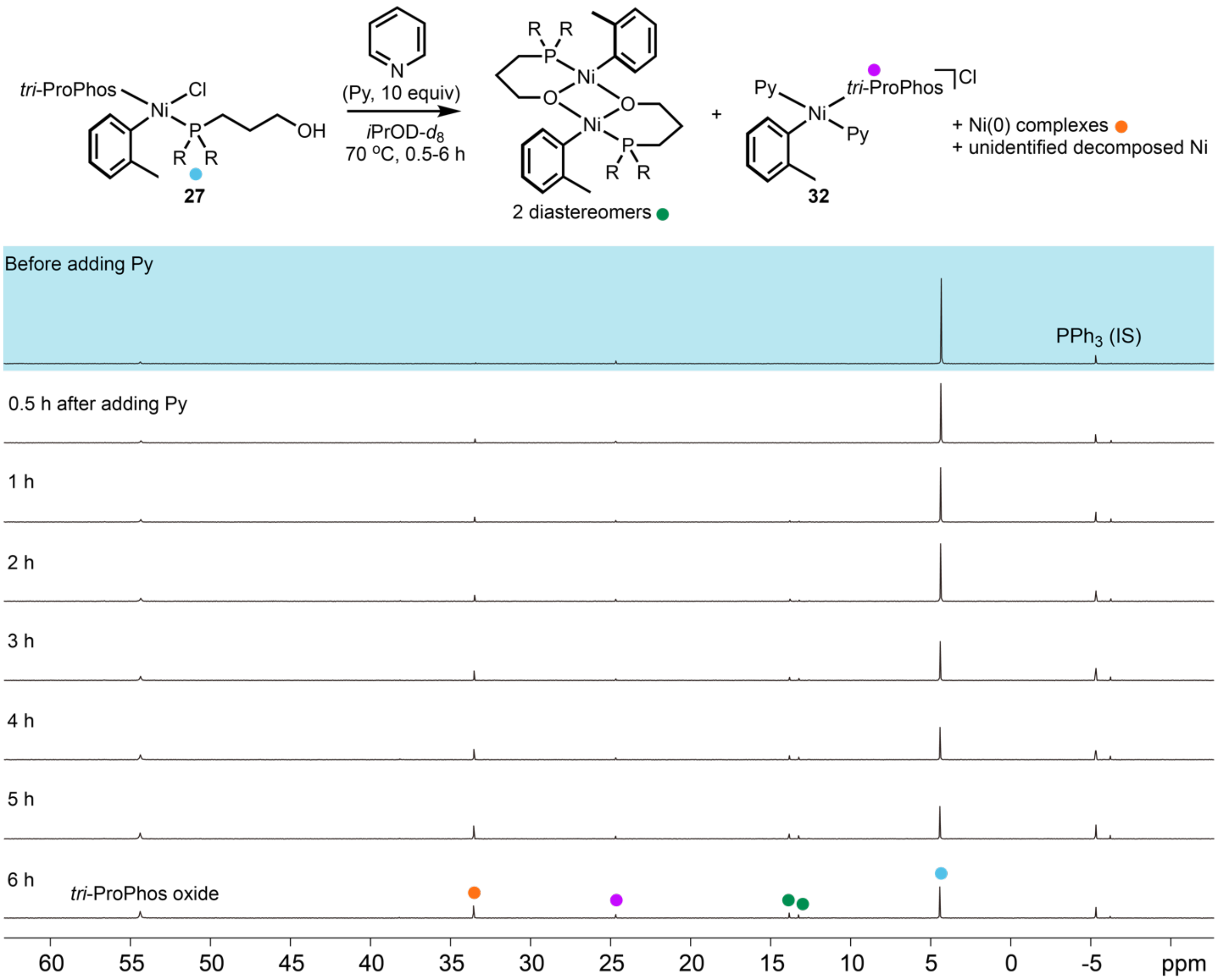

Figure S21 $^{31}P\{^{1}H\}$ NMR (202.47 MHz, *i*PrOD-d_8) spectra of the reaction of Ni(*tri*-ProPhos)$_2$Cl(*o*-Tol) **27** with pyridine (10 equiv) at 70 °C overtime. R = $(CH_2)_3OH$.

Scheme S5. Proposed Decomposition Pathway of Ni(*tri*-ProPhos)$_2$Cl(*o*-Tol) **27** Facilitated by Added Pyridine at High Temperature in *i*PrOD-d_8. R = $CH_2CH_2CH_2OH$.

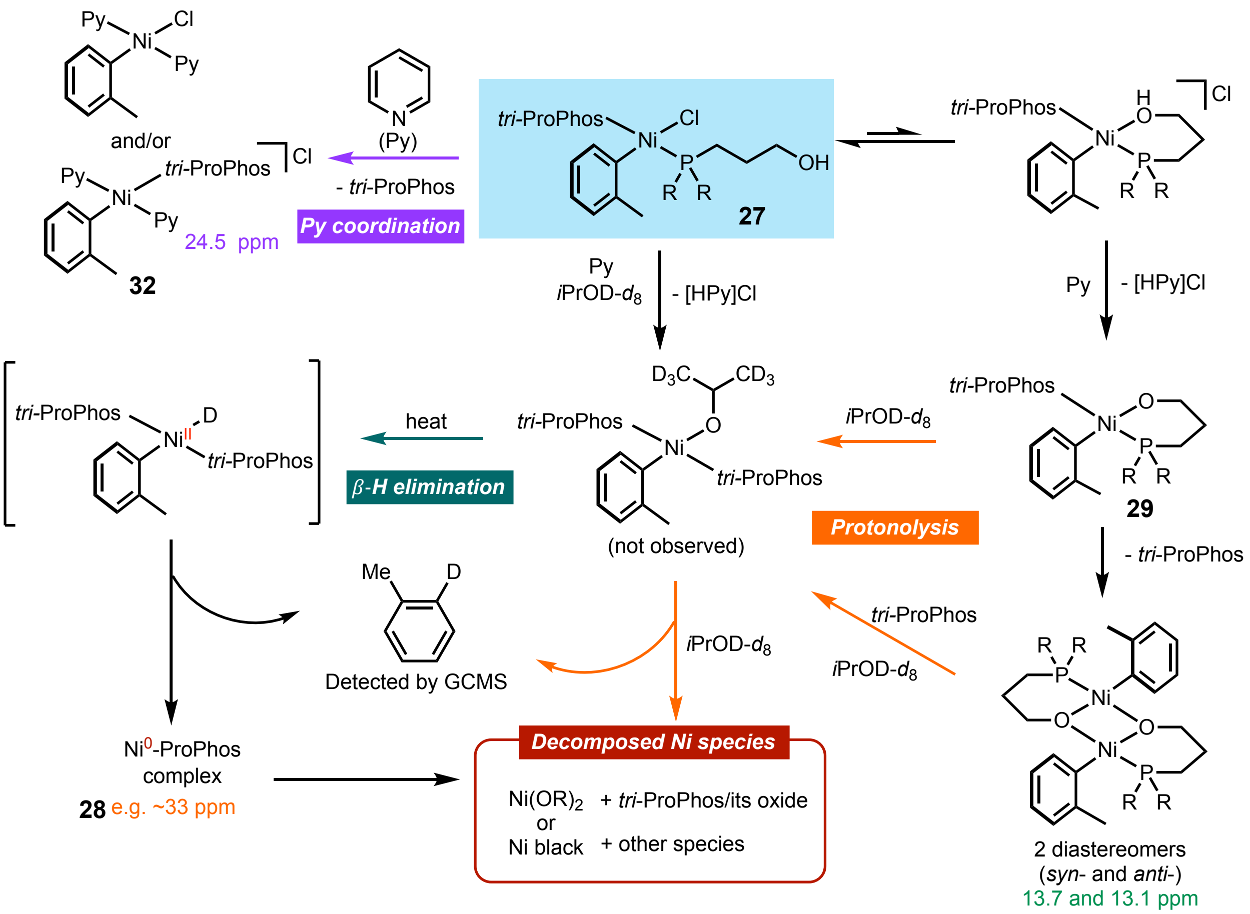

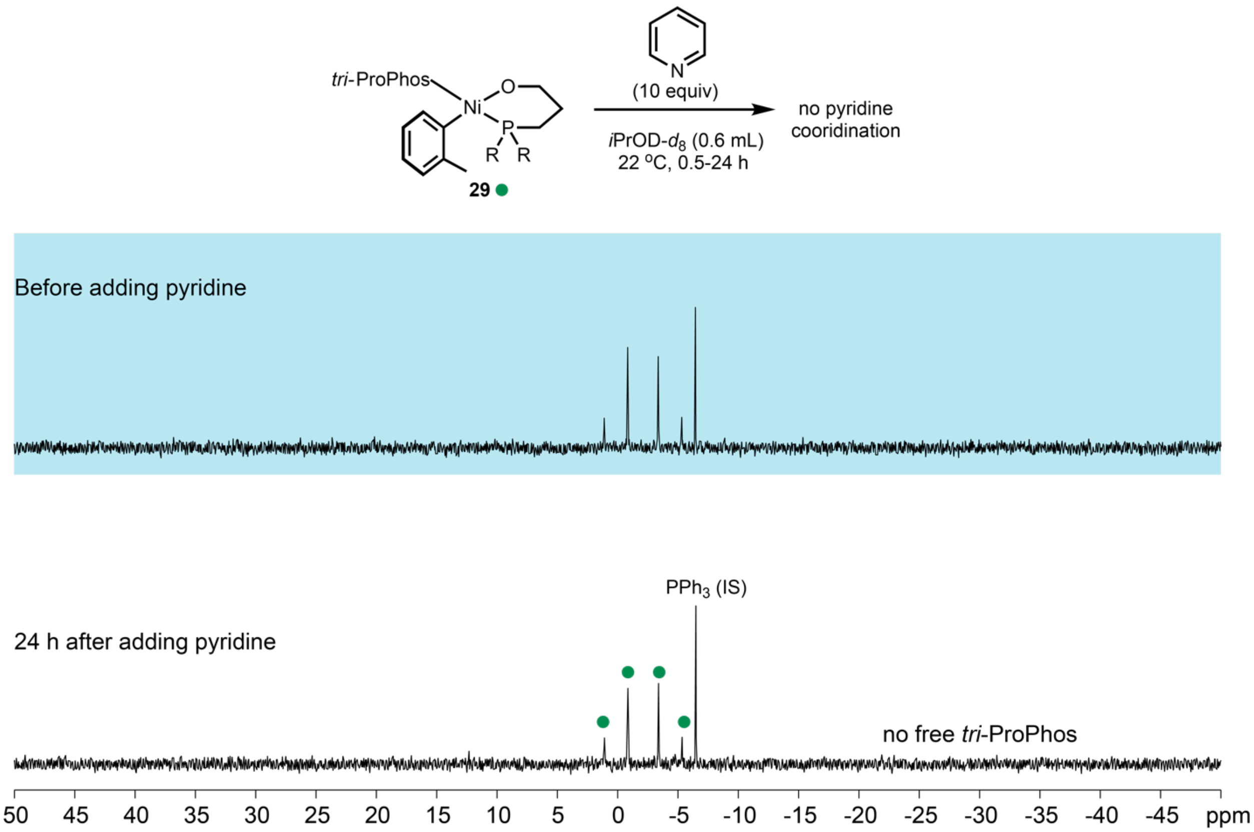

Figure S22 $^{31}P\{^1H\}$ NMR (162.04 MHz, *i*PrOD-d_8) spectra of the reaction of Ni(*tri*-ProPhos) metallacycle **29** with pyridine (10 equiv) at room temperature, showing no coordination of pyridine. R = $(CH_2)_3OH$.

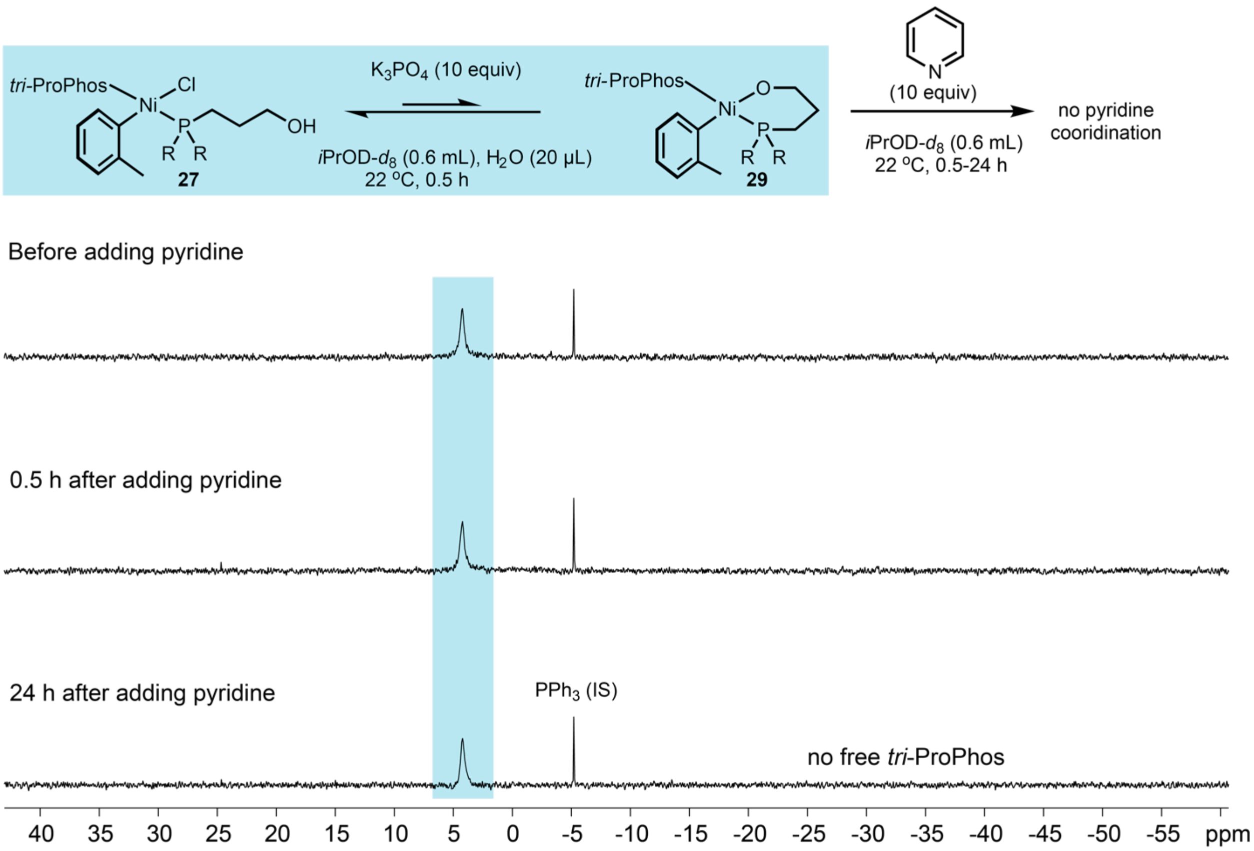

Figure S23 $^{31}P\{^{1}H\}$ NMR (162.04 MHz, *i*PrOD-*d*$_8$) spectra of the reaction of Ni(*tri*-ProPhos)$_2$Cl(*o*-Tol) **27** (pre-treated with 10 equiv of K_3PO_4) with pyridine (10 equiv) at room temperature, showing no coordination of pyridine. R = $(CH_2)_3OH$.

Robustness test of (tri-ProPhos)Ni catalysts and Pd(dppf)Cl_2

Since both Ni(*tri*-ProPhos)$_2$Cl(*o*-Tol) **27** and Ni(*tri*-ProPhos) metallacycle **29** were observed in our resting-state analysis, we believe both species are likely involved in the catalytic cycle. To further probe their relevance and test their robustness against heterocycle inhibition, we conducted a robustness test following Glorius' protocol[45], using model reaction 1 with pyridine as an additive (known to inhibit some Ni catalysts[8] and performed the reaction under air. The catalysts tested included Ni(*tri*-ProPhos)$_2$Cl(*o*-Tol) **27**, and Ni(*tri*-ProPhos) metallacycle **29** and Pd(dppf)Cl_2.

The results (Table S4) show:

1. Both Ni resting-state complexes are catalytically active.
2. The isolated precatalyst gave slightly lower yields than the in-situ generated Ni(*tri*-ProPhos) catalyst, possibly due to partial oxidation of the phosphine ligand when exposed to air.
3. The Ni(*tri*-ProPhos)-based systems outperformed Pd(dppf)Cl_2 under these conditions.
4. The presence of pyridine had little impact on the Ni(*tri*-ProPhos) catalysts but appeared to slightly inhibit the Pd-catalyzed reaction.

Table S4. Robustness Test Using Model Reaction 1.

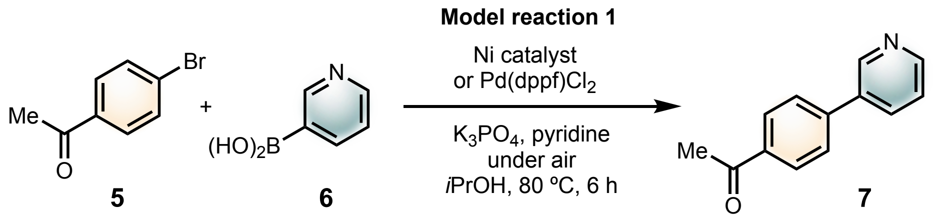

Entry	Catalyst (mol%)	Ligand (mol%)	%Yield of 7
**1**	$NiCl_2$•$6H_2O$ (2)	*tri*-ProPhos (8)	95
**2**	Ni(*tri*-ProPhos)$_2$Cl(*o*-Tol) **27** (2)	n/a	89
**3**	Pd(dppf)Cl_2 (2)	n/a	84
**4**	Ni metallacycle **29** (2)	n/a	92

Condition: [ArBr] = 0.50 M (**5**, 0.20 mmol, 1 equiv), [B(3-Py)$(OH)_2$] = 0.75 M (**6**, 1.5 equiv), K_3PO_4 = 106 mg (2.5 equiv), [pyridine] = 0.50 M (1 equiv). %Yield determined by GC-FID.

Competitive coordination of between ProPhos and tri-ProPhos

One possible reason *tri*-ProPhos outperforms ProPhos at very low catalyst loadings in model reaction 1 (Figure 2) is its stronger σ-donating ability, which may help stabilize the Ni catalyst and prevent deactivation by heterocycle coordination. To test this hypothesis, we carried out a series of ligand substitution experiments. Ni(ProPhos)$_2$Cl(*o*-Tol) **S1** (1.0 equiv) or Ni(*tri*-ProPhos)$_2$Cl(*o*-Tol) **27** (1.0 equiv) was weighed into a 4 mL vial containing a stir bar. ProPhos (2 equiv) or *tri*-ProPhos (2.4 equiv) was then added to the vial. *i*PrOD-d_8 (0.8 mL) was added to the vial and transferred to an NMR tube. Monitoring was performed using ^{31}P{^{1}H} NMR spectroscopy.

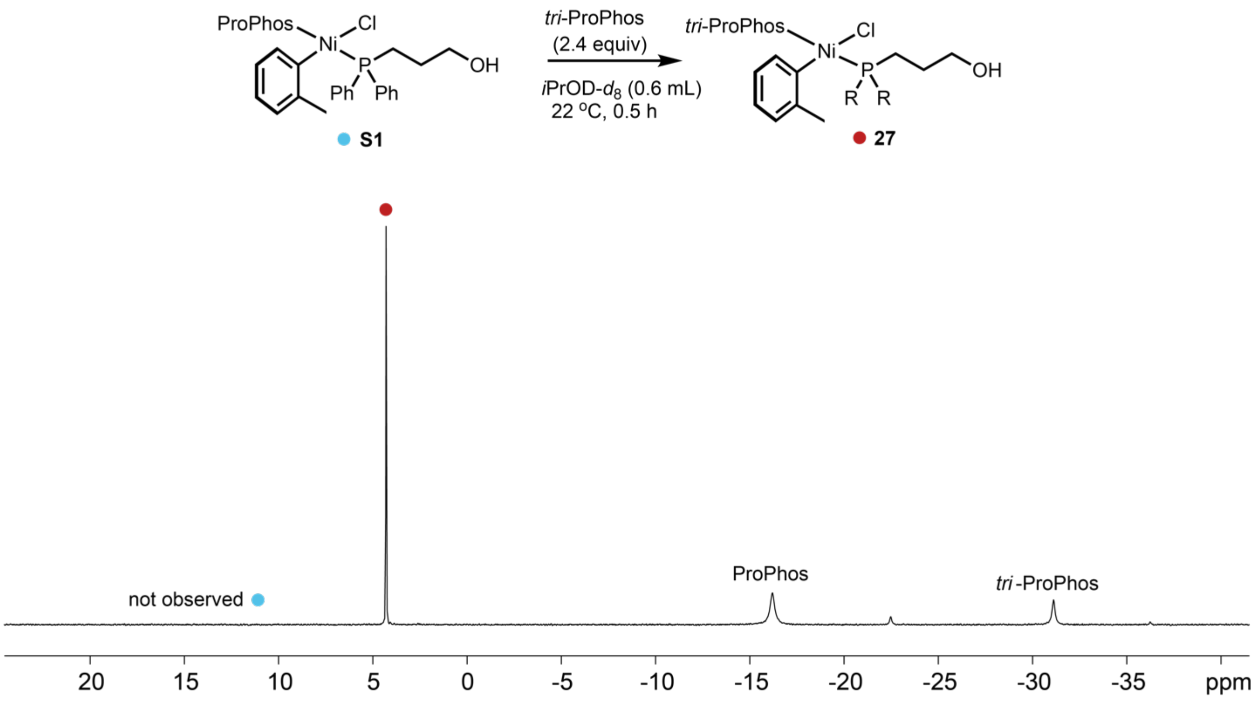

Figure S24. ^{31}P{^{1}H} NMR (202.47 MHz, *i*PrOD-d_8) spectrum of reaction of Ni(ProPhos)$_2$Cl(*o*-Tol) **S1** with *tri*-ProPhos at room temperature. R = (CH$_2$)$_3$OH.

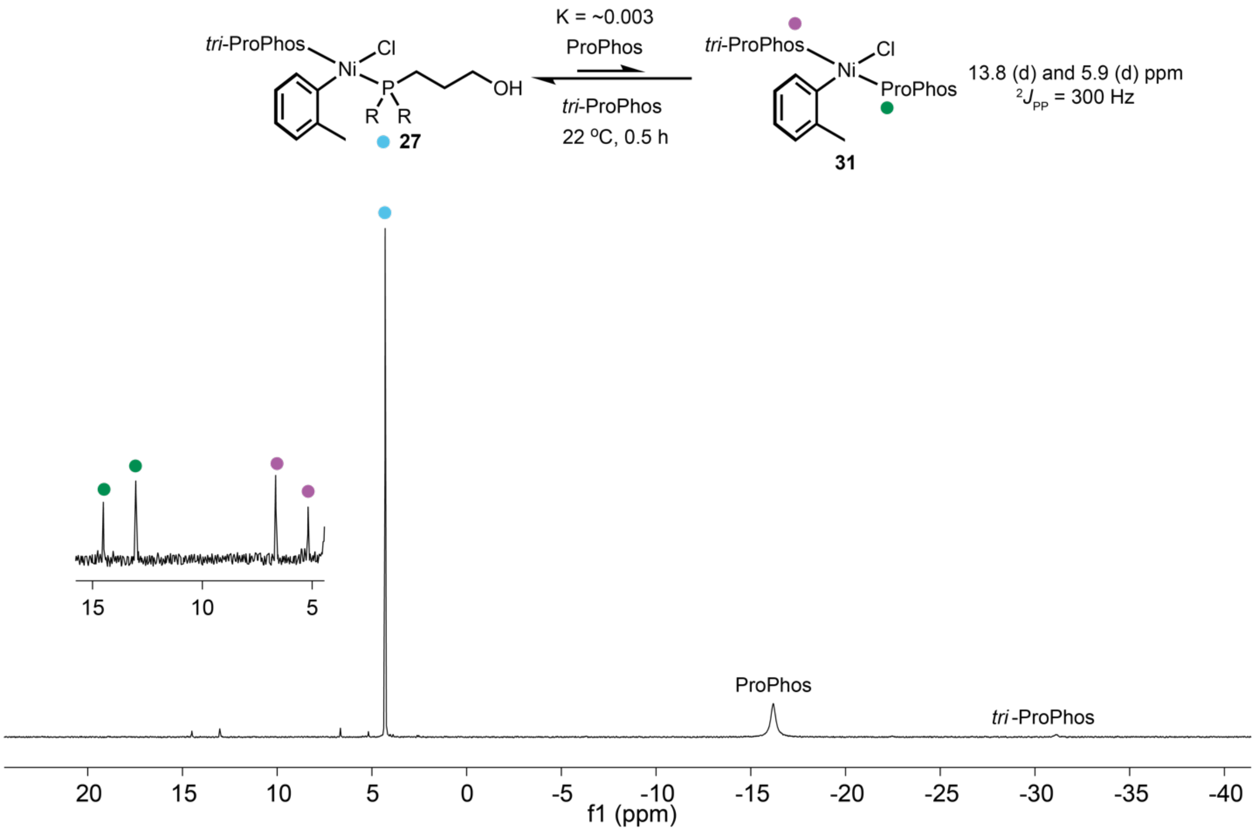

Figure S25 $^{31}P\{^1H\}$ NMR (202.47 MHz, *i*PrOD-d_8) spectrum of reaction of Ni(*tri*-ProPhos)$_2$Cl(*o*-Tol) **27** with ProPhos at room temperature. R = $(CH_2)_3OH$.

7. NMR Spectra

NMR spectra of phosphine ligands

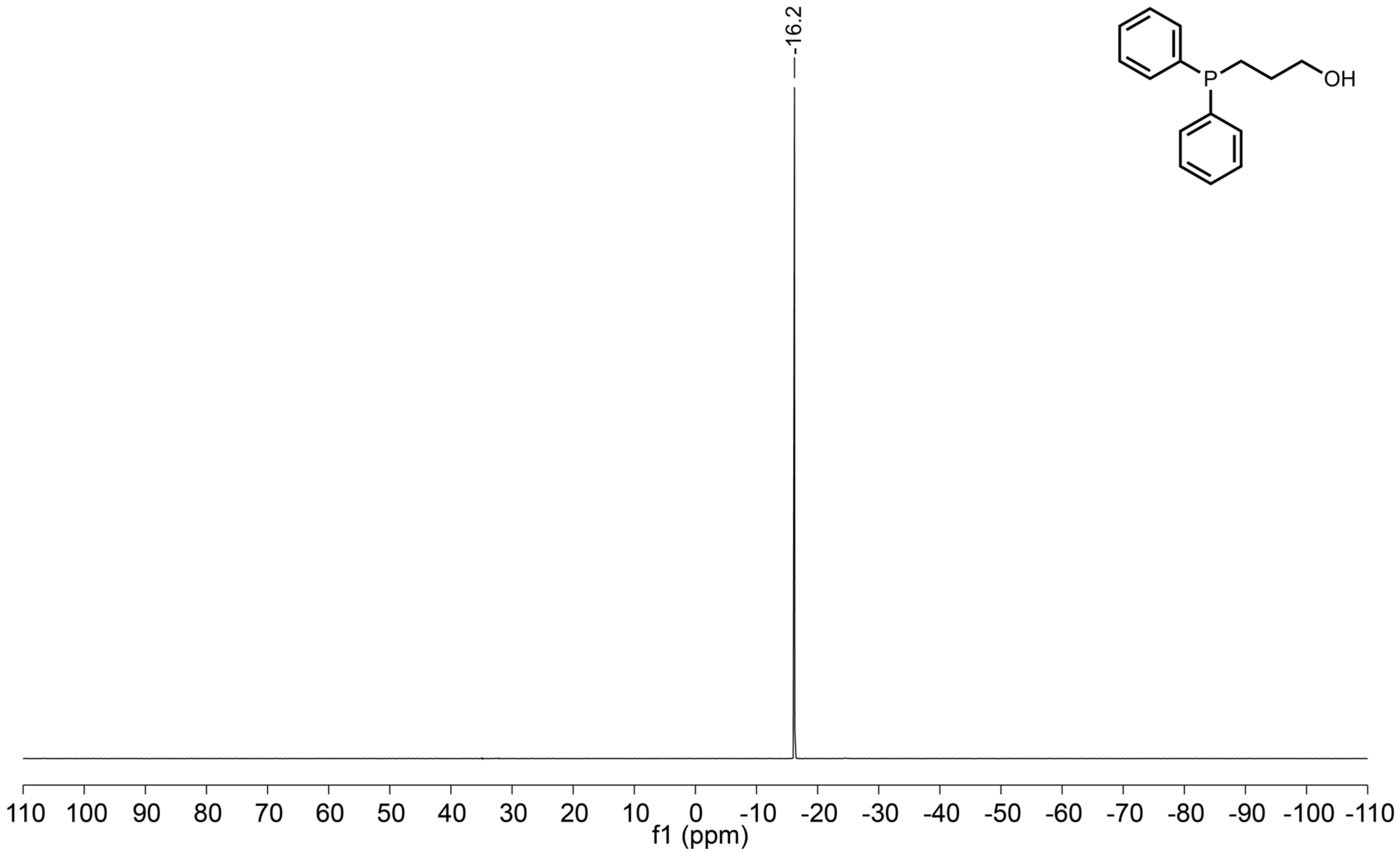

Figure S26. $^{31}P\{^{1}H\}$ NMR (202.47 MHz, $CDCl_3$) spectrum of $PPh_2CH_2CH_2CH_2OH$ (ProPhos).

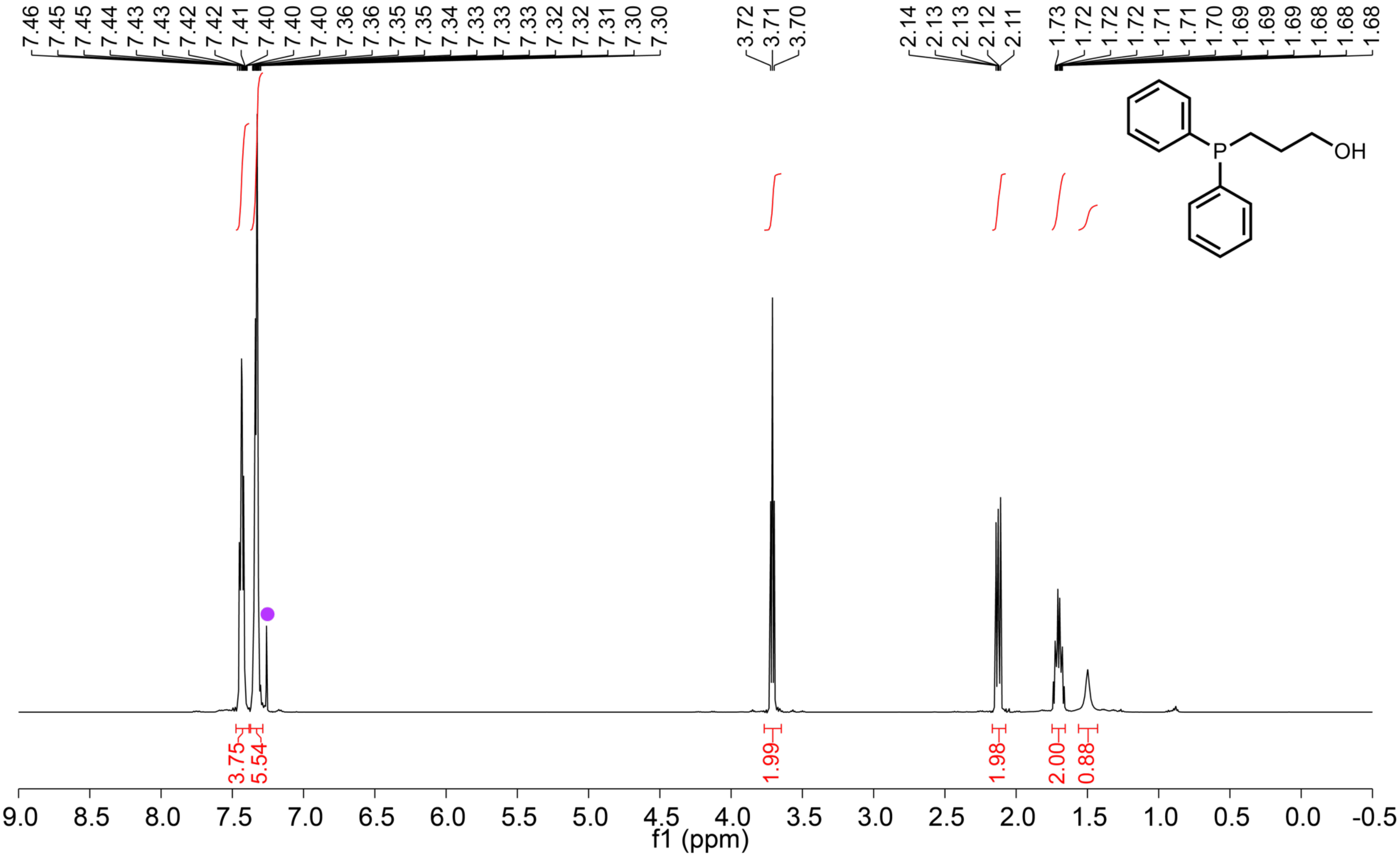

Figure S27. ^{1}H NMR (500.20 MHz, $CDCl_3$) spectrum of $PPh_2CH_2CH_2CH_2OH$ (ProPhos). Residual proteo-solvent (•).

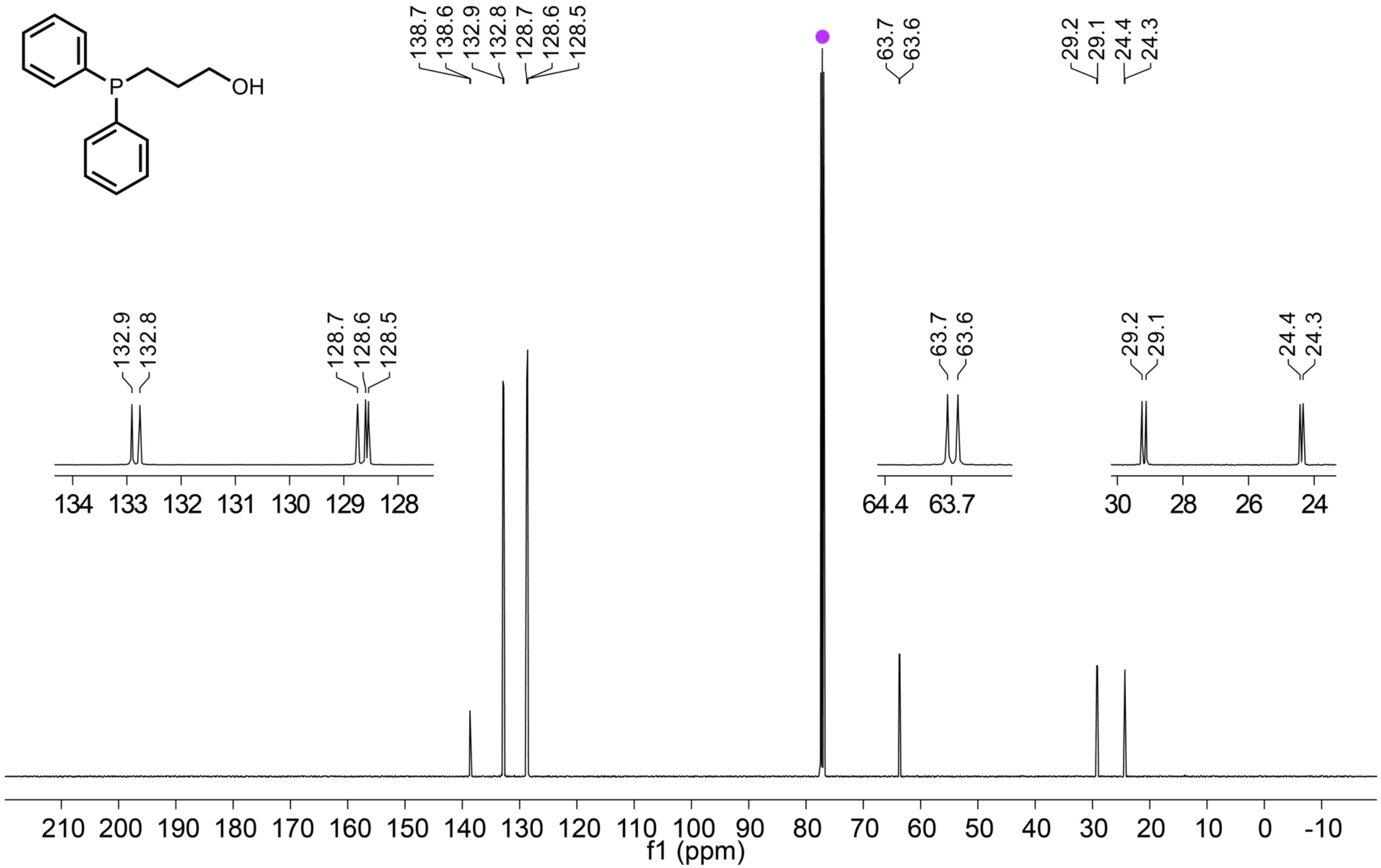

Figure S28. $^{13}C\{^1H\}$ NMR (125.79 MHz, $CDCl_3$) spectrum of $PPh_2CH_2CH_2CH_2OH$ (ProPhos). Deuterated solvent (•).

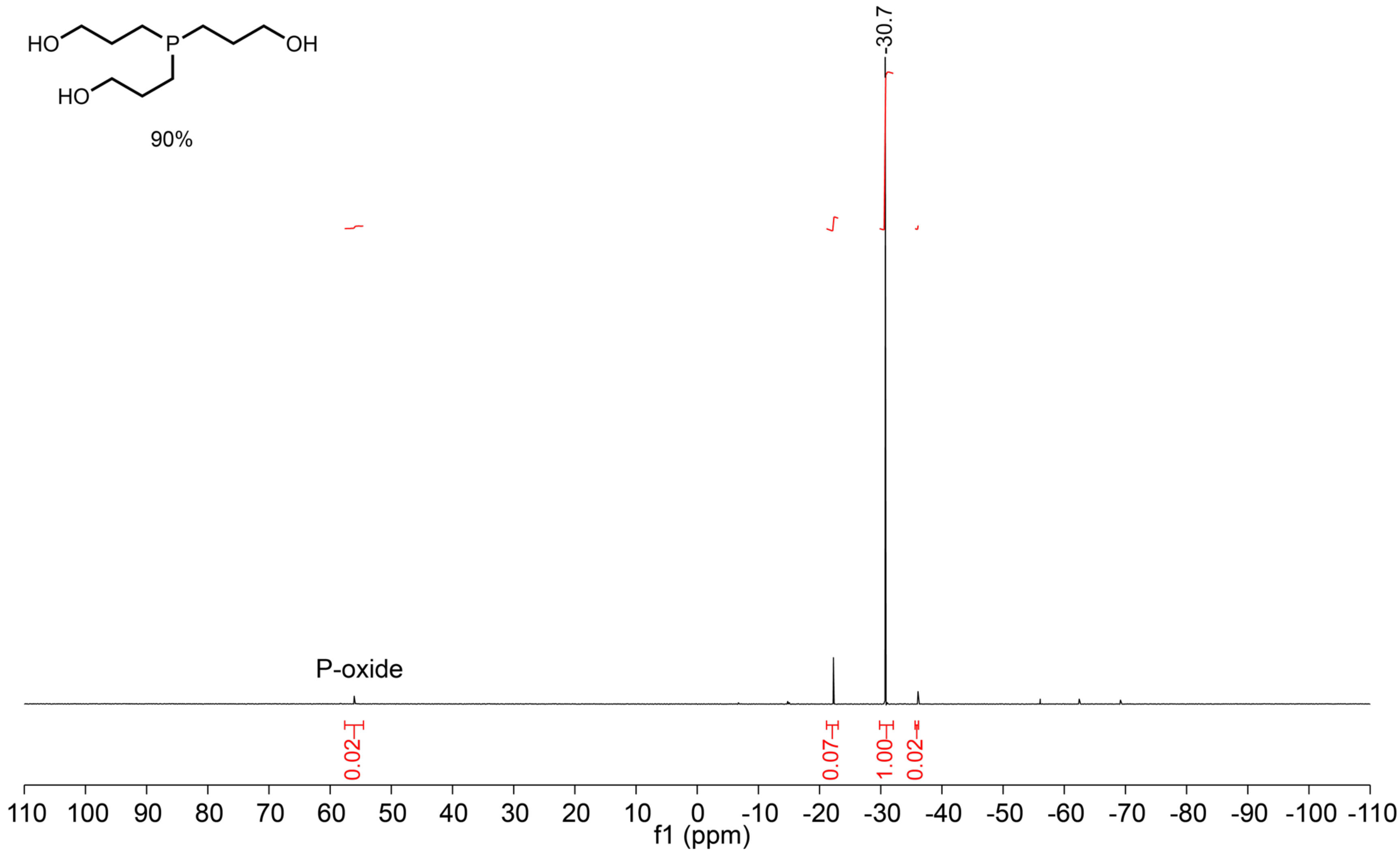

Figure S29. $^{31}P\{^{1}H\}$ NMR (202.47 MHz, CD_3OD) spectrum of commercially available $P(CH_2CH_2CH_2OH)_3$ (*tri*-ProPhos) with unidentified impurities. The purity of the phosphine is determined to be around 90%.

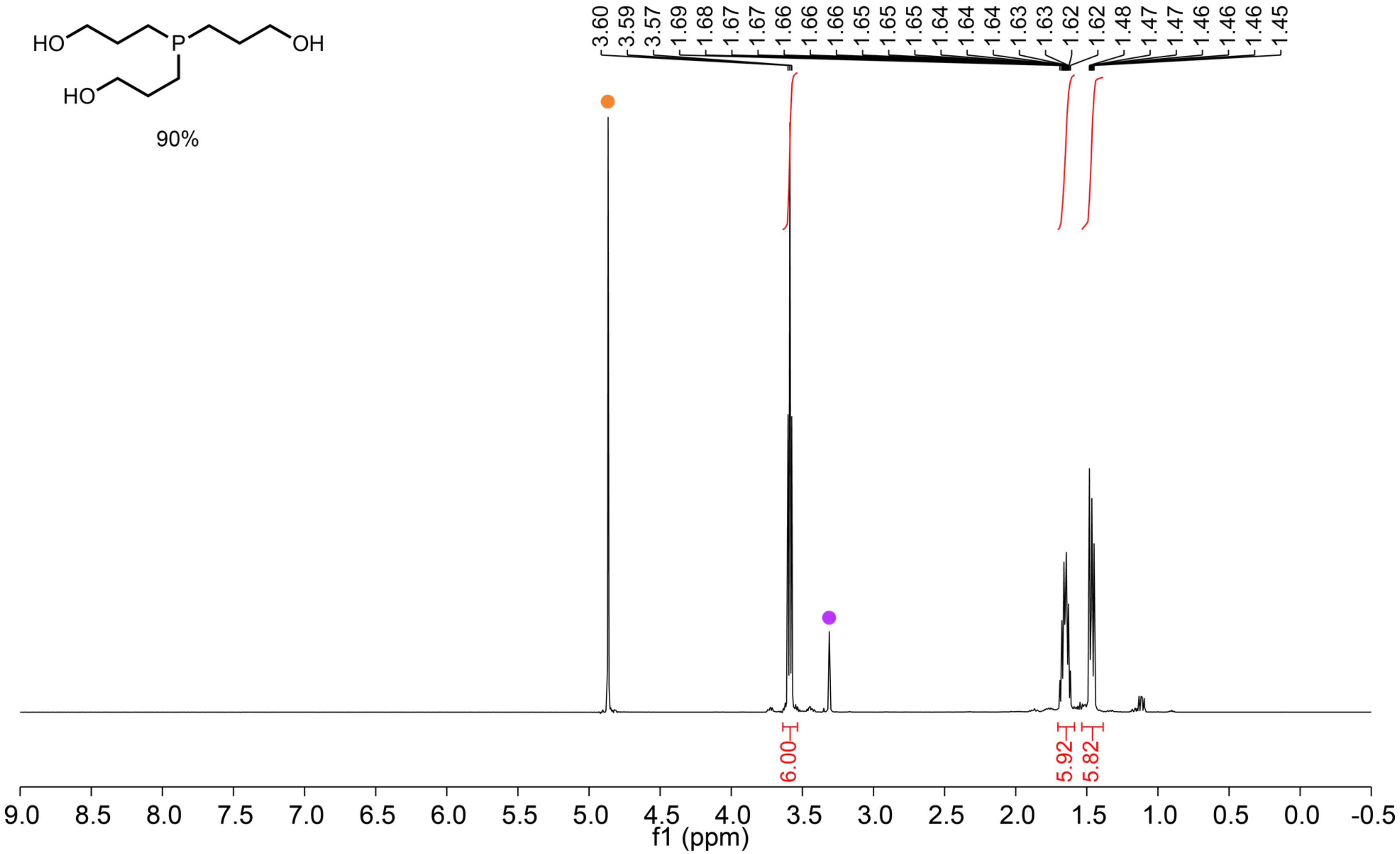

Figure S30. ^{1}H NMR (500.20 MHz, CD_3OD) spectrum of commercially available $P(CH_2CH_2CH_2OH)_3$ (*tri*-ProPhos) with unidentified impurities. H_2O (●) and residual proteo-solvent (●).

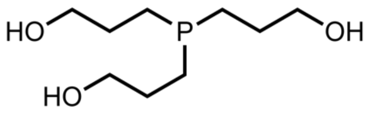

90%

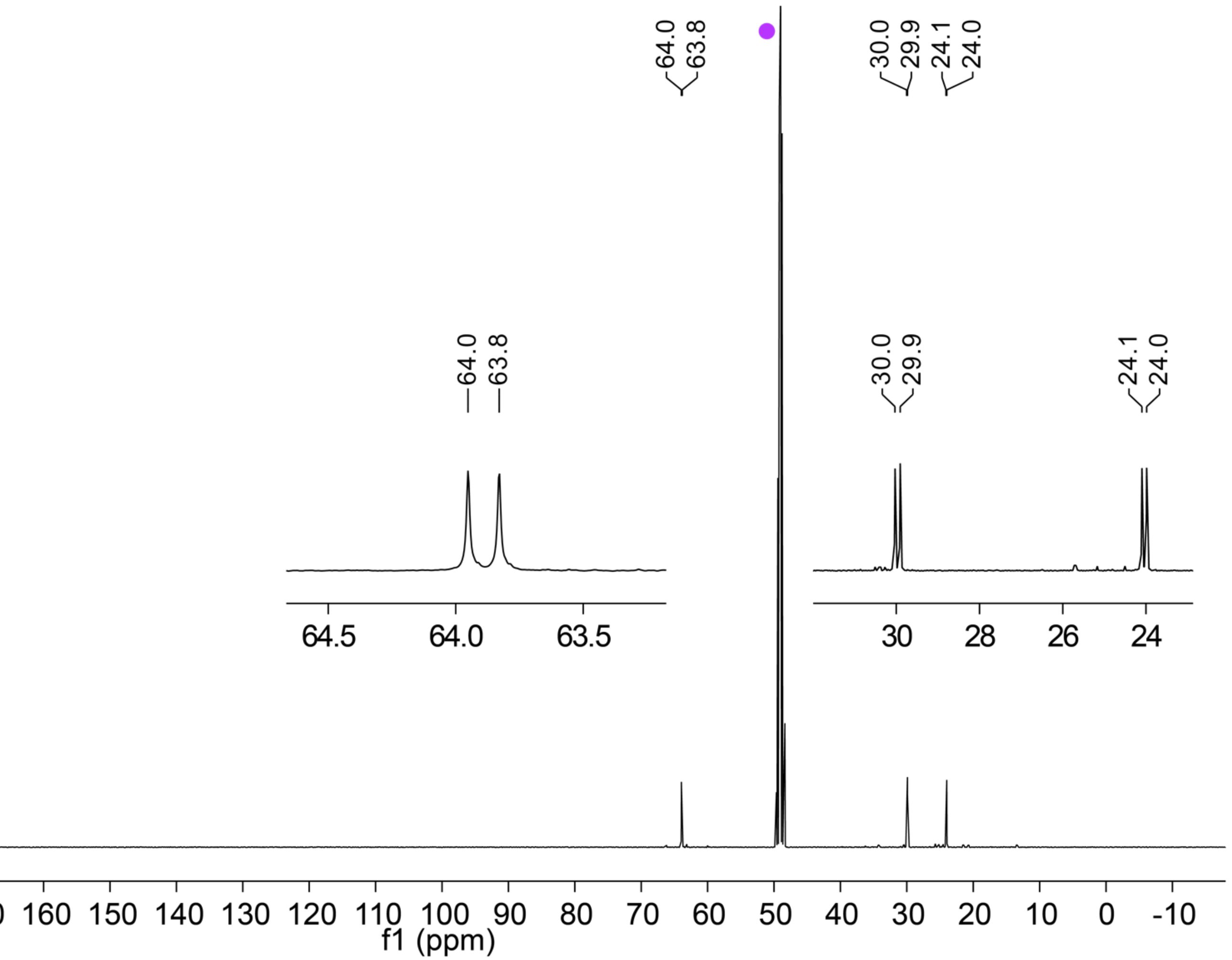

Figure S31 $^{13}C\{^1H\}$ NMR (100.67 MHz, CD_3OD) spectrum of commercially available $P(CH_2CH_2CH_2OH)_3$ (*tri*-ProPhos) with unidentified impurities. Deuterated solvent (●).

NMR spectra of organometallic compounds

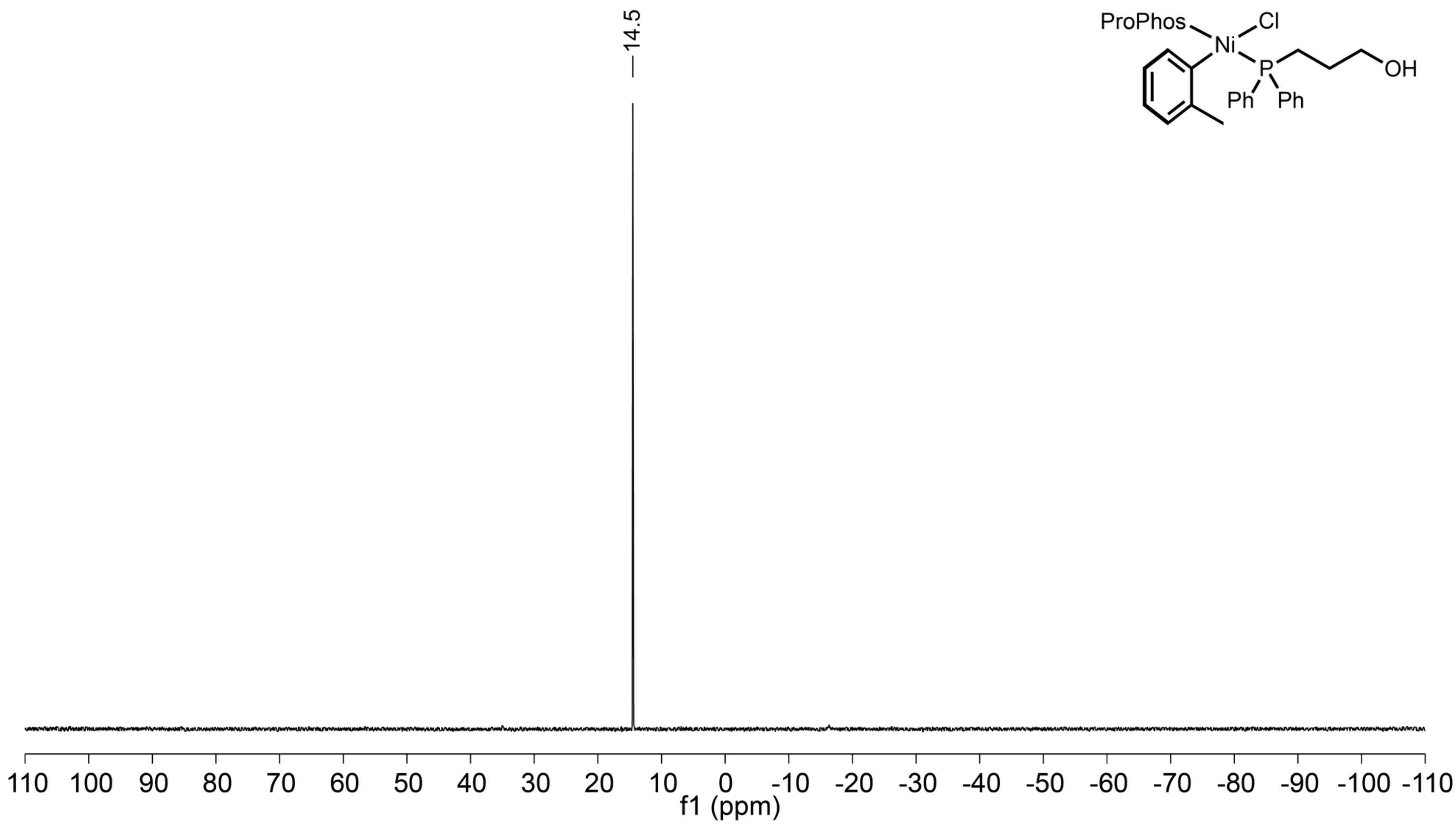

Figure S32. $^{31}P\{^1H\}$ NMR (202.47 MHz, *i*PrOD-d_8) spectrum of Ni(ProPhos)$_2$Cl(*o*-Tol) **S1**.

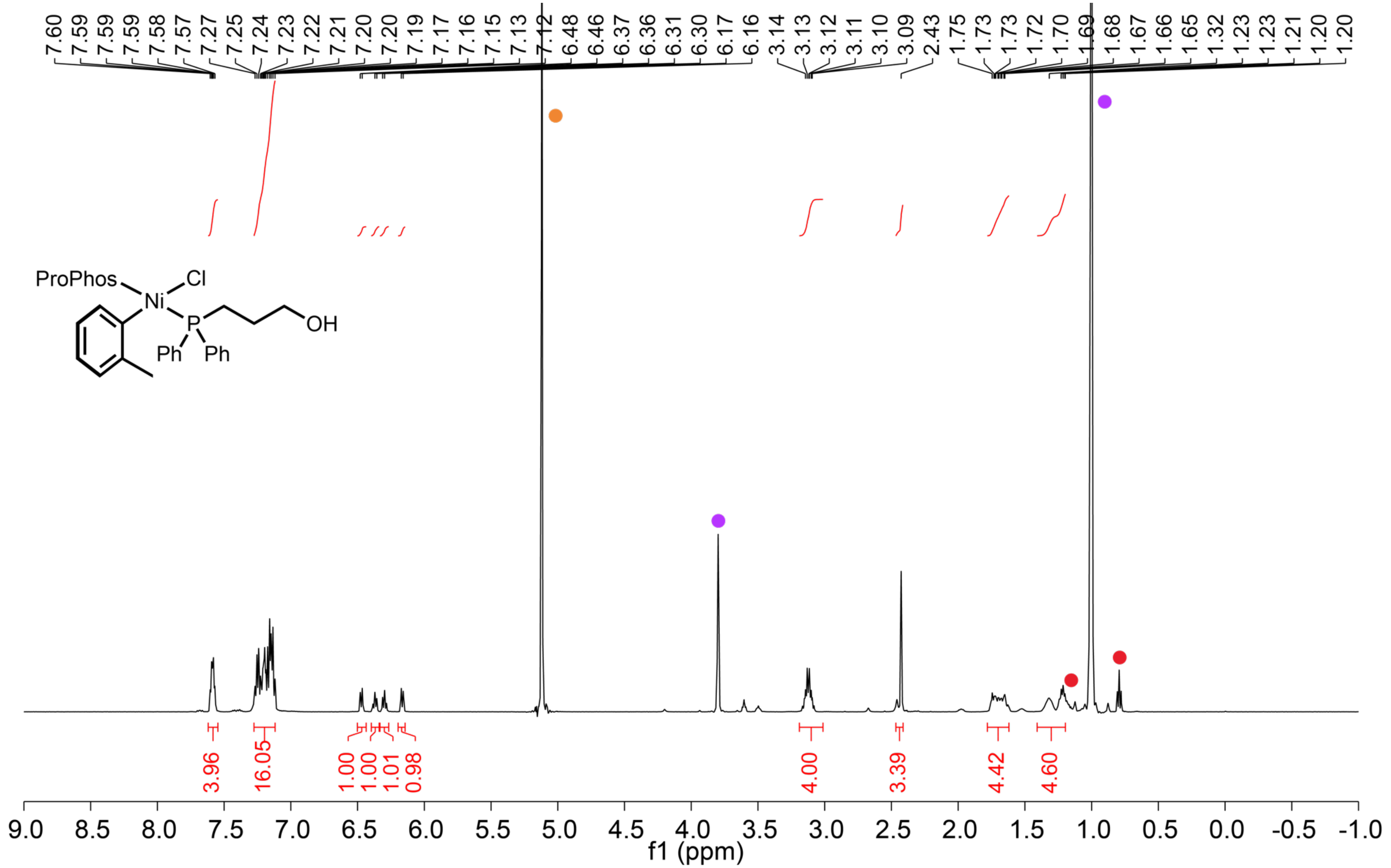

Figure S33 ^{1}H NMR (500.20 MHz, *i*PrOD-d_8) spectrum of Ni(ProPhos)$_2$Cl(*o*-Tol) **S1**. H_2O (●), residual proteo-solvent (●) and pentane (●).

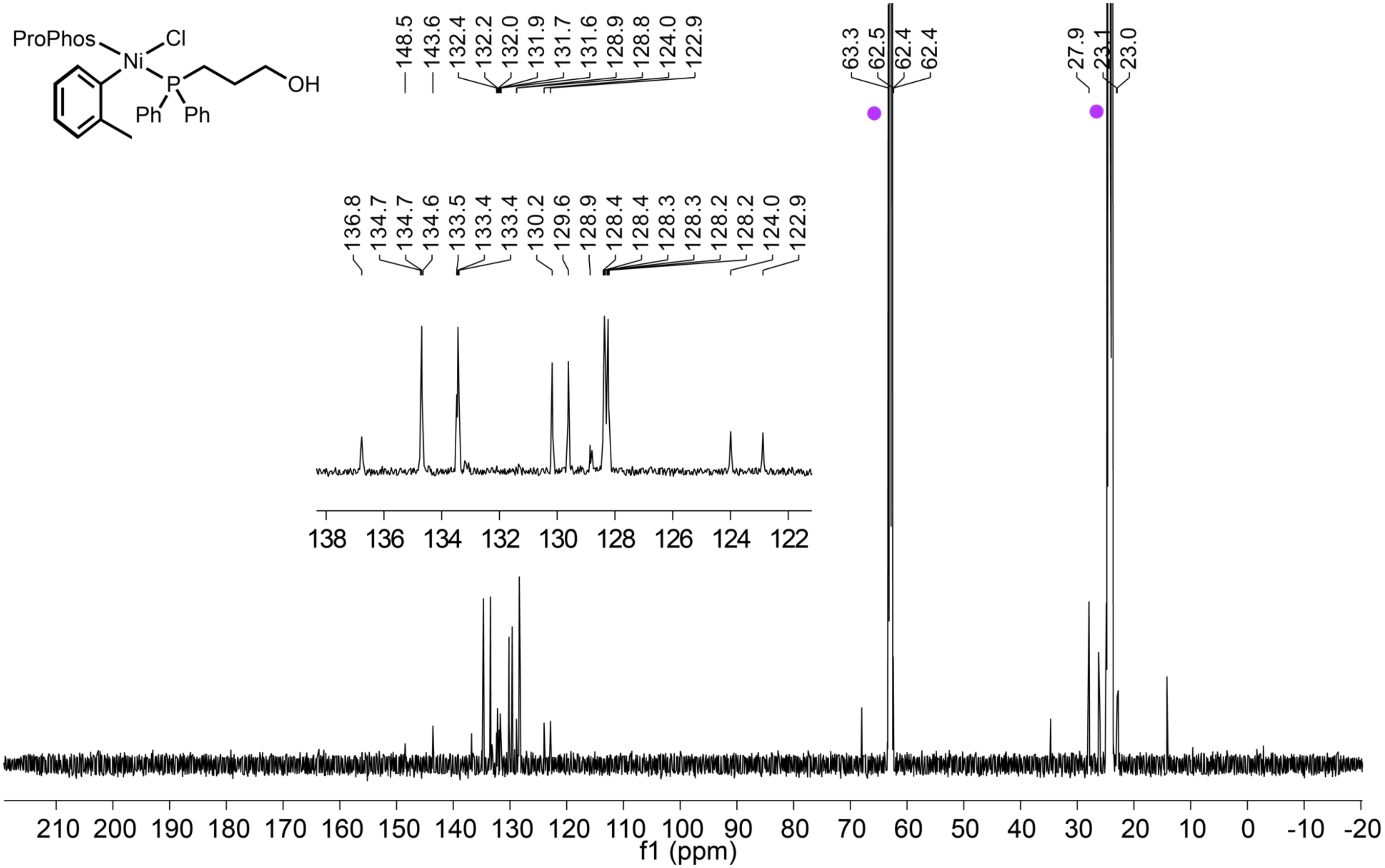

Figure S34. $^{13}C\{^{1}H\}$ (125.79 MHz, *i*PrOD-d_8) and $^{13}C\{^{1}H\}$ DEPT-135 (inset) NMR spectra of Ni(ProPhos)$_2$Cl(*o*-Tol) **S1**. Deuterated solvent (•).

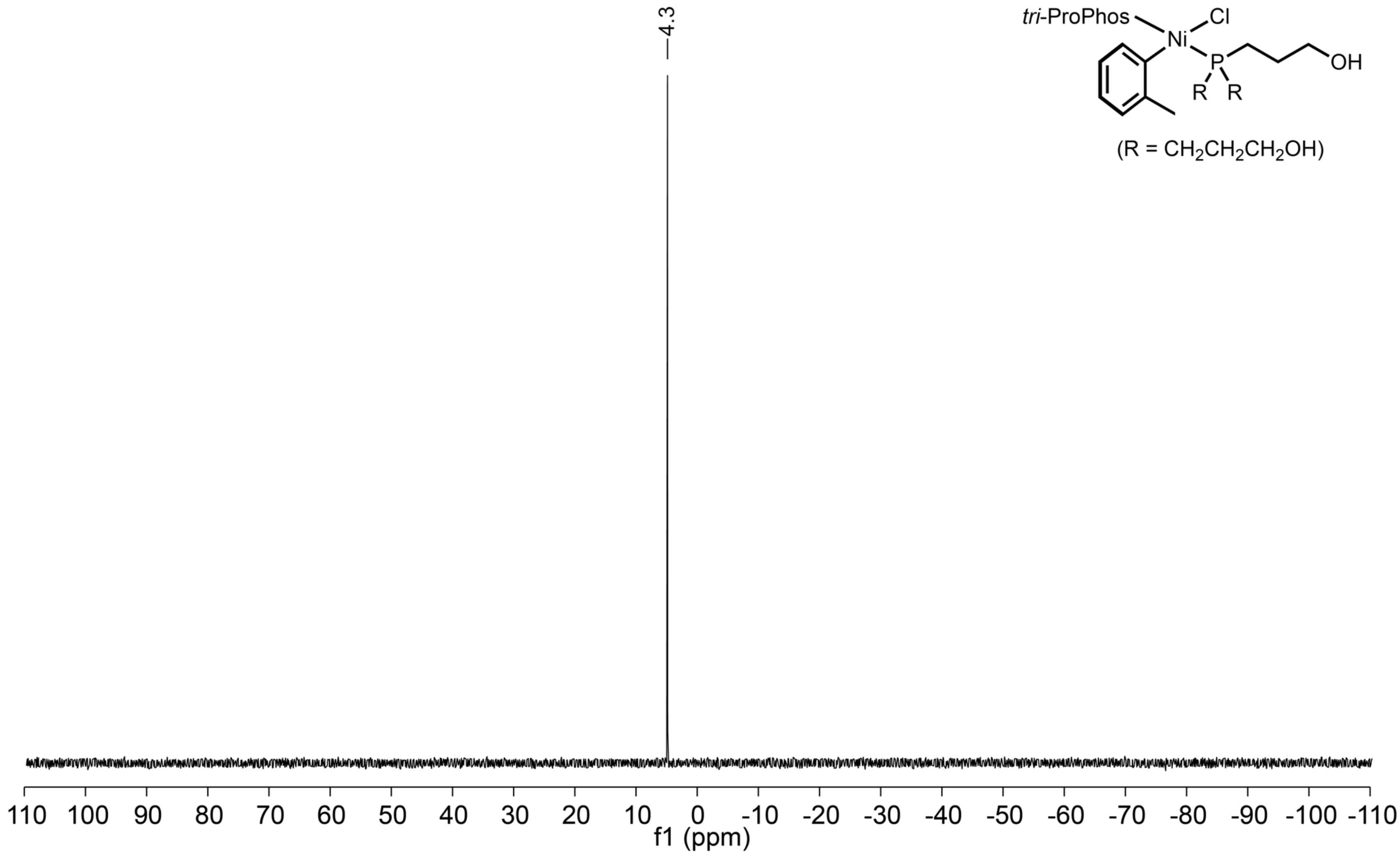

Figure S35. $^{31}P\{^{1}H\}$ NMR (202.47 MHz, *i*PrOD-d_8) spectrum of Ni(*tri*-ProPhos)$_2$Cl(*o*-Tol) **27**.

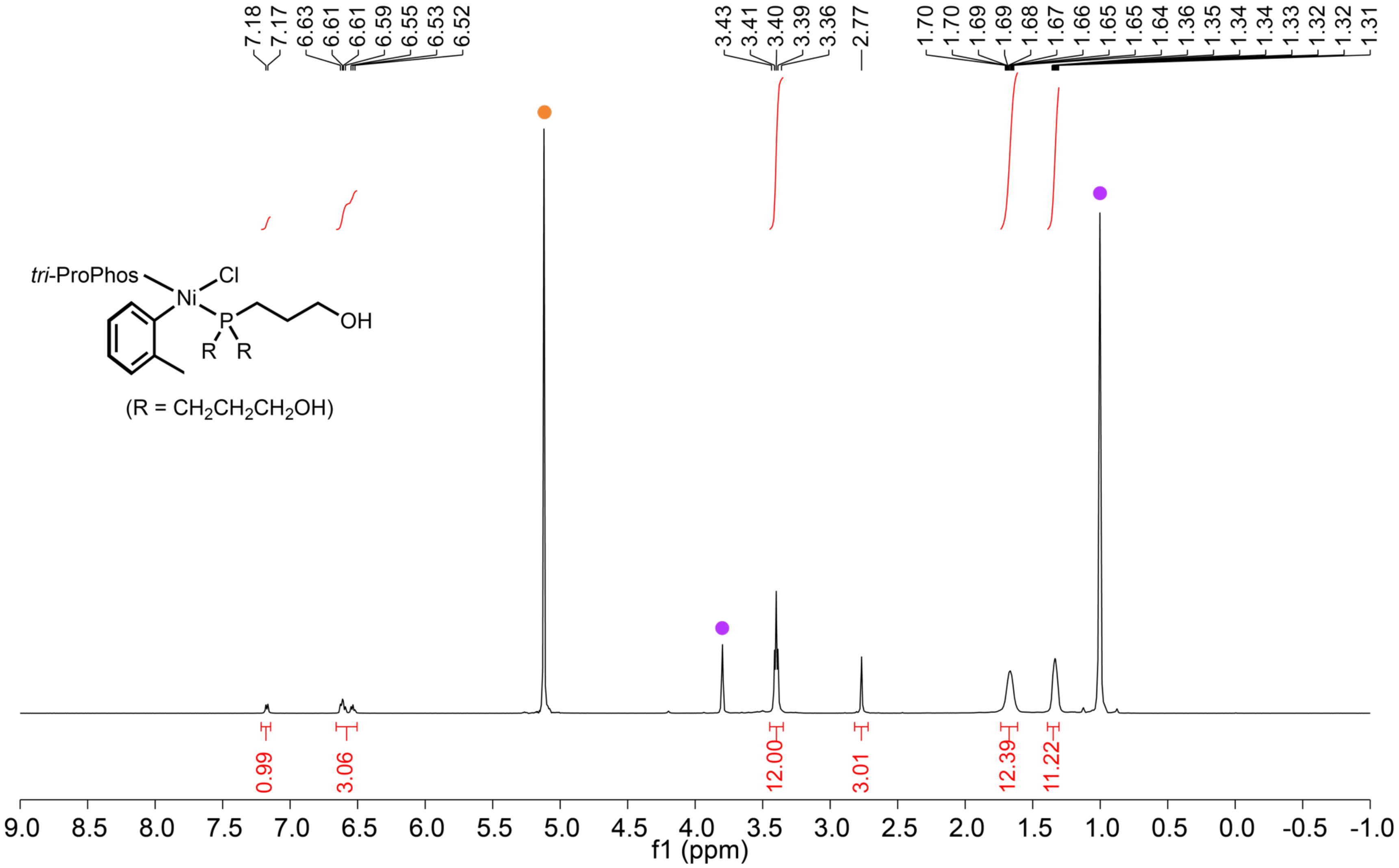

Figure S36. ^{1}H NMR (500.20 MHz, *i*PrOD-d_8) spectrum of Ni(*tri*-ProPhos)$_2$Cl(*o*-Tol) **27**. H_2O (●) and residual proteo-solvent (●).

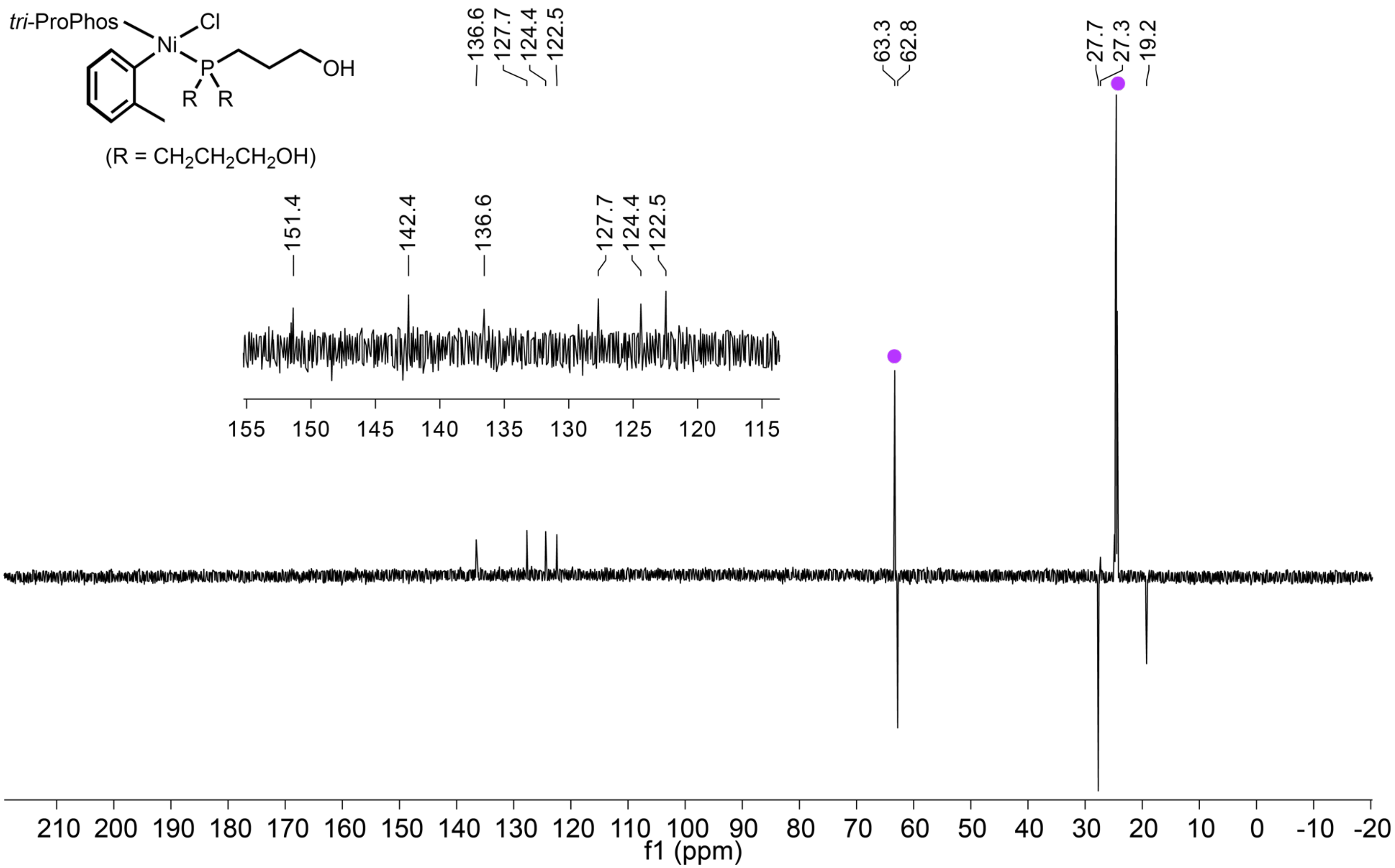

Figure S37. $^{13}C\{^1H\}$ DEPT-135 (125.79 MHz, *i*PrOD-d_8) and $^{13}C\{^1H\}$ NMR (inset) spectra of Ni(*tri*-ProPhos)$_2$Cl(*o*-Tol) **27**. Deuterated solvent (•).

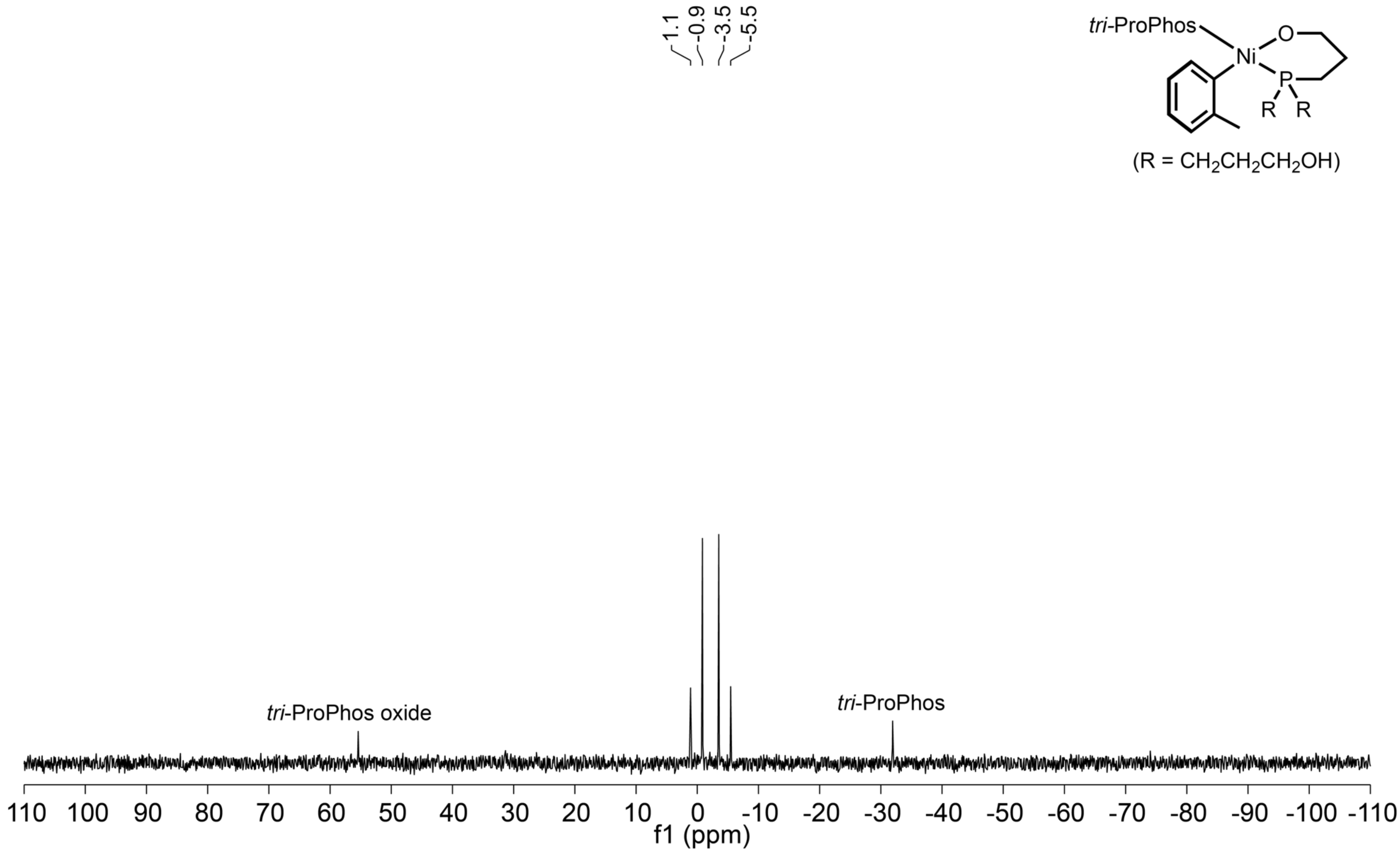

Figure S38. $^{31}P\{^{1}H\}$ NMR (162.04 MHz, *i*PrOD-d_8) spectrum of Ni(*tri*-ProPhos) metallacycle **29**, which would slowly decompose in *i*PrOH and H_2O. The oily, paste-like **29** makes it challenging to separate *tri*-ProPhos and its oxide from itself.

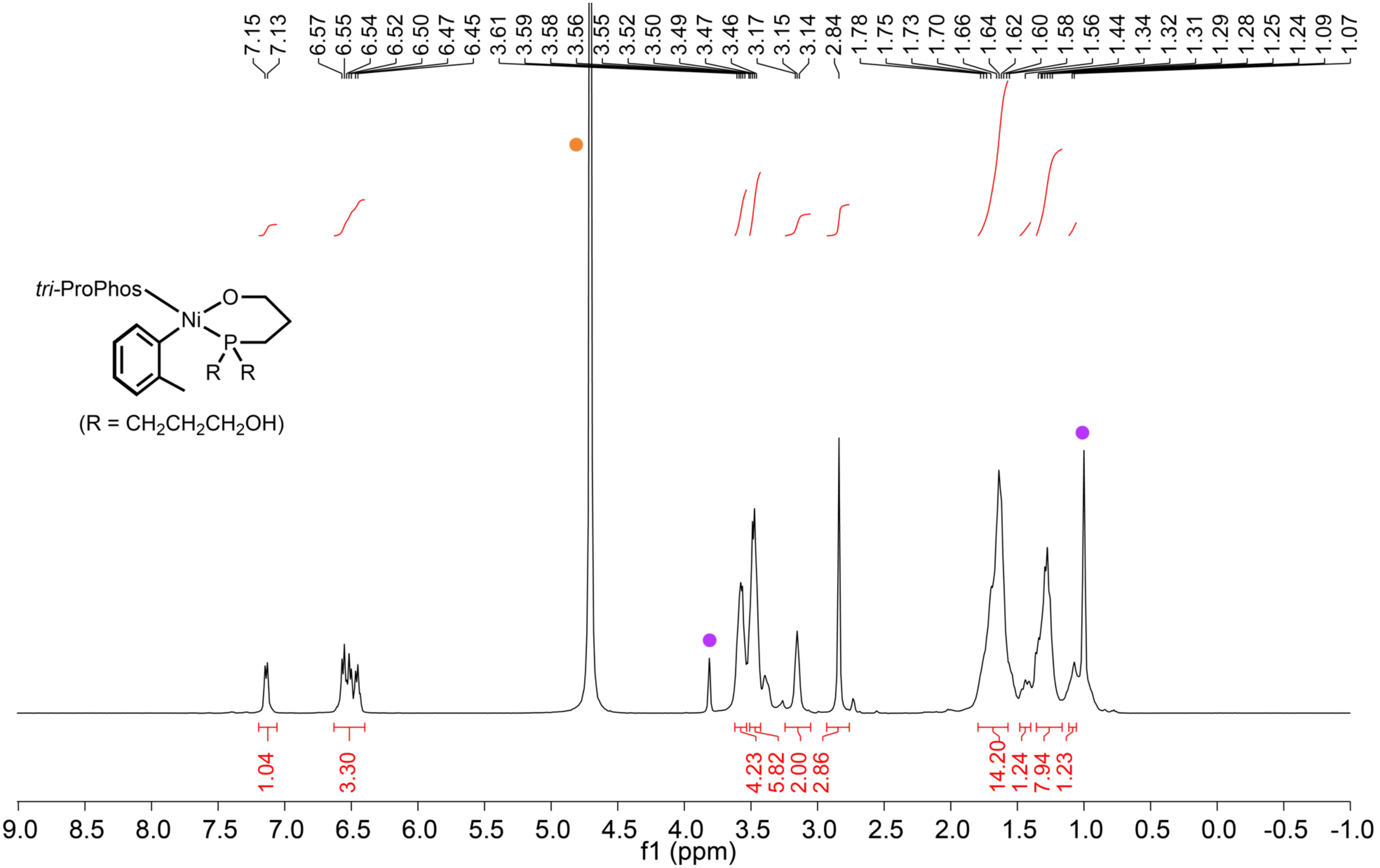

Figure S39. ^{1}H NMR (400.30 MHz, *i*PrOD-d_8) spectrum of Ni(*tri*-ProPhos) metallacycle **29**. H_2O (●) and residual proteo-solvent (●).

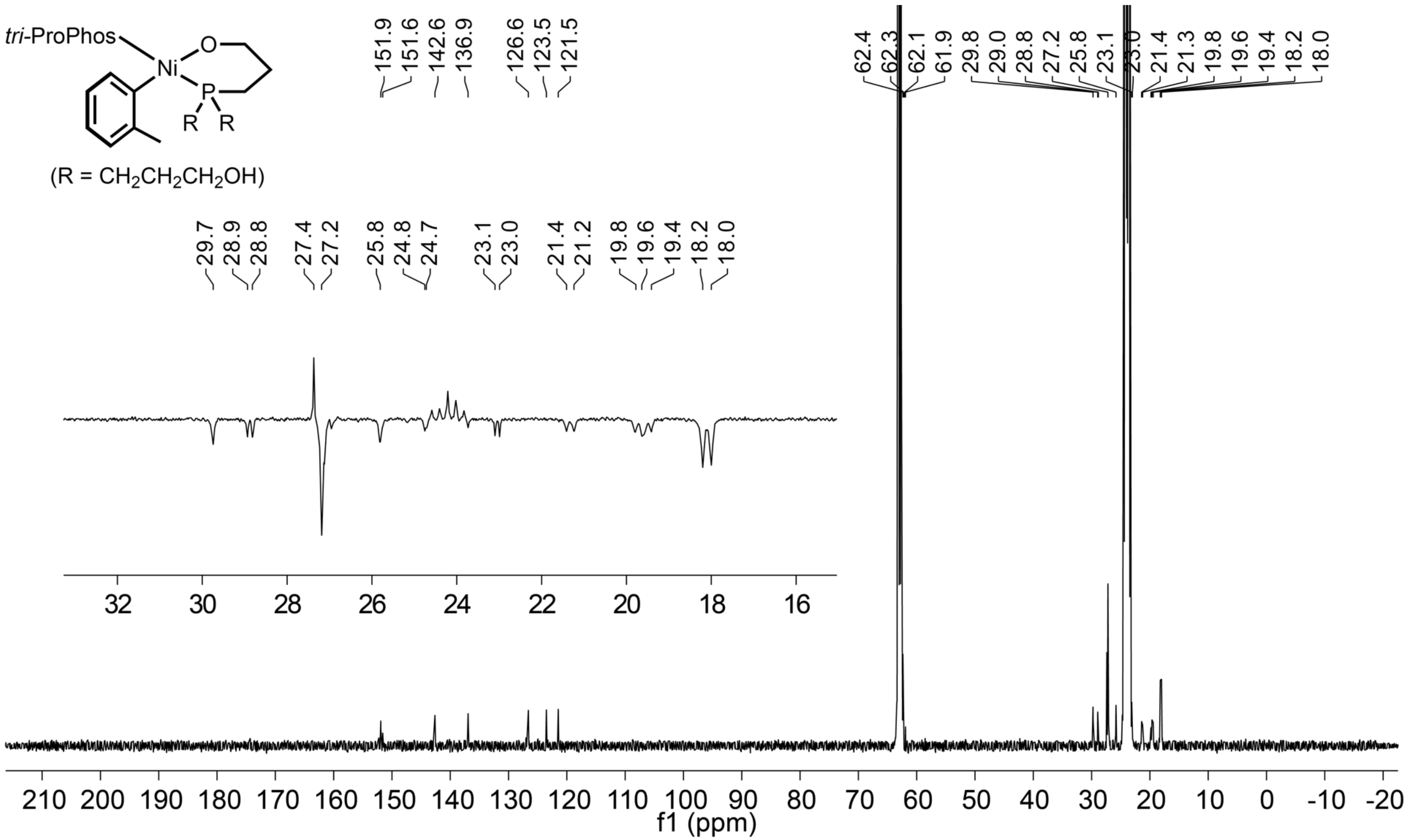

Figure S40. $^{13}C\{^{1}H\}$ NMR (100.67 MHz, *i*PrOD-*d*$_8$) spectrum of Ni(*tri*-ProPhos) metallacycle **29**. Deuterated solvent (•).

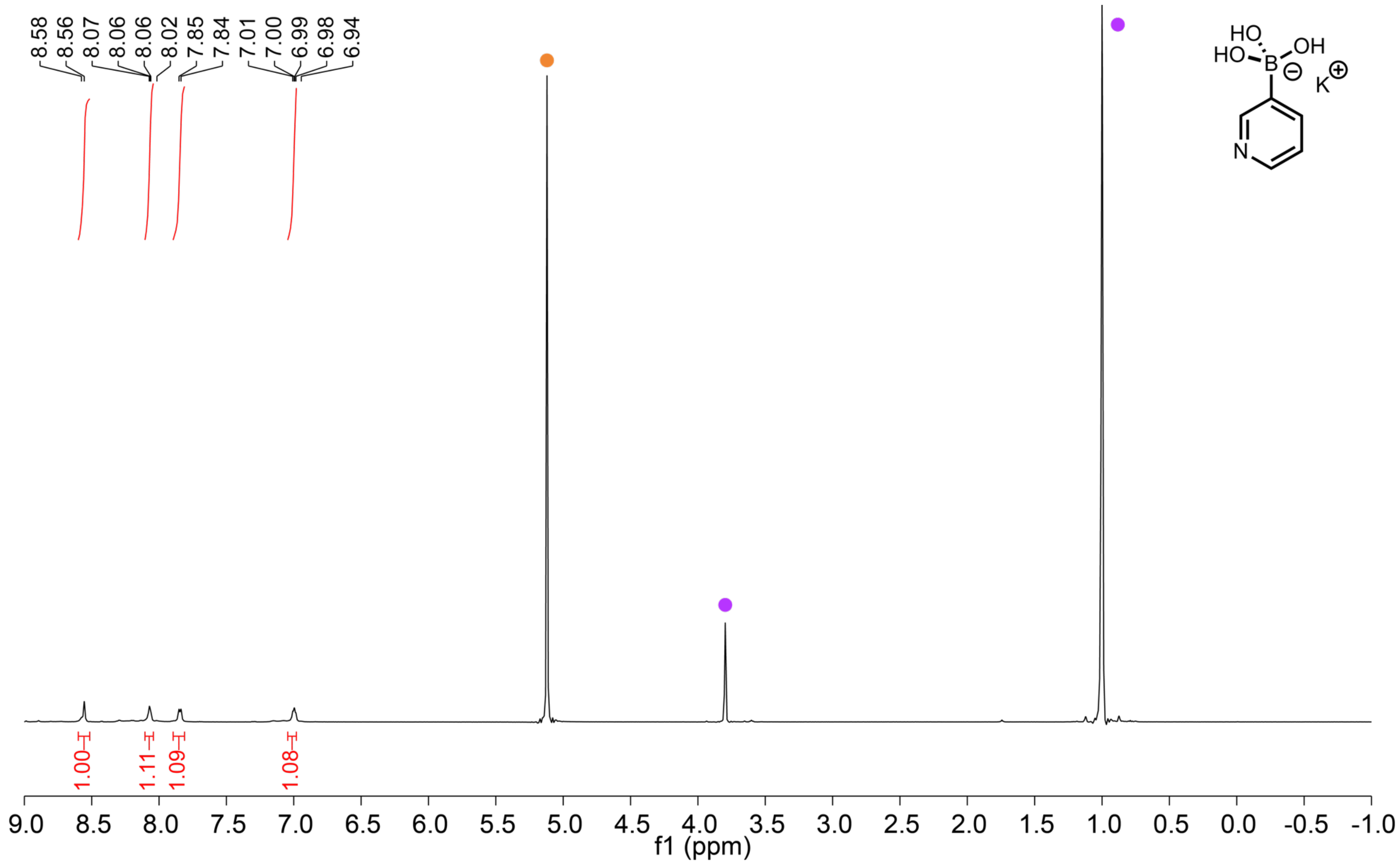

Figure S41. ^{1}H NMR (500.20 MHz, *i*PrOD-d_8) spectrum of K[B(3-Py)(OH)$_3$] **30**. H_2O (●) and residual proteo-solvent (●).

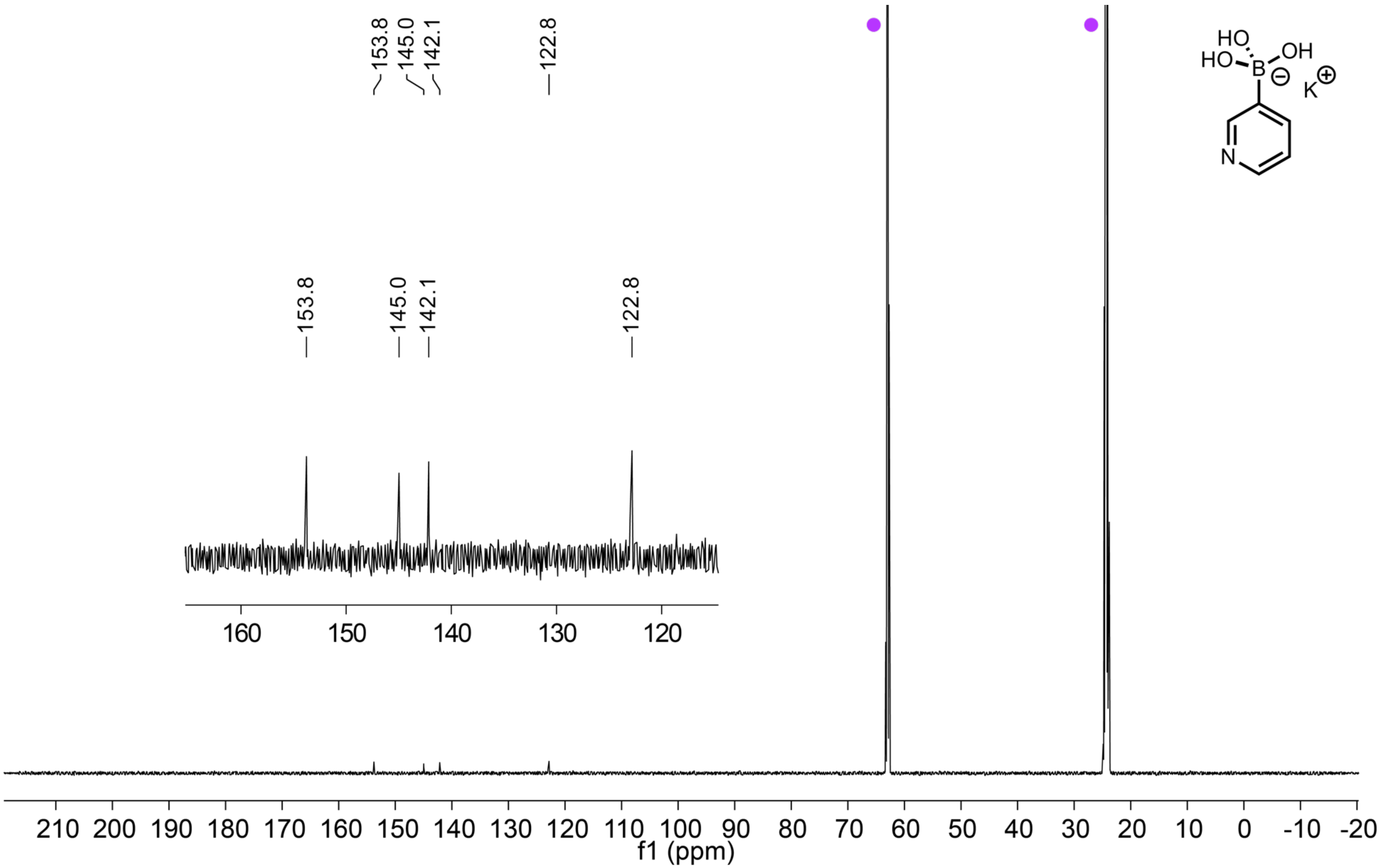

Figure S42. $^{13}C\{^1H\}$ NMR (125.79 MHz, *i*PrOD-d_8) spectrum of K[B(3-Py)(OH)$_3$] **30**. Deuterated solvent (•).

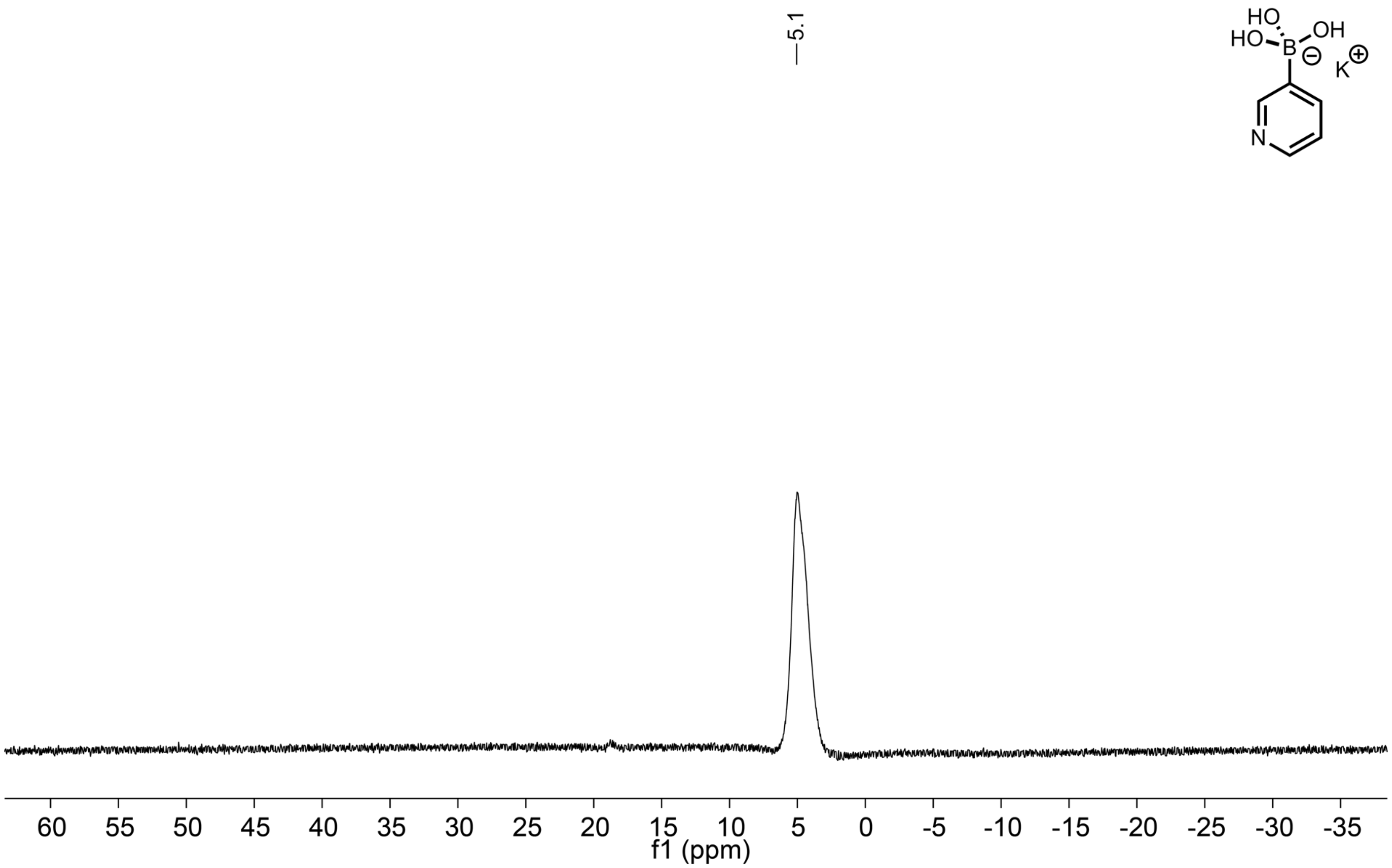

Figure S43. ^{11}B NMR (160.48 MHz, *i*PrOD-d_8) spectrum of K[B(3-Py)$(OH)_3$] **30**.

<u>NMR spectra of Ni-SMC products</u>

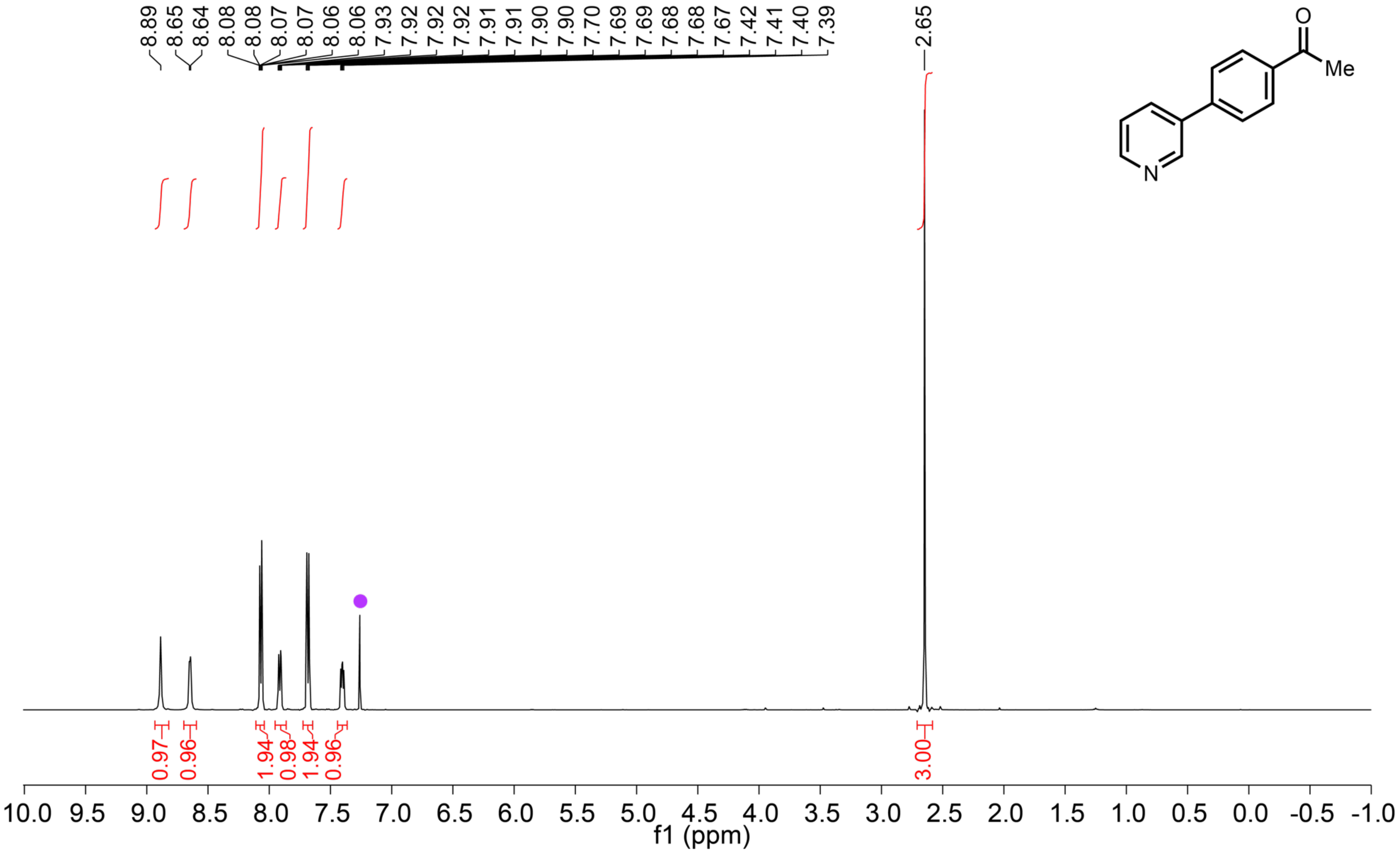

Figure S44. ^{1}H NMR (400.30 MHz, $CDCl_3$) spectrum of compound **7**. Residual proteo-solvent (•).

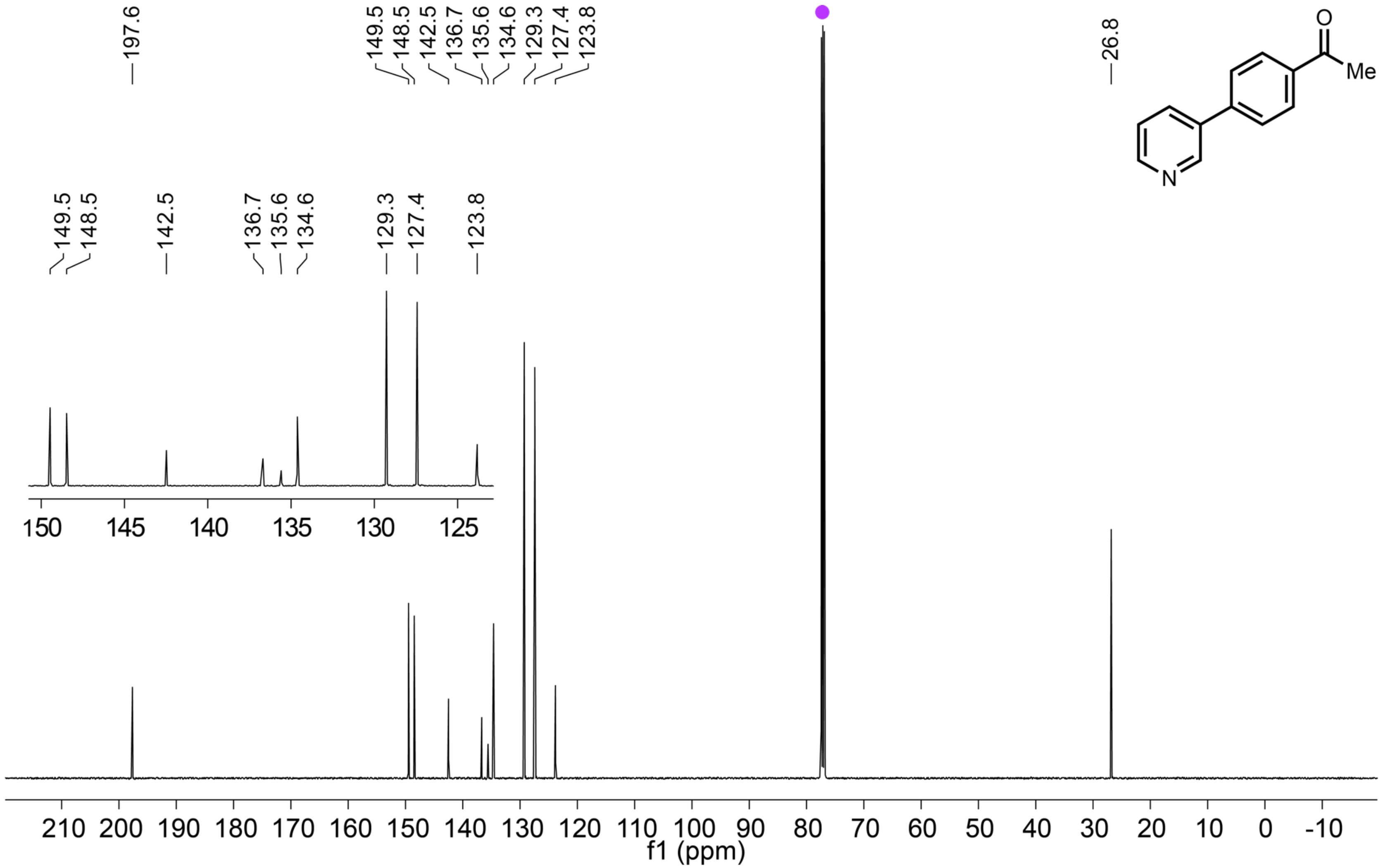

Figure S45. $^{13}C\{^{1}H\}$ NMR (100.67 MHz, $CDCl_3$) spectrum of compound **7**. Deuterated solvent (•).

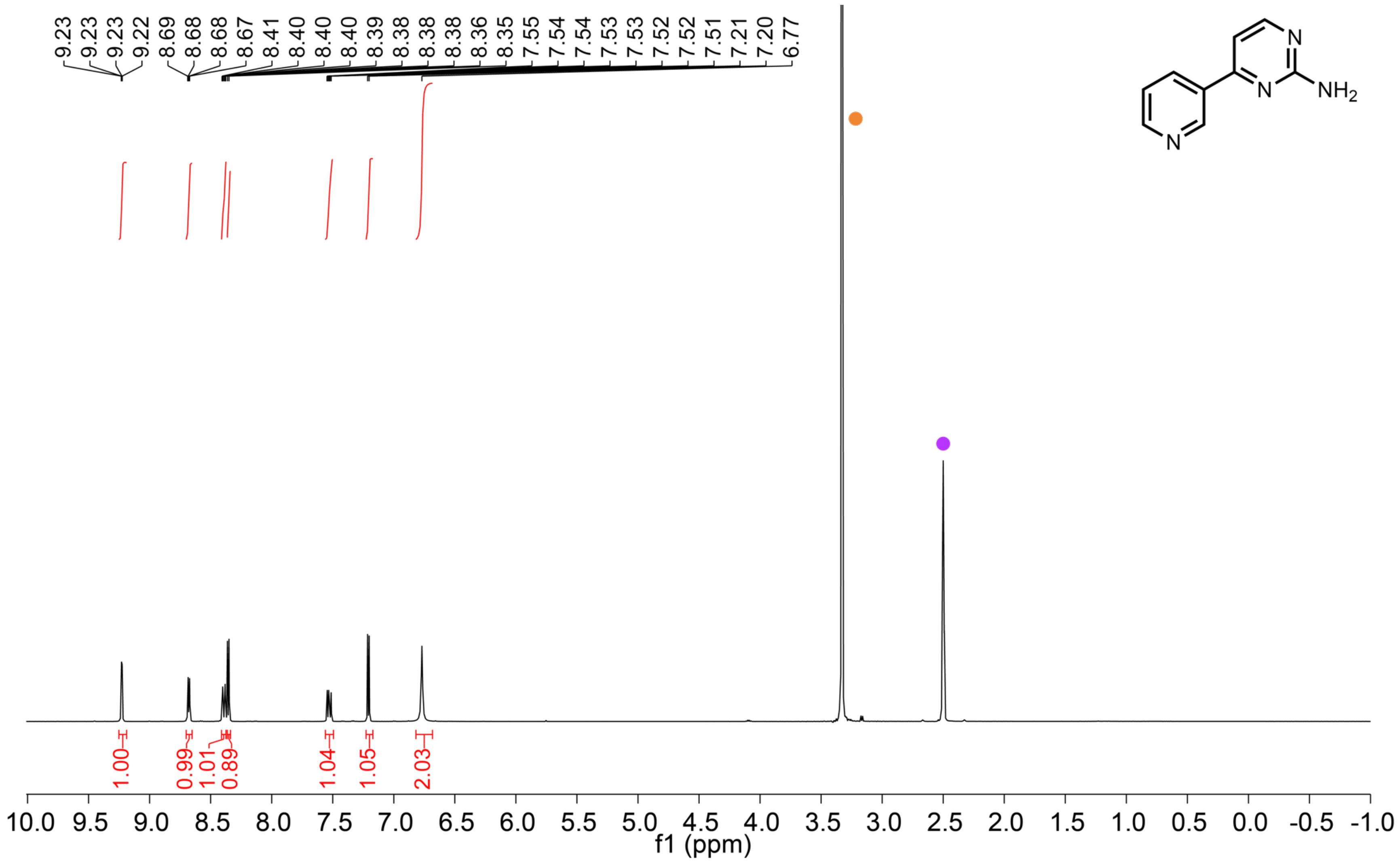

Figure S46. ^{1}H NMR (400.30 MHz, DMSO-d_6) spectrum of compound **10**. H_2O (●) and residual proteo-solvent (●).

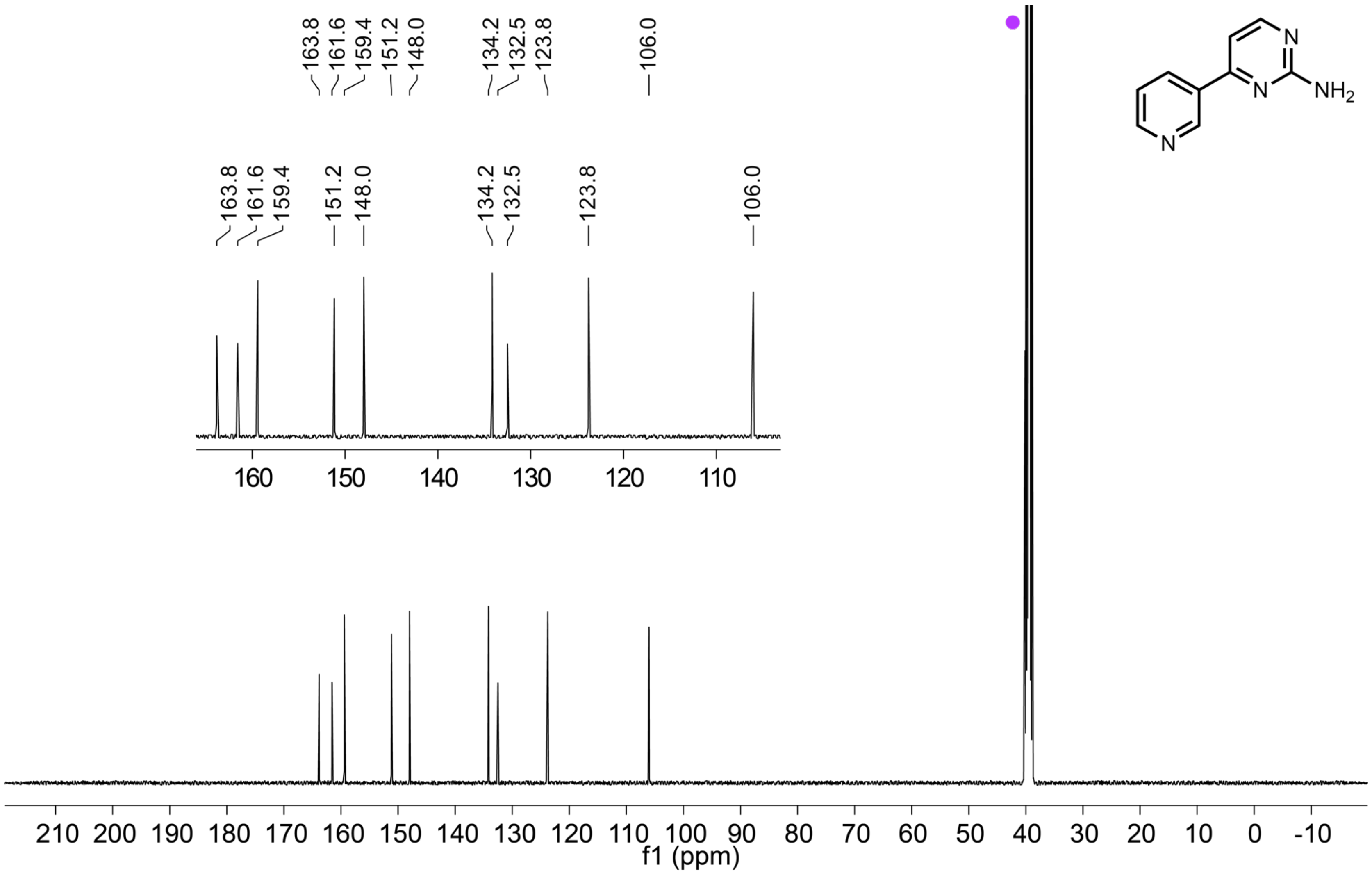

Figure S47. $^{13}C\{^{1}H\}$ NMR (100.67 MHz, DMSO-d_6) spectrum of compound **10**. Deuterated solvent (•).

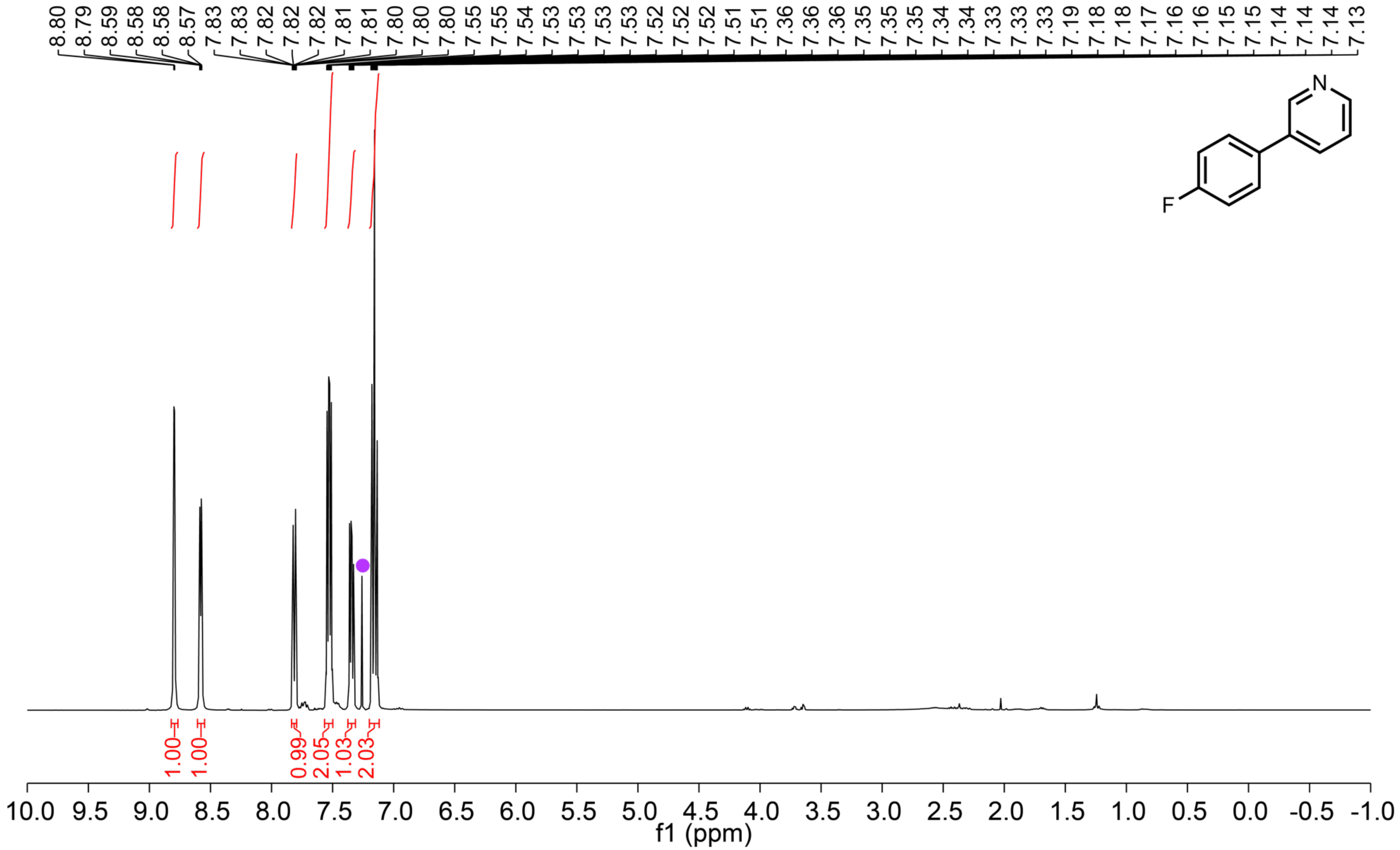

Figure S48. ^{1}H NMR (400.30 MHz, $CDCl_3$) spectrum of compound **11**. Residual proteo-solvent (•).

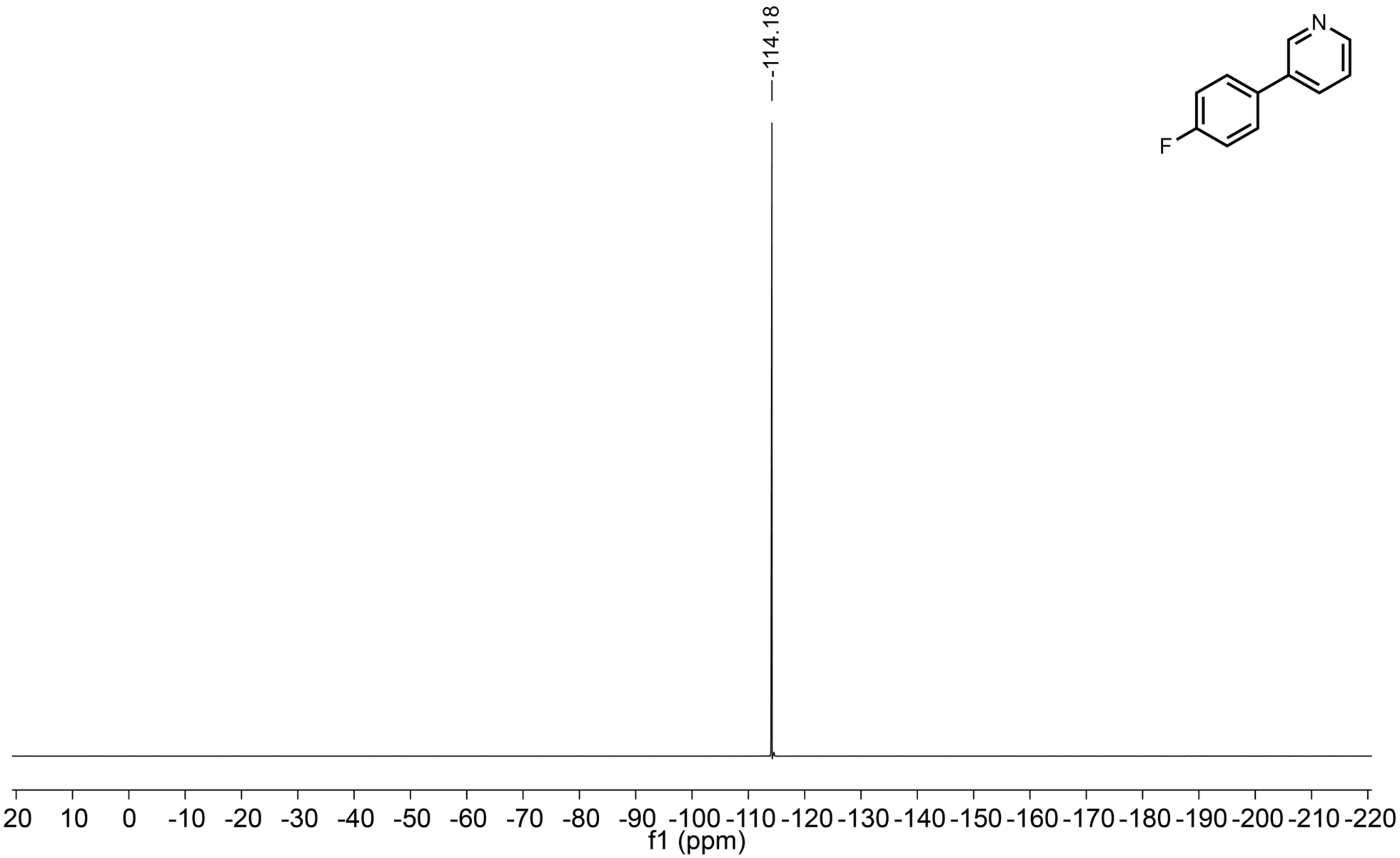

Figure S49. $^{19}F\{^1H\}$ NMR (470.61 MHz, $CDCl_3$) spectrum of compound **11**.

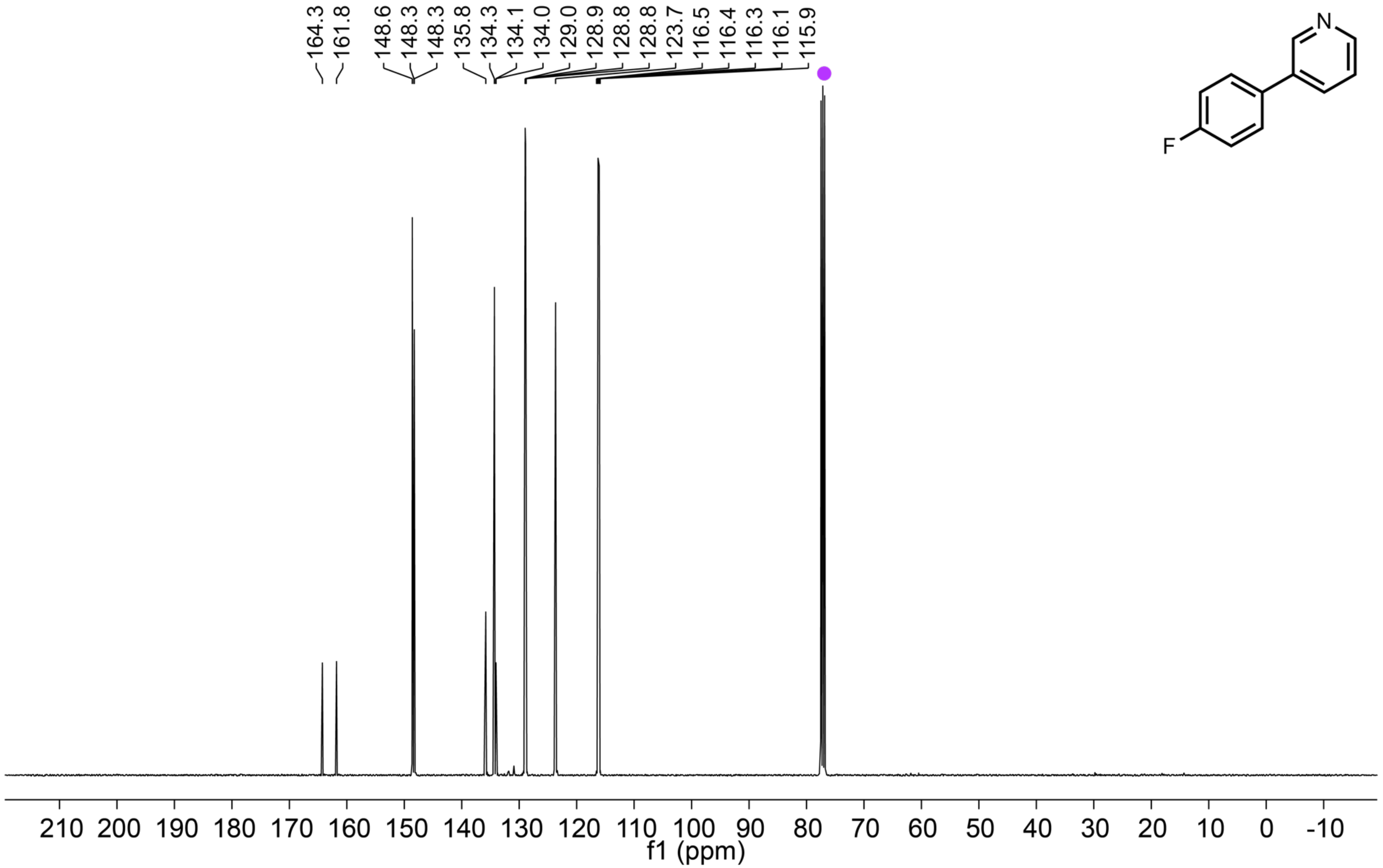

Figure S50. $^{13}C\{^{1}H\}$ NMR (100.67 MHz, $CDCl_3$) spectrum of compound **11**. Deuterated solvent (•).

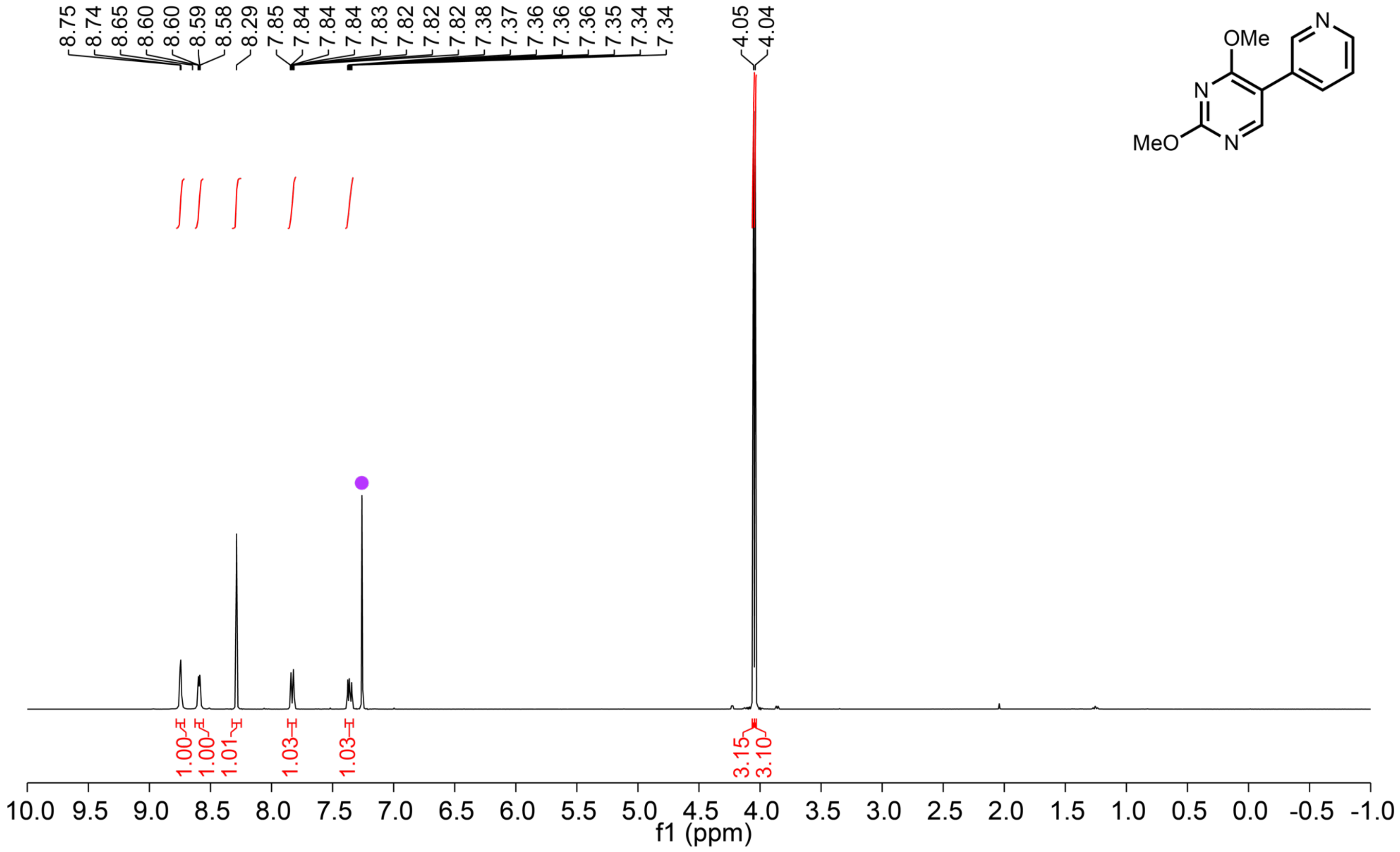

Figure S51. ^{1}H NMR (400.30 MHz, $CDCl_3$) spectrum of compound **12**. Residual proteo-solvent (•).

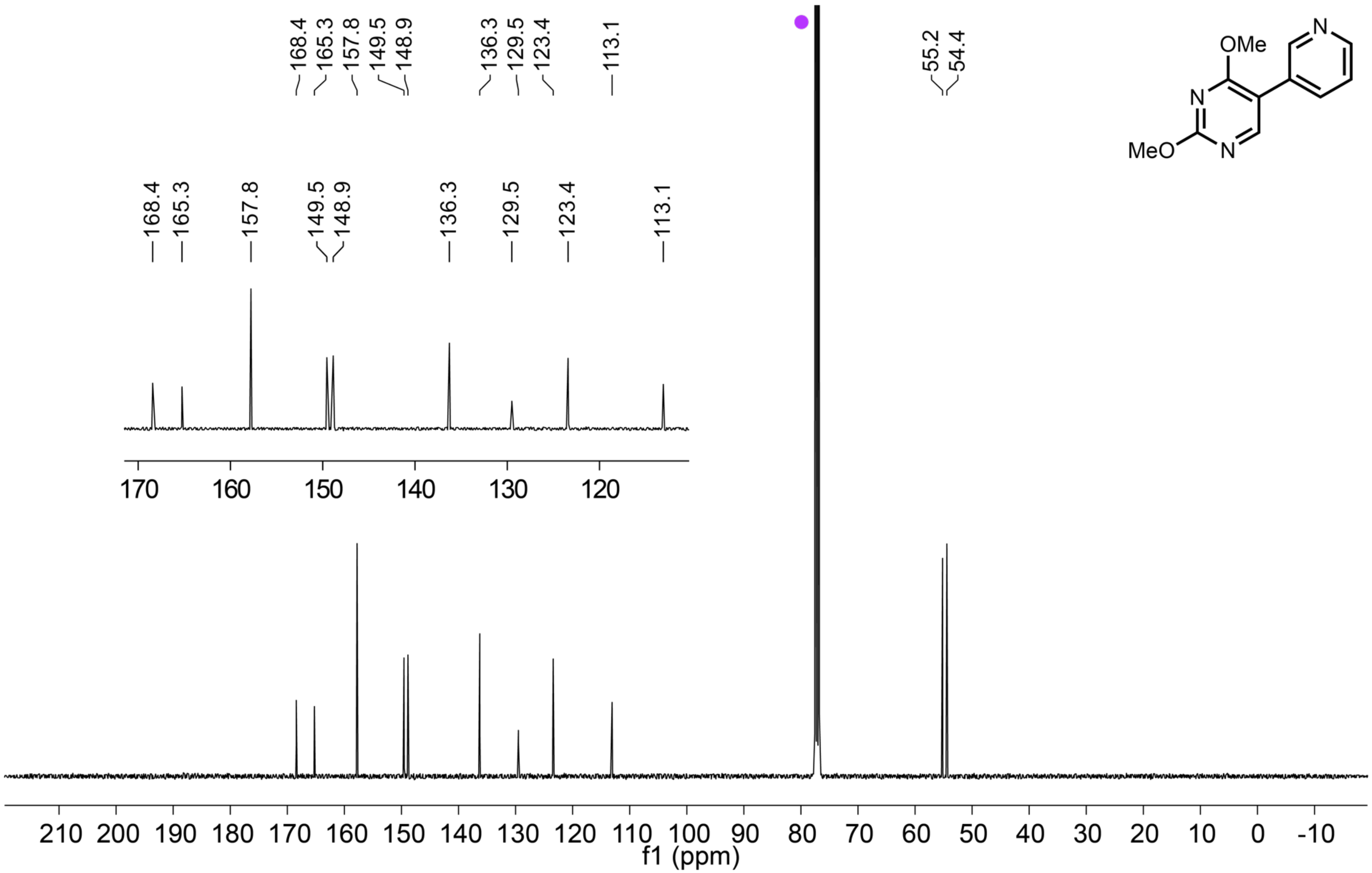

Figure S52. $^{13}C\{^{1}H\}$ NMR (100.67 MHz, $CDCl_3$) spectrum of compound **12**. Deuterated solvent (•).

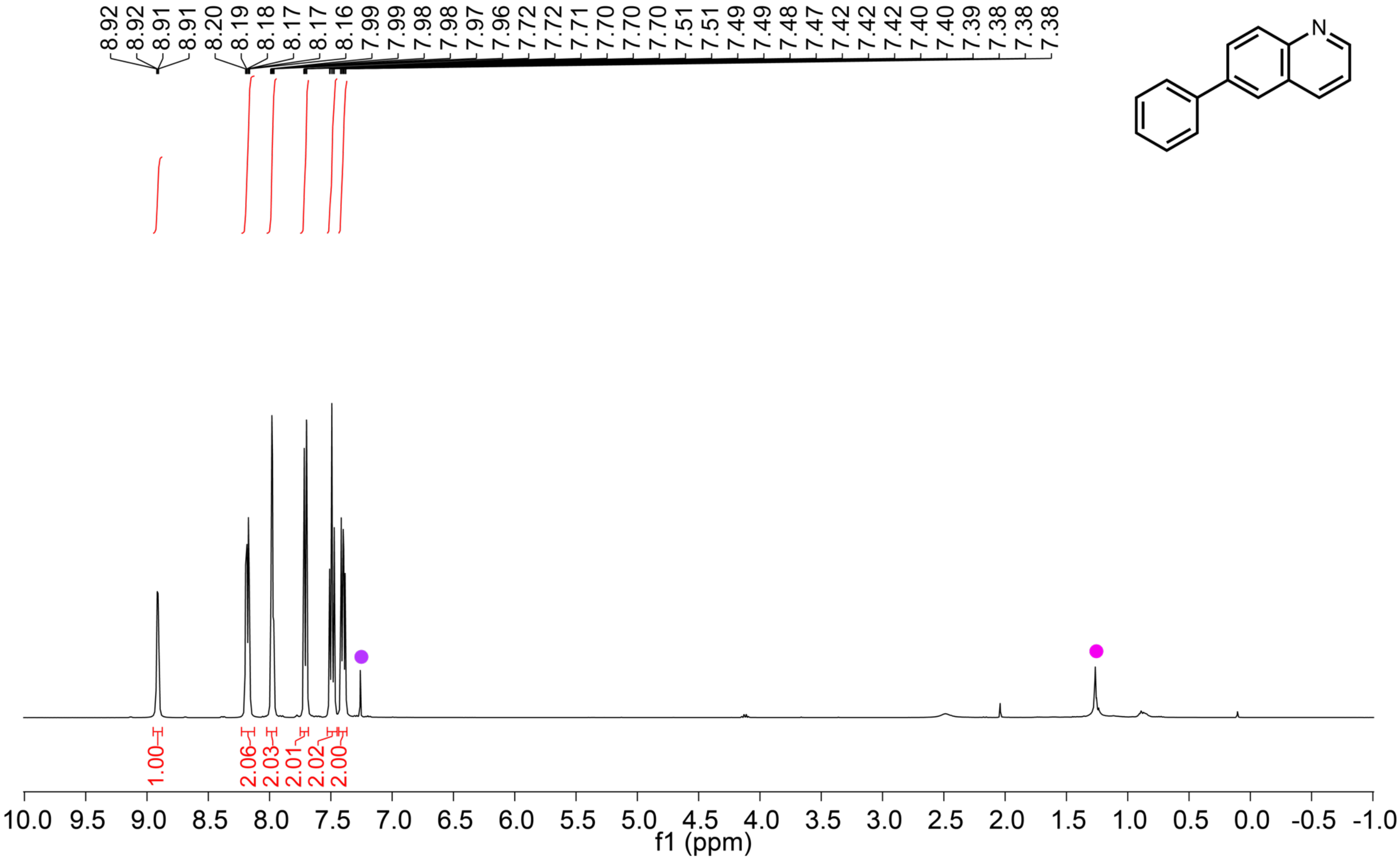

Figure S53. ^{1}H NMR (400.30 MHz, $CDCl_3$) spectrum of compound **13**. Residual proteo-solvent (•) and grease (•).

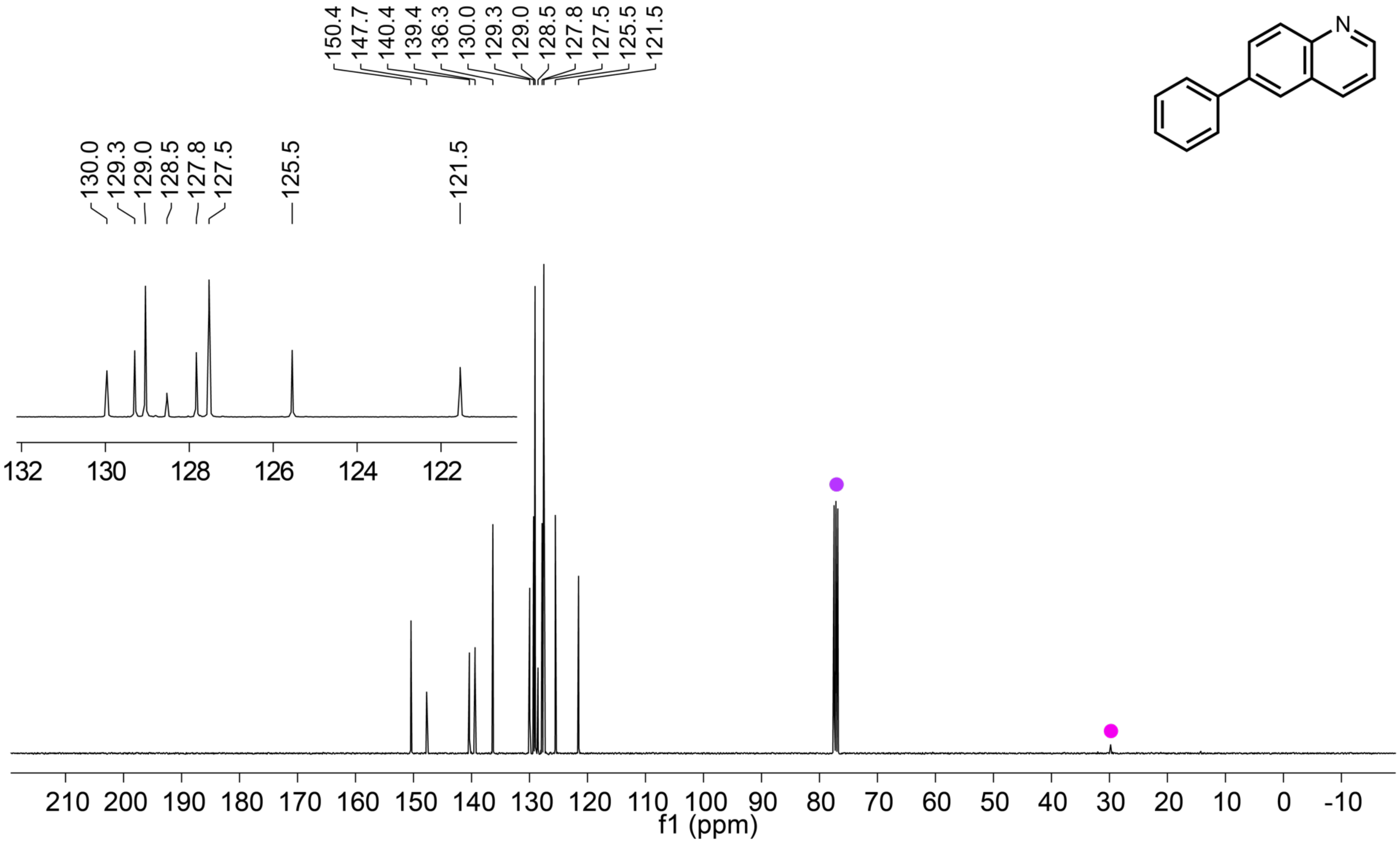

Figure S54. $^{13}C\{^{1}H\}$ NMR (100.67 MHz, $CDCl_3$) spectrum of compound **13**. Deuterated solvent (•) and grease (•).

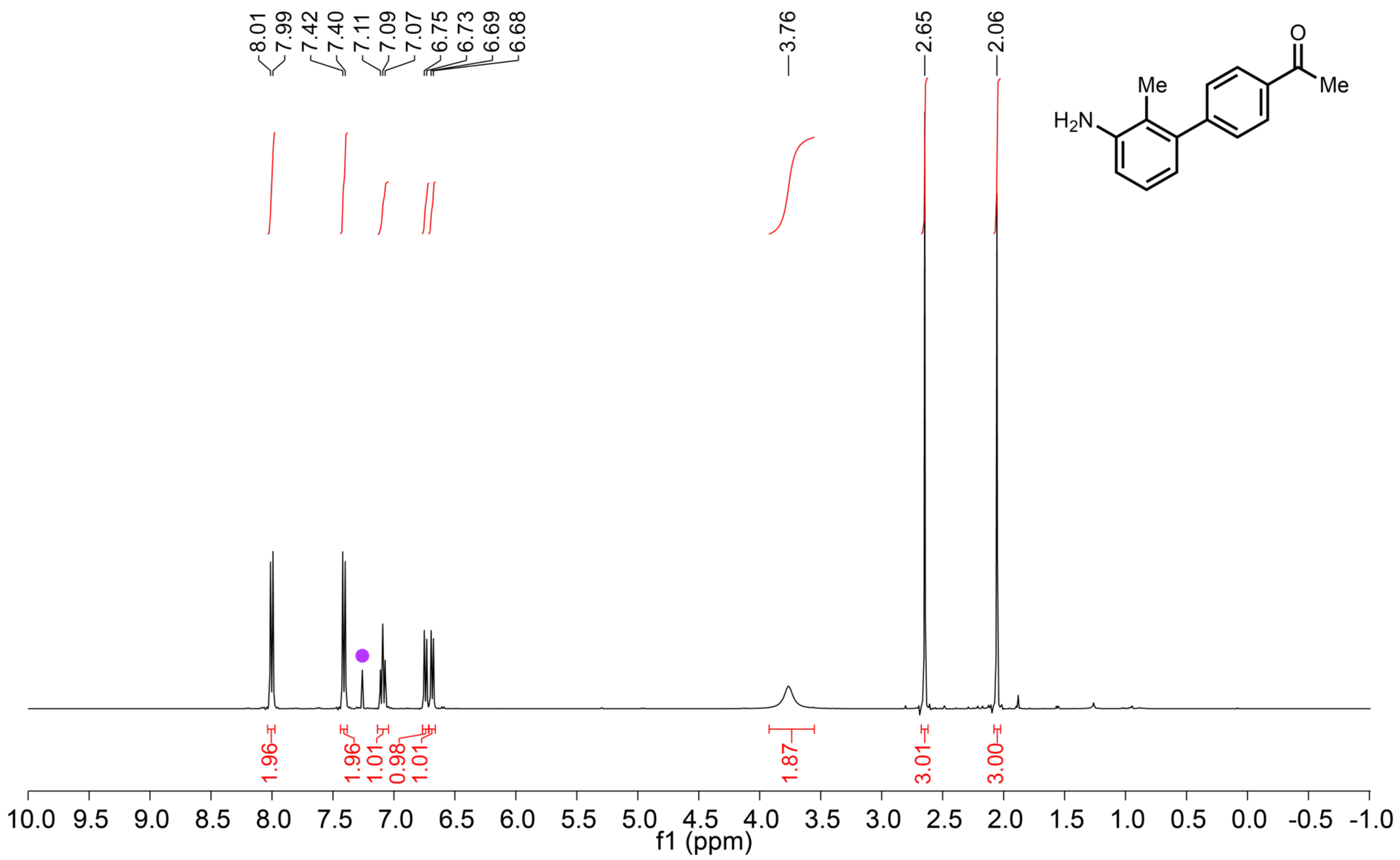

Figure S55. ^{1}H NMR (400.30 MHz, $CDCl_3$) spectrum of compound **14**. Residual proteo-solvent (•).

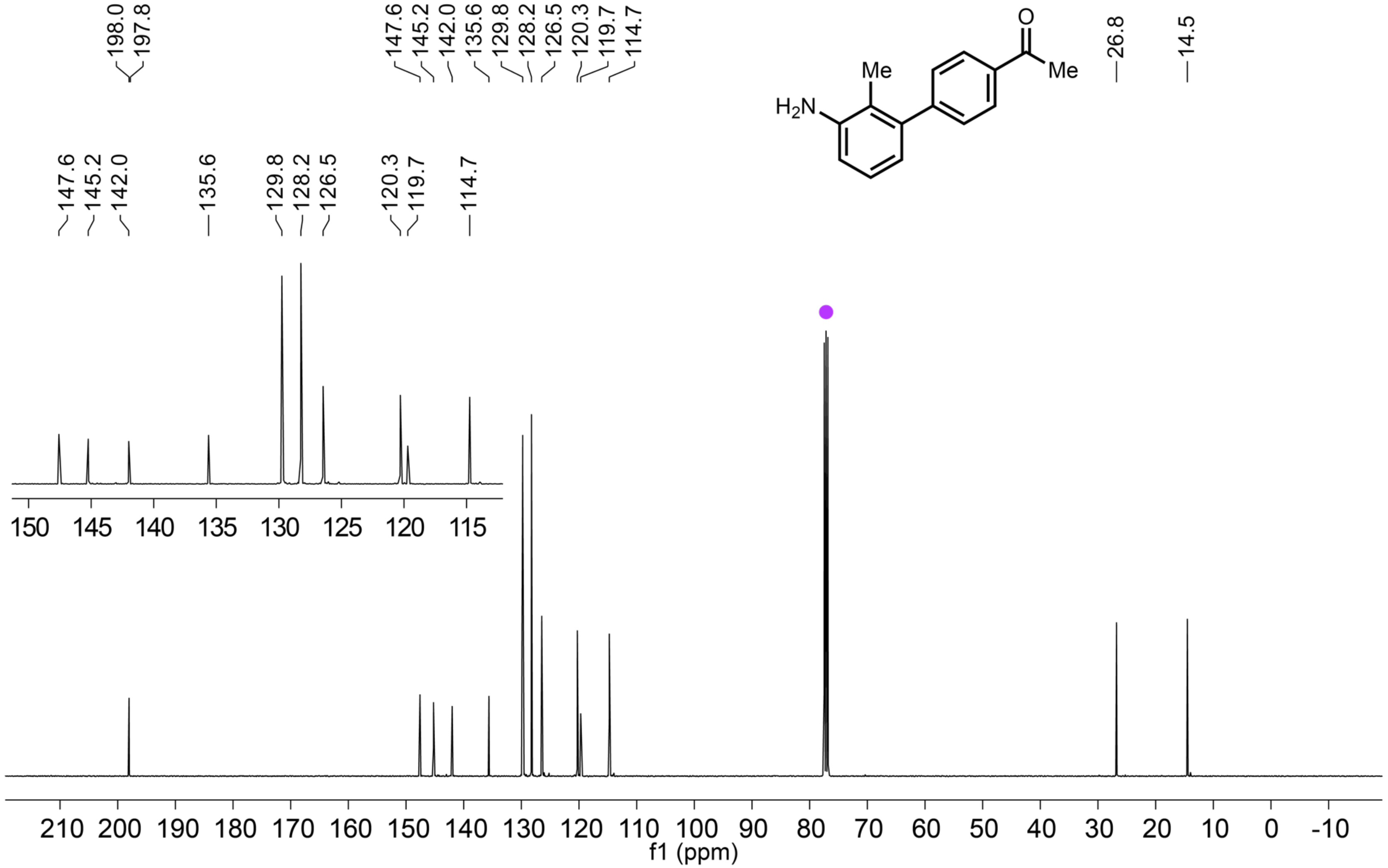

Figure S56. $^{13}C\{^{1}H\}$ NMR (100.67 MHz, $CDCl_3$) spectrum of compound **14**. Deuterated solvent (•).

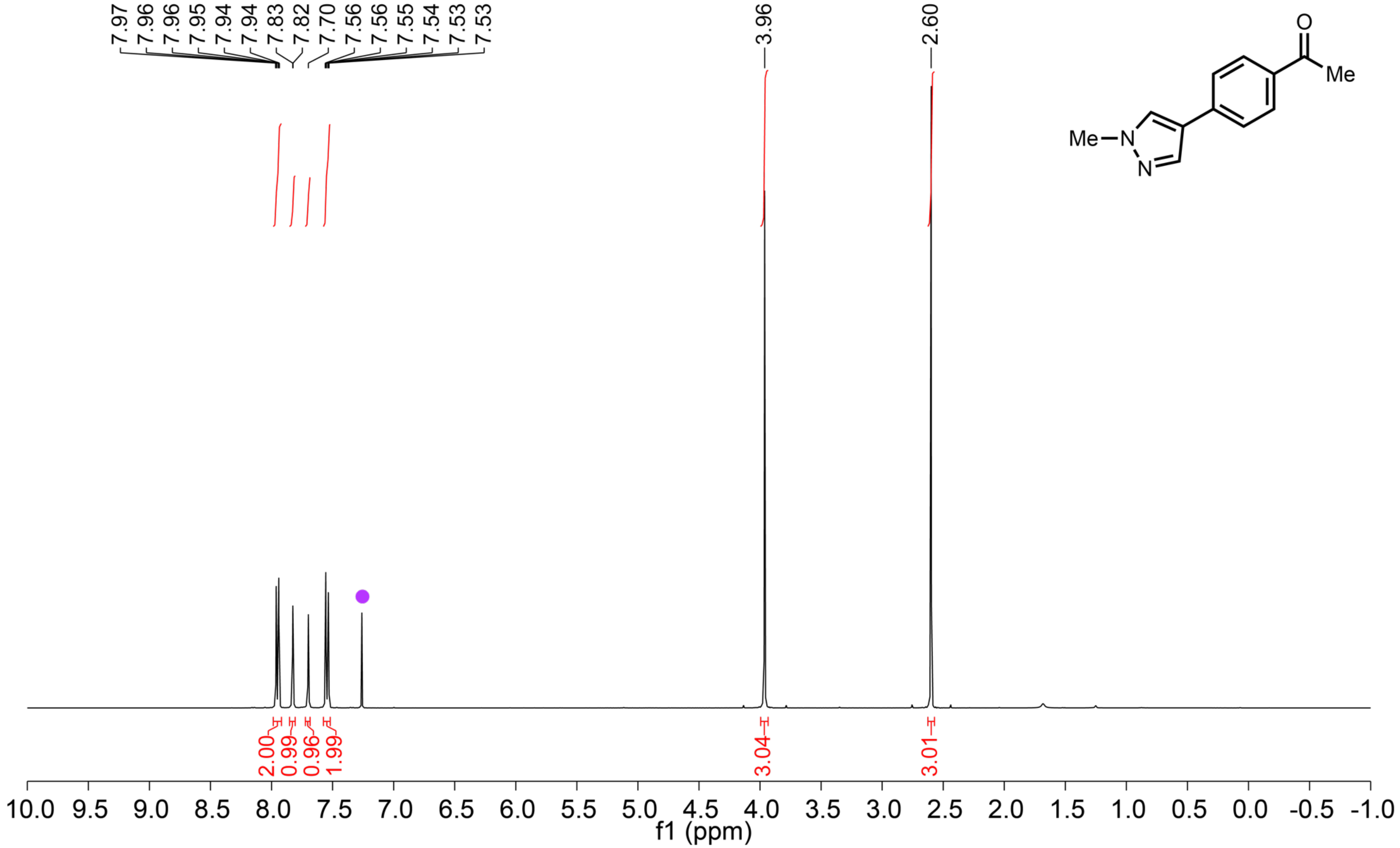

Figure S57. ^{1}H NMR (400.30 MHz, $CDCl_3$) spectrum of compound **15**. Residual proteo-solvent (•).

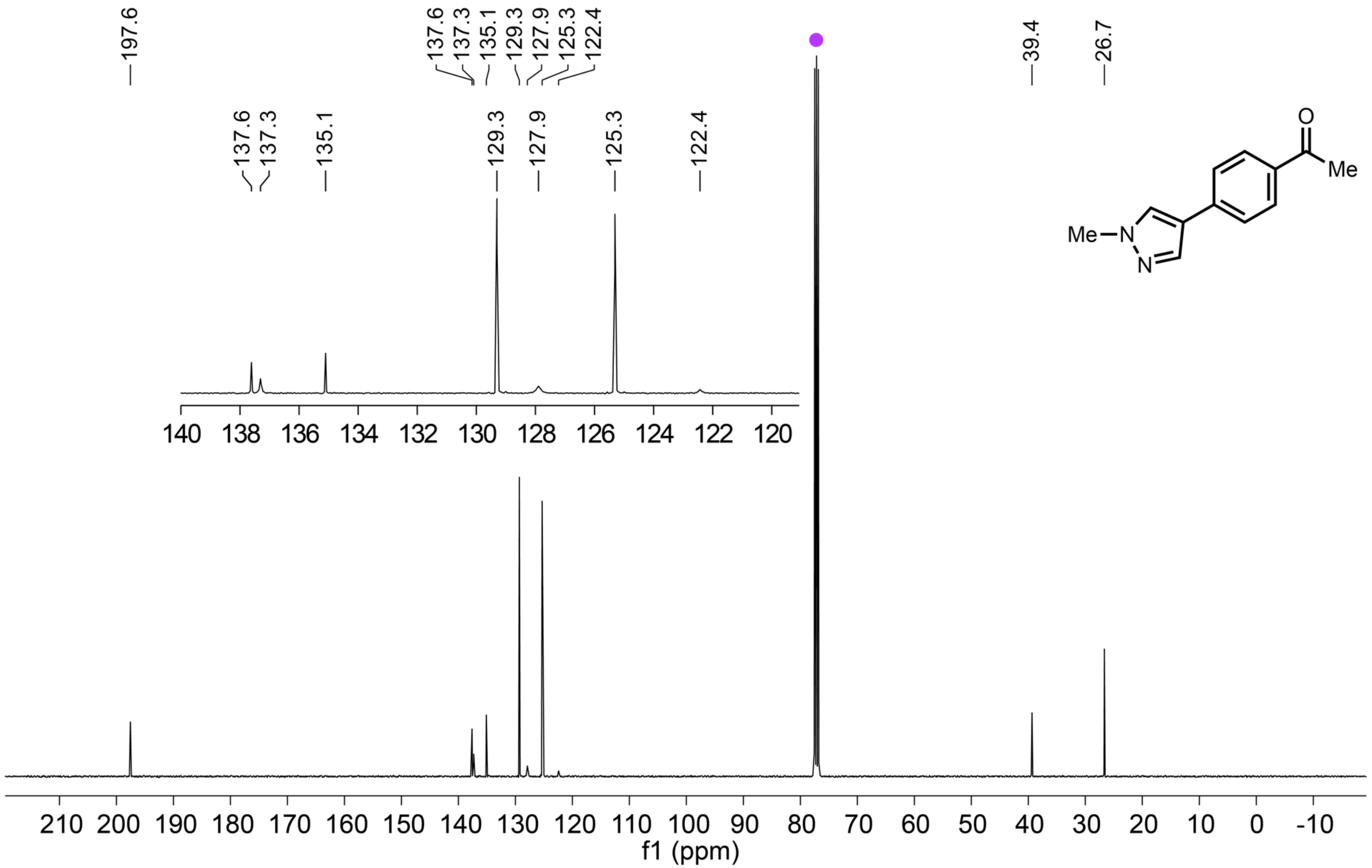

Figure S58. $^{13}C\{^1H\}$ NMR (100.67 MHz, $CDCl_3$) spectrum of compound **15**. Deuterated solvent (•).

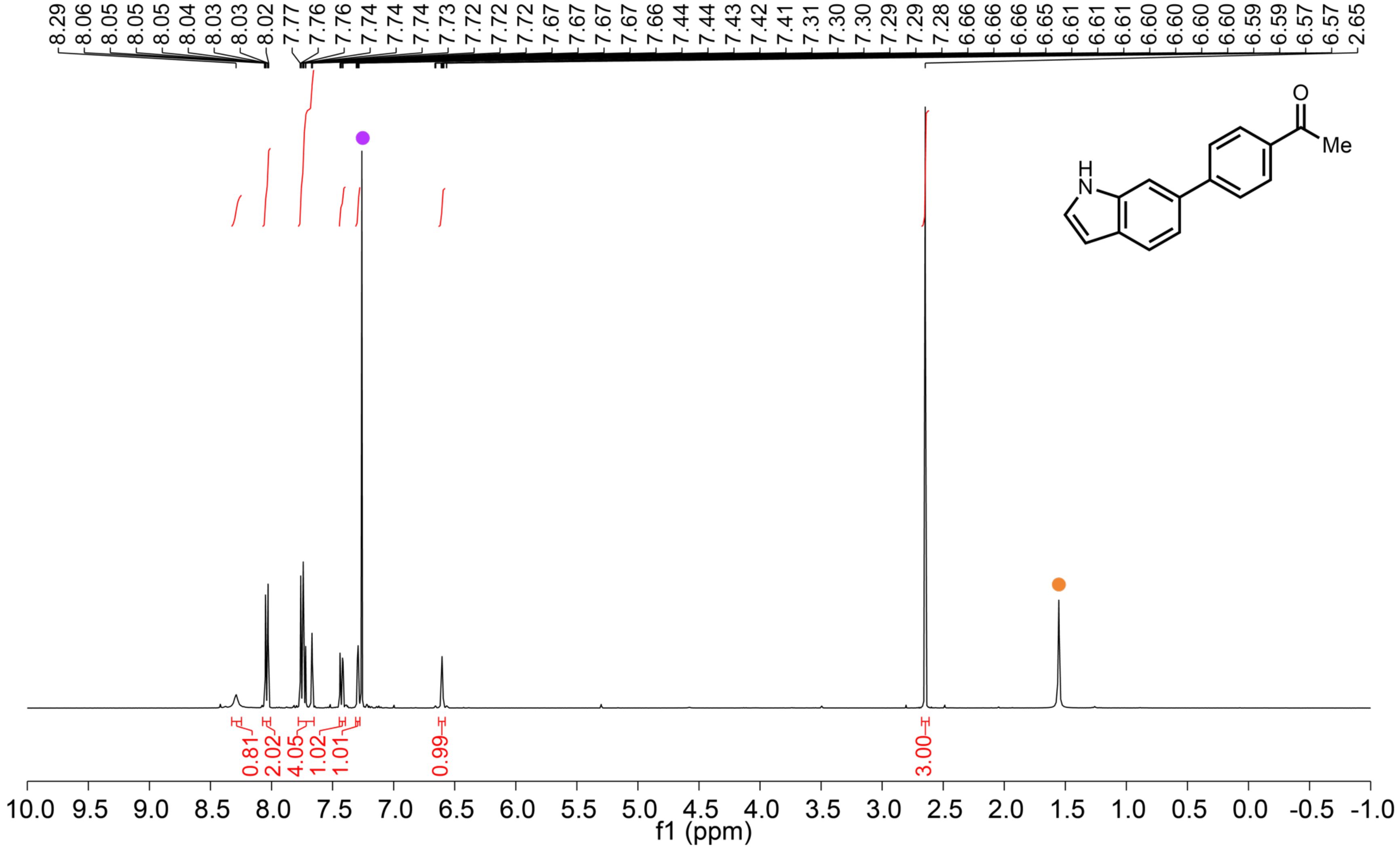

Figure S59. ^{1}H NMR (400.30 MHz, $CDCl_3$) spectrum of compound **16**. H_2O (•) and residual proteo-solvent (•).

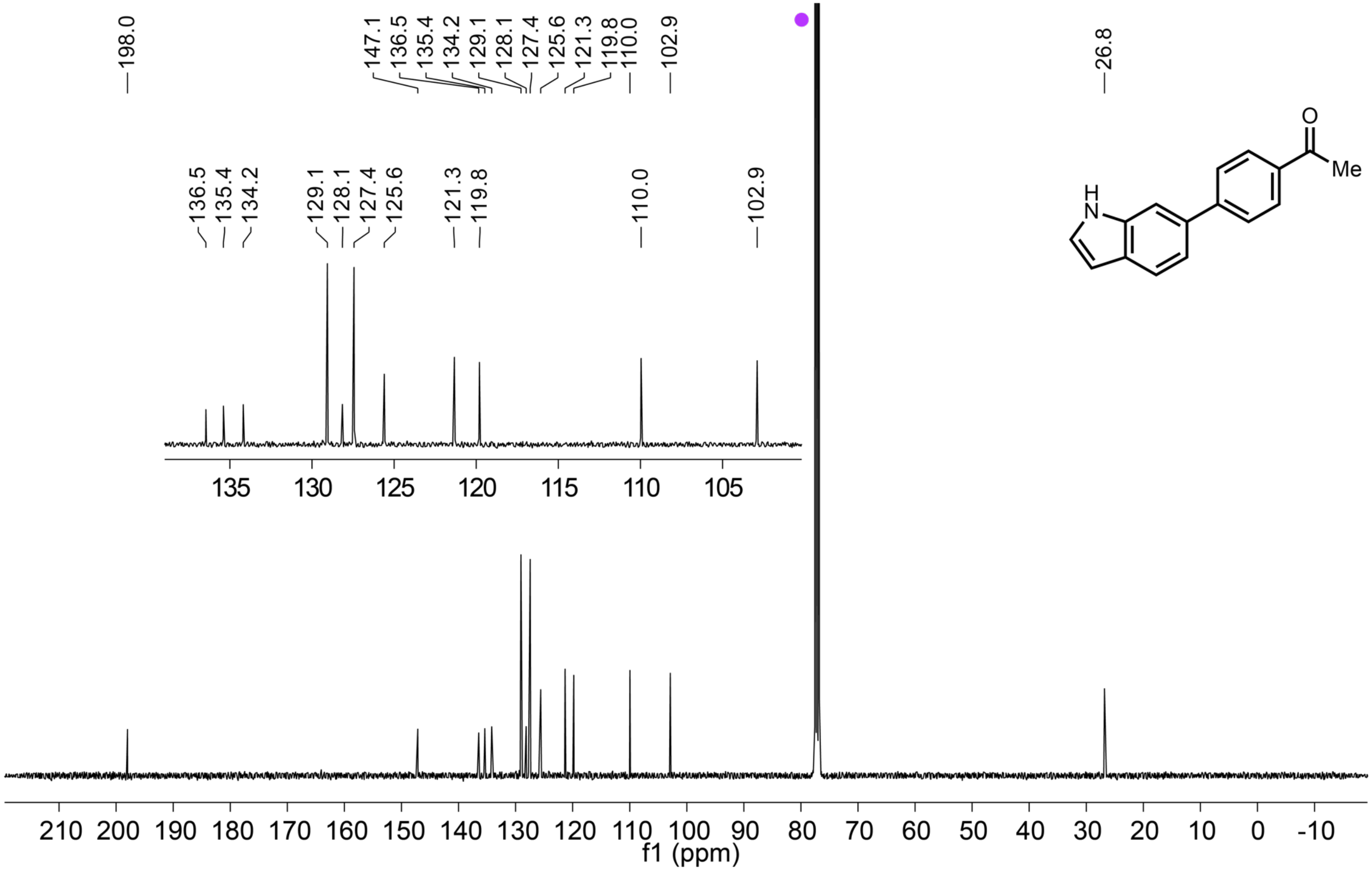

Figure S60. $^{13}C\{^{1}H\}$ NMR (100.67 MHz, $CDCl_3$) spectrum of compound **16**. Deuterated solvent (•).

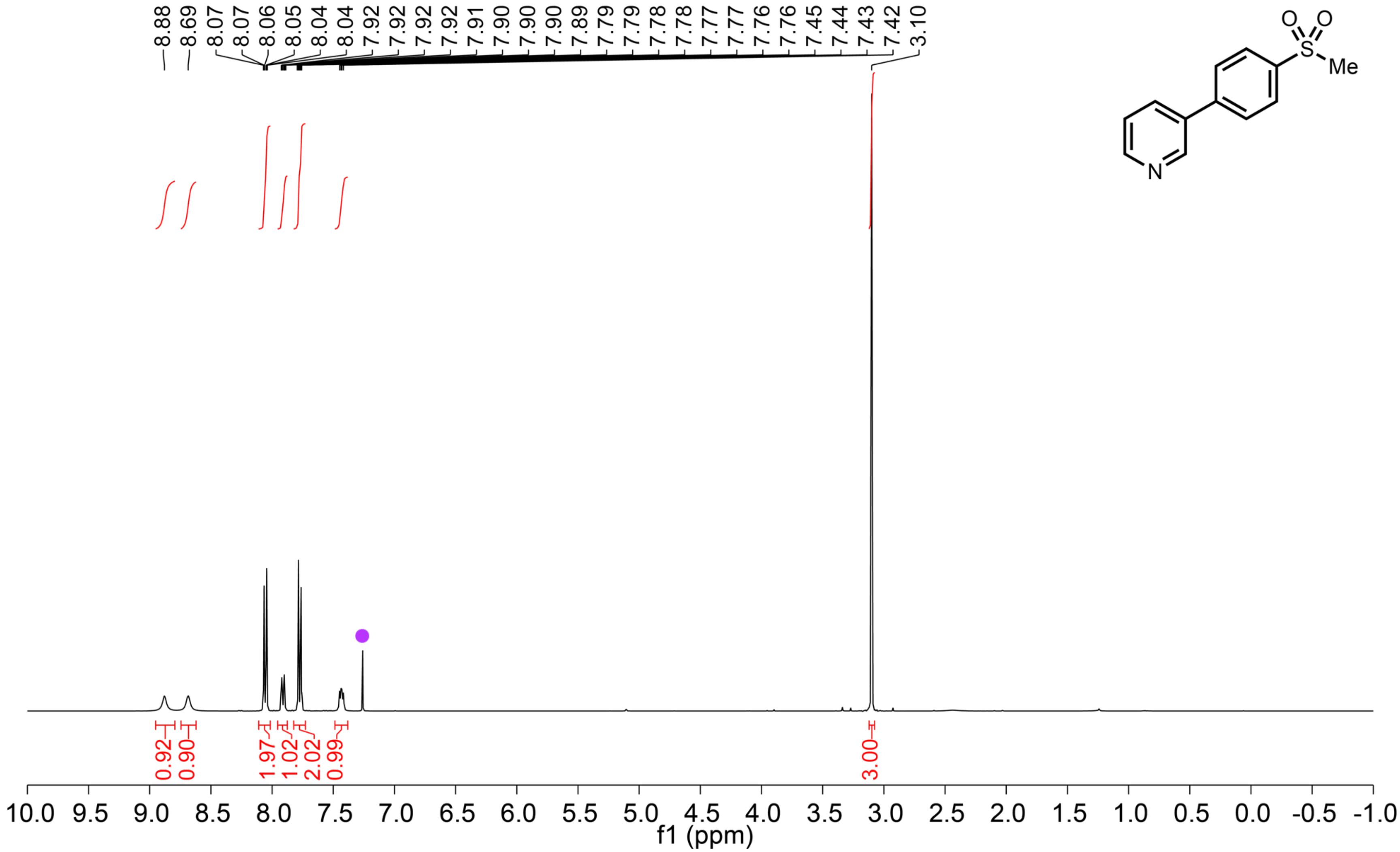

Figure S61. ^{1}H NMR (400.30 MHz, $CDCl_3$) spectrum of compound **17**. Residual proteo-solvent (•).

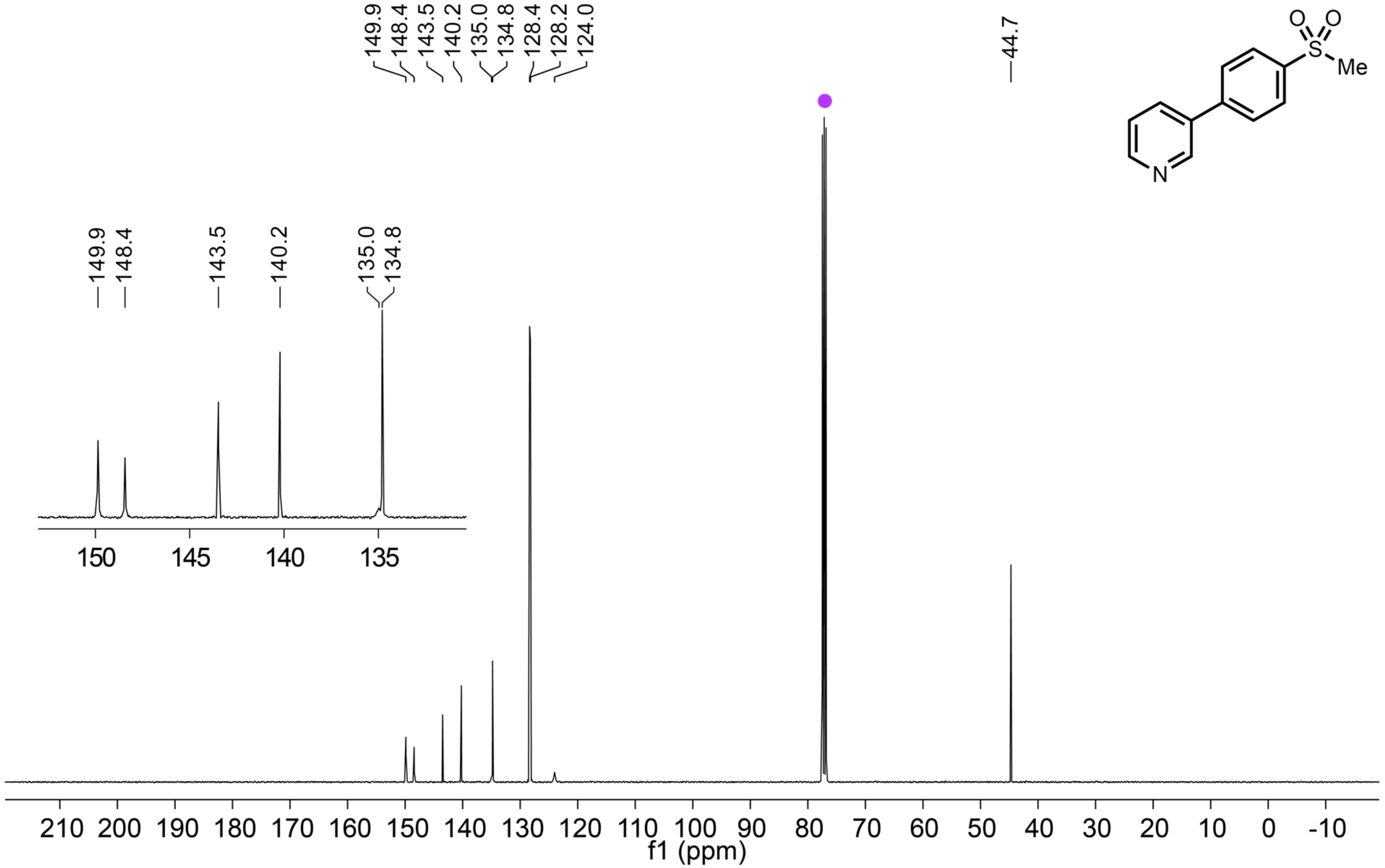

Figure S62. $^{13}C\{^1H\}$ NMR (100.67 MHz, $CDCl_3$) spectrum of compound **17**. Deuterated solvent (•).

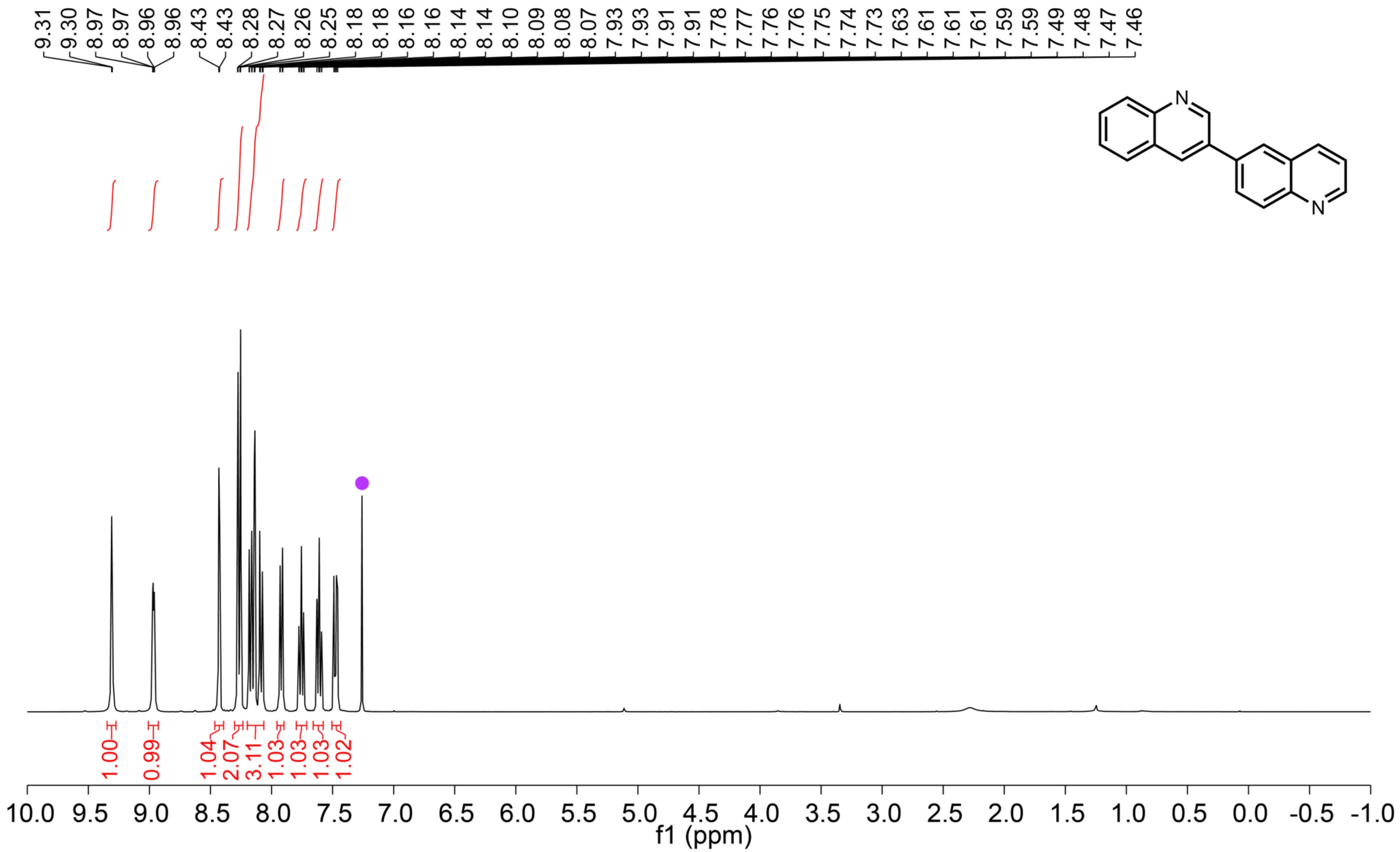

Figure S63. ^{1}H NMR (400.30 MHz, $CDCl_3$) spectrum of compound **18**. Residual proteo-solvent (•).

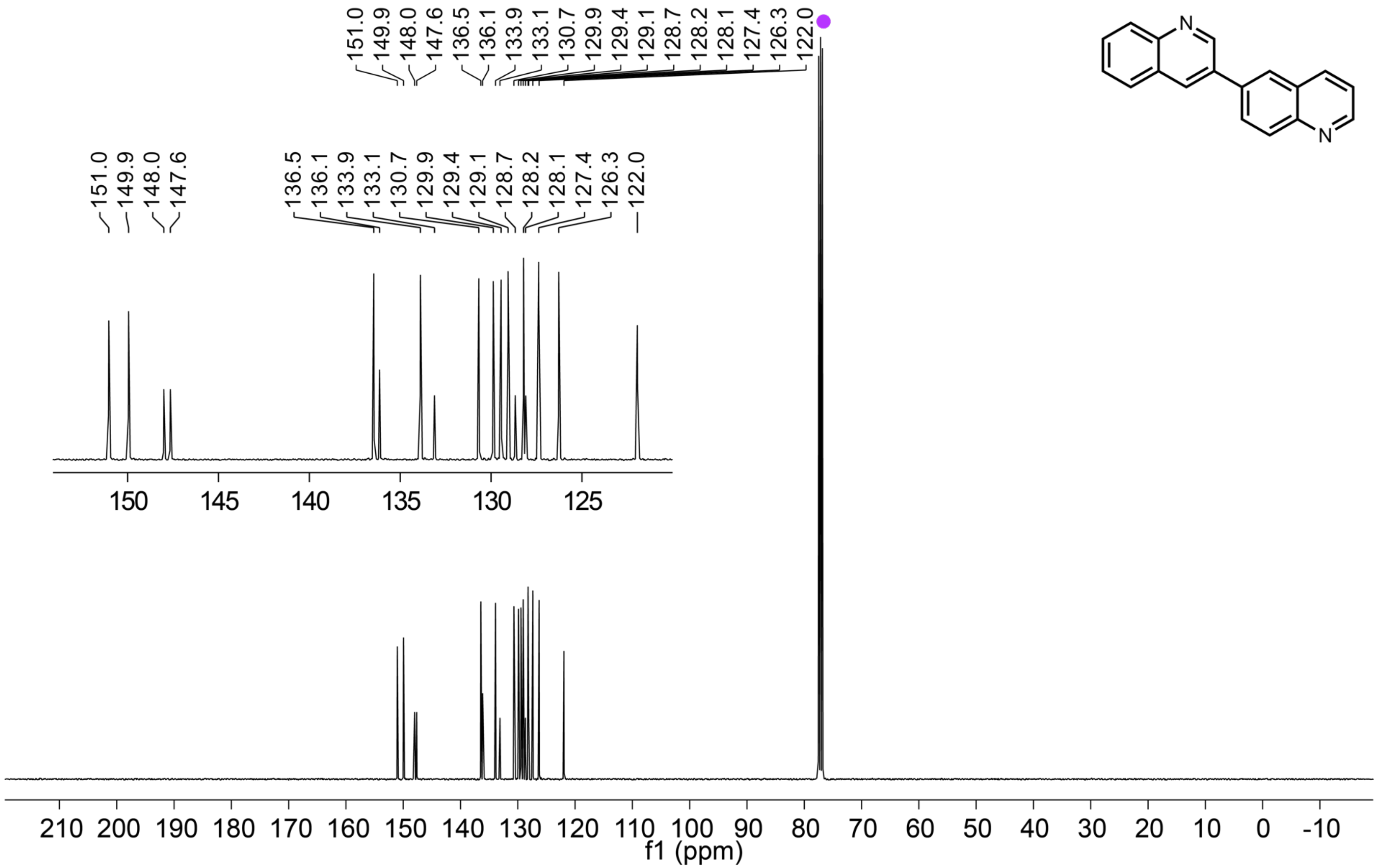

Figure S64. $^{13}C\{^1H\}$ NMR (100.67 MHz, $CDCl_3$) spectrum of compound **18**. Deuterated solvent (•).

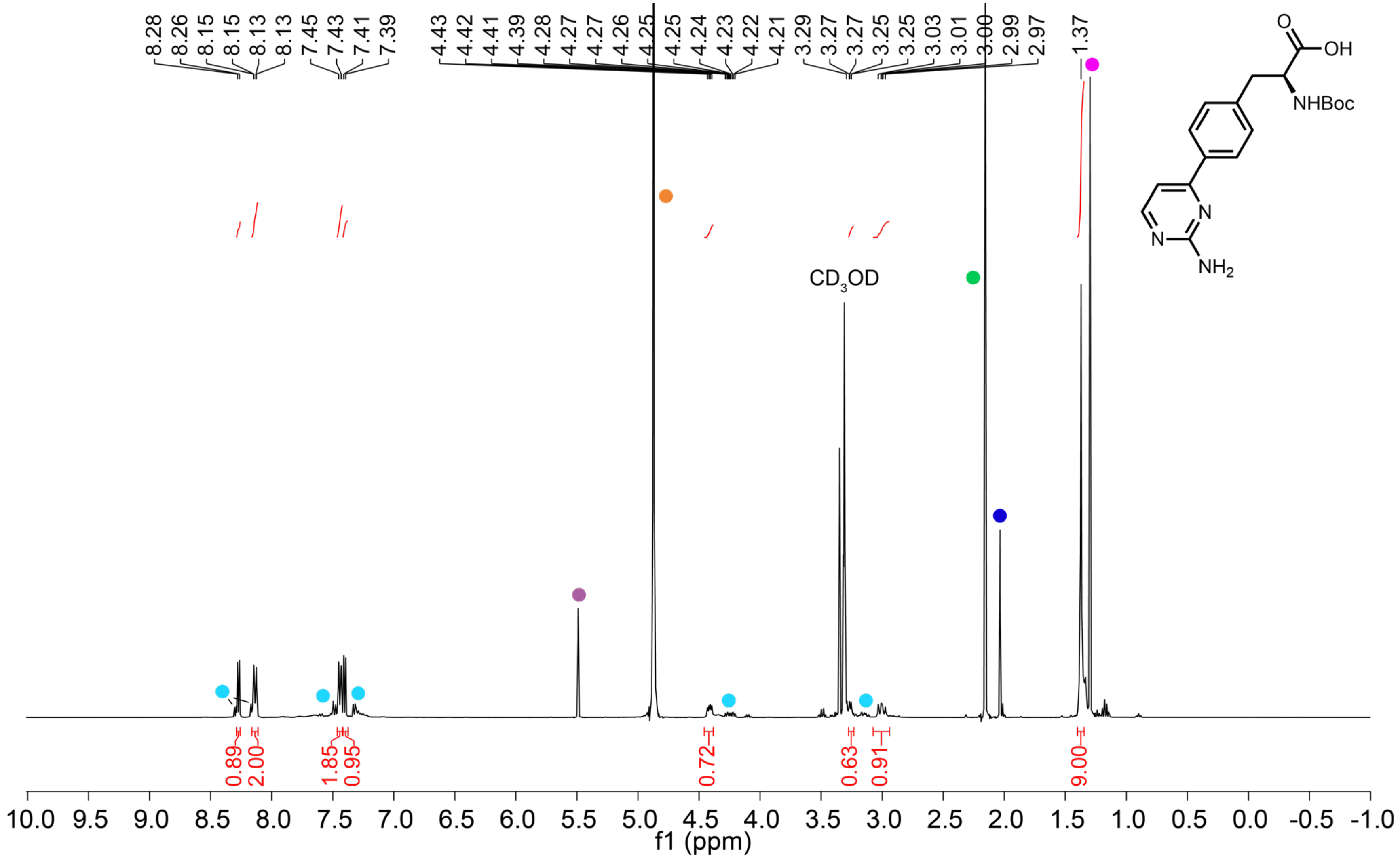

Figure S65. ^{1}H NMR (400.30 MHz, CD_3OD) spectrum of the crude of compound **19**. Unidentified impurity (•), DCM (•), H_2O (•), acetone (•), MeCN (•), grease (•).

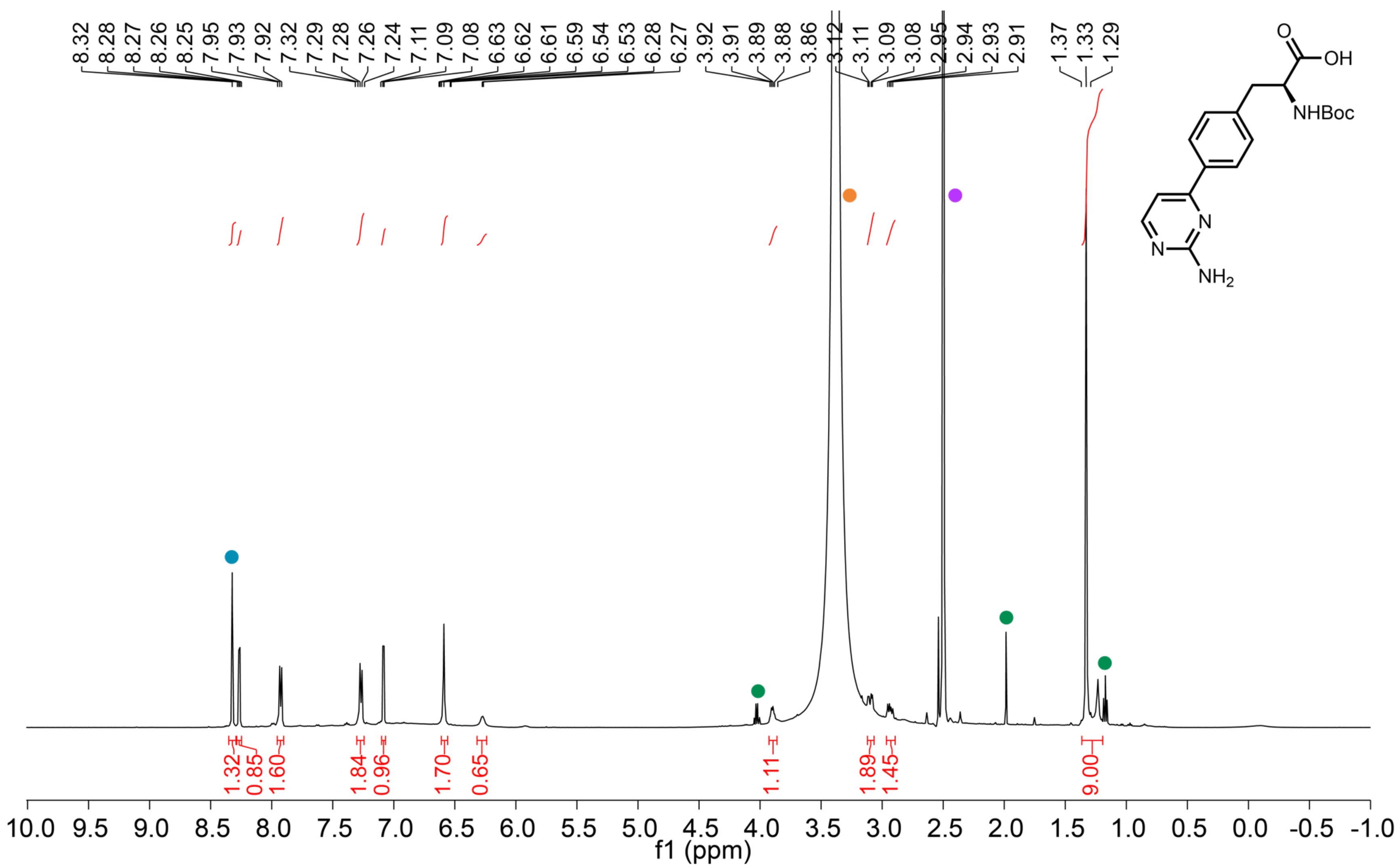

Figure S66. ^{1}H NMR (500.20 MHz, DMSO-d_6) spectrum of compound **19**. EtOAc (●), H_2O (●) and residual proteo-solvent (●). Note: after purification by HPLC, the isolated compound contains 1 equiv of formic acid (●). The formic acid is from the HPLC eluent.

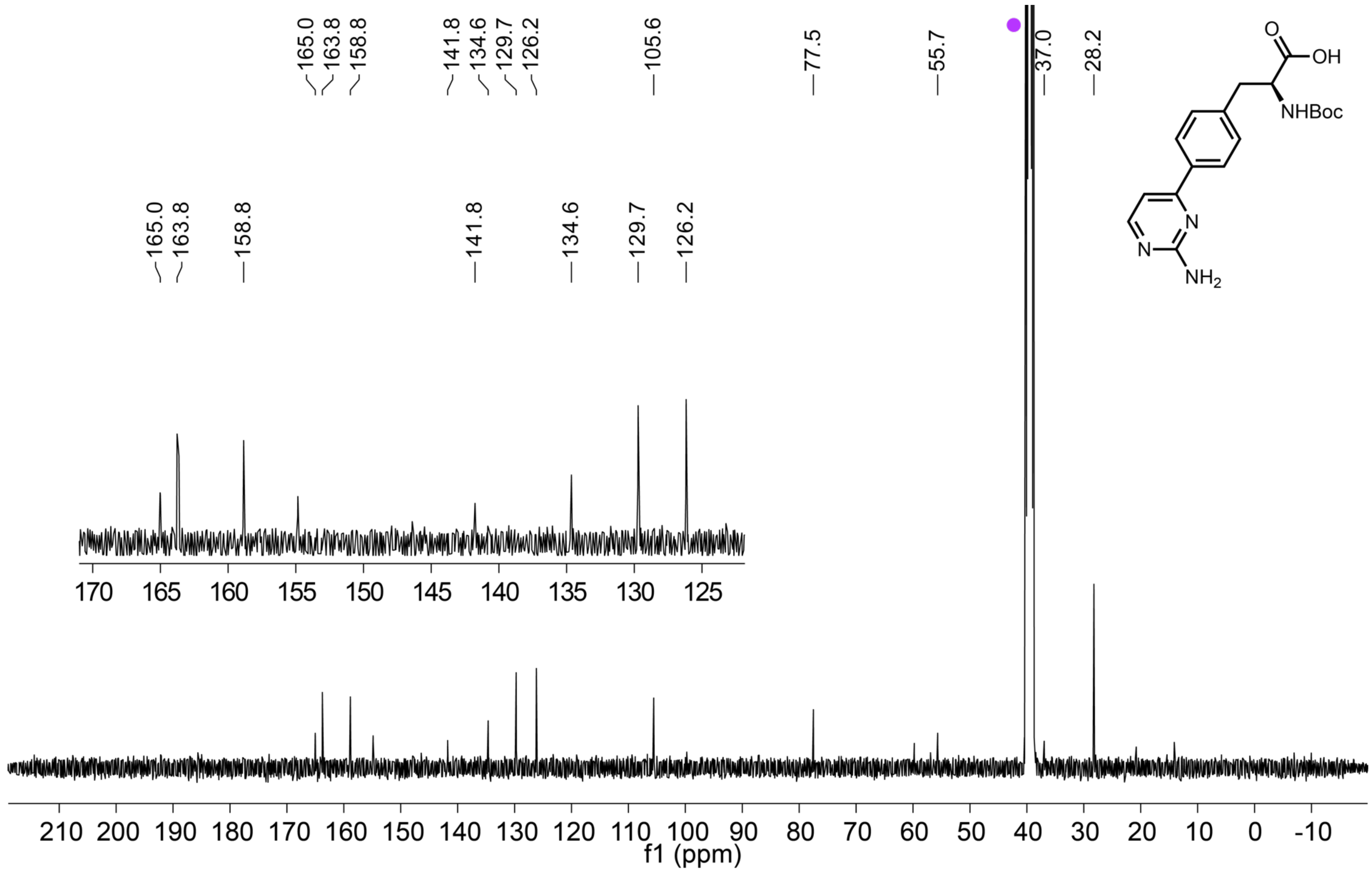

Figure S67. $^{13}C\{^{1}H\}$ NMR (100.67 MHz, DMSO-d_6) spectrum of compound **19**. Deuterated solvent (•).

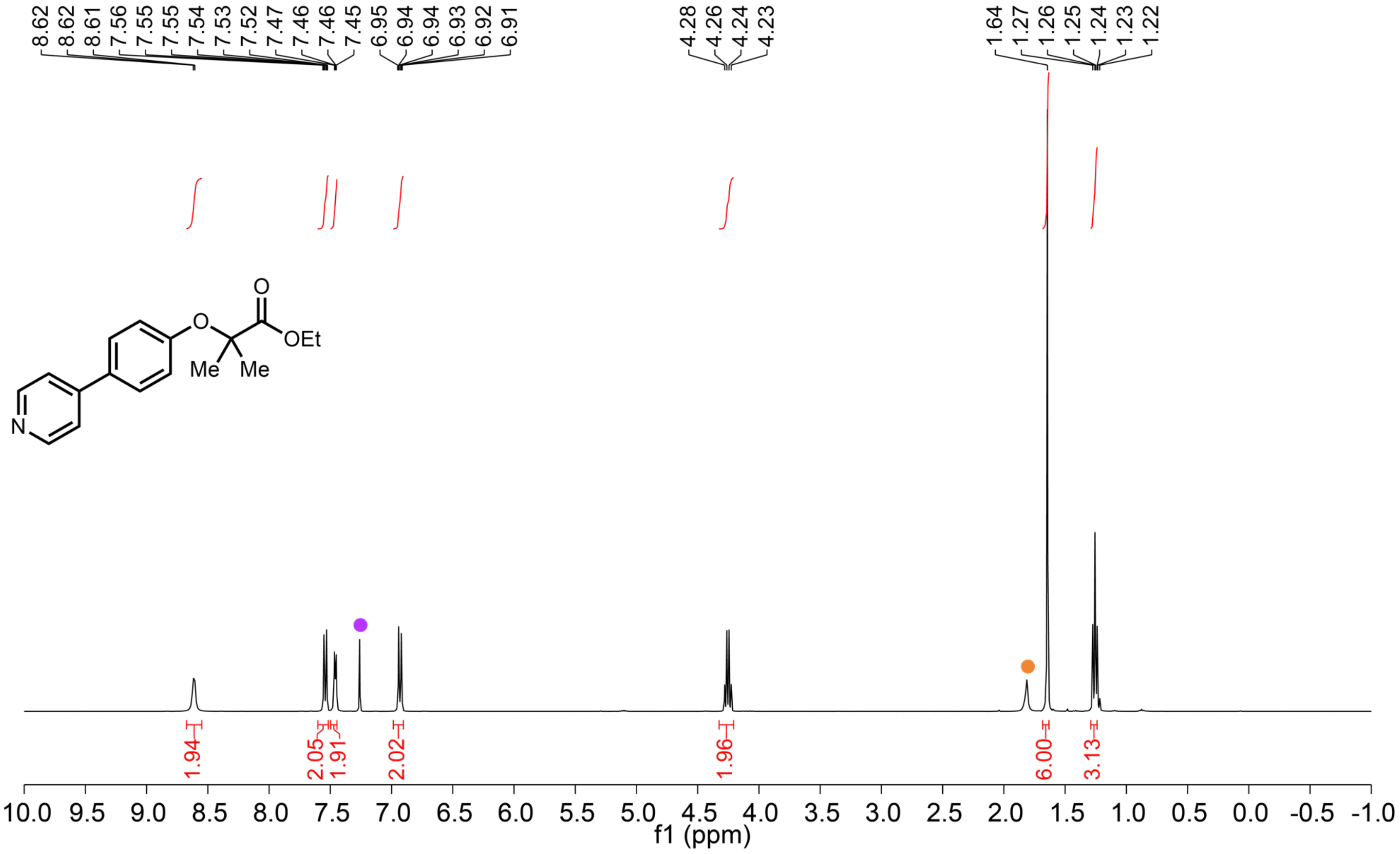

Figure S68. ^{1}H NMR (400.30 MHz, $CDCl_3$) spectrum of compound **20**. H_2O (●) and residual proteo-solvent (●).

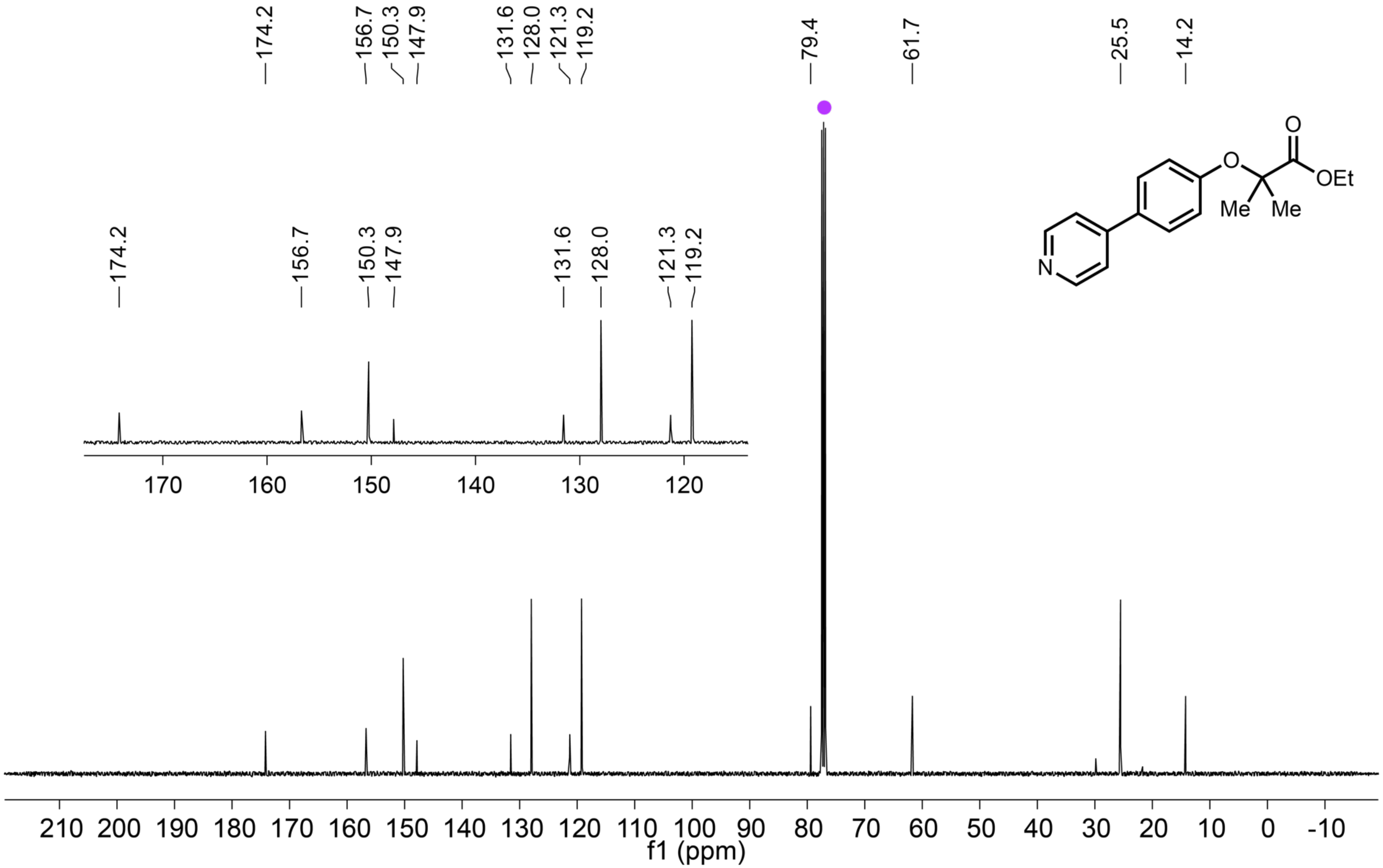

Figure S69. $^{13}C\{^{1}H\}$ NMR (100.67 MHz, $CDCl_3$) spectrum of compound **20**. Deuterated solvent (•).

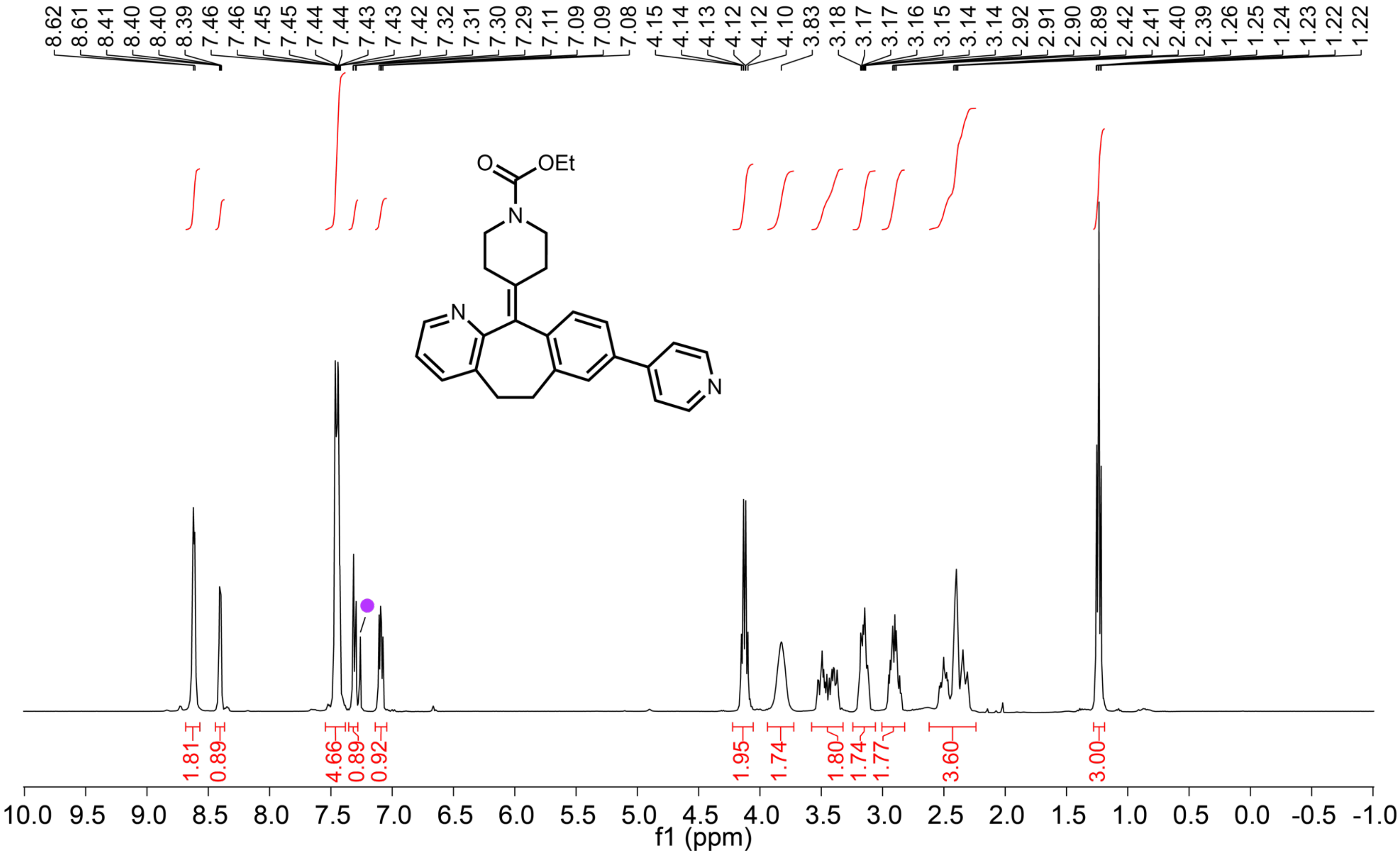

Figure S70. ^{1}H NMR (400.30 MHz, $CDCl_3$) spectrum of compound **21** (from loratadine). Residual proteo-solvent (•).

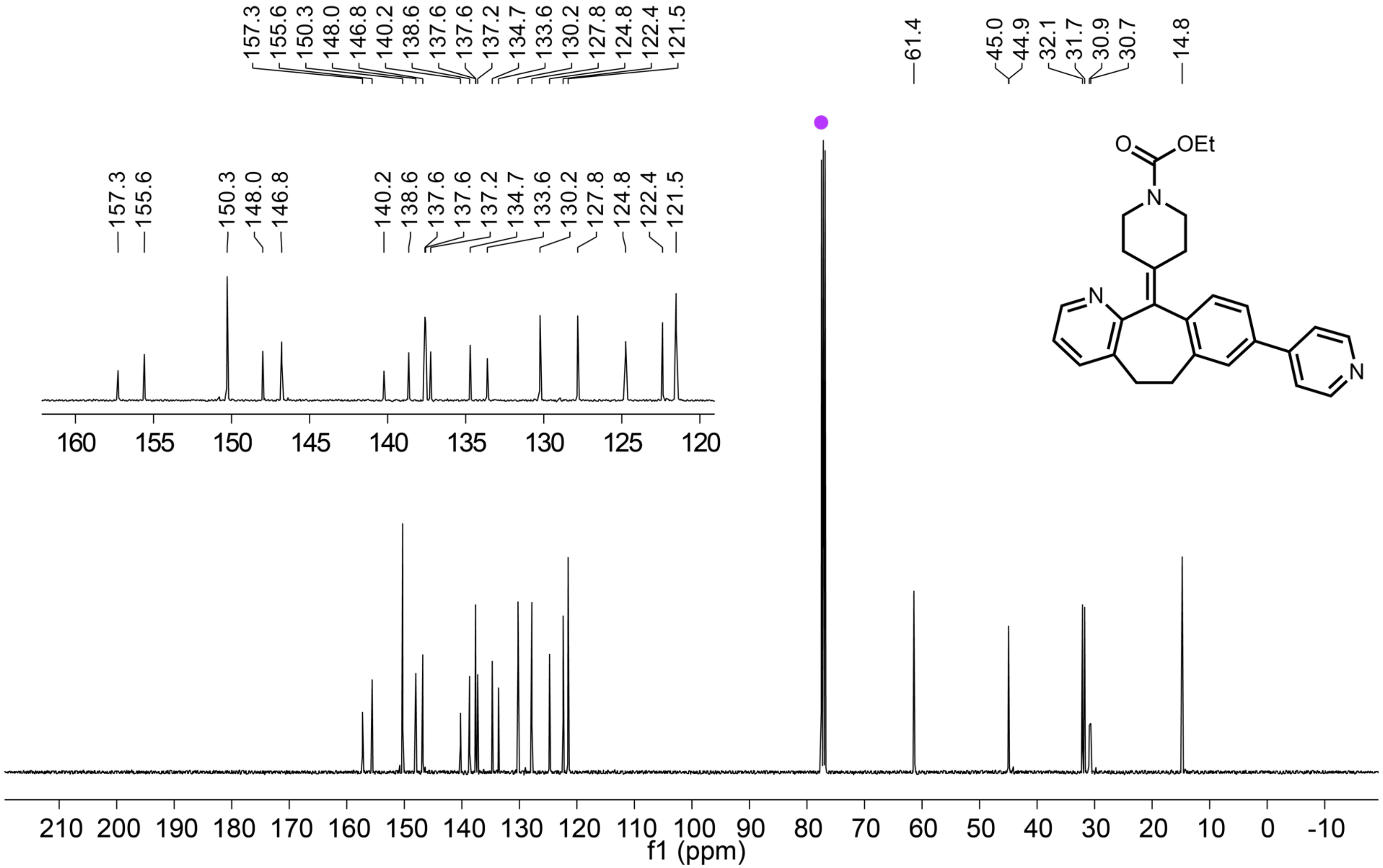

Figure S71. $^{13}C\{^1H\}$ NMR (100.67 MHz, $CDCl_3$) spectrum of compound **21** (from loratadine). Deuterated solvent (•).

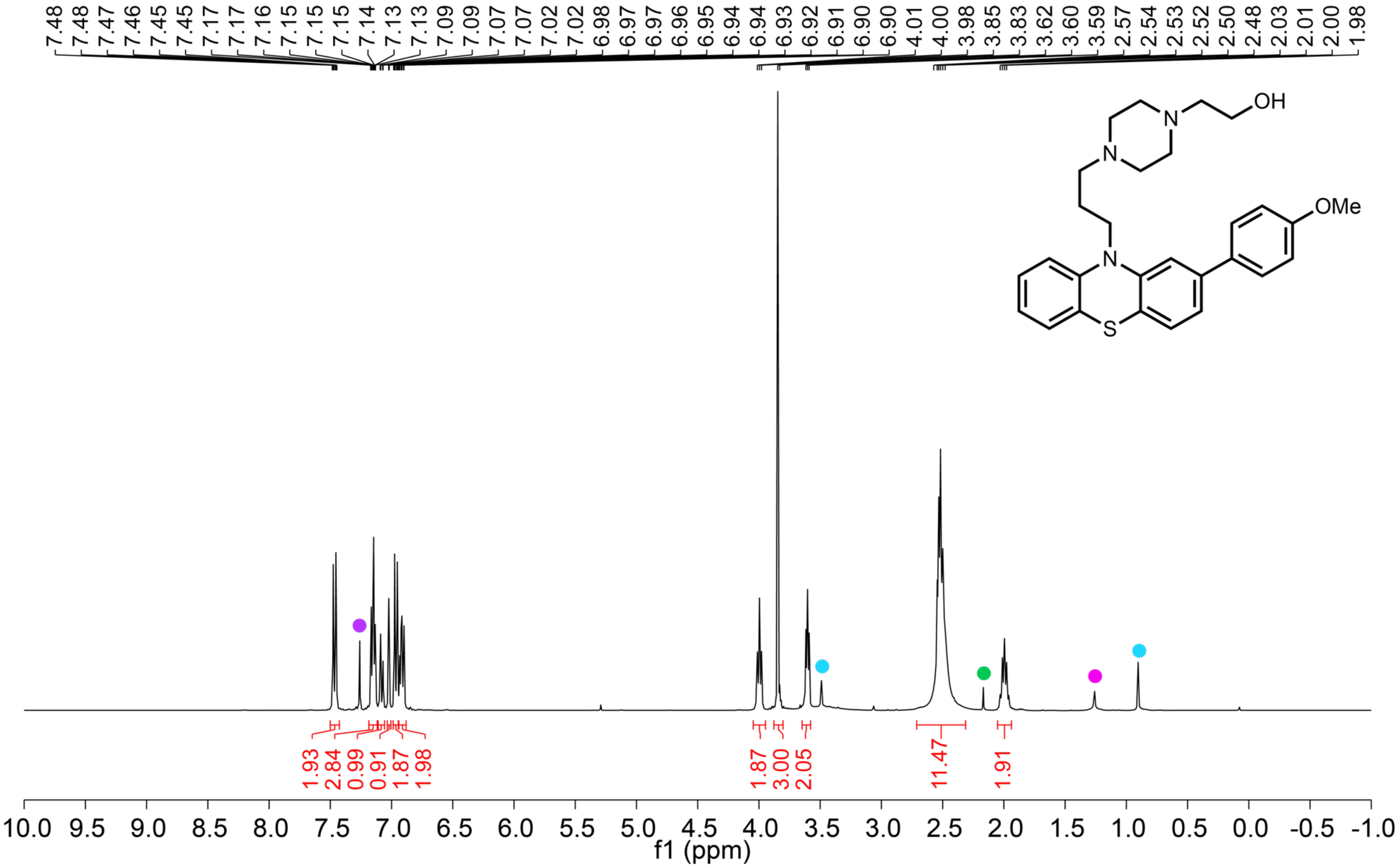

Figure S72. ^{1}H NMR (400.30 MHz, $CDCl_3$) spectrum of compound **22** (from perphenazine). Residual proteo-solvent (●), neopentyl glycol (●), acetone (●) and grease (●). Isolated from the water condition.

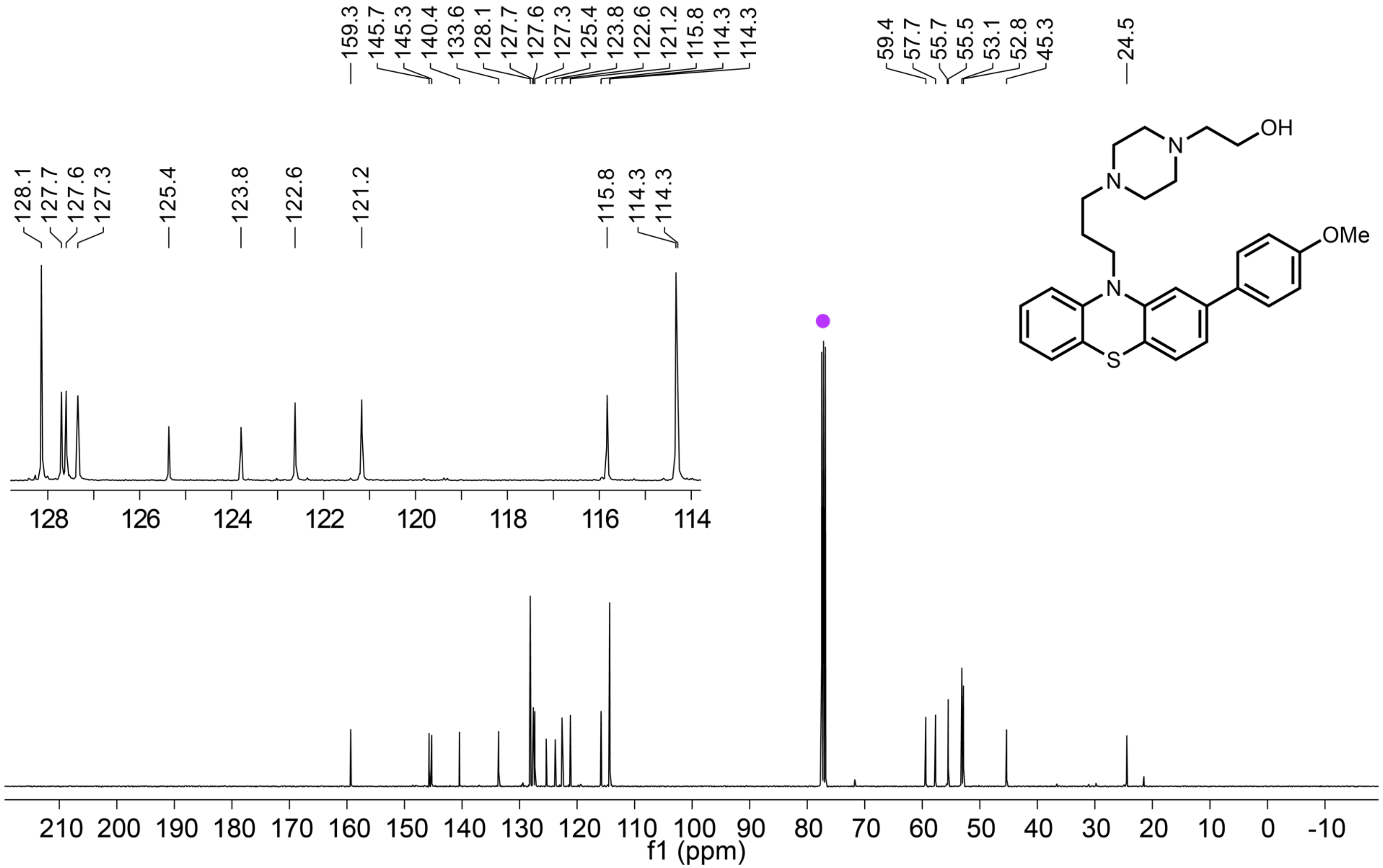

Figure S73. $^{13}C\{^1H\}$ NMR (100.67 MHz, $CDCl_3$) spectrum of compound **22** (from perphenazine). Deuterated solvent (•).

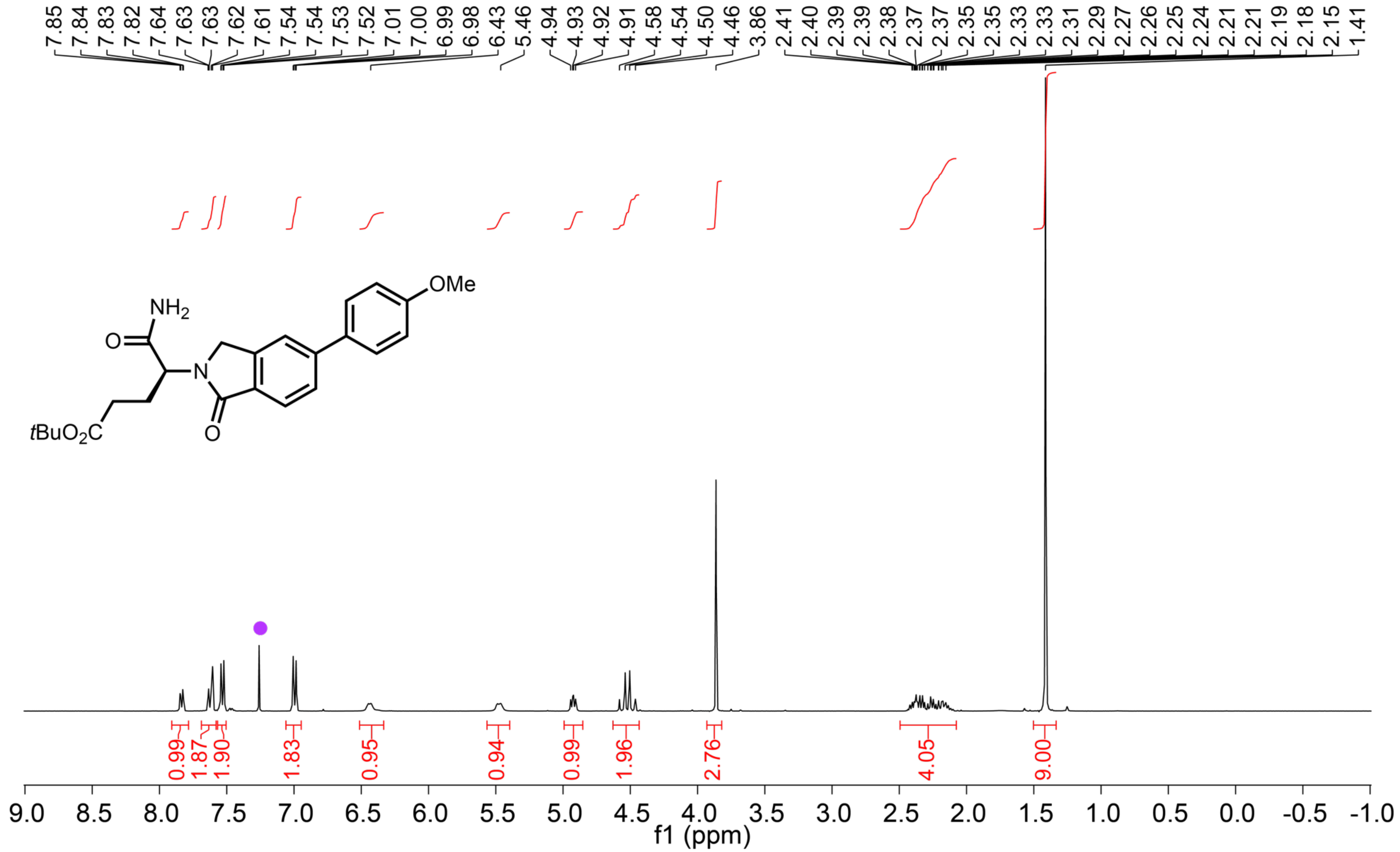

Figure S74. ^{1}H NMR (400.30 MHz, $CDCl_3$) spectrum of compound **23**. Residual proteo-solvent (•) and H_2O (•).

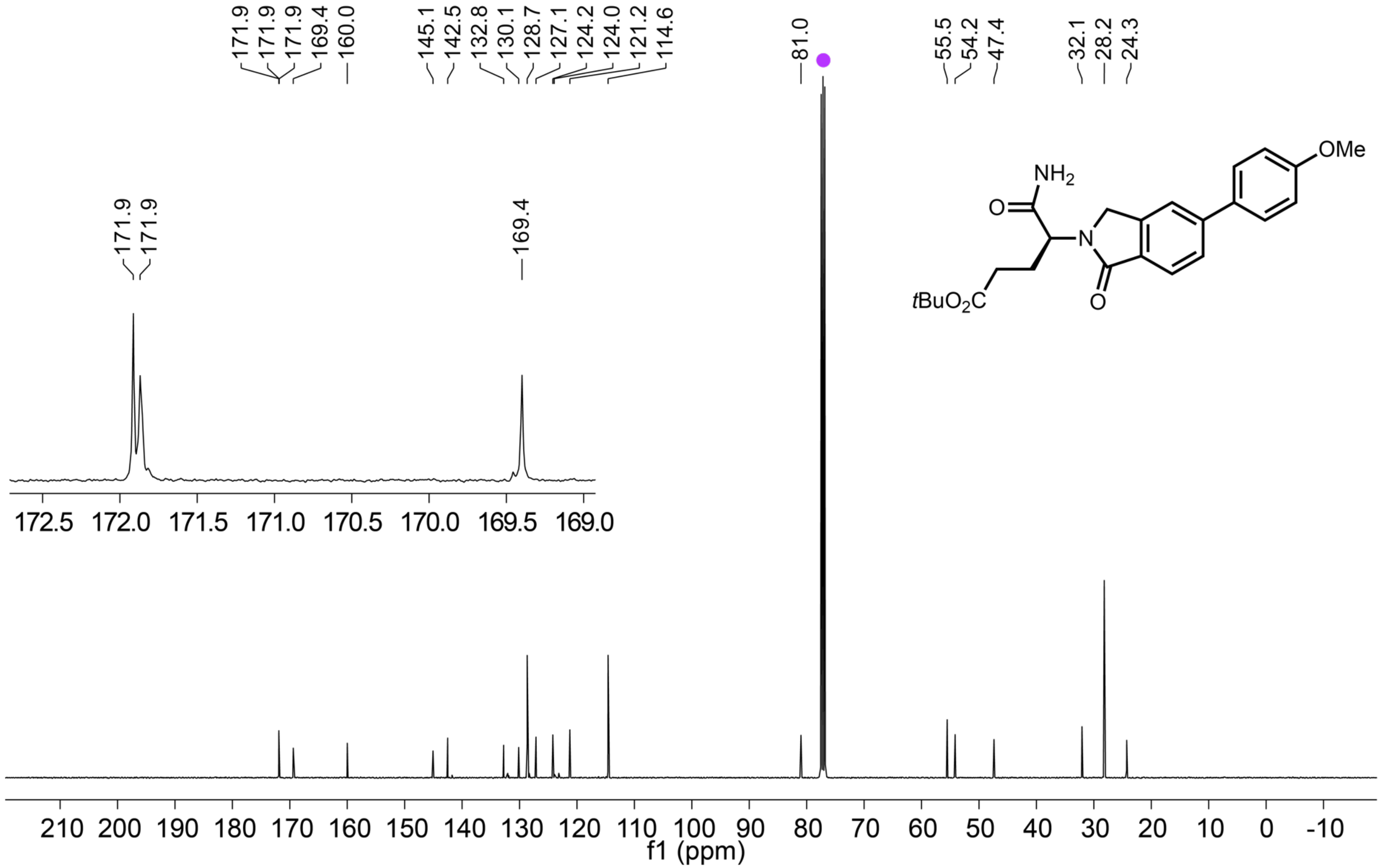

Figure S75. $^{13}C\{^1H\}$ NMR (100.67 MHz, $CDCl_3$) spectrum of compound **23**. Deuterated solvent (•).

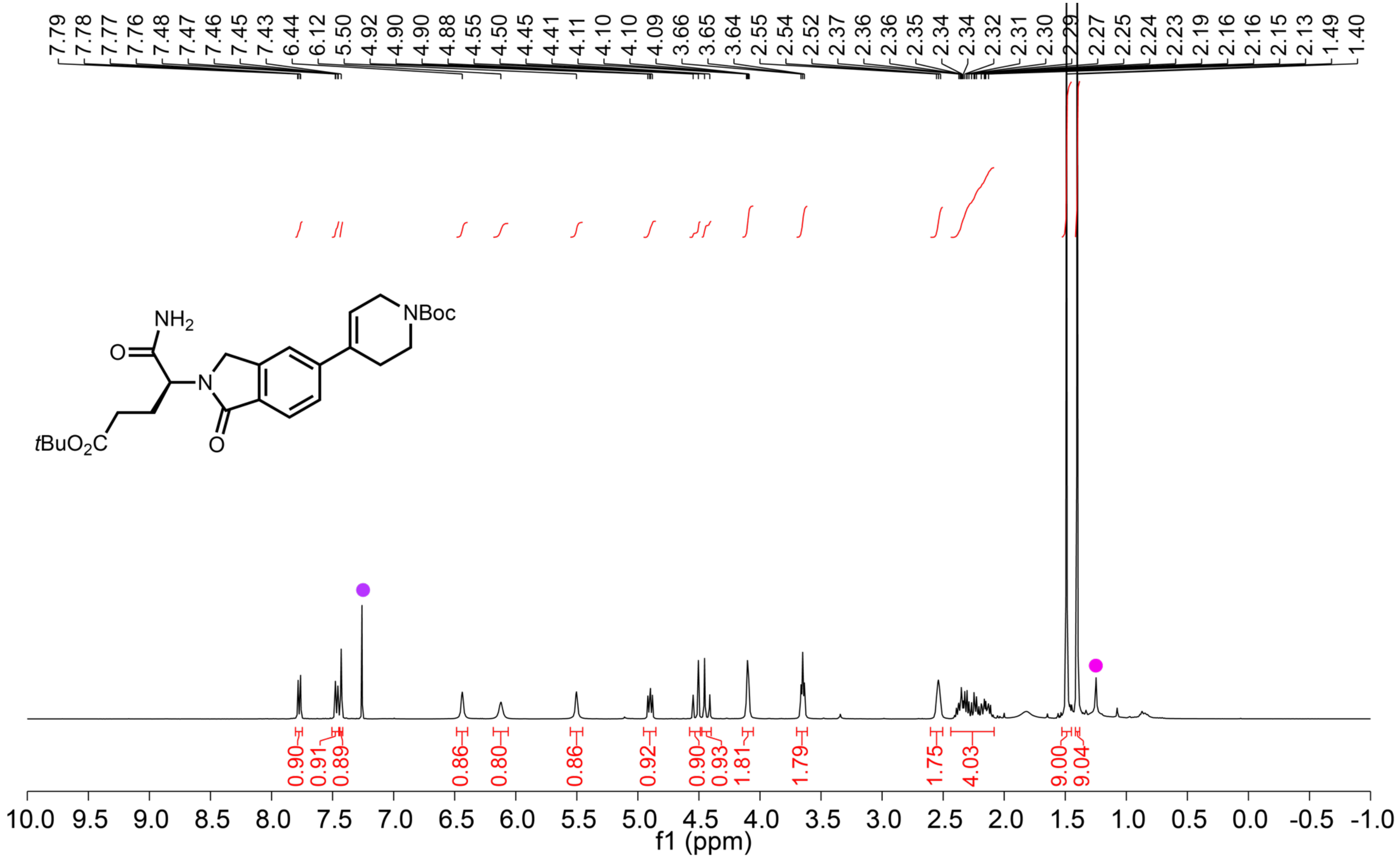

Figure S76. ^{1}H NMR (400.30 MHz, $CDCl_3$) spectrum of compound **24**. Residual proteo-solvent (•) and grease (•).

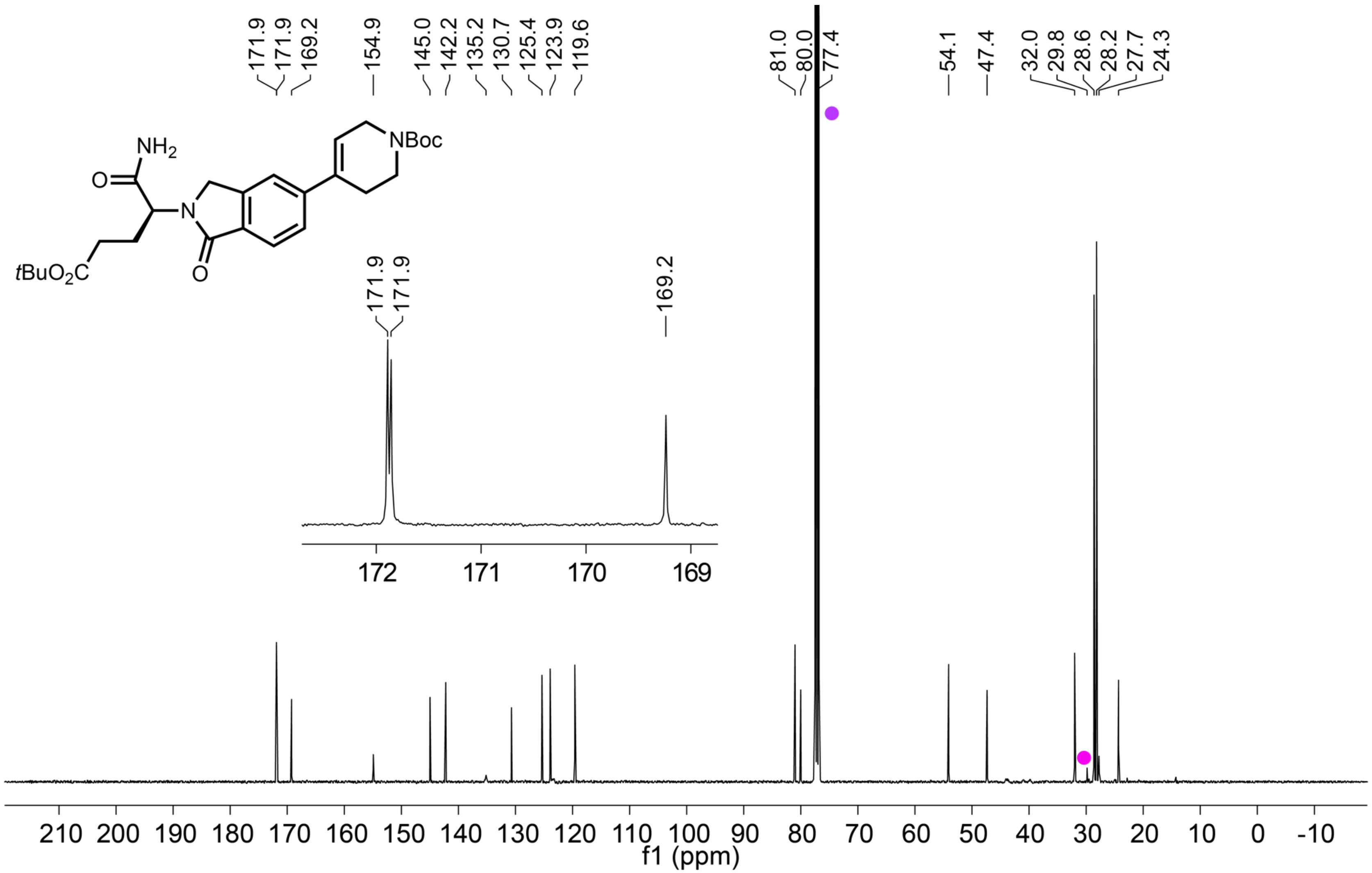

Figure S77. $^{13}C\{^1H\}$ NMR (100.67 MHz, $CDCl_3$) spectrum of compound **24**. Deuterated solvent (•) and grease (•).

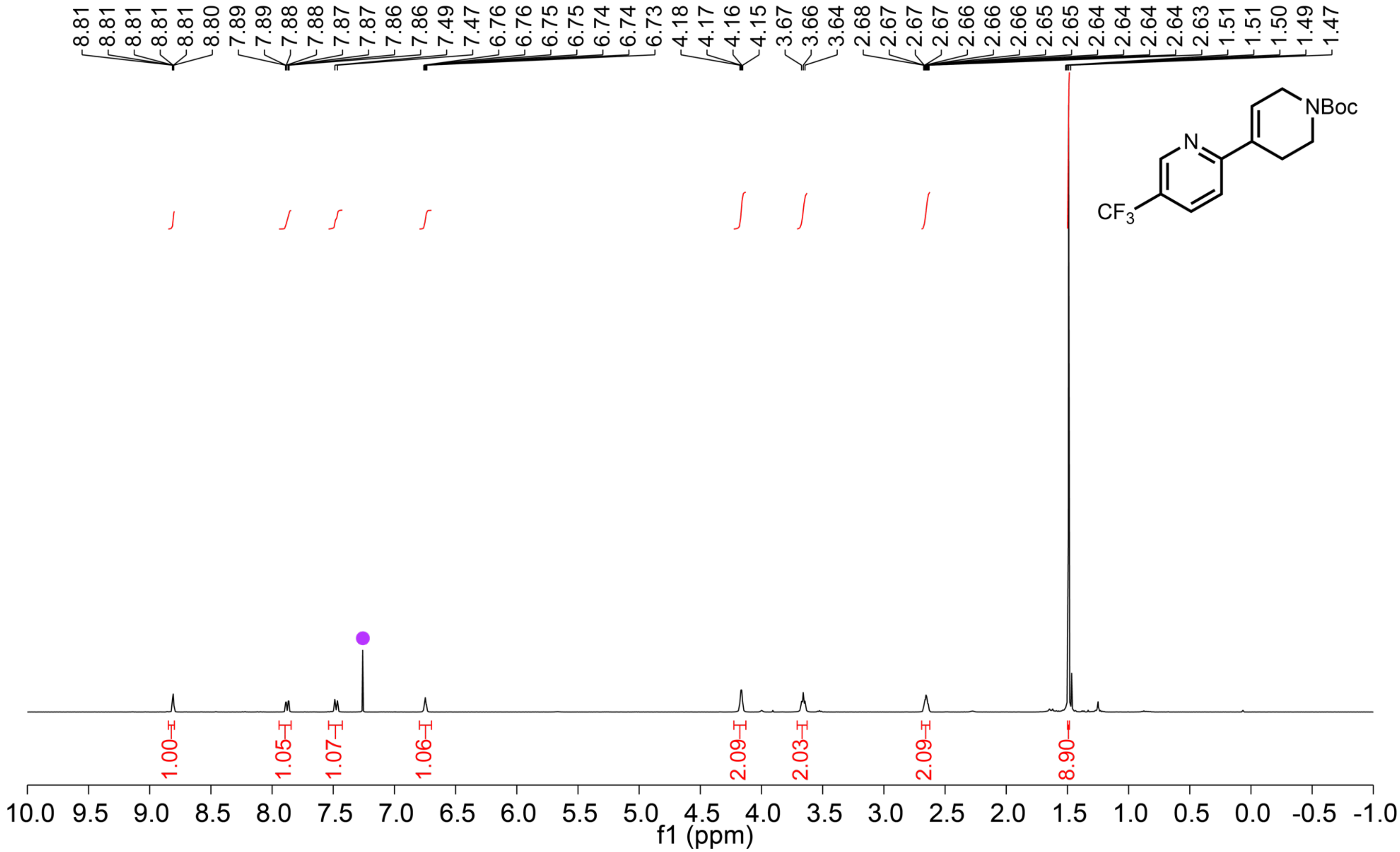

Figure S78. ^{1}H NMR (400.30 MHz, $CDCl_3$) spectrum of compound **25**. Residual proteo-solvent (•).

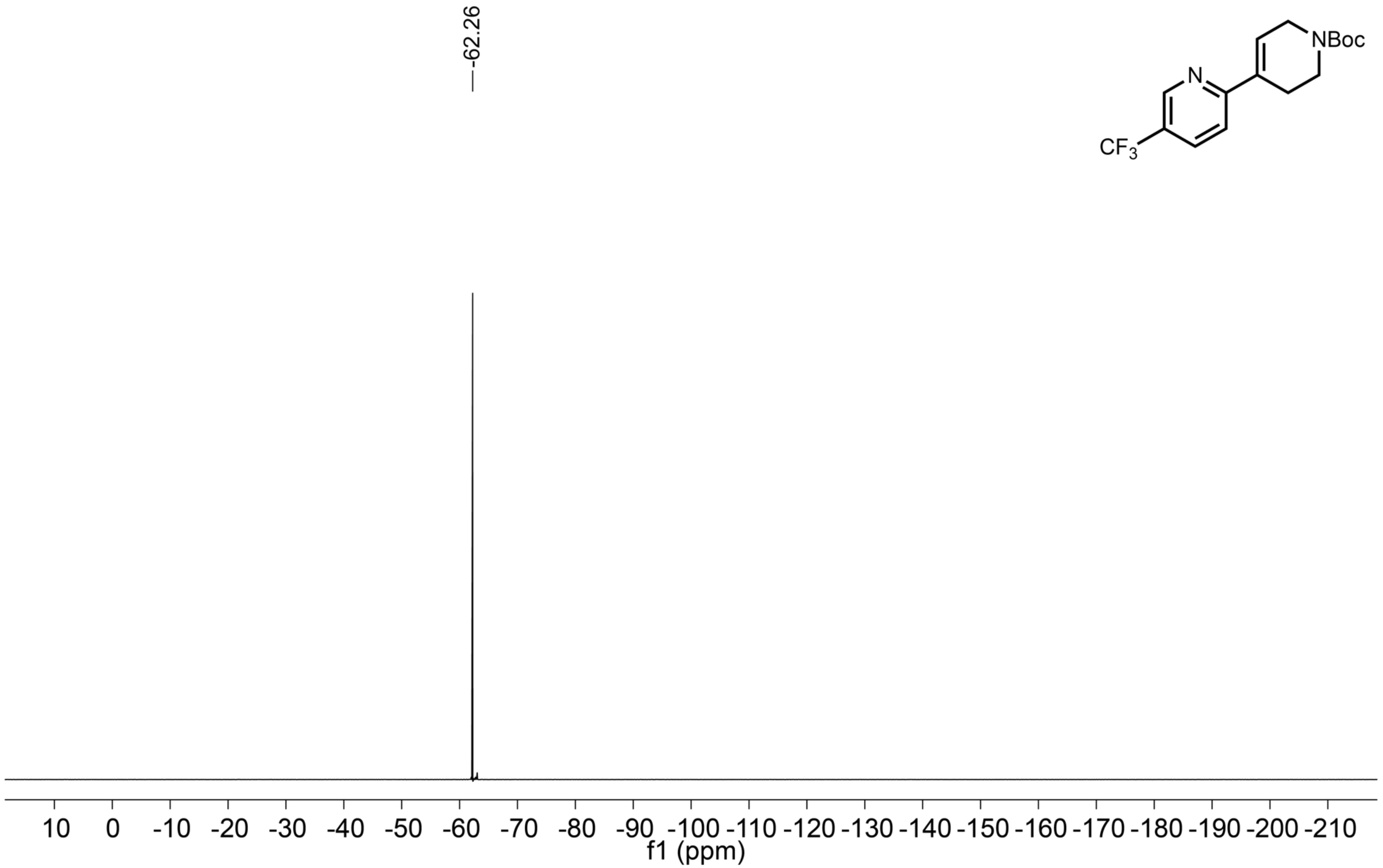

Figure S79. $^{19}F\{^1H\}$ NMR (470.61 MHz, $CDCl_3$) spectrum of compound **25**.

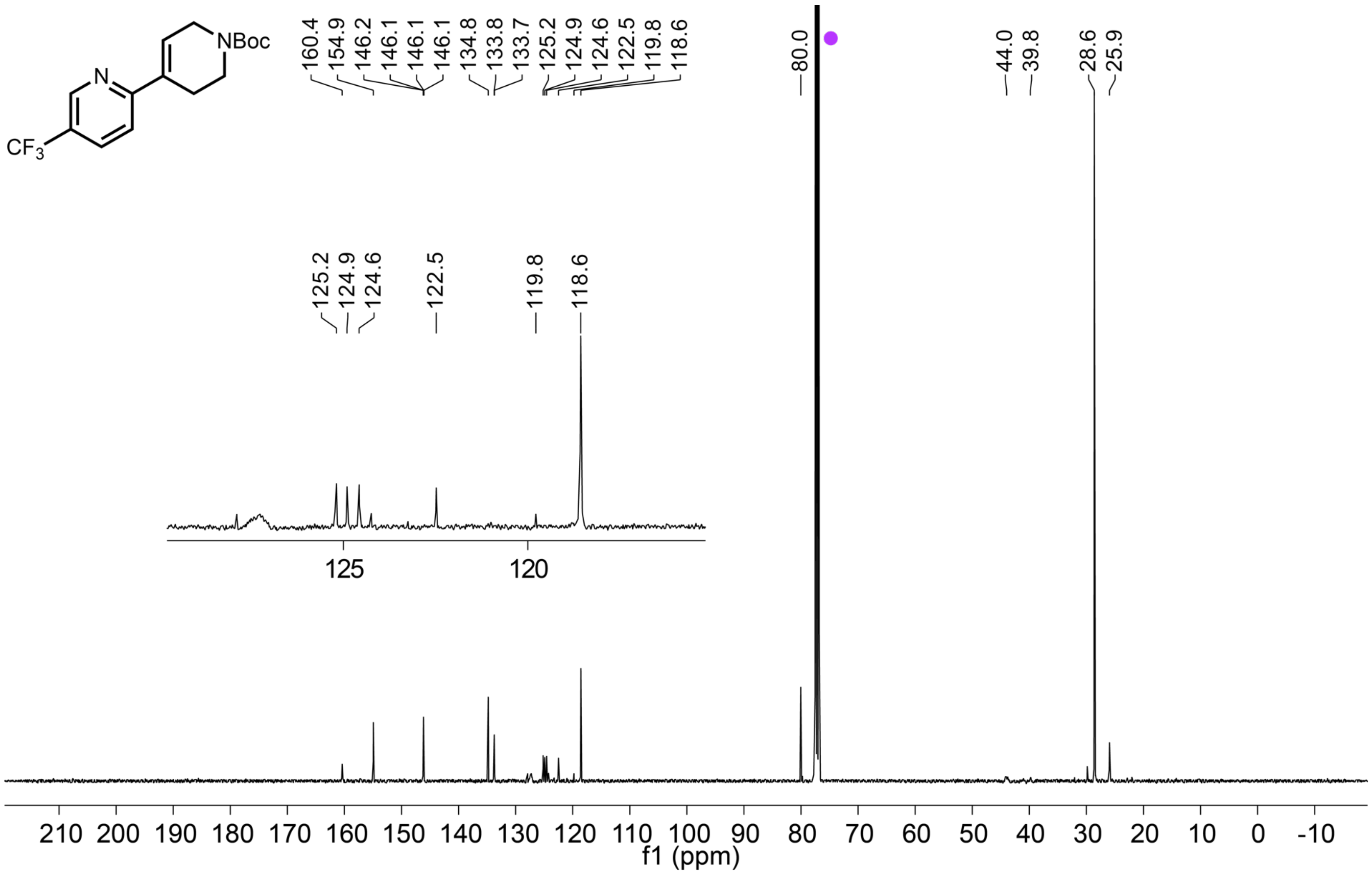

Figure S80. $^{13}C\{^{1}H\}$ NMR (100.67 MHz, $CDCl_3$) spectrum of compound **25**. Deuterated solvent (•).

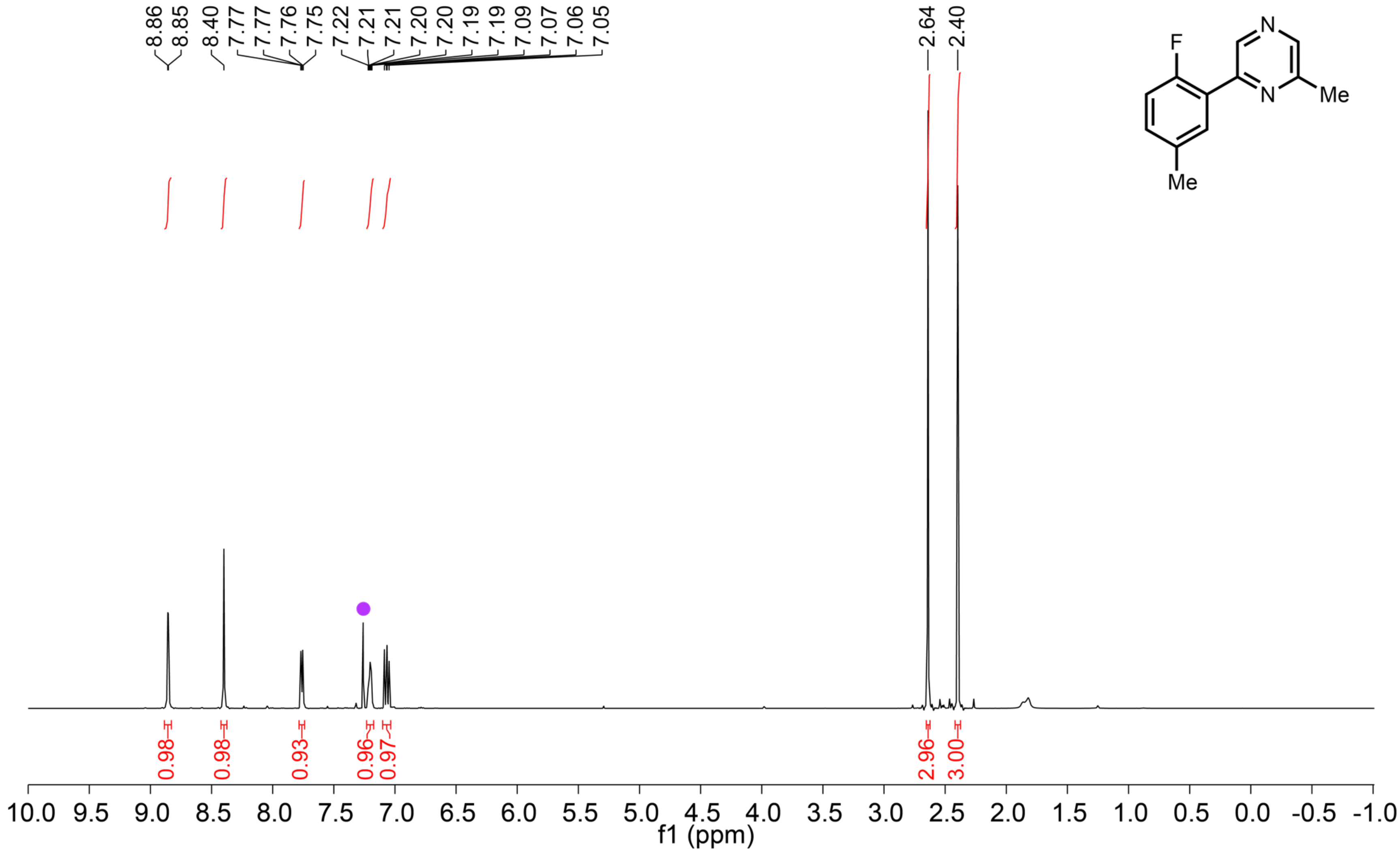

Figure S81. ^{1}H NMR (500.20 MHz, $CDCl_3$) spectrum of compound **26**. Residual proteo-solvent (•).

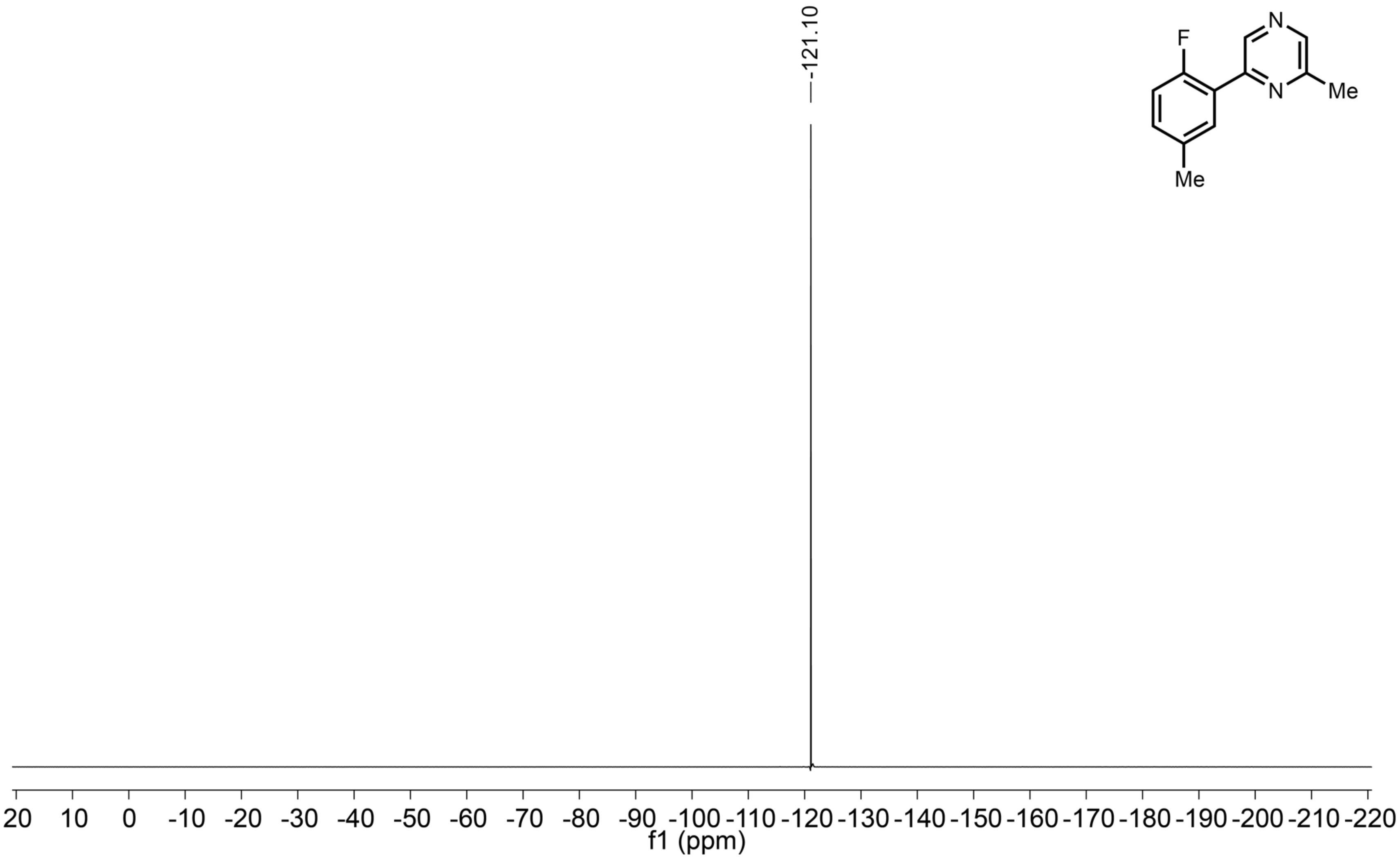

Figure S82. $^{19}F\{^{1}H\}$ NMR (470.61 MHz, $CDCl_3$) spectrum of compound **26**.

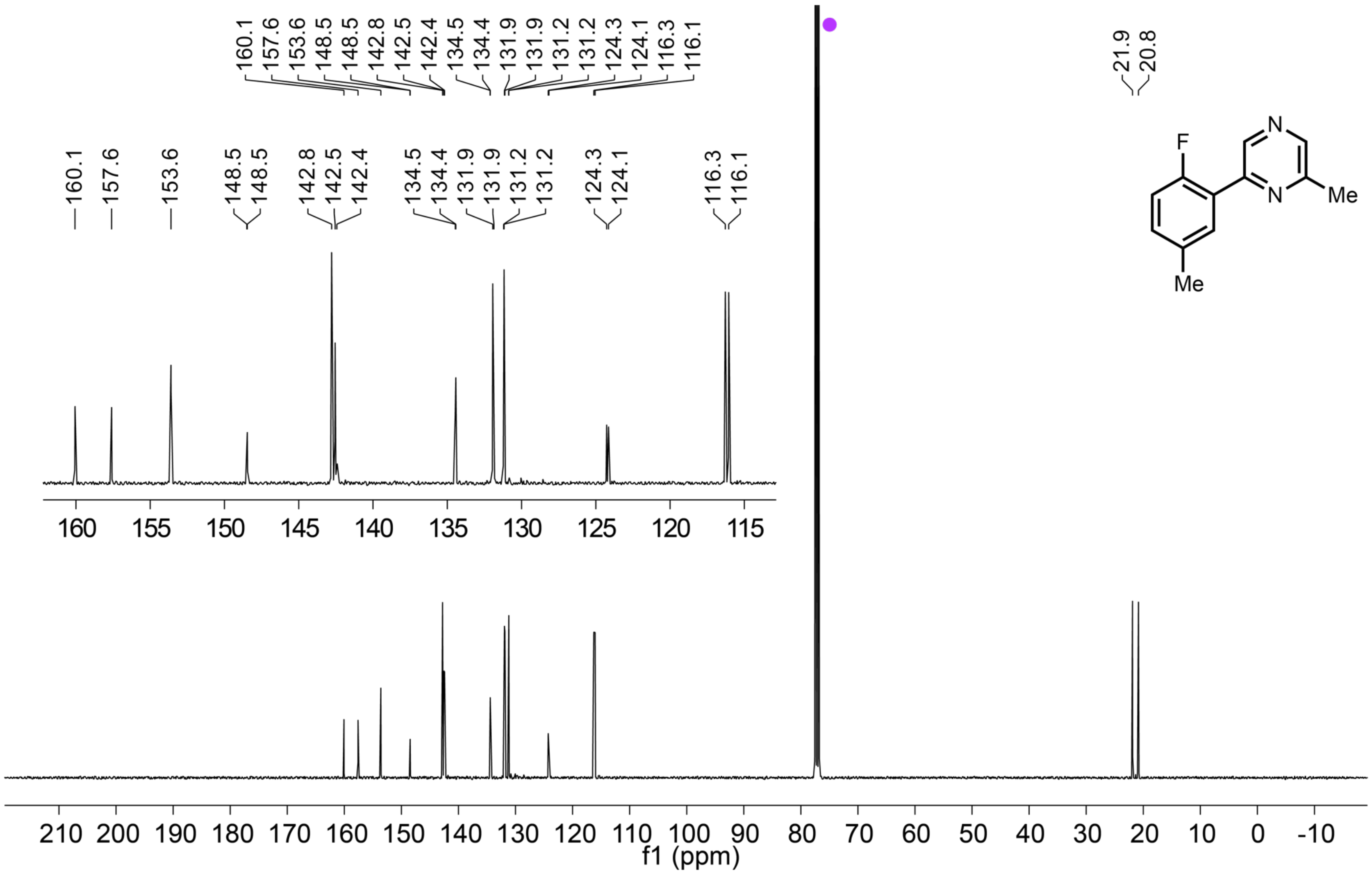

Figure S83. $^{13}C\{^1H\}$ NMR (100.67 MHz, $CDCl_3$) spectrum of compound **26**. Deuterated solvent (•).

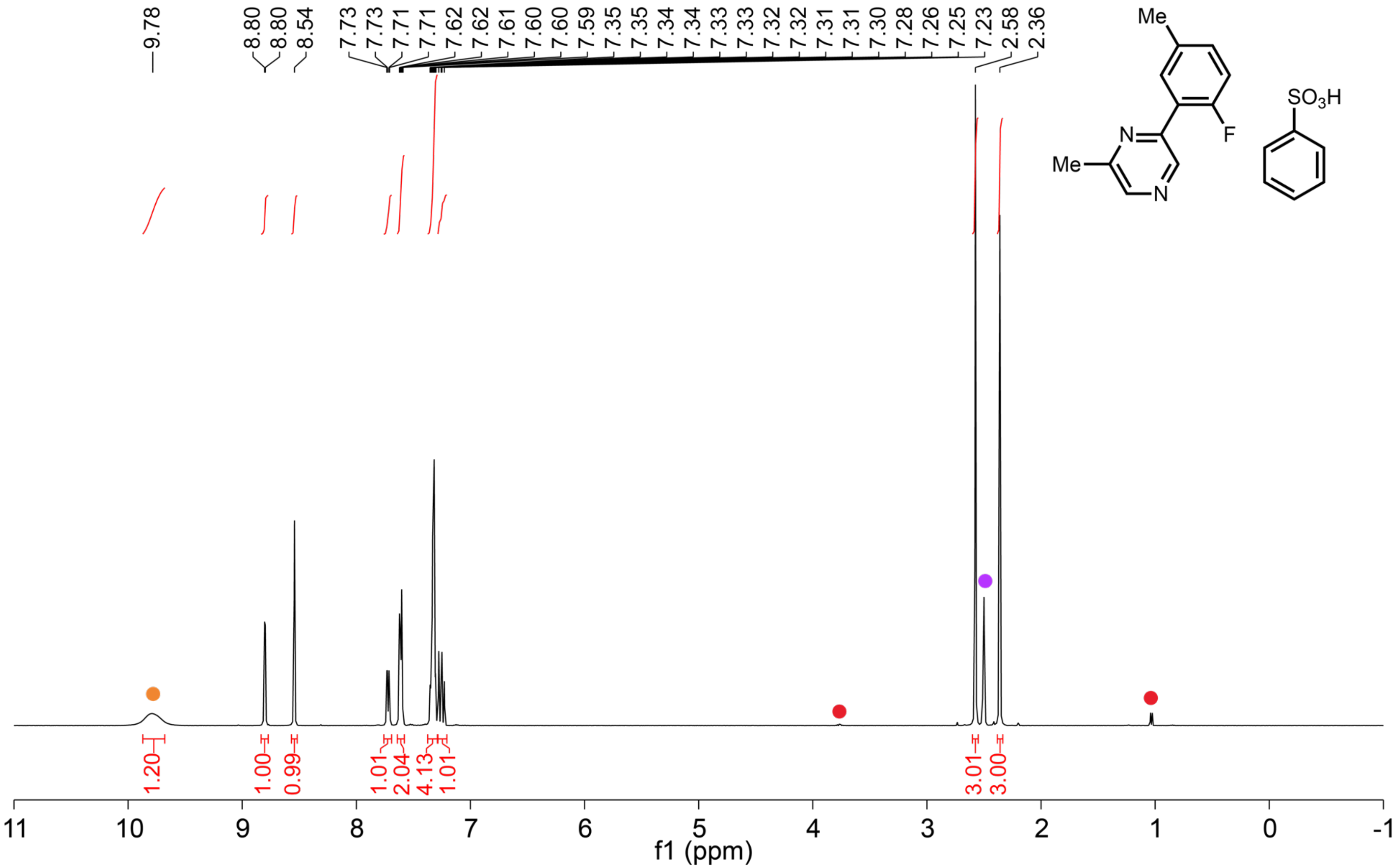

Figure S84. ^{1}H NMR (400.30 MHz, DMSO-*d*$_6$) spectrum of **26**•$C_6H_5SO_3H$. H_2O & SO_3H (●), *i*PrOH (●) and residual proteo-solvent (●).

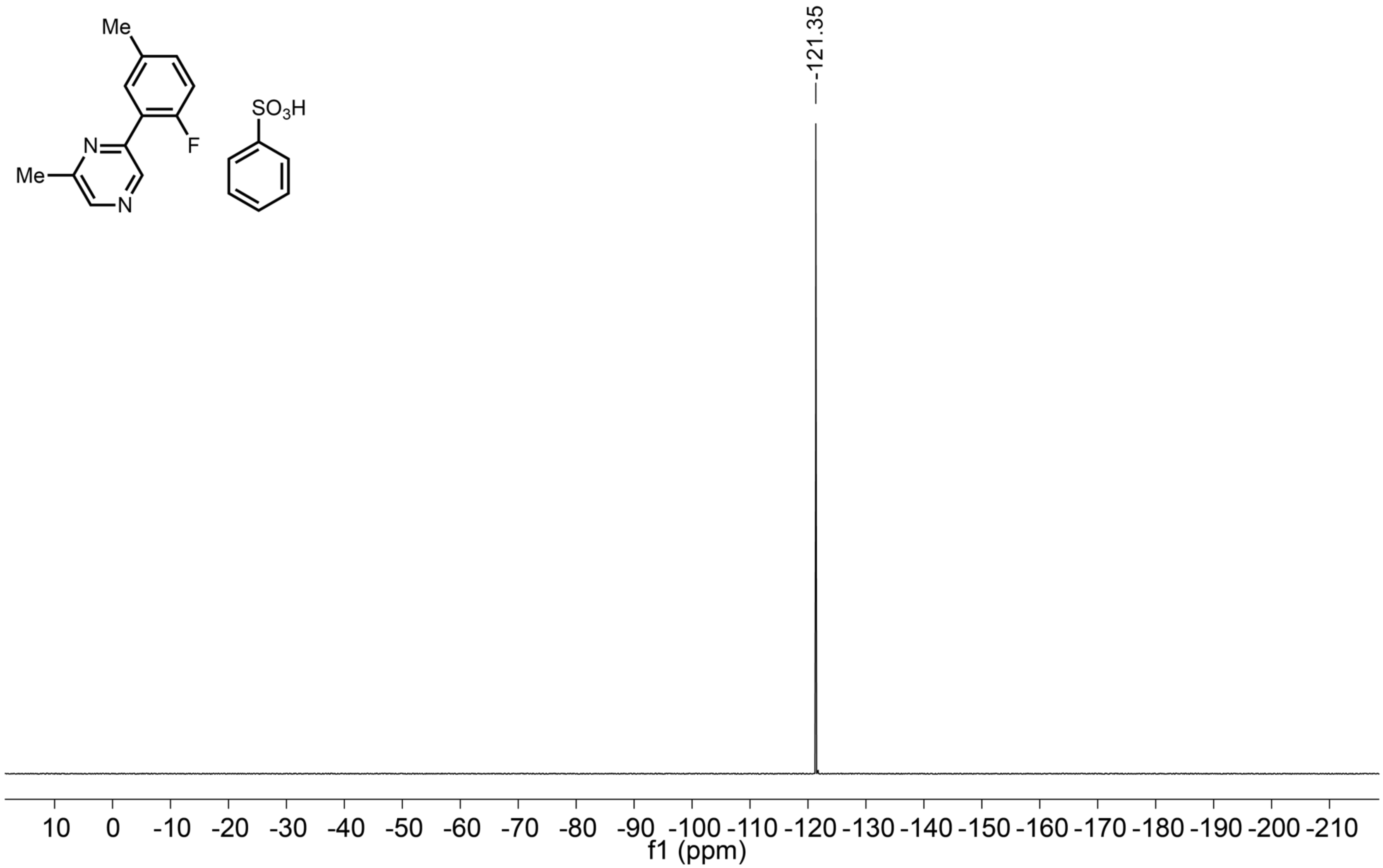

Figure S85. $^{19}F\{^{1}H\}$ NMR (376.46 MHz, DMSO-d_6) spectrum of **26**•$C_6H_5SO_3H$.

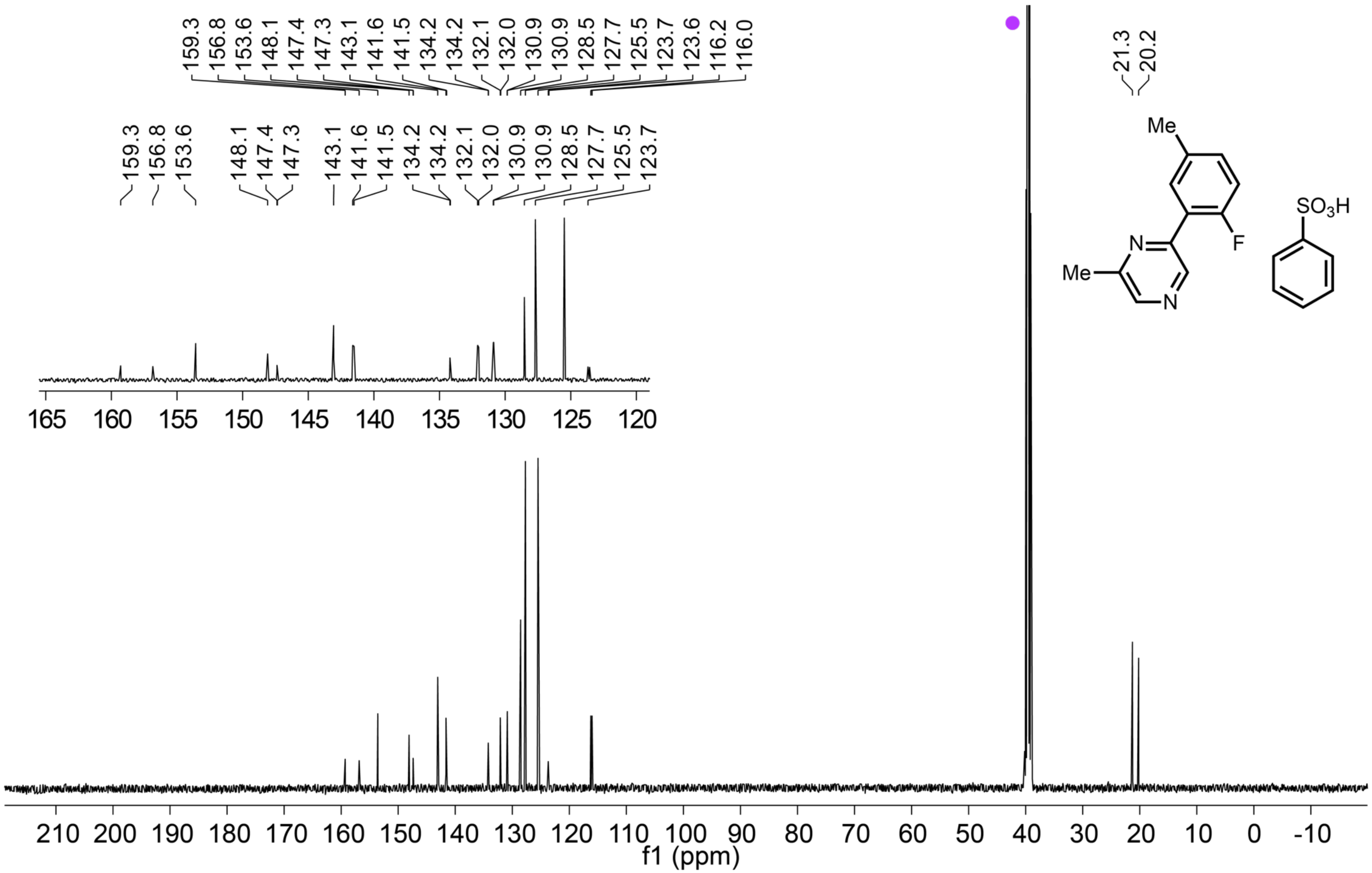

Figure S86. ${}^{13}C\{{}^{1}H\}$ NMR (100.67 MHz, DMSO-d_6) spectrum of **27**•$C_6H_5SO_3H$. Deuterated solvent (•).

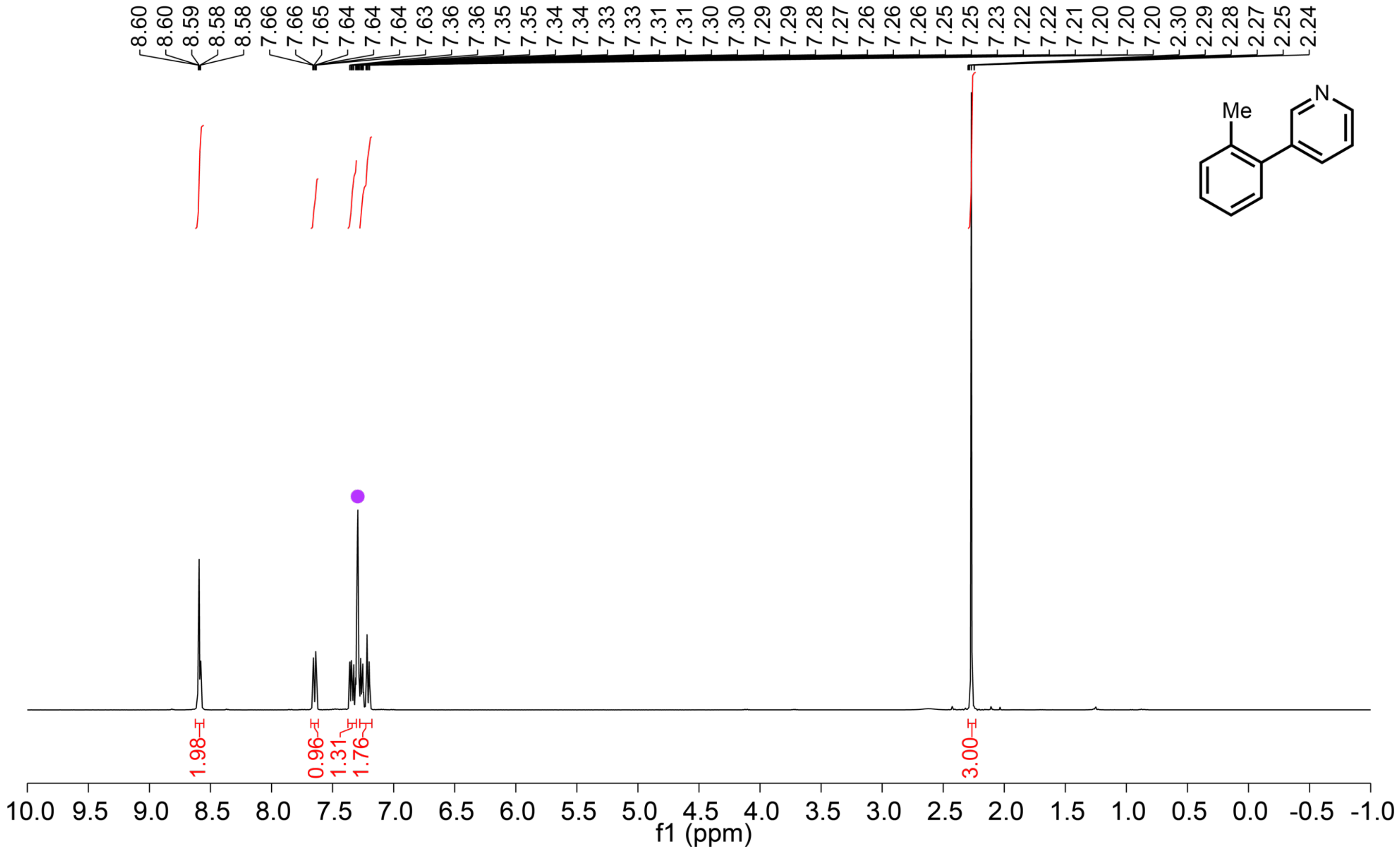

Figure S87. ^{1}H NMR (400.30 MHz, $CDCl_3$) spectrum of compound **S3**. Residual proteo-solvent (•).

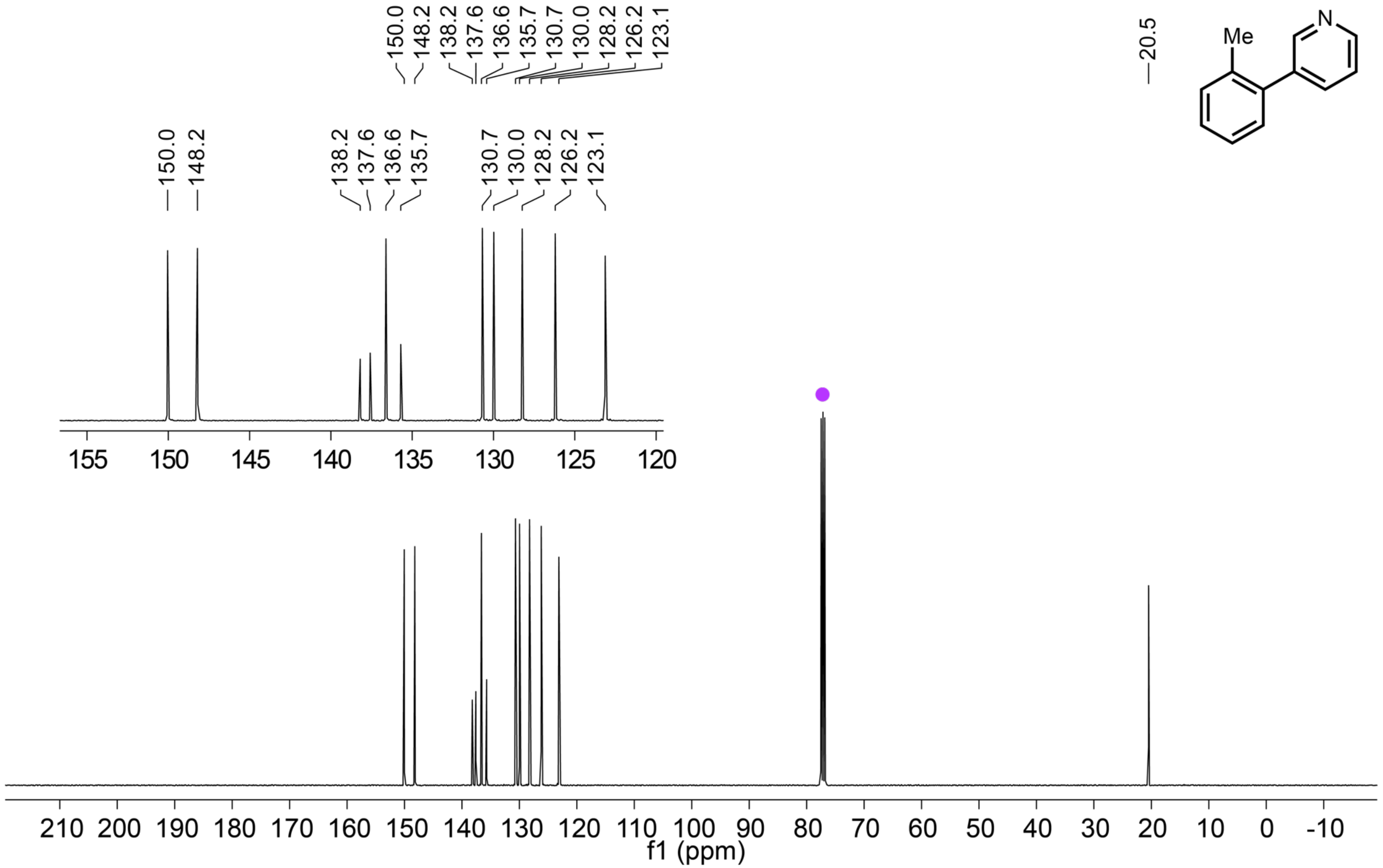

Figure S88. $^{13}C\{^{1}H\}$ NMR (100.67 MHz, $CDCl_3$) spectrum of compound **S3**. Deuterated solvent (•).

8. References

1. Magano, J.; Monfette, S., Development of an Air-Stable, Broadly Applicable Nickel Source for Nickel-Catalyzed Cross-Coupling. *ACS Catal.* **2015,** *5*, 3120-3123.

2. Yang, J.; Neary, M. C.; Diao, T., ProPhos: A Ligand for Promoting Nickel-Catalyzed Suzuki-Miyaura Coupling Inspired by Mechanistic Insights into Transmetalation. *J. Am. Chem. Soc.* **2024,** *146*, 6360-6368.

3. Klabunde, U.; Mulhaupt, R.; Herskovitz, T.; Janowicz, A. H.; Calabrese, J.; Ittel, S. D., Ethylene homopolymerization with P, O-chelated nickel catalysts. *J. Polym. Sci. Part A: Polym. Chem.* **1987,** *25*, 1989-2003.

4. Soula, R.; Broyer, J. P.; Llauro, M. F.; Tomov, A.; Spitz, R.; Claverie, J.; Drujon, X.; Malinge, J.; Saudemont, T., Very Active Neutral P,O-Chelated Nickel Catalysts for Ethylene Polymerization. *Macromolecules* **2001,** *34*, 2438-2442.

5. Hood, T. M.; Chaplin, A. B., Synthesis and Reactivity of Iridium Complexes of a Macrocyclic PNP Pincer Ligand. *Dalton Trans.* **2021,** *50*, 2472-2482.

6. Carrow, B. P.; Hartwig, J. F., Distinguishing Between Pathways for Transmetalation in Suzuki-Miyaura Reactions. *J. Am. Chem. Soc.* **2011,** *133*, 2116-2119.

7. Goldfogel, M. J.; Guo, X.; Meléndez Matos, J. L.; Gurak, J. A., Jr.; Joannou, M. V.; Moffat, W. B.; Simmons, E. M.; Wisniewski, S. R., Advancing Base-Metal Catalysis: Development of a Screening Method for Nickel-Catalyzed Suzuki–Miyaura Reactions of Pharmaceutically Relevant Heterocycles. *Org. Process Res. Dev.* **2022,** *26*, 785-794.

8. Haibach, M. C.; Ickes, A. R.; Tcyrulnikov, S.; Shekhar, S.; Monfette, S.; Swiatowiec, R.; Kotecki, B. J.; Wang, J.; Wall, A. L.; Henry, R. F.; Hansen, E. C., Enabling Suzuki–Miyaura Coupling of Lewis-Basic Arylboronic Esters with a Nonprecious Metal Catalyst. *Chem. Sci.* **2022,** *13*, 12906-12912.

9. John, M. E.; Nutt, M. J.; Offer, J. E.; Duczynski, J. A.; Yamazaki, K.; Miura, T.; Moggach, S. A.; Koutsantonis, G. A.; Dorta, R.; Stewart, S. G., Efficient Nickel Precatalysts for Suzuki-Miyaura Cross-Coupling of Aryl Chlorides and Arylboronic Acids Under Mild Conditions. *Angew. Chem. Int. Ed.* **2025,** *64*, e202504108.

10. Saeb, R.; Roh, B.; Cornella, J., “Naked Nickel”-Catalyzed Heteroaryl–Heteroaryl Suzuki–Miyaura Coupling. *Angew. Chem. Int. Ed.* **2025,** *64*, e202424051.

11. Shields, J. D.; Gray, E. E.; Doyle, A. G., A Modular, Air-Stable Nickel Precatalyst. *Org. Lett.* **2015,** *17*, 2166-2169.

12. Tran, V. T.; Li, Z.-Q.; Apolinar, O.; Derosa, J.; Joannou, M. V.; Wisniewski, S. R.; Eastgate, M. D.; Engle, K. M., Ni(COD)(DQ): An Air-Stable 18-Electron Nickel(0)–Olefin Precatalyst. *Angew. Chem. Int. Ed.* **2020,** *59*, 7409-7413.

13. Tran, V. T.; Kim, N.; Rubel, C. Z.; Wu, X. Y.; Kang, T. H.; Jankins, T. C.; Li, Z. Q.; Joannou, M. V.; Ayers, S.; Gembicky, M.; Bailey, J.; Sturgell, E. J.; Sanchez, B. B.; Chen, J. S.; Lin, S.; Eastgate, M. D.; Wisniewski, S. R.; Engle, K. M., Structurally Diverse Bench-Stable Nickel(0) Pre-Catalysts: A Practical Toolkit for In Situ Ligation Protocols. *Angew. Chem. Int. Ed.* **2023,** *62*, 10.

14. Nattmann, L.; Saeb, R.; Nöthling, N.; Cornella, J., An Air-Stable Binary Ni(0)–Olefin Catalyst. *Nat. Catal.* **2020,** *3*, 6-13.

15. Rosen, B. M.; Quasdorf, K. W.; Wilson, D. A.; Zhang, N.; Resmerita, A.-M.; Garg, N. K.; Percec, V., Nickel-Catalyzed Cross-Couplings Involving Carbon−Oxygen Bonds. *Chem. Rev.* **2011,** *111*, 1346-1416.

16. Hazari, N.; Melvin, P. R.; Beromi, M. M., Well-Defined Nickel and Palladium Precatalysts for Cross-Coupling. *Nat. Rev. Chem.* **2017,** *1*, 0025.

17. Borowski, J. E.; Newman-Stonebraker, S. H.; Doyle, A. G., Comparison of Monophosphine and Bisphosphine Precatalysts for Ni-Catalyzed Suzuki–Miyaura Cross-Coupling: Understanding the Role of the Ligation State in Catalysis. *ACS Catal.* **2023,** *13*, 7966-7977.

18. Sawatzky, R. S.; Stradiotto, M., (DPEPhos)Ni(mesityl)Br: An Air-Stable Pre-Catalyst for Challenging Suzuki–Miyaura Cross-Couplings Leading to Unsymmetrical Biheteroaryls. *Synlett* **2018,** *29*, 799-804.

19. Ramgren, S. D.; Hie, L.; Ye, Y.; Garg, N. K., Nickel-Catalyzed Suzuki–Miyaura Couplings in Green Solvents. *Org. Lett.* **2013,** *15*, 3950-3953.

20. Ge, S.; Hartwig, J. F., Highly Reactive, Single-Component Nickel Catalyst Precursor for Suzuki–Miyuara Cross-Coupling of Heteroaryl Boronic Acids with Heteroaryl Halides. *Angew. Chem. Int. Ed.* **2012,** *51*, 12837-12841.

21. Guard, L. M.; Mohadjer Beromi, M.; Brudvig, G. W.; Hazari, N.; Vinyard, D. J., Comparison of dppf-Supported Nickel Precatalysts for the Suzuki–Miyaura Reaction: The Observation and Activity of Nickel(I). *Angew. Chem. Int. Ed.* **2015,** *54*, 13352-13356.

22. West, M. J.; Watson, A. J. B., Ni vs. Pd in Suzuki–Miyaura Sp^2–Sp^2 Cross-Coupling: A Head-to-Head Study in a Comparable Precatalyst/Ligand System. *Org. Biomol. Chem.* **2019,** *17*, 5055-5059.

23. Guo, X.; Dang, H.; Wisniewski, S. R.; Simmons, E. M., Nickel-Catalyzed Suzuki–Miyaura Cross-Coupling Facilitated by a Weak Amine Base with Water as a Cosolvent. *Organometallics* **2022,** *41*, 1269-1274.

24. Ando, S.; Matsunaga, H.; Ishizuka, T., An N-Heterocyclic Carbene-Nickel Half-Sandwich Complex as a Precatalyst for Suzuki–Miyaura Coupling of Aryl/Heteroaryl Halides with Aryl/Heteroarylboronic Acids. *J. Org. Chem.* **2017,** *82*, 1266-1272.

25. Ohtsuki, A.; Yanagisawa, K.; Furukawa, T.; Tobisu, M.; Chatani, N., Nickel/N-Heterocyclic Carbene-Catalyzed Suzuki–Miyaura Type Cross-Coupling of Aryl Carbamates. *J. Org. Chem.* **2016,** *81*, 9409-9414.

26. Martín, L.; Molins, E.; Vallribera, A., Nickel and Palladium Nanocomposite Carbon Aerogels as Recyclable Catalysts for Suzuki–Miyaura Reaction under Eerobic and Phosphine-Free Conditions in Water. *Tetrahedron* **2012,** *68*, 6517-6520.

27. Handa, S.; Slack, E. D.; Lipshutz, B. H., Nanonickel-Catalyzed Suzuki–Miyaura Cross-Couplings in Water. *Angew. Chem. Int. Ed.* **2015,** *54*, 11994-11998.

28. Dander, J. E.; Giroud, M.; Racine, S.; Darzi, E. R.; Alvizo, O.; Entwistle, D.; Garg, N. K., Chemoenzymatic Conversion of Amides to Enantioenriched Alcohols in Aqueous Medium. *Commun. Chem.* **2019,** *2*, 82.

29. Yu, Y.-C.; Sung, Y.-C.; Fu, J.-H.; Peng, W.-S.; Yu, Y.-C.; Li, J.; Chan, Y.-T.; Tsai, F.-Y., Nickel-Catalyzed Suzuki–Miyaura Coupling in Water for the Synthesis of 2-Aryl Allyl Phosphonates and Sulfones. *J. Org. Chem.* **2024,** *89*, 2448-2458.

30. Chen, G.-J.; Huang, J.; Gao, L.-X.; Han, F.-S., Nickel-Catalyzed Cross-Coupling of Phenols and Arylboronic Acids Through an In Situ Phenol Activation Mediated by PyBroP. *Chem. Eur. J.* **2011,** *17*, 4038-4042.

31. Heo, Y.; Hyun, D.; Kumar, M. R.; Jung, H. M.; Lee, S., Preparation of Copper(II) Oxide Bound on Polystyrene Beads and Its Application in the Aryl Aminations: Synthesis of Imatinib. *Tetrahedron Lett.* **2012,** *53*, 6657-6661.

32. Salamanca, V.; Toledo, A.; Albéniz, A. C., [2,2′-Bipyridin]-6(1H)-one, a Truly Cooperating Ligand in the Palladium-Mediated C–H Activation Step: Experimental Evidence in the Direct C-3 Arylation of Pyridine. *J. Am. Chem. Soc.* **2018,** *140*, 17851-17856.

33. Cheng, H.; Wu, Q. Y.; Han, F.; Yang, G. F., Efficient Synthesis of 4-Substituted Pyrazole *via* Microwave-Promoted Suzuki Cross-Coupling Reaction. *Chin. Chem. Lett.* **2014,** *25*, 705-709.

34. Marzabadi, M. R.; Wetzel, J.; Deleon, J. E.; Jiang, Y. Preparation of Substituted Anilinic Piperidines as MCH Selective Antagonists.WO2003/004027 A1. 2003.

35. Cervantes-Reyes, A.; Smith, A. C.; Chinigo, G. M.; Blakemore, D. C.; Szostak, M., Decarbonylative Pd-Catalyzed Suzuki Cross-Coupling for the Synthesis of Structurally Diverse Heterobiaryls. *Org. Lett.* **2022,** *24*, 1678-1683.

36. Cooper, A. K.; Burton, P. M.; Nelson, D. J., Nickel versus Palladium in Cross-Coupling Catalysis: On the Role of Substrate Coordination to Zerovalent Metal Complexes. *Synthesis* **2020,** *52*, 565-573.

37. Bhattacharya, D.; Studer, A., meta-Hydroxylation of Pyridines, Quinolines, and Isoquinolines Using Dearomatized Intermediates. *Angew. Chem. Int. Ed.* **2025,** *64*, e202423512.

38. Hsu, H.-H.; Kang, S.; Chen, C.-C.; Sk, M. R.; Thomas, A. A., Functionalization of Pyridines at the C4 Position via Metalation and Capture. *Angew. Chem. Int. Ed.* **2025,** *64*, e202424172.

39. Esteves, H. A.; Goldfogel, M. J.; Shemet, A.; Peng, C.; Hritzko, B.; Simmons, E. M.; Wisniewski, S. R., Advancing Base-Metal Catalysis: Developing Nickel Catalysis for the Direct Telescope of Miyaura Borylation and Suzuki–Miyaura Cross-Coupling Reactions. *Org. Process Res. Dev.* **2024,** *28*, 4039-4045.

40. Hornberger, K. R.; Snyder, L. B.; Wang, J. Rapidly Accelerating Fibrosarcoma Protein Degrading Compounds and Associated Methods of Use. WO2022/047145 A1. 2022.

41. Xiong, Y.; Schrader, T.; Chen, A.; Roppe, J. R.; Baccei, J. M.; Bravo, Y. Preparation of Heteroaryl Substituted Piperazine or Diazabicyclooctane Compounds as Muscarinic Acetylcholine M1 Receptor Antagonists. WO2019/241131 A1. 2019.

42. Lennox, A. J. J.; Lloyd-Jones, G. C., Selection of Boron Reagents for Suzuki–Miyaura Coupling. *Chem. Soc. Rev.* **2014,** *43*, 412-443.

43. Meringdal, J. W.; Menche, D., Suzuki–Miyaura (Hetero-)Aryl Cross-Coupling: Recent Findings and Recommendations. *Chem. Soc. Rev.* **2025,** *54*, 5746-5765.

44. Seen, A. J.; Cavell, K. J.; Mau, A. W. H.; Hodges, A. M., Ethene Dimerization Using a Nafion® Supported σ-Arylnickel(II) Species. *J. Mol. Catal.* **1994,** *90*, 245-256.

45. Collins, K. D.; Glorius, F., A Robustness Screen for the Rapid Assessment of Chemical Reactions. *Nat. Chem.* **2013,** *5*, 597-601.
